# Technical note: a functional data analysis approach to analyze the light-adapted electroretinogram in children and adolescents

**DOI:** 10.1007/s10633-026-10112-y

**Published:** 2026-05-27

**Authors:** Zdeněk Hlávka, Paul A. Constable, Fernando Marmolejo-Ramos, Lynne Loh, Dorothy A. Thompson, Jan Kalina, Marco Marozzi

**Affiliations:** 1 Faculty of Mathematics and Physics, Department of Statistics, Charles University, Prague, Czech Republic; 2 College of Health and Enablement, Flinders University, Bedford Park, Adelaide 5000 Australia; 3 College of Human Sciences and Culture, Flinders University, Bedford Park, Australia; 4 The Tony Kriss Visual Electrophysiology Unit, Clinical and Academic, Department of Ophthalmology, Great Ormond Street Hospital for Children NHS Trust, London, UK; 5 UCL Great Ormond Street Institute of Child Health, University College London, London, UK; 6 Department of Mathematics and Computer Science, University of Ferrara, Ferrara, Italy

**Keywords:** Oscillatory potentials, Time series, Waveform analysis, Multivariate

## Abstract

**Purpose:**

To present a new framework for the analysis of the light-adapted electroretinogram using Functional Data Analysis (FDA).

**Methods:**

Light adapted full-field electroretinograms and extracted Oscillatory Potentials waveforms were analyzed using an FDA approach. Waveforms from 71 individuals with autism spectrum disorder and 98 Control participants (mean ± SD age in years): ASD (12.8 ± 4.4) and Control (13.5 ± 4.7) were reanalyzed from previous studies. Robust and powerful nonparametric permutation-based tests were developed for the mean, variance, and first derivative of the waveforms.

**Results:**

FDA identified a significant group difference (p=0.049) for the mean of the waveforms between the a-wave minima and b-wave maxima for the 356 and 445 Td.s flash strengths. The Oscillatory Potentials mean and covariance for the mean and first derivative of the waveforms exhibited significant group differences across multiple flash strengths (p=.0244). The combined test statistic for the mean and covariance showed group differences by age and group, but not by sex.

**Conclusion:**

By treating time-series recordings as functions, the proposed FDA framework enables a more informative assessment of group differences through the analysis of mean structure, covariance, and waveform dynamics captured by first derivatives. This new functional perspective extends beyond traditional time series methods based on peak time and amplitude to provide a powerful tool for uncovering subtle differences between groups.

**Supplementary Information:**

The online version contains supplementary material available at 10.1007/s10633-026-10112-y.

## Background

The full-field electroretinogram (ffERG) is a complex waveform derived from retinal photoreceptors and neurons in response to a flash of light [1]. The light adapted (LA) ffERG waveform is cone driven. The early negative deflection, the a-wave, is formed by cone photoreceptor hyperpolarization and second order neurons including horizontal cells [2, 3]. The following positive deflection, the b-wave, is dominated by bipolar and glial cells [4–8] contributions, with high frequency components (the Oscillatory Potentials (OPs)) apparent as small ripples on the ascending limb of the b-wave thought to derive from amacrine cells [9]. Following the b-wave, the photopic negative response is formed by ganglion cells [10]. The integration over time of relative contributions of the neural generators has stimulated the application of several analysis methods aiming to capture the nuances of the ffERG waveform. These may provide additional ways to quantify and interpret the ffERG beyond the peak amplitudes and timings of the principal components that are described in the International Society for Clinical Electrophysiology of Vision (ISCEV) clinical ffERG Standard [1].

**Fig. 1:**
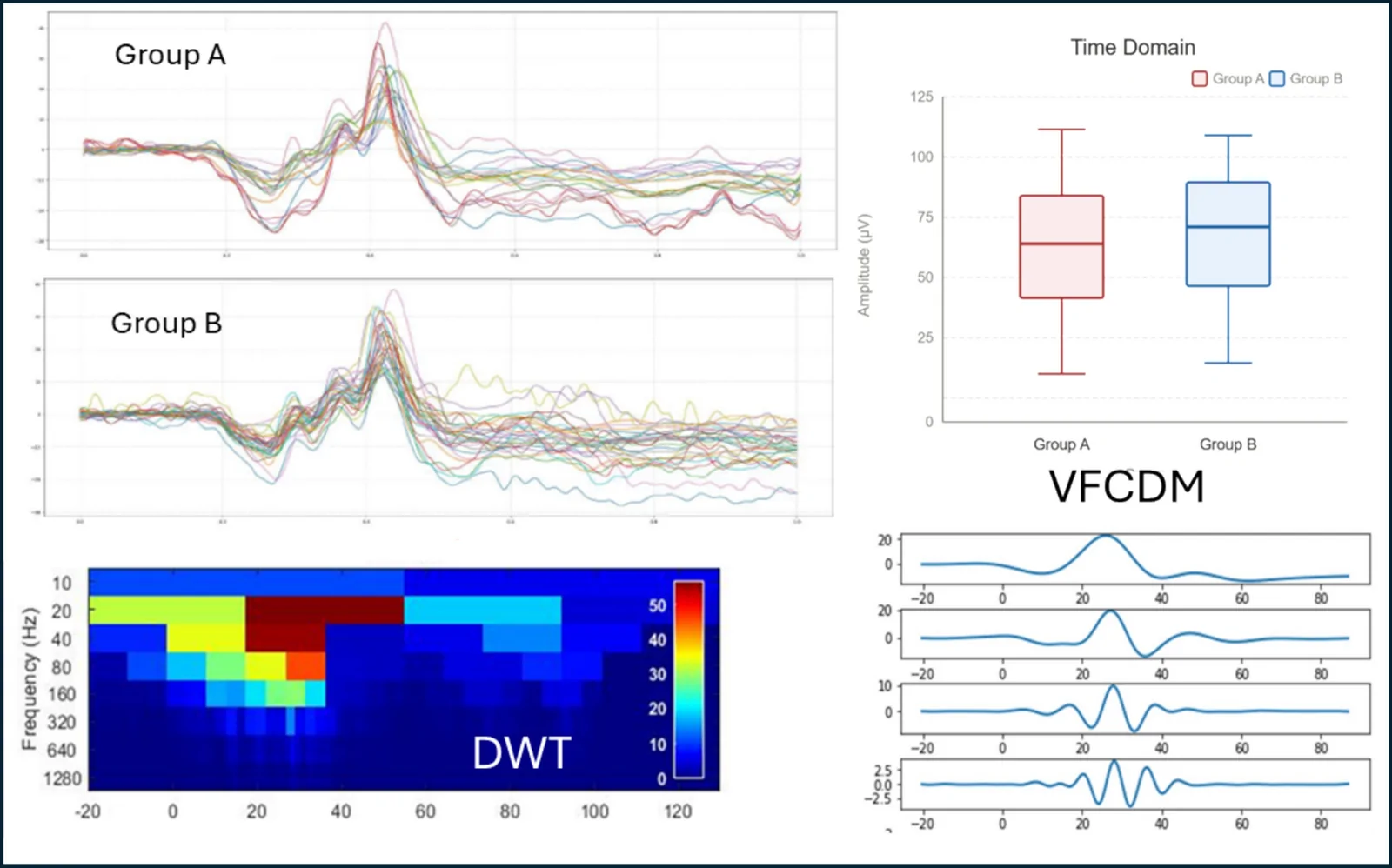
Methods for analyzing properties of the electroretinogram waveform include comparing medians of time domain parameters such as the a- and b-wave amplitudes and times to peak between groups which provide information about the waveform at a discrete time point. Spectral domain analysis enables a richer dataset by decomposing the waveform into discrete time windows centered on clinically relevant frequency bands such as Discrete Wavelet Analysis (DWT) or into a selection of bandpass filters using the Hilbert Transform to display how the amplitude envelope varies over time within a frequency band which is known as Variable Frequency Complex Demodulation (VFCDM)

Mathematical models of the ffERG waveform have focused mainly on the a-wave using exponential functions [11, 12] to characterize phototransduction kinetics [13]. Time–frequency analyses have been used to describe the energy contributions of different parts of the ffERG waveform. One such method is wavelet transforms, which decomposes the signal into different frequency components and identifies when they occur. [14] Continuous wavelet transforms include redundant continuous information which helps visualization whilst discrete wavelet transformations remove noise to focus on region of interest providing temporal resolution of the a- and b-waves and the high frequency OPs and energy within the ON- and OFF-pathways [15–18]. Variable Frequency Complex Demodulation is alternative to discrete wavelet analysis and provides a higher resolution due to the ability to use a greater number of frequency bands with which to decompose the ffERG waveform into. However, this method does not provide any temporal localization of the waveform [19, 20]. The OPs are a complex series of peaks and troughs that are traditionally reported as the sum of the main peaks and time to peaks [1]. Modelling using a combination of a Gaussian function that envelopes the OPs and sine wave carrier have been proposed to model the OPs across species and provides a general overview of the OPs shape but not whether a specific peak is atypical [21]. To better quantify and analyze the individual OPs in greater detail Gauthier et al. (2025) [22] have proposed the use of the Hilbert Transform (which when multiplied by the OPs waveform produces an analytic signal that provides important information on individual peaks such as their time to peaks and main peak time differences) unlike discrete wavelet transform, and the Gaussian envelope approaches that provide a global measures of the OPs energy [16, 17] and peak amplitude [21]. These approaches are summarized in Fig. 1. In contrast to these methods, Functional Data Analysis treats the amplitude and time as x- and y- co-ordinates and creates a function from these points with which to describe how the mean (size) of the function varies, or the variance (spread) over time. The function can be defined within a region of interest of the waveform (a-wave, b-wave or OP2) for example or the entire waveform can be treated as a single observation giving some flexibility in the type of analysis required.

The main objective of this report was to provide a background into FDA which has been applied to datasets in epidemiology [23], medicine [24] and biophysics [24], but to date has not been applied to visual electrophysiology. FDA is an active branch of statistics that focuses on analyzing data in the form of continuous functions or curves defined over a continuum (such as time) instead of as isolated discrete x, y co-ordinates [25, 26]. In an FDA framework, each observation (e.g., each subject’s measurements) is an entire function—for example, a smoothly varying curve over time—which allows examination of the shape of the waveform trajectories and their dynamics as a continuous function of time and amplitude (Wang et al., 2016). This approach makes it possible to capture both what and when changes occur (slope, mean or covariance), and not just whether an outcome differs at a specific time points such as the a- and b-wave trough or peak. Statistical approaches have used aspects of FDA, notably to model the ascending portion of the b-wave [27, 28] and smoothing splines have been used to provide a contour plot of the initial portion of the ffERG as part of the luminance response function [29].

The implementation of splines to fit the function is a key aspect of FDA. In practice multiple polynomials are fitted to small subsets of the waveform forming together the piecewise polynomial spline function that interpolates and smooths the shape of the waveform. So rather than a single mathematical expression representing the waveform the spline function is a composite of polynomials connected at points to smoothly represent the varying nature of the underlying waveform [30].

FDA enables the complex waveform shape of the ffERG and OPs to be treated as a single observation defined as a spline that best fits the waveform’s shape. In this scenario the comparison of the amplitude of the waveforms was determined by comparing the mean values of the functions either between groups (ASD vs Control) or within a group such as the effect of age or sex within the ASD population. The second comparison was between the functions covariance- which in FDA encompasses two parts. One the spread or variance of the function that is the variance about the mean and secondly how the trajectories of the function follow the same path or co-vary across time. Measures of mean and covariance differences can then be applied to the waveform function and to the first derivative of the waveform function (its slope) to assess how the mean and covariance differ for the waveform function and its first derivative. In summary, the mean function describes the average curve of the dataset and highlights differences in the waveform’s amplitude. The covariance operators describe how different points in time along the waveform vary together and how much spread in the data there is about the mean. This enables a model of curve-to-curve variability, and the first derivative reveals the slope of the waveform which is needed to identify inflection points at peaks and troughs.

We performed and visualized various two-sample comparisons using a general measure transportation-based approach to permutation tests [31]. This nonparametric statistical method extends traditional permutation tests that ‘shuffle’ the data thousands of times but do not extend naturally to multidimensional tests (which are composed of multiple test statistics—hence ‘multidimensional’). The advantages of permutation tests are that they make no assumptions about the normal distribution of the data, they handle complex functional data naturally and are reliable in small samples. The measure transportation-based permutation tests additionally allow us to work with multidimensional test statistics and to calculate the contributions of marginal components of the test statistic (studied aspects) to the test result. Taken together, these properties enable inferences to be made on the full functional profile of the ffERG, capturing subtle and potentially clinically meaningful differences that may not manifest at the peaks or troughs alone. By analyzing the whole dataset rather than data summaries, the method is data efficient (extracting more information from each recording), a central consideration given the resource-intensive nature of ffERG acquisition and the need to further utilize the potential of the ffERG in clinical testing [32]. This aspect represents an important advantage, enabling clinicians to obtain more insights from existing recordings without needing additional recordings, thereby optimizing resource allocation in clinical settings. This comprehensive use of the data is an important methodological novelty in the field and opens the door to a richer characterization of retinal function, especially in early or subclinical disease stages where changes may be distributed across the waveform rather than concentrated at specific peaks and troughs in retinal and neurological conditions [10, 33–36].

A key innovation of this FDA-based approach lies in its ability to assess overlooked aspects of the waveform, such as the first derivative which provides valuable information on the rate of change in the retinal response dynamics. The first derivative can reveal subtle alterations in signal morphology that may correlate with early pathological changes, offering a more sensitive diagnostic tool. Furthermore, the method computes the impact of the studied aspects on group comparisons, identifying the most discriminating features among means and variability. In this example the functions of the mean (amplitude), covariance and first derivative (slope) of the waveforms were used to compare group differences and to calculate the relative contribution of the mean and covariance or first derivative to the overall test significance.

## Methods

### Dataset

The dataset has been described in detail previously [37, 38] and in this case consisted of LA-ffERGs recorded in 71 autism spectrum disorder (ASD) and 98 Control participants using the RETeval (LKC Diagnosys, Germantown, USA) hand held device with skin electrodes. Two groups were included in this example to illustrate a case–control comparison and a within group application of FDA. White flash strengths were 12, 21, 35, 70, 85, 113, 178, 251, 356 and 446 Td.s on a 1130 Td or 848 Td white background for the 85 Td.s flash strength. The mean ± SD age of the ASD group was 12.8 ± 4.4 years and for the Control group 13.5 ± 4.7 years (Mann–Whitney U-test, *p* = 0.21). The ASD group contained 57 male and 14 female participants and for the Control group there was 56 male and 42 female participants (Chi-Squared *p* < 0.001). There were no significant differences between groups for the position of the electrode height (Mann–Whitney U-test *p* = 0.23) or for iris color (Mann–Whitney U-test *p* = 0.12) with mean ± SD iris color for the ASD group (1.21 ± 0.10) and the Control group (1.24 ± 0.13). Within the ASD group (n = 48) the full scale intelligence quotient was 100 ± 16 with a mean ASD severity (n = 57) score of 6.1 ± 2.3) [39]. For this analysis one recording per eye was incorporated into the study based on waveform quality (baseline drift, noise) and the OPs waveforms were extracted from the ffERG waveform using a 80–300 Hz filter with RFF Extractor software (LKC Diagnosys, Germantown, USA). For the ASD group total of 654 ffERG and 450 OPs waveforms were extracted and for the Control group a total of 868 ffERG and 789 OPs waveforms were extracted (See Table 1). The extraction of a complete array of the OPs waveforms was not always successful owing to the software unable to perform the *post-hoc* filtering of the averaged waveform.

**Table 1:** Summary of the number of waveforms (n) included in the analysis for the Oscillatory Potentials and electroretinograms (right eye only) for each group at the ten flash strengths

Flash strength (Td.s)	Electroretinogram (n)		Oscillatory potentials (n)	
	Control	ASD	Control	ASD
12	84	61	75	42
21	86	67	79	46
35	86	66	77	45
70	89	67	80	47
85	79	57	76	38
113	88	67	80	47
178	90	69	80	47
251	88	66	80	45
356	89	67	81	46
446	89	67	81	47
Total	**868**	**654**	**789**	**450**

In this analysis portions of interest of the ffERG were treated separately to highlight the potential of FDA to isolate time intervals of interest within the overall waveform. Notably from t = 0 to the a-wave minima (Part A); from the a-wave minima to the b-wave maxima (Part B) and from the b-wave maxima to t = 72 ms (Part C). These regions corresponded to the main portions of the ffERG that may be of clinical relevance. The OPs waveform was examined between t = 0 and 60 ms. Here we report the right eye results for the mean, covariance and first derivatives for Part B and the effect of sex and age on the OPs for the ASD group as exemplars. Group comparisons for Part A is included to show a separate region of interest analysis. See Supplementary Material for complete results of all analyses.

### Functional data analysis

We were interested in two-sample comparisons of mean functions and covariance operators of the observed functions and their derivatives. Due to the complex nature of functional test statistics, permutation methods are the standard approach for assessing significance in FDA, as they avoid strict parametric assumptions that are rarely satisfied in practice. [40, 41]. As recommended in [42] we used the maximum of pointwise calculated F-tests (Fmax) for comparisons of mean functions [43] and the square-root distance (SQ) for testing equality of the covariance operators [44]. Since the time coordinates were not identical for all functional observations, we used spline interpolation [45] to normalize the time coordinates by transforming all functional observations into the same grid. The analysis was performed in the R-statistical computing environment [46].

**Fig. 2:**
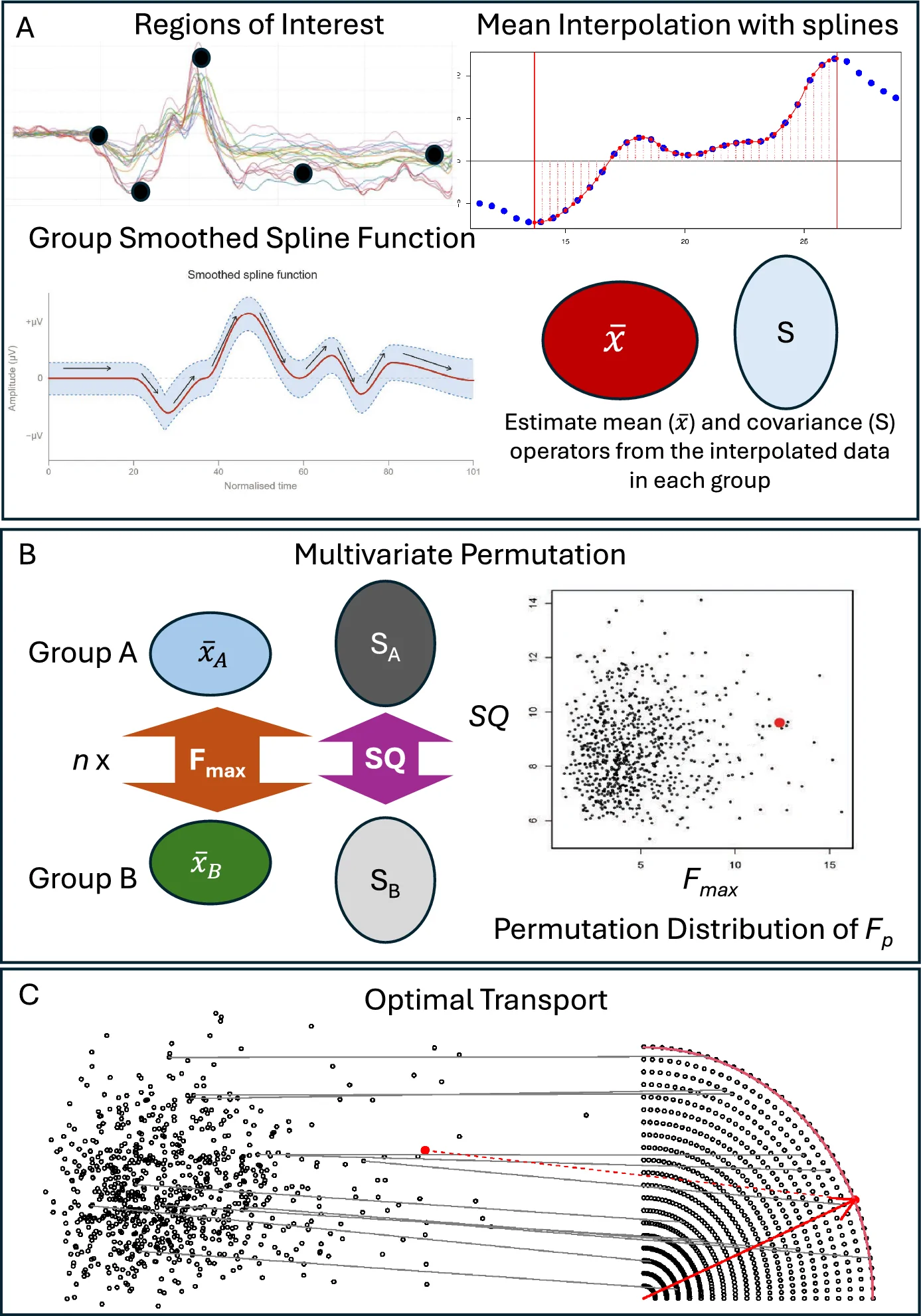
(**A**) Regions of interest or the whole waveform can be analyzed and selected as indicated by the group’s waveforms to explore the a-wave, b-wave or later regions of the electroretinogram wave form. The mean interpolation of splines uses short polynomial strings to fit the mean of the group’s waveforms. This enables interpolation (red points) along the function that is fitted to the observed data (blue points). In this example Part A (a-wave region of interest) is shown for one subject. From the smoothed function two arrays of data are generated represented by *F*_*max*_ (amplitude) and SQ (covariance represented by the light shaded area and the trajectories represented by the small arrows). These datasets can then be compared between groups using multivariate permutations as shown in (**B**). For the multivariate permutation each group’s data for *F*_*max*_ and SQ are randomly shuffled between each group label (ASD or Control in this case) and for each permutation the test statistic (F_p_) is calculated. The values of SQ and *F*_*max*_ for each permutation can be plotted as the permutation distribution. (**C**) To evaluate the significance of the observed test statistic (F_T_) compared to the permutation distribution of F_p_ optimal transportation is used to transform the values of F_p_ to a spherical grid so that visualization S_T_ (red arrow, right) of the observed test statistic F_T_ (red point, left) can be placed within the grid to determine its significance. If the value of S_T_ falls outside in this case the 95th centile (red circle, right) then there is a significant group difference with the values of x representing the contribution of *F*_*max*_ and y the contribution of SQ to the overall significance

To visualize the test results and to perform (1) simultaneous two-sample comparisons of mean functions and covariance operators and (2) simultaneous two-sample comparisons of means for the wave function and its first derivative, we used the empirical measure transportation-based approach to multivariate permutation tests. See Sect. 6 in Hlávka et al. (2025) [31]. The *transportation-approach* allows to observe the multivariate rank of the (multidimensional) test statistic with respect to the permutation distribution and to define the corresponding p-value naturally. The greater the multivariate rank the smaller p-value and the more significant the test statistic becomes. Statistically we are asking if we randomly shuffle data points between groups and calculate the test statistic for each permutation (F_p_) to construct the permutation distribution of F_max_ and SQ we can then evaluate F_T_ (the true test statistic) to see where the test statistic falls within the permutation distribution. If both groups have the same distribution for their data, then shuffling data between groups using Monte Carlo will result in F_p_ having the same probability distribution as F_T_. Here to observe the p-value of F_T_ with respect to F_p_ we used the transportation approach to ‘transport’ the values of F_p_ onto a spherical grid so the permutation distribution is represented for easier visualization/representation of the value of F_T_ and the respective contributions of F_max_ and SQ. Figure 2 provides a flow chart to illustrate the steps in FDA, spline smoothing and the transportation of the permutation distribution to a spherical grid.

### Mean and covariance

**Fig. 3:**
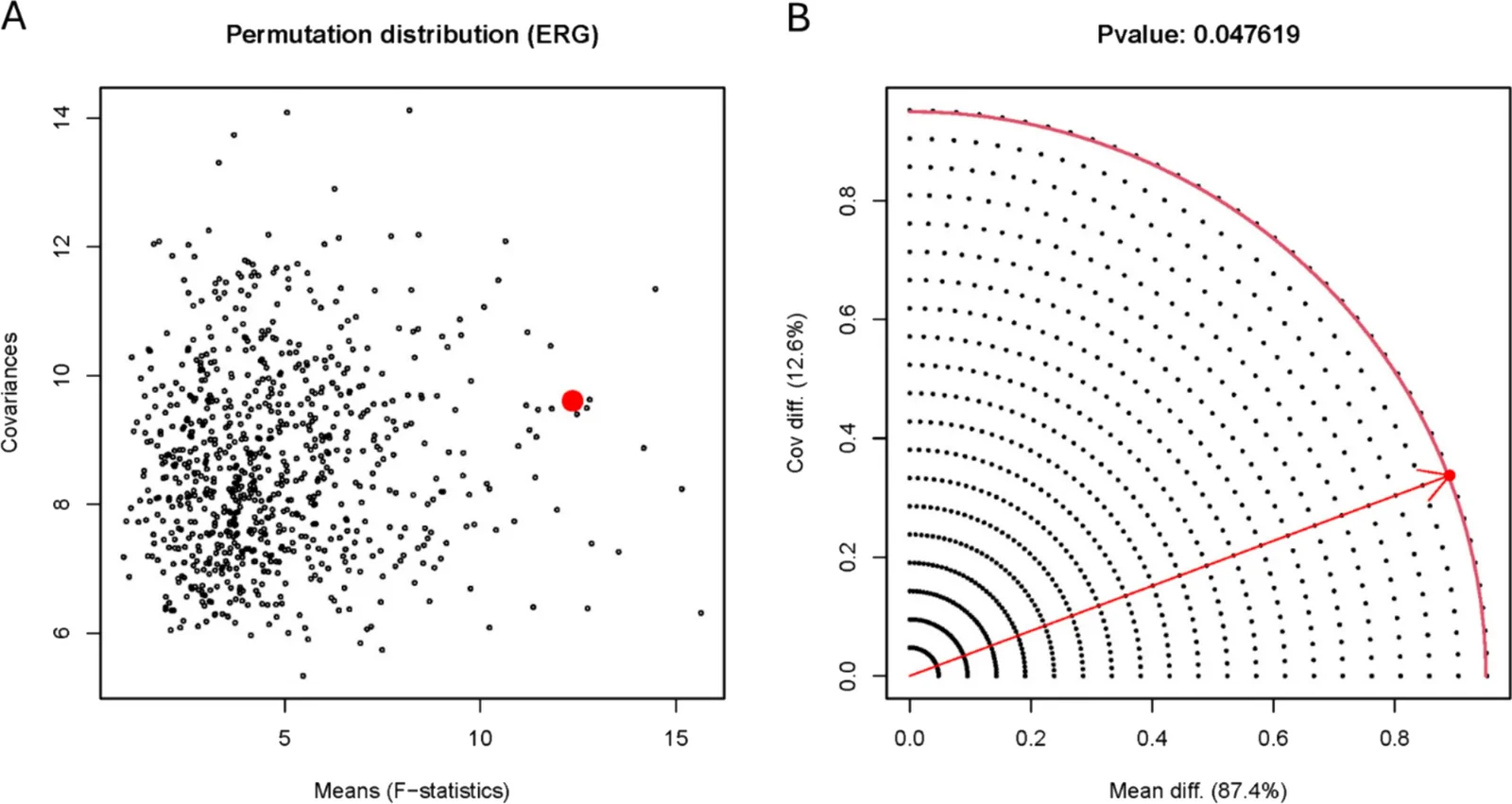
Permutation distribution of the 70 Td.s flash strength of the ffERG waveform for right eye. Figure A shows the permutation distribution and B shows the transformed distribution to a spherical grid with x-axis the mean difference between groups and the y axis the covariance difference between groups. The grid extends to 20/21 = 0.9524 so that if the red vector passes the critical limit of the grid represented by the red circle of radius (1—0.05), then a significant difference is reached. In this case, 1—0.9524 = 0.0476 is the smallest possible p-value. The squared values of the x and y axis, S_1_^2^ = 0.793 and S_2_^2^ = 0.114, provide the absolute contributions of the two test statistics to the test result, corresponding to the p-value 1-(S_1_^2^ + S_2_^2^) ^0.5^ =.0476 and relative contributions 87.4% and 12.6%

To showcase the capability of our framework we tested for equality between the distributions of the FDA functions for the ASD and Control groups 70 Td.s flash strength and right eye. We analyzed the data set as is, i.e., without the spline interpolation so that each curve was represented by 101 points corresponding to an equally spaced grid from approximately t =  − 20 ms to t = 31 ms. The test statistic combined (1) the F_max_ test statistic for equality of the mean functions (amplitude) and (2) the test statistic SQ for the equality of covariance operators (variance). See Fig. 3. The observed bivariate test statistic (F_T_) (red point) was compared to the null distribution obtained by permutations and in this case its significance was evaluated using the ‘center-outward distribution function’ [47], i.e., by transforming the permutation distribution (Fig. 3A) to a suitable spherical grid (Fig. 3B) using the empirical measure transportation approach. The arrow denotes the value S_T_ = (S_1_, S_2_)^T^ of the empirical center-outward distribution function corresponding to the observed test statistic: F_T_ = (F_1_, F_2_)^T^. From this notation, the p-value can be calculated as: 1—∥S_T_∥, i.e., as the probability mass of the permutation distribution that is more extreme than the observed test statistic from the center-outward perspective.

In this case, the *p*-value was 0.045 and the equality of the two functional distributions was rejected at a 5% significance level. See also that: ∥S_T_∥^2^ = S_1_^2^ + S_2_^2^, where S_1_ (x axis) corresponds to the first test (F_max_, equality of means) and S_2_ (SQ, y axis) corresponds to the second test (equality of covariance operators). Therefore, S_1_^2^/∥S_T_∥^2^ and S_2_^2^/∥S_T_∥^2^ can be interpreted as the relative contributions of the two marginal tests to the test decision: in this example, the difference in means was more important, because the relative contribution of the observed mean differences was 87.4%. This interpretation of the relative contributions of the mean and variance to the test decision allows the conclusion that the null hypothesis is rejected mainly because of differences in mean functions with a relative contribution of 87.4%, shown by the red arrow pointing closely to the direction of the x axis, with a minimal contribution of covariance (12.6%).

Note that we reject the null hypothesis at a 5% significance level if ∥S_T_∥^2^ = S_1_^2^ + S_2_^2^ ≥ (1—0.05)^2^ and, therefore, the values S_1_^2^ and S_2_^2^ can be interpreted as the absolute contributions of the two marginal tests to the overall test results, with the null hypothesis being rejected if and only if the sum of these absolute contributions is large enough. In this case the absolute contributions of the Mean and Covariance difference were S_1_^2^ = 0.793 and S_2_^2^ = 0.114, respectively, corresponding to the *p*-value of 1-(S_1_^2^ + S_2_^2^) ^0.5^ = 0.0476 for the combined test statistic.

**Fig. 4:**
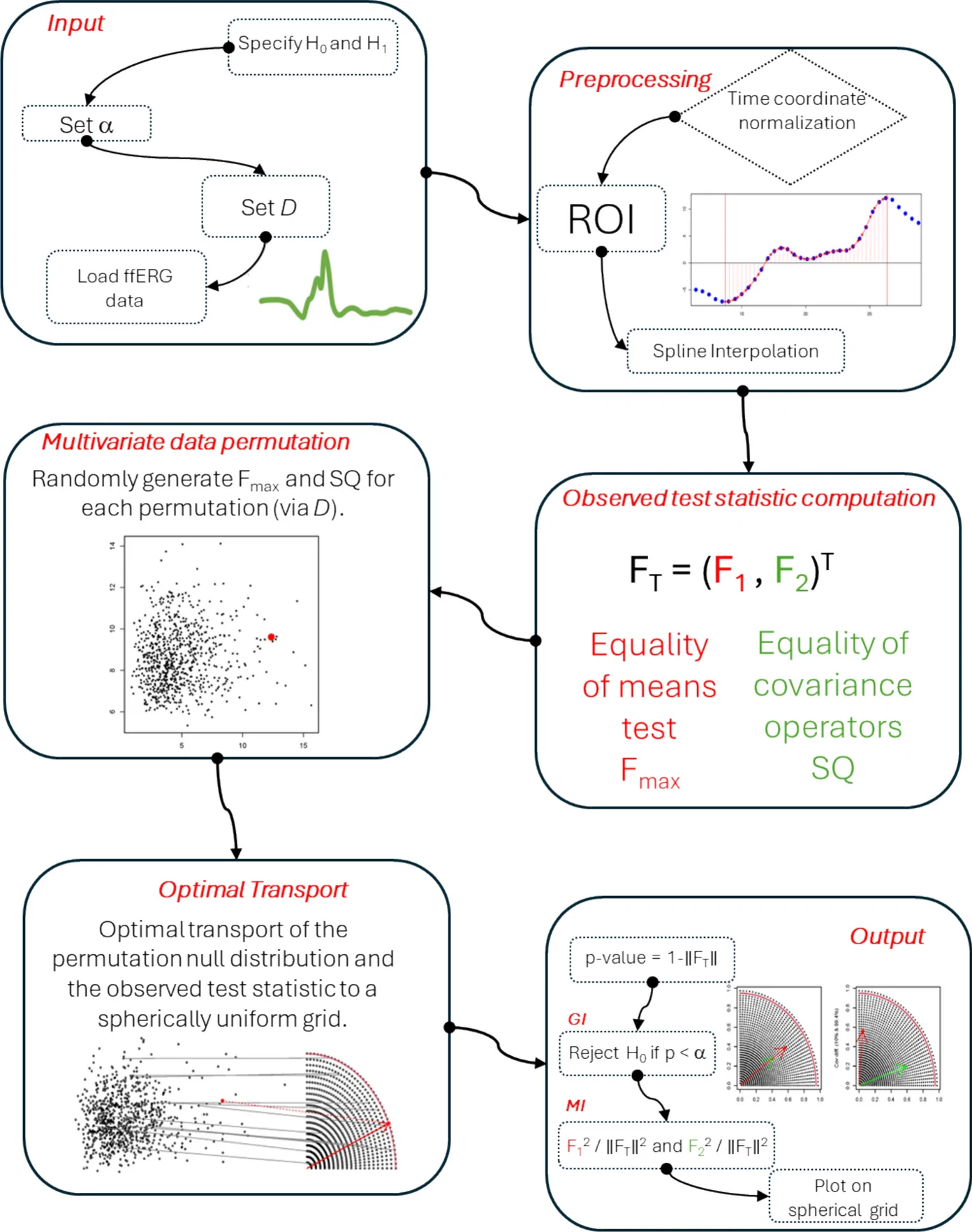
The main stages in FDA and the multivariate analysis. The input is the group waveforms where a decision the appropriate p-value and number of permutations (D) to be performed are made. The time scale is normalized, and regions of interest (ROI) are selected within the waveform for spline interpolation (SI) and smoothing. From the smoothed splines, multivariate permutations generate possible values for the test parameters (F_max_ – mean and SQ – square root for covariance operators). The values of F_max_ and SQ are then transposed to a spherical grid using the optimal transport approach with the test of significance for the observed test statistic (F_T_) visualized for SQ and F_max_. GI is the Global Inference relating to F_T_ and the MI is the Marginal Inference is looking at more detail at the contributions of F_max_ and SQ to the global significance

Figure 4 provides a summary of the methods as a flow chart highlighting the main steps in obtaining the results.

## Results

**Fig. 5:**
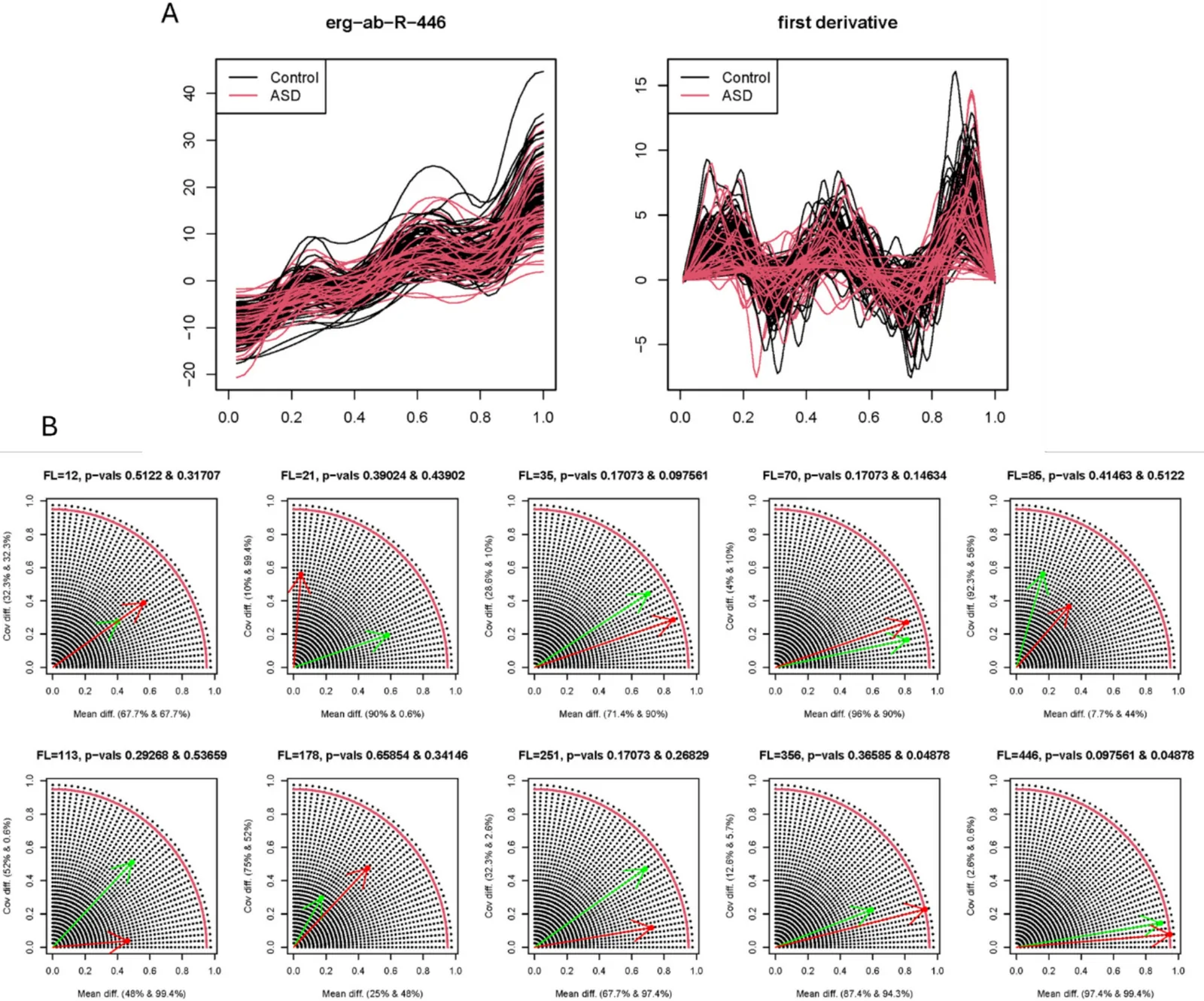
**A** A shows a representative plot for the 446 Td.s Flash strength electroretinogram between the a-wave minima and b-wave maxima with the waveform function and first derivative. Time axis normalized (grid size 40 for the wave function and 120 for the first derivative). **B** shows the grid plots for all flash strengths with red arrows, the mean and green arrows the perimeter set at the confidence level of 0.05. The x axis represents the contribution to the test statistic’s mean and the y axis the covariance. Thus, the grid plots provide a visual representation of the overall significance based on the mean function and first derivative and relative contributions of mean (amplitude) -x axis and covariance -y axis

Figure 5A illustrates the waveform mean function and the first derivative of the interval between the a-wave minima and b-wave maxima (Part B) with grid plots indicating significance contributions of mean and covariance. In this case there was a significant difference between the control and ASD groups in the first derivative for the 356 and 446 Td.s strengths with the main contribution (> 92%) from the mean difference (*p* = 0.049). Note the plot representing the first derivative that captures the changing slope of the waveform.

The grid plots (Fig. 5B) provide a visual representation of the relative contributions to the overall significance based on the contributions of mean (amplitude) and covariance of the functions. The resultant arrow or ‘vector’ in the grid plot is the resultant vector of the contributions of covariance (y axis) and mean (x axis). In the plots below the red arrow represents the mean functions and the green arrow the first derivative or change in amplitude with time of the waveform function. Significance is reached if the arrow reaches the boundary of the grid that in this case is set at 0.95 representing a p-value of 0.05. When observing the red arrow in the dimmer flash the arrow “significance” has a greater contribution from covariance than mean with the arrow closer to the y-axis. In contrast the stronger flashes represented by the last two plots are influenced by a mean difference in the functions and was closer to the x-axis. Thus, the grid plots provide insights into the mean values of the ffERGs and their covariance based on the mean function (red arrow) and its first derivative (green arrow).

**Fig. 6:**
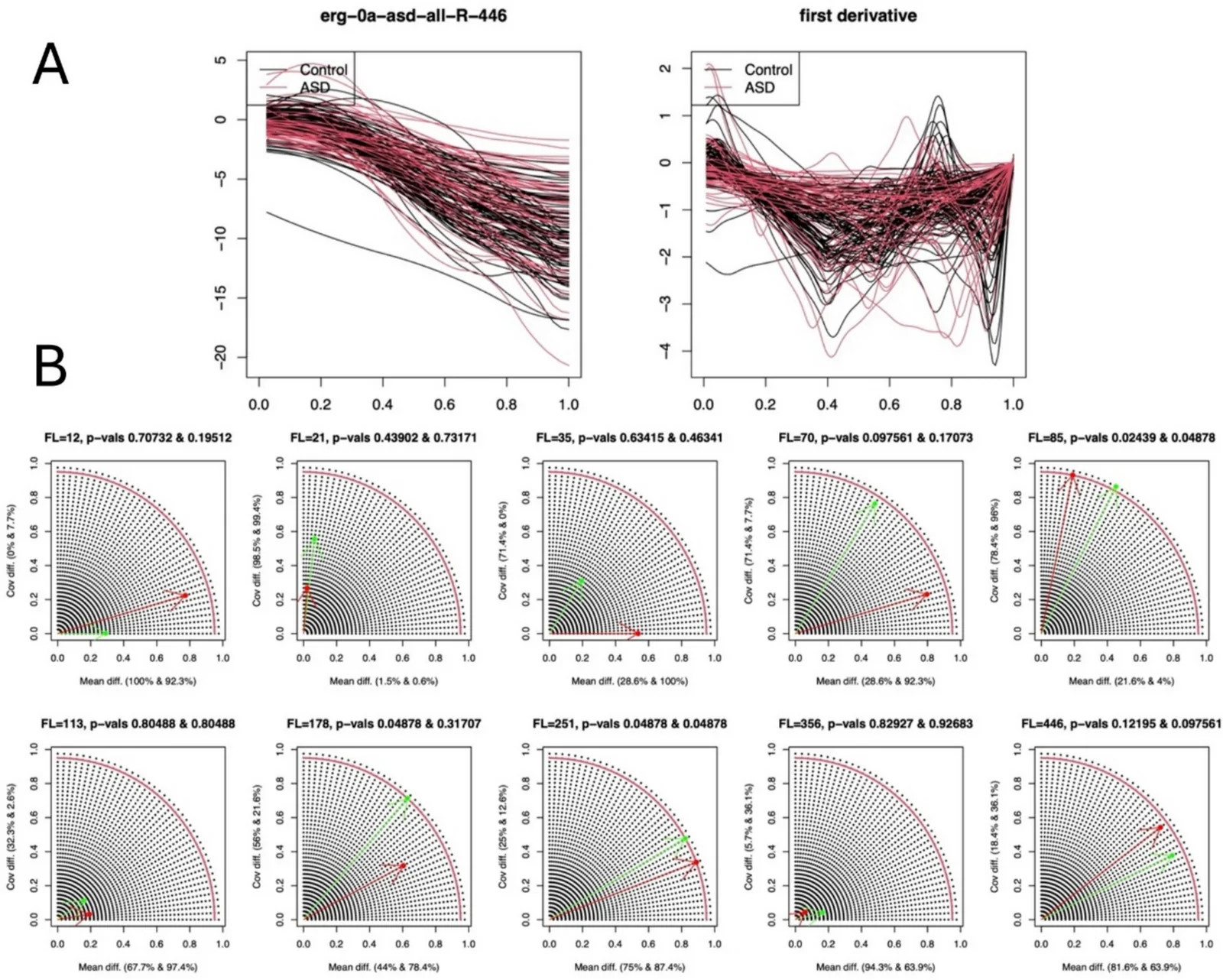
**A** Representative plot for the 446 Td.s Flash strength electroretinogram between the baseline and a-wave minima with the waveform function and first derivative. Time axis normalized (grid size 40 for the wave function and 120 for the first derivative). **B** The grid plots for all flash strengths with red arrows, the mean and green arrows the perimeter set at the confidence level of 0.05. The x axis represents the contribution to the test statistic’s mean and the y axis the covariance. Thus, the grid plots provide a visual representation of the overall significance based on the mean function and first derivative and relative contributions of mean (amplitude) -x axis and covariance -y axis

To further illustrate the application of FDA to focus on specific regions of the waveform, Fig. 6 illustrates the corresponding plots for (Part A) which contain the a-wave. We observe significant differences for some flash strengths, but a closer inspection of the waveform plots reveals that this could be due to presence of outliers.

### Oscillatory potentials

**Fig. 7:**
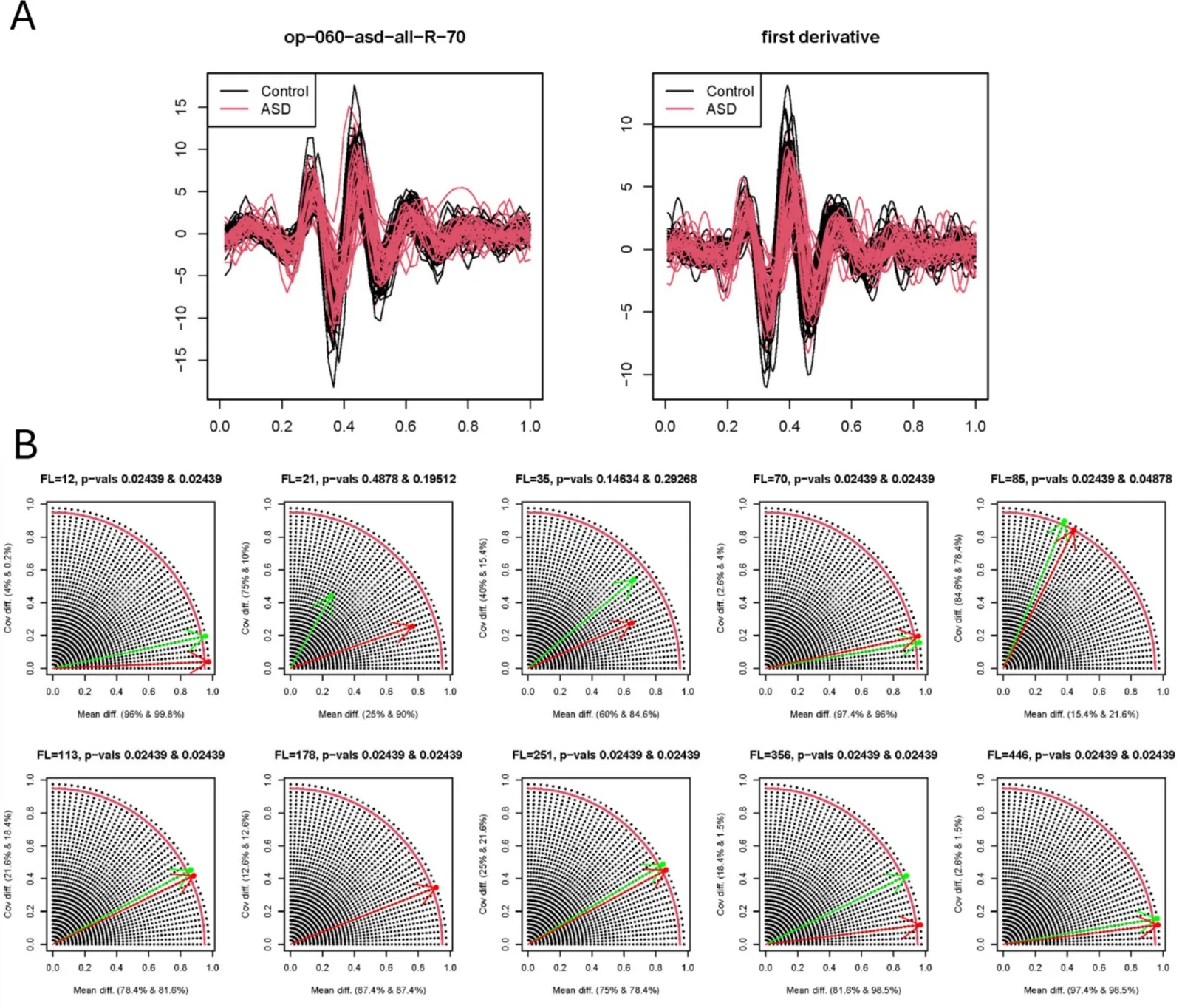
**A** Representative plot for the 70 Td.s Flash strength oscillatory potentials with the waveform function and first derivative. Time axis normalized (60 grid points for the wave function and 150 grid points for the first derivative). **B** Grid plots with red arrow the mean and green arrow the first derivative for each flash strength

The OPs waveform functions were assessed for group differences in mean and first derivative in the time window 0–60 ms and are shown in Fig. 7.

### Age

This example shows the analysis using the mean OPs function and its first derivative to evaluate age as a factor for *all participants*. In this case the participants were divided into two arbitrary groups: either young < 12 years of age or older ≥ 12 years of age. A significant group differences (*p* = 0.024) was observed with the main contribution arising from the mean values in all but the 85 Td.s strength where covariance was the dominant contributor to the p-value. (Table 2).

**Table 2:** Summary statistics for the mean waveform function and the first derivative of the Oscillatory Potentials for the ten flash strengths. Significant group differences were observed across most flash strengths for each analysis with the percentage contribution indicated for each p-value. Blank cells indicate a non-significant result

Strength (Td.s)	*p -*value (mean)	Mean (%)	Covariance (%)	*p -*value (1st derivative)	Mean (%)	Covariance (%)
12	0.0244	96.0	4.0	0.0244	99.8	0.2
21	0.4878			0.1951		
35	0.1463			0.2927		
70	0.0244	97.4	2.6	0.0244	96.0	4.0
85	0.0244	15.4	84.6	0.0488	21.6	78.4
113	0.0244	78.4	21.6	0.0244	81.6	18.4
178	0.0244	87.4	12.6	0.0244	87.4	12.6
251	0.0244	75.0	25.0	0.0244	78.4	21.6
356	0.0244	81.6	18.4	0.0244	98.5	1.5
446	0.0244	97.4	2.6	0.0244	98.5	1.5

### Combined mean and first derivative test statistic

**Fig. 8:**
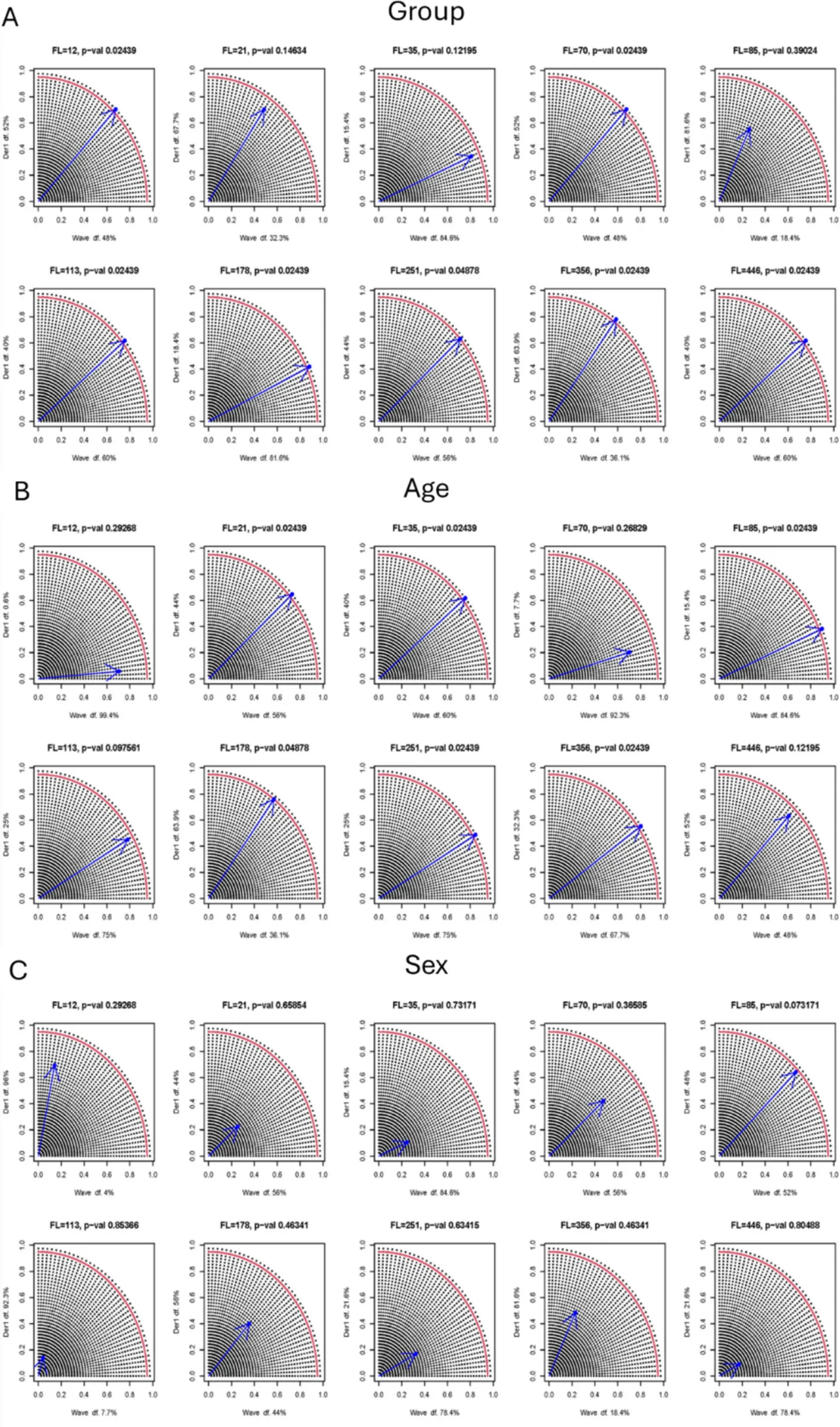
Grid plots of the oscillatory potentials combining the test statistic Fmax for mean and the first derivative (blue arrow). **A** shows the ASD vs Control comparison with **B** and **C** showing the results for the ASD group only that test for Age (less than 12 years or older than 12 years of age) or sex (male or female) respectively

Figure 8 illustrates a possible analysis where a single test statistic represents the contribution of the mean and first derivative combining amplitude and slope as contributors to the overall difference with percentage contributions calculated and shown in Table 3. In this case the test statistic was calculated for ASD vs Control and within the ASD group solely to illustrate a within group analysis for age (with 12 years as the arbitrary cut-off between childhood and early adolescence) as previously defined) or sex (male or female) as the factors for the OPs.

**Table 3:** Summarizes the significant differences for group (ASD vs Control); Age and Sex (within the ASD group). Blank cells indicate non-significant results. There were no significant differences based on sex within the ASD group and so the mean and 1 st derivative are not reported in the table

Strength (Td.s)	*p-* value Group	mean (%)	1 st Derivative (%)	*p-* value Age	Mean (%)	1 st Derivative (%)	*p -* value Sex
12	0.0244	48.0	52.0	0.2927			0.2927
21	0.1463			0.0244	56.0	44.0	0.6585
35	0.1220			0.0244	60.0	40.0	0.7317
70	0.0244	48.0	52.0	0.2683			0.3659
85	0.3902			0.0244	84.6	15.4	0.0732
113	0.0244	60.0	40.0	0.0976			0.8537
178	0.0244	81.6	18.4	0.0488	36.1	63.9	0.4634
251	0.0488	56.0	44.0	0.0244	75.0	25.0	0.6341
356	0.0244	36.1	63.9	0.0244	67.7	32.3	0.4634
446	0.0244	60.0	40.0	0.1220			0.8049

## Discussion

The objective of this technical report was to introduce FDA and multivariate test statistics as potential tools in the analysis of the ffERG and related visual electrophysiological waveforms whose shape and slope can be defined based on FDA analysis [1, 48, 49]. Spline interpolation allows the normalization of the time coordinates across different functional observations and calculate derivatives. The bivariate test statistic derived from the FDA analysis and the bivariate permutation test enable simultaneous comparison of the overall mean and covariance differences between waveforms and their percentage contributions to the final test statistic. Theoretically, more dimensional test statistics could also be considered, with all calculations and formulas extending naturally, but the visualization of test results would become more complicated. This approach can be applied to regions of interest within the waveform such as the a-wave, b-wave or PhNR regions or the entire waveform. An important advantage of transforming the ffERG into a function is that the waveform can be treated as any mathematical function, allowing derivatives to be computed, such as the first derivative analyzed here, to assess changes in slope and dynamic features across the entire or part of the waveform. This not the first time that the first derivative has been applied to the analysis of the ffERG. Lachapelle and Molotchnikoff (1986) [50] who manually performed a first derivative using Δt of ~ 0.5 ms in dark and light-adapted ffERGs using a broad (1–1000 Hz) and narrower (100–1000 Hz) band pass filters to proposed that the b-wave and part of the a-wave could be better modelled by high frequency OPs rather than the integration of the PI, PII, and PIII components suggested by Granit (1933) [51].

The FDA approach contributes to the expanding methods available with which to explore differences in the ffERG from signal analysis and machine learning [15, 16, 20, 33, 52] with synthetic datasets [53, 54]. Discrete Wavelet Transform analysis of the ERG had been applied to several studies harnessing this powerful time–frequency analysis in many fields including neurodevelopmental disorders [33], optic nerve disease [55], glaucoma [34, 52] and congenital stationary night blindness multifocal ERG ON− and OFF- pathway analysis [56]. The strength of wavelet analysis is that it provides global quantifiable measures for the ON- and OFF- pathways related to the a- and b-wave time windows, along with the early and later OPs as exemplified by Gauvin and colleagues’ seminal works [16–18]. In contrast, FDA with multivariate statistics for the mean and first derivative waveforms brings further insights into analysis of the amplitude and covariance of the waveform and its first derivative and the relative contribution of amplitude and covariance to the overall test statistic by using the transportation approach to permutation tests. Thus, clinically a significant difference may be interpreted based on the contributions of the mean or covariance differences to the test result.

The analysis of the OPs and their components have attracted the attention of multiple approaches to their description. The ISCEV ffERG standard [1] recommends a qualitative or if required a quantitative analysis based on individual peak amplitudes, the summed amplitudes of the peaks and times to peak or the root-mean-square amplitude of the OPs waveform. Fourier analysis, which determines the power spectrum, provides a quantifiable measure of the OPs waveform [57]. Bui et al. (2002) [21] used a Gabor model composed of a Gaussian envelope encompassing the OPs in the time domain and a sine wave carrier to provide a quantifiable model for the OPs. The Discrete Wavelet Transform model added to these methods by providing a simple solution to the energy contained with the early and later OPs centered on 80 and 160 Hz [16, 17]. A further advance was made by Gauthier et al. (2025) [22] who introduced the use of Hilbert Transforms that are especially suited to OPs with low signal to noise ratios and provided quantifiable measures for the peak time and its instantaneous energy. The FDA approach builds on these earlier studies to characterize the slope of the ERG—notably Lachapelle and Molotchnikoff (1986) [50] and provides the opportunity to select single or multiple OPs for a more selective analysis of mean and covariance of the waveform functions.

The continued development of approaches to modelling or analyzing the ERG waveform has resulted in different approaches that all contribute to improvements in visual electrophysiological signal analysis. Each method, time-domain, frequency-domain, Bayesian Statistics or functional data analysis provide different insights into either the morphology or energy spectrum within the ERG. Perhaps what is needed now is for many of these techniques to be engrained within the testing protocols so that clinicians can make better use of ERG analytical techniques.

In the case of this FDA analysis, which provides grid plots to represent the test statistics, and their percentage contributions provide a clear representation of the underlying differences in the waveforms based on amplitude, covariance and the first derivative (waveform dynamics). These metrics may offer an advantage over single point measures based on the peaks and troughs of the waveform. The grid plots allow for the visualization of the test results for different flash strengths, time domains, or subgroups, with the arrow pointing towards the main reason for rejecting the null hypothesis in each setup. This FDA-based approach may be particularly useful in exploratory data analysis, enabling the detection of subtle yet important data features and more focused follow-up analyses on the most important aspects. This report provides an initial overview of FDA and its potential in visual electrophysiology, treating the waveform as a function for analysis.

In this example the ASD group was included to demonstrate the application of FDA in a case–control scenario. In addition, a within group example was shown using age—with an arbitrarily cut-off of 12 years of age that was close to the mean ages of each group and represented a time point between childhood and adolescence to illustrate the potential to explore the effects of demographic features within a group. In contrast to previous studies using the same dataset with time–frequency analyses the degree of significance was not as strong between groups when the FDA approach was used [33, 37, 38] but still identified the OPs and higher flash strengths as the characteristic features that separated the ASD and Control ERG waveforms.

From a practical point of view, the advantages of this method are particularly compelling for medical practitioners. Its flexibility allows for tailored analyses that adapt to diverse clinical questions. Results are presented through intuitive graphical visualizations that are readily interpretable even by those without statistical expertise, enabling easy integration into multidisciplinary team practice and clinical uptake. Moreover, the method’s reproducibility is ensured through openly available R code, promoting transparency and enabling widespread adoption in both research and clinical practice.

## Supplementary Information

Below is the link to the electronic supplementary material.

Supplementary file1 (PDF 19228 kb)

## Data Availability

Dataset is available at: 10.25451/flinders.30968443 R-Code is available at: https://www.karlin.mff.cuni.cz/~hlavka/stat.html

## Code availability

R code can be downloaded from: https://www.karlin.mff.cuni.cz/~hlavka/stat.html
