# Supplementary material for: Technical note: a functional data analysis approach to analyze the light-adapted electroretinogram in children and adolescents
Source: Doc Ophthalmol. 2026 May 27;153(1):89–106. doi: 10.1007/s10633-026-10112-y (PMC13506519; doi:10.1007/s10633-026-10112-y)
Supplement: Supplementary file 1 [file 10633_2026_10112_MOESM1_ESM.pdf]

SUPPLEMENTARY MATERIAL
Technical Note: A Functional Data Analysis approach to analyze the light-adapted electroretinogram in children and adolescents

Zdeněk Hlávka, Paul A. Constable, Fernando Marmolejo Ramos, Lynne Loh, Dorothy A. Thompson, Jan Kalina, Marco Marozzi

April 14, 2026

Contents

1 Two-sample simultaneous comparison of mean (functions) and covariance (operators) 4

1.1 Example: Two-sample comparisons for right eye and all flash strengths 5

2 Comparisons for ERG (atime–btime or btime–72) and OP (0–60), effects of ASD×Control, Sex, and Age – within subgroups, right eye 7

2.1 OP wave by asd in 0–60 – all obs. 8

2.2 ERG wave by asd in 0–atime – all obs. 12

2.3 ERG wave by asd in atime–btime – all obs. 16

2.4 ERG wave by asd in btime–72 – all obs. 21

2.5 OP wave by sex in 0–60 – ctr subgroup 25

2.6 OP wave by sex in 0–60 – asd subgroup 29

2.7 ERG wave by sex in 0–atime – ctr subgroup 33

2.8 ERG wave by sex in 0–atime – asd subgroup 38

2.9 ERG wave by sex in atime–btime – ctr subgroup 43

2.10 ERG wave by sex in atime–btime – asd subgroup 48

2.11 ERG wave by sex in btime–72 – ctr subgroup 53

2.12 ERG wave by sex in btime–72 – asd subgroup 58

2.13 OP wave by age in 0–60 – ctr subgroup 63

2.14 OP wave by age in 0–60 – asd subgroup 67

2.15 ERG wave by age in 0–atime – ctr subgroup 71

2.16 ERG wave by age in 0–atime – asd subgroup 76

2.17 ERG wave by age in atime–btime – ctr subgroup 81

2.18 ERG wave by age in atime–btime – asd subgroup 86

2.19 ERG wave by age in btime–72 – ctr subgroup 91

2.20 ERG wave by age in btime–72 – asd subgroup 96

3 Two-sample simultaneous comparison of mean functions (the wave and its 1st derivative), right eye 101

3.1 OP wave by asd in 0–60 – all obs. 102

3.2 ERG wave by asd in 0–atime – all obs. 103

3.3 ERG wave by asd in atime–btime – all obs. 104

3.4 ERG wave by asd in btime–72 – all obs. 105

3.5 OP wave by sex in 0–60 – ctr subgroup 106

3.6 OP wave by sex in 0–60 – asd subgroup 107

3.7 ERG wave by sex in 0–atime – ctr subgroup 108

3.8 ERG wave by sex in 0–atime – asd subgroup 109

3.9 ERG wave by sex in atime–btime – ctr subgroup 110

3.10 ERG wave by sex in atime–btime – asd subgroup 111

3.11 ERG wave by sex in btime–72 – ctr subgroup 112

3.12 ERG wave by sex in btime–72 – asd subgroup 113

3.13 OP wave by age in 0–60 – ctr subgroup 114

3.14 OP wave by age in 0–60 – asd subgroup 115

3.15 ERG wave by age in 0–atime – ctr subgroup 116

3.16 ERG wave by age in 0–atime – asd subgroup 117

3.17 ERG wave by age in atime–btime – ctr subgroup 118

3.18 ERG wave by age in atime–btime – asd subgroup 119

3.19 ERG wave by age in btime–72 – ctr subgroup 120

3.20 ERG wave by age in btime–72 – asd subgroup 121

Chapter 1

Two-sample simultaneous comparison of mean (functions) and covariance (operators)

We start by testing equality of distributions of the observed two groups of functions for right eye and flash strength 70. The (bivariate) test statistic combines a test statistic for equality of mean functions and a test statistic for equality of covariance operators.

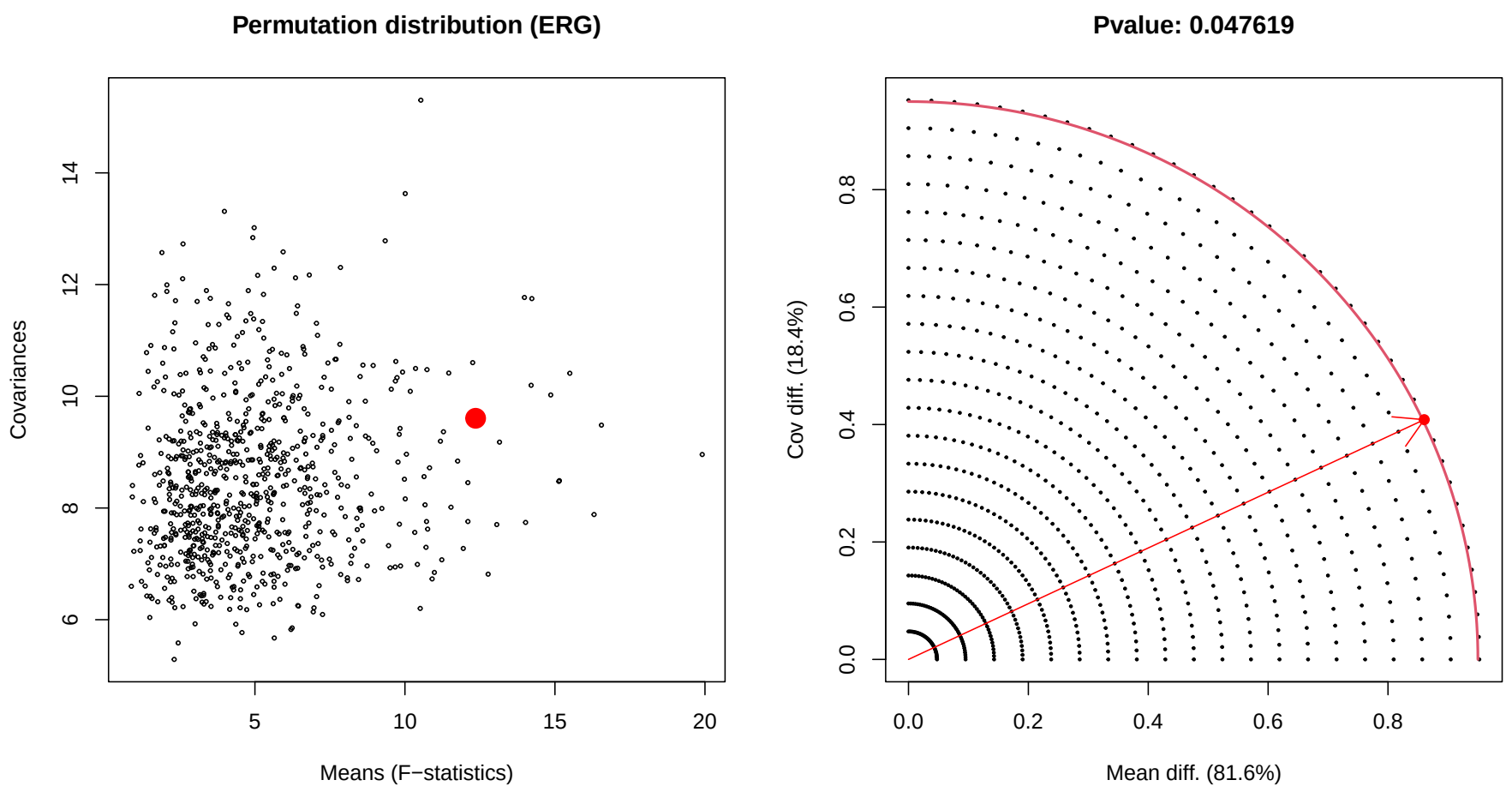

The observed test statistic (red point, say $F_T = (F_1, F_2)^\top$) is compared to the null distribution obtained by permutations — in our approach, this is

achieved by using the so-called center-outward distribution function, i.e., by transforming the permutation distribution (on the left) to a suitable spherical grid (on the right). The arrow denotes the location corresponding to the observed test statistic, say $S_T = (S_1, S_2)^\top$. With this notation, we can calculate the p-value as $1 - \|S_T\|$.

In this case, the p-value is 0.045 and we reject equality of the two functional distributions.

Notice also that $\|S_T\|^2 = S_1^2 + S_2^2$, where S_1 corresponds to the first test (equality of means) and S_2 corresponds to the second test (equality of covariance operators). Therefore, we can interpret $S_1^2/\|S_T\|^2$ and $S_2^2/\|S_T\|^2$ as the relative contributions of the two marginal tests to the test decision: in this example, the difference in means is much more important because the relative contribution of the observed mean differences is 87.4%.

Altogether, we can conclude that we reject the null hypothesis (p-value 0.045) mainly because of differences in mean functions (relative contribution of the "means test" is 87.4%).

1.1 Example: Two-sample comparisons for right eye and all flash strengths

Graphical representation of the ten tests for right eye and 10 flash strengths:

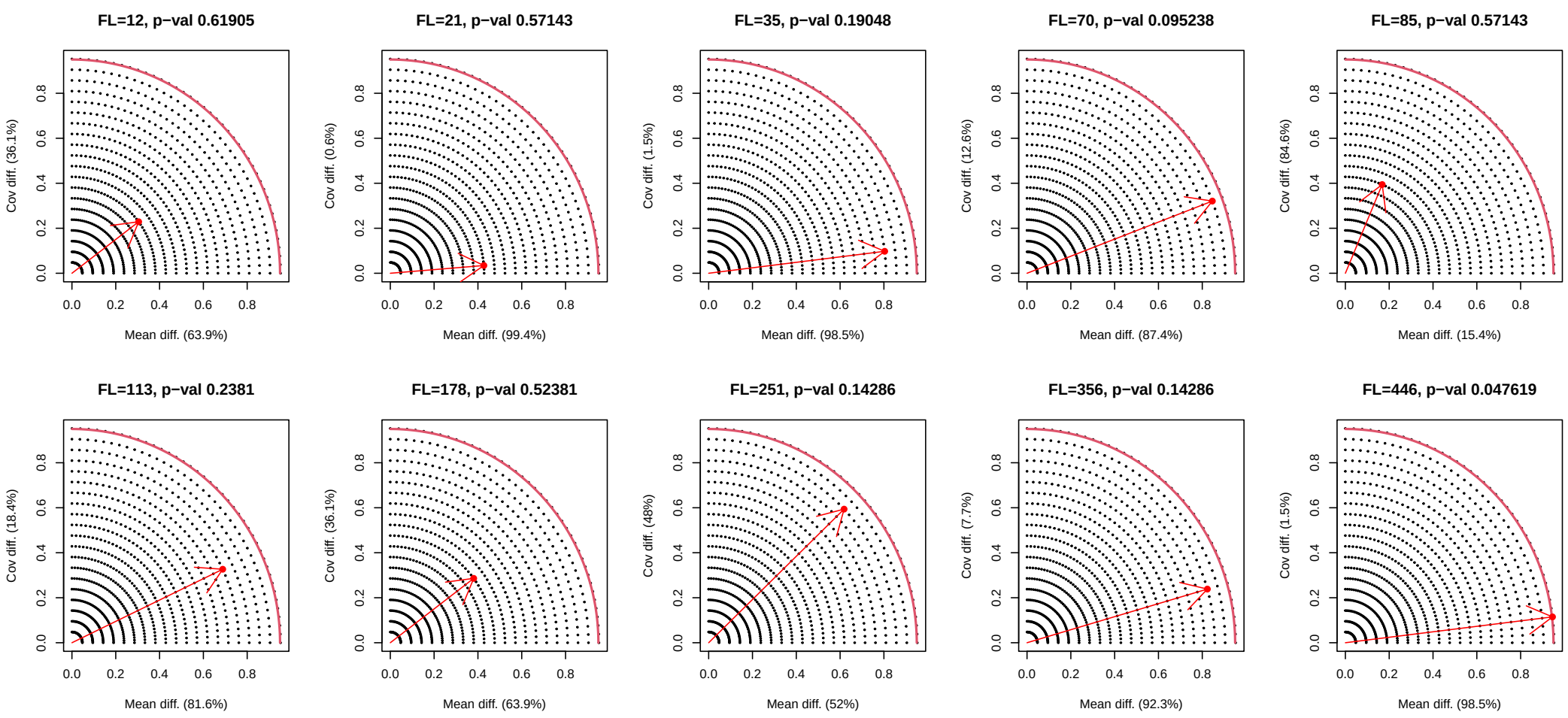

P-values and relative contributions of the two tests are given in the plots, the test is significant (with level 0.05) if the arrow crosses the red circle (with radius $1 - 0.05$).

Alternatively, the test results can be summarized also in a table, see next chapters.

Chapter 2

Comparisons for ERG (atime–btime or btime–72) and OP (0–60), effects of ASD×Control, Sex, and Age – within subgroups, right eye

Data set: ERGandOP.xlsx, spline approximations of the waves.

The spline approximation of OP wave is a bit more coarse due to numerical problems with finer grid (when comparing covariance operators).

Green arrows represent test results for observed waveforms, red arrows are for first derivatives.

2.1 OP wave by asd in 0–60 – all obs.

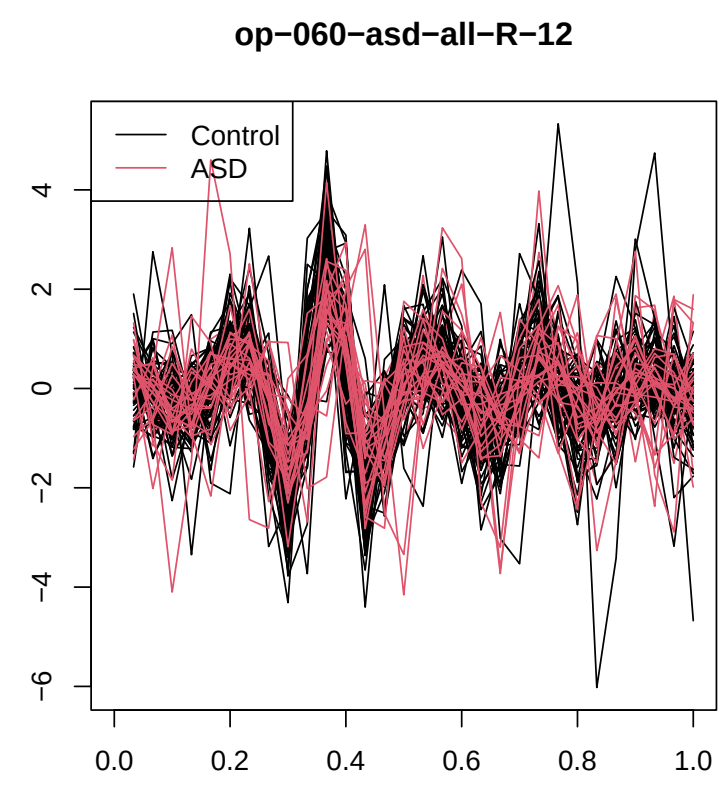

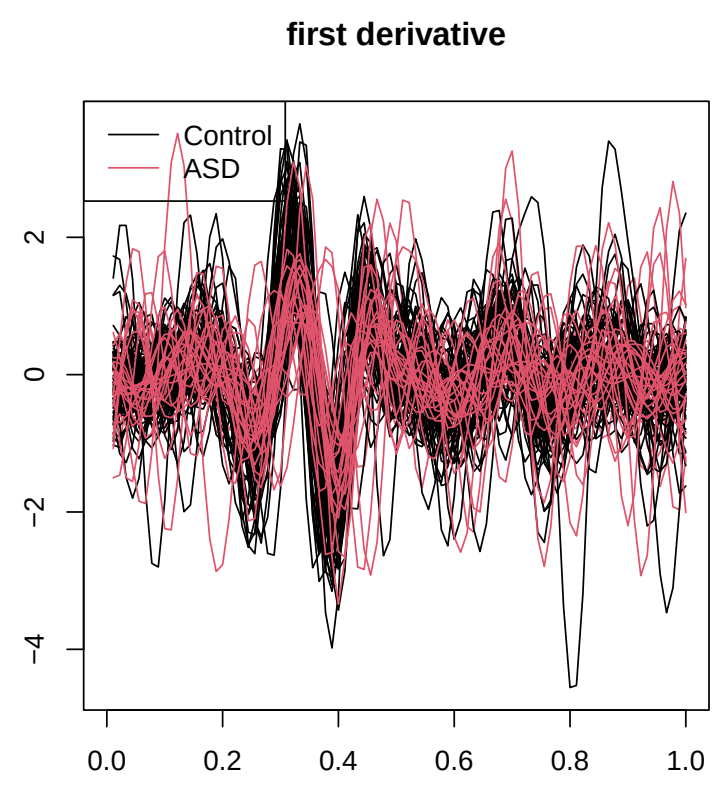

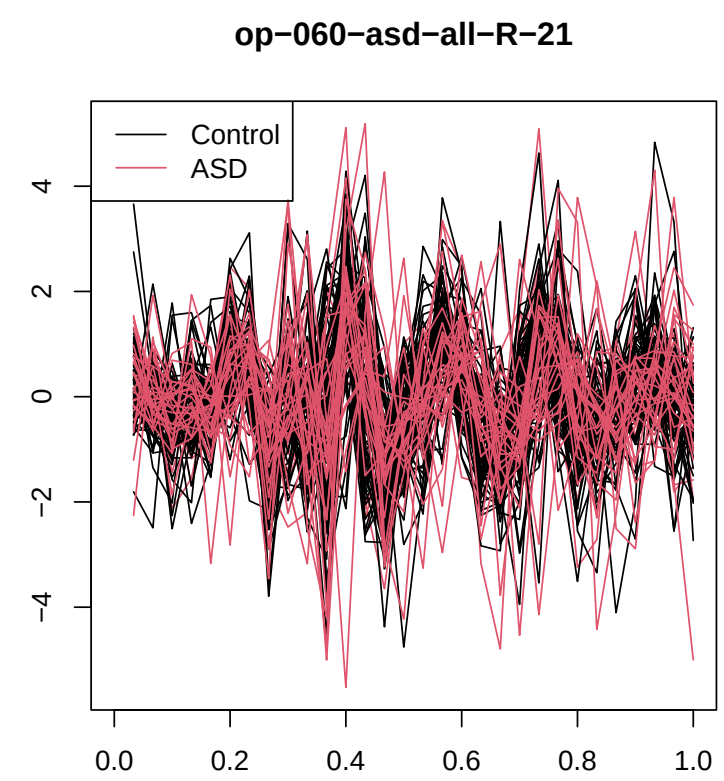

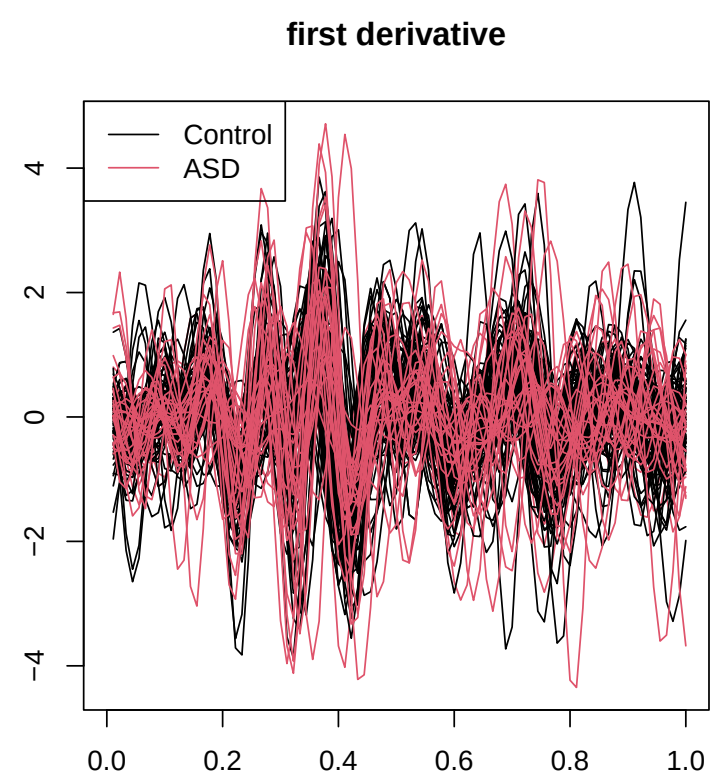

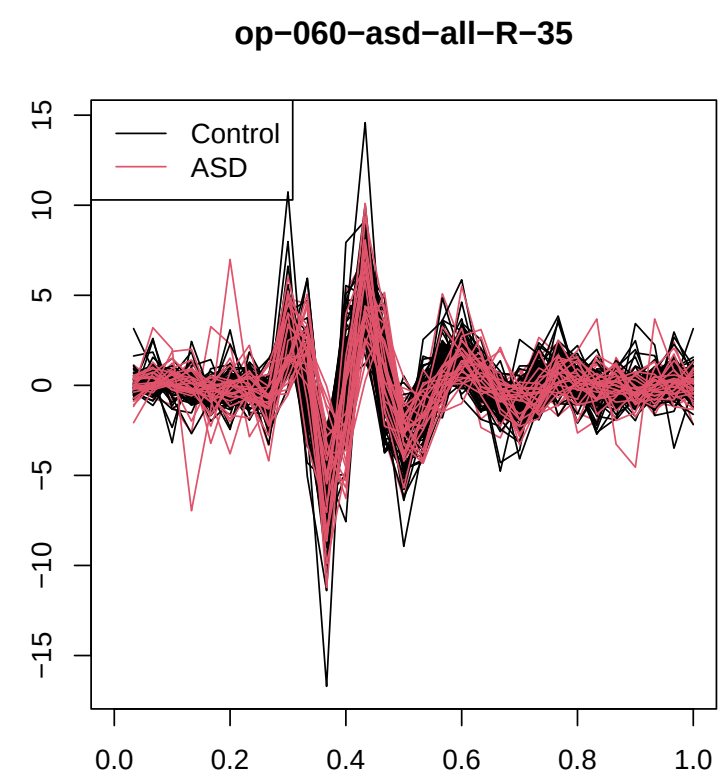

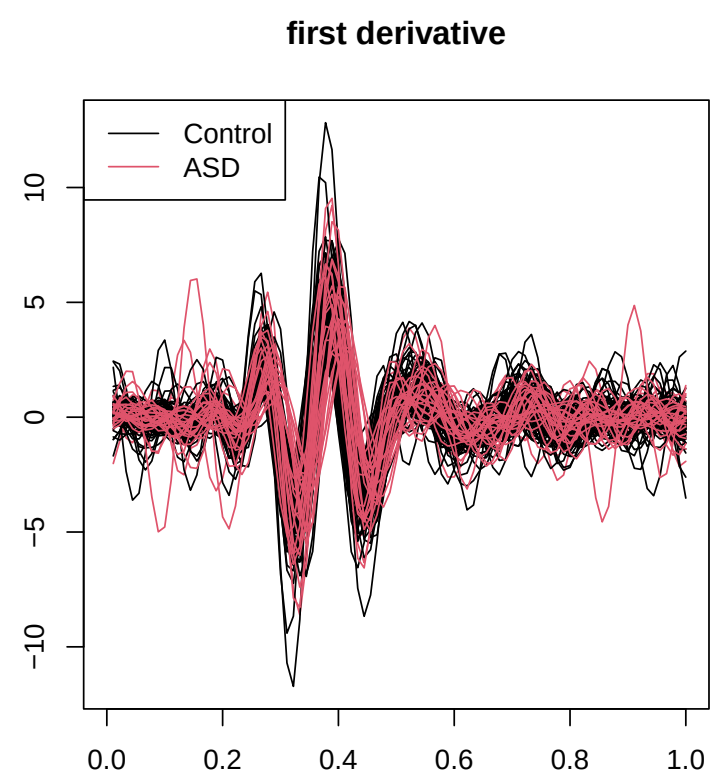

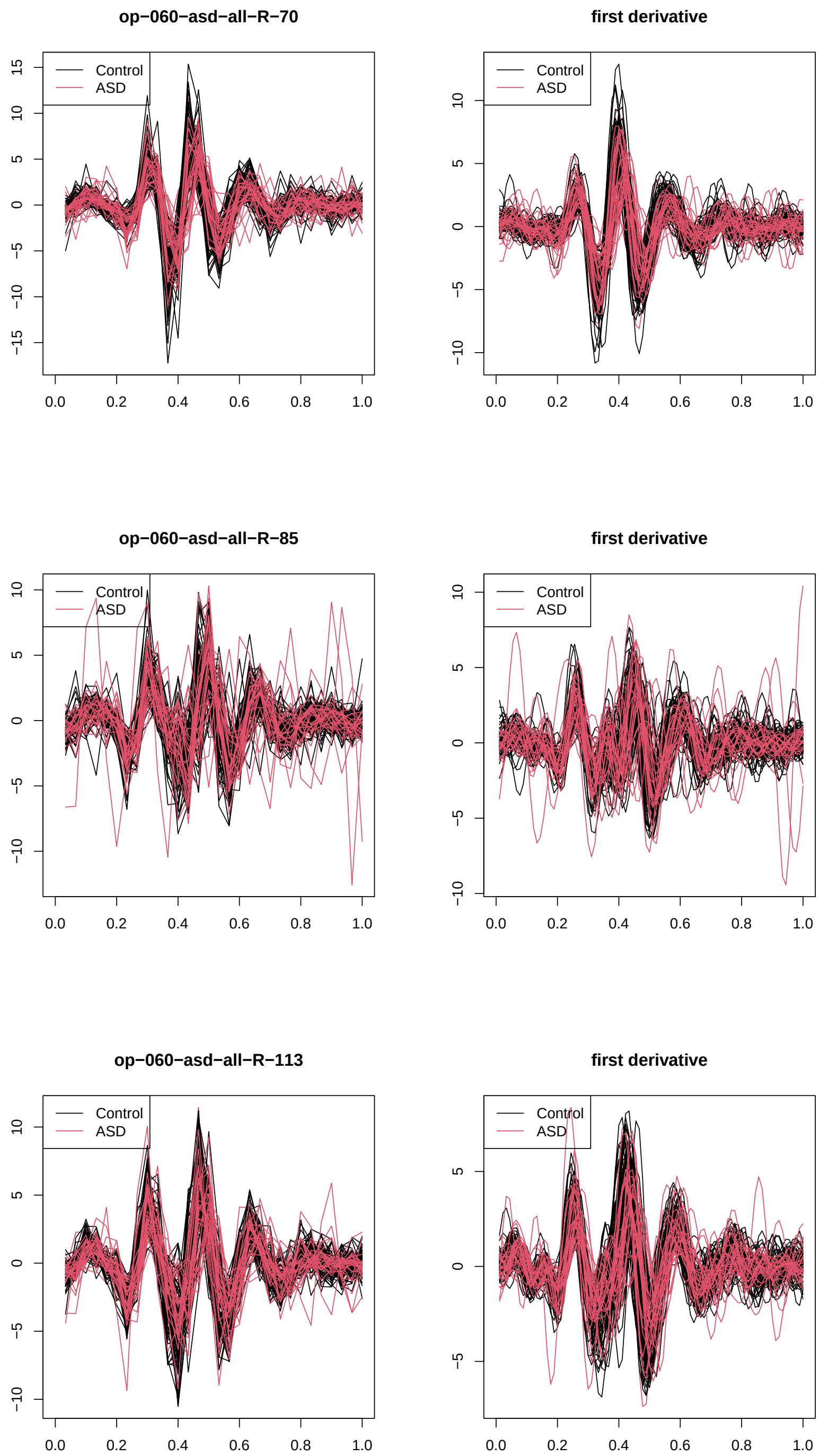
op−060−asd−all−R−70
Control
ASD
first derivative
Control
ASD
op−060−asd−all−R−85
Control
ASD
first derivative
Control
ASD
op−060−asd−all−R−113
Control
ASD
first derivative
Control
ASD

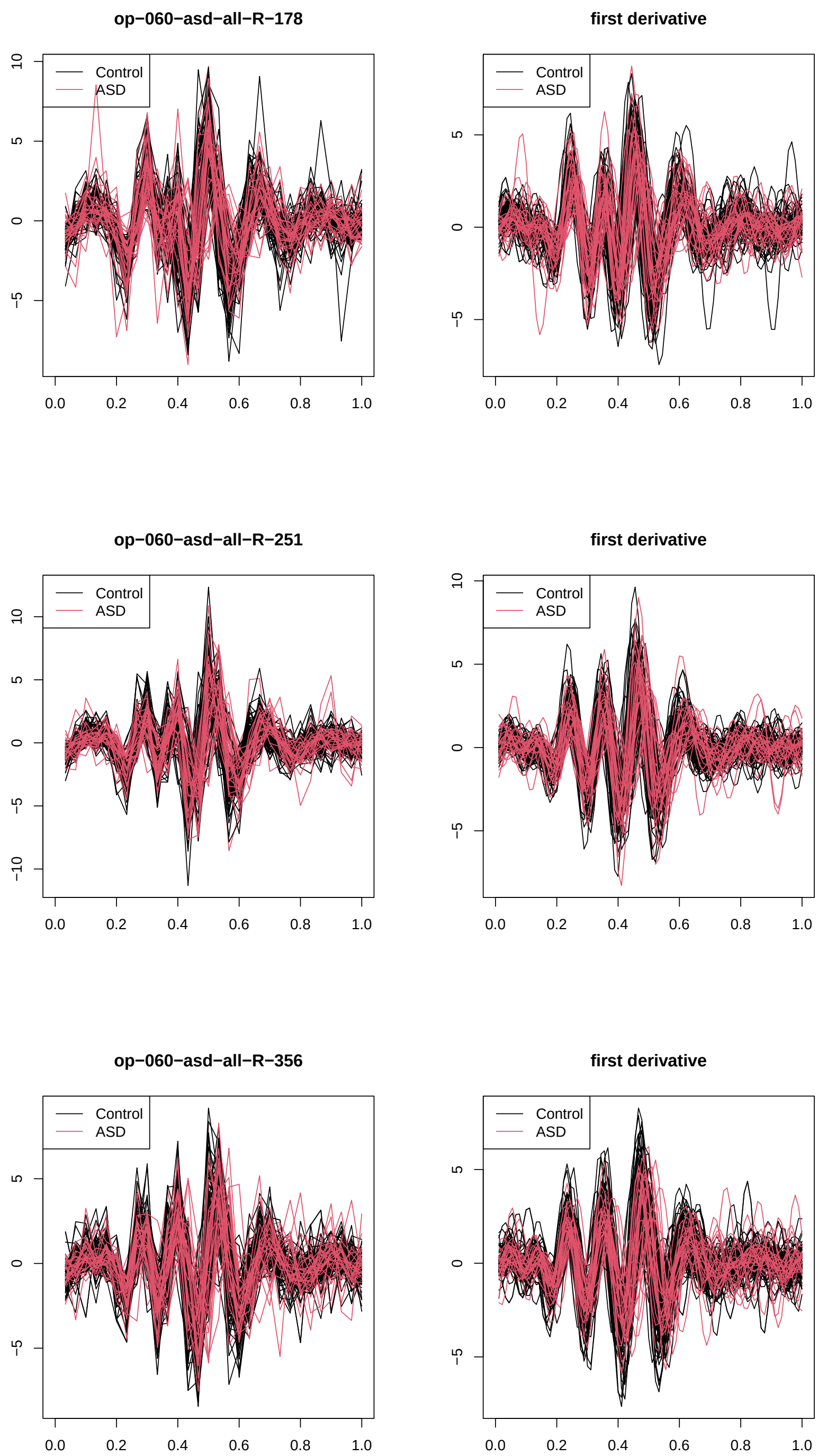
op−060−asd−all−R−178
Control
ASD
first derivative
Control
ASD
op−060−asd−all−R−251
Control
ASD
first derivative
Control
ASD
op−060−asd−all−R−356
Control
ASD
first derivative
Control
ASD
0.0 0.2 0.4 0.6 0.8 1.0

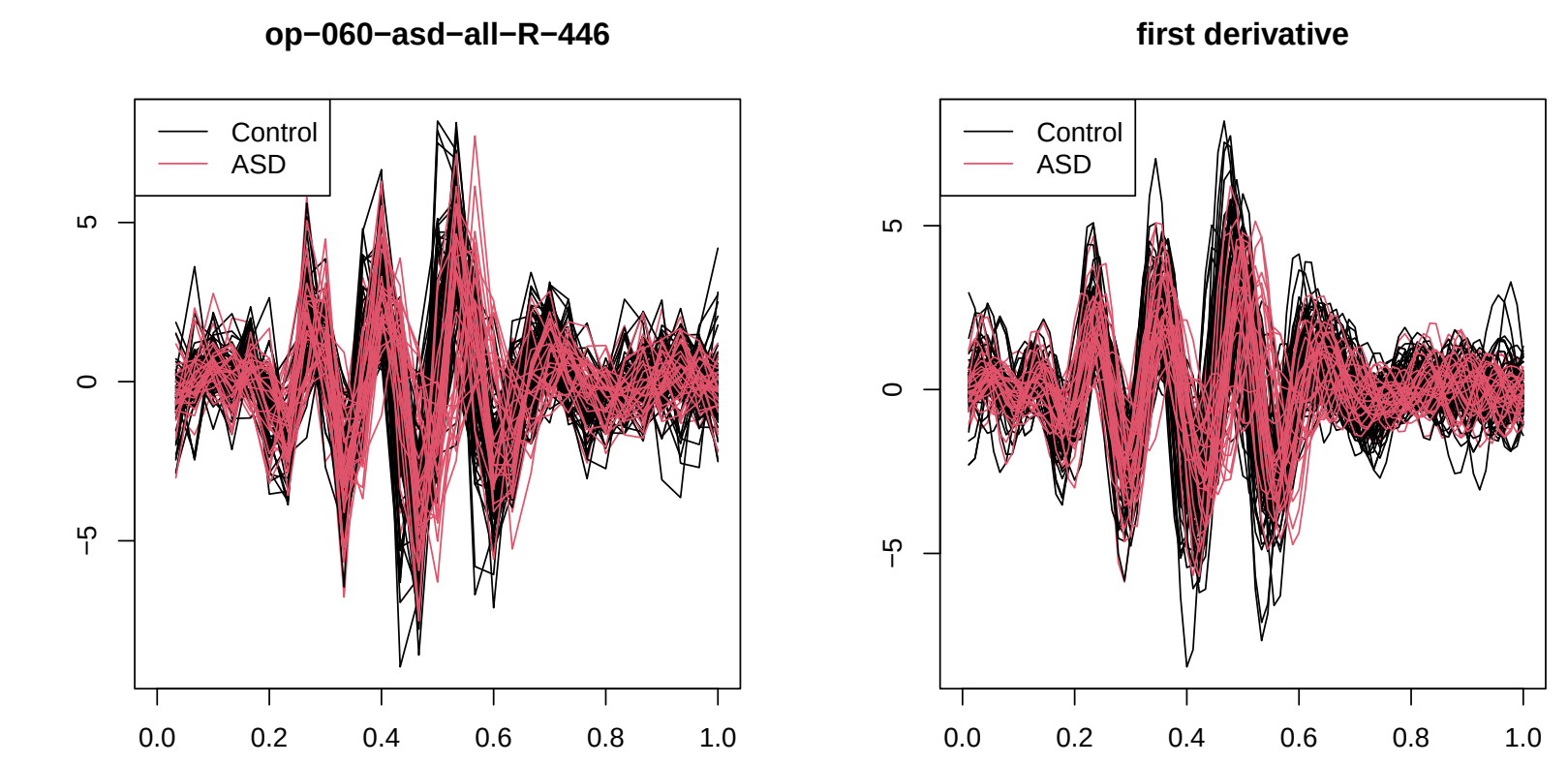

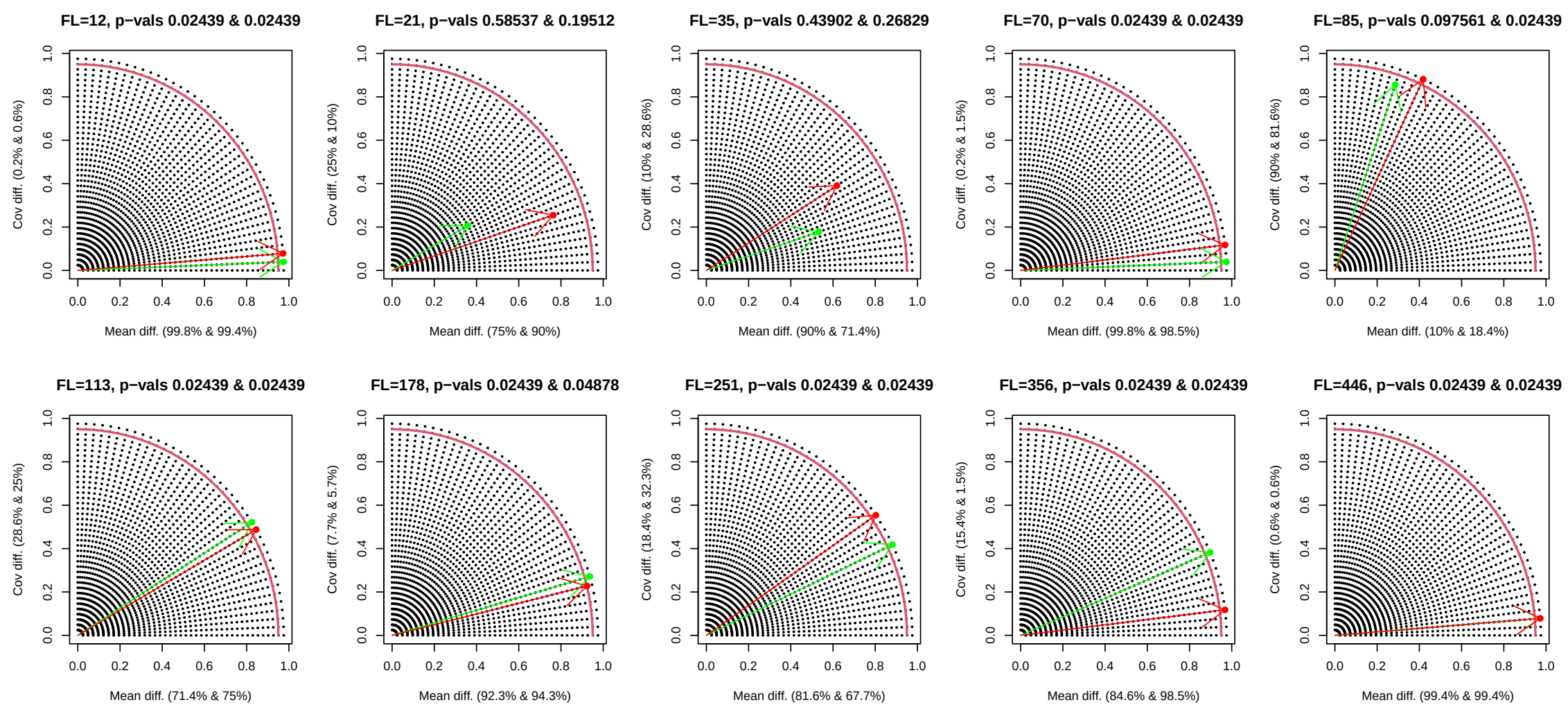

Table for OP wave by asd in 0–60 – all obs.

	pval-op-060	mean perc	cov perc	pval-derivative	mean perc	cov perc
12	0.0244	99.8	0.2	0.0244	99.4	0.6
21	0.5854			0.1951		
35	0.4390			0.2683		
70	0.0244	99.8	0.2	0.0244	98.5	1.5
85	0.0976			0.0244	18.4	81.6
113	0.0244	71.4	28.6	0.0244	75	25
178	0.0244	92.3	7.7	0.0488	94.3	5.7
251	0.0244	81.6	18.4	0.0244	67.7	32.3
356	0.0244	84.6	15.4	0.0244	98.5	1.5
446	0.0244	99.4	0.6	0.0244	99.4	0.6

2.2 ERG wave by asd in 0–atime – all obs.

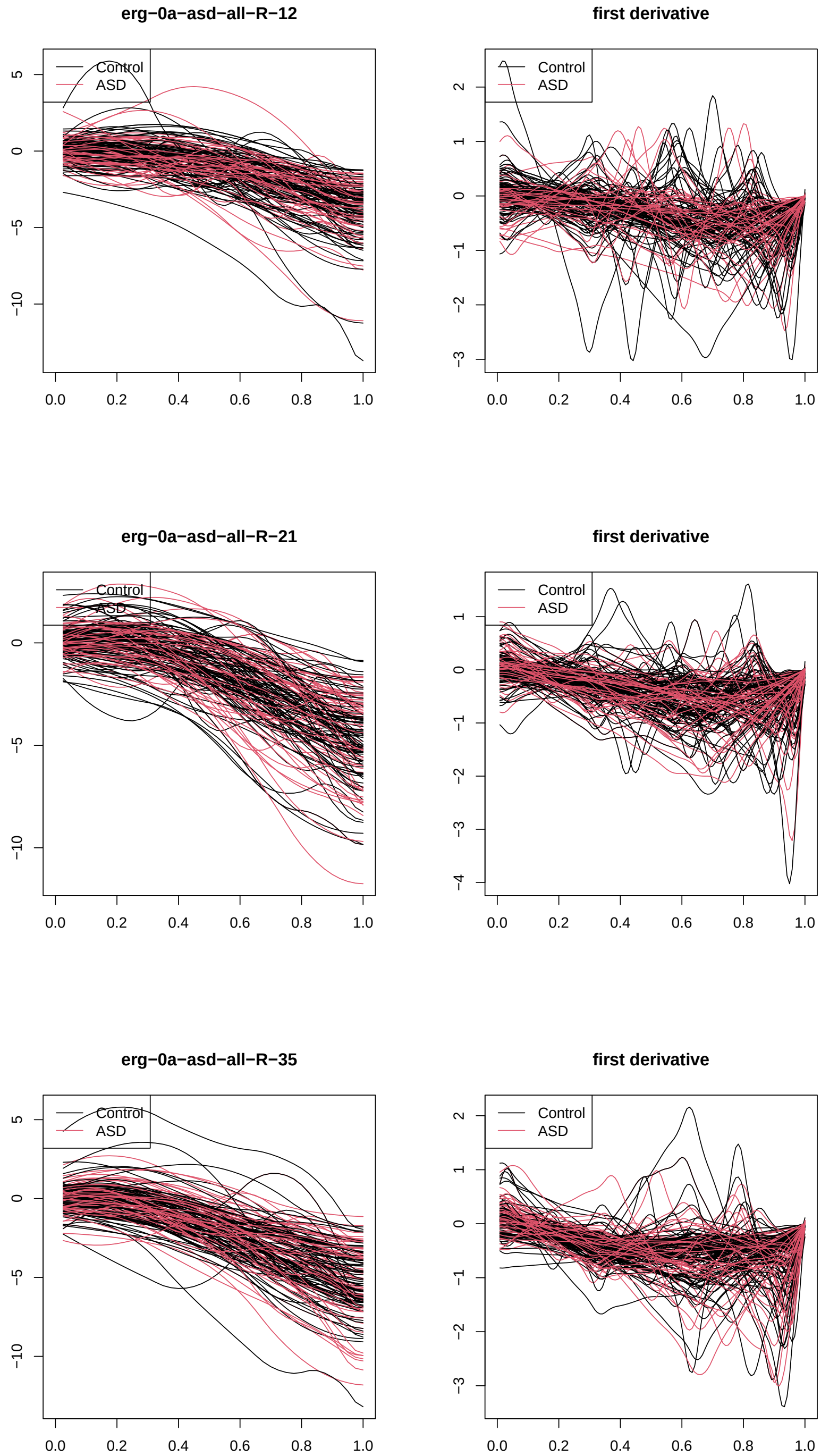

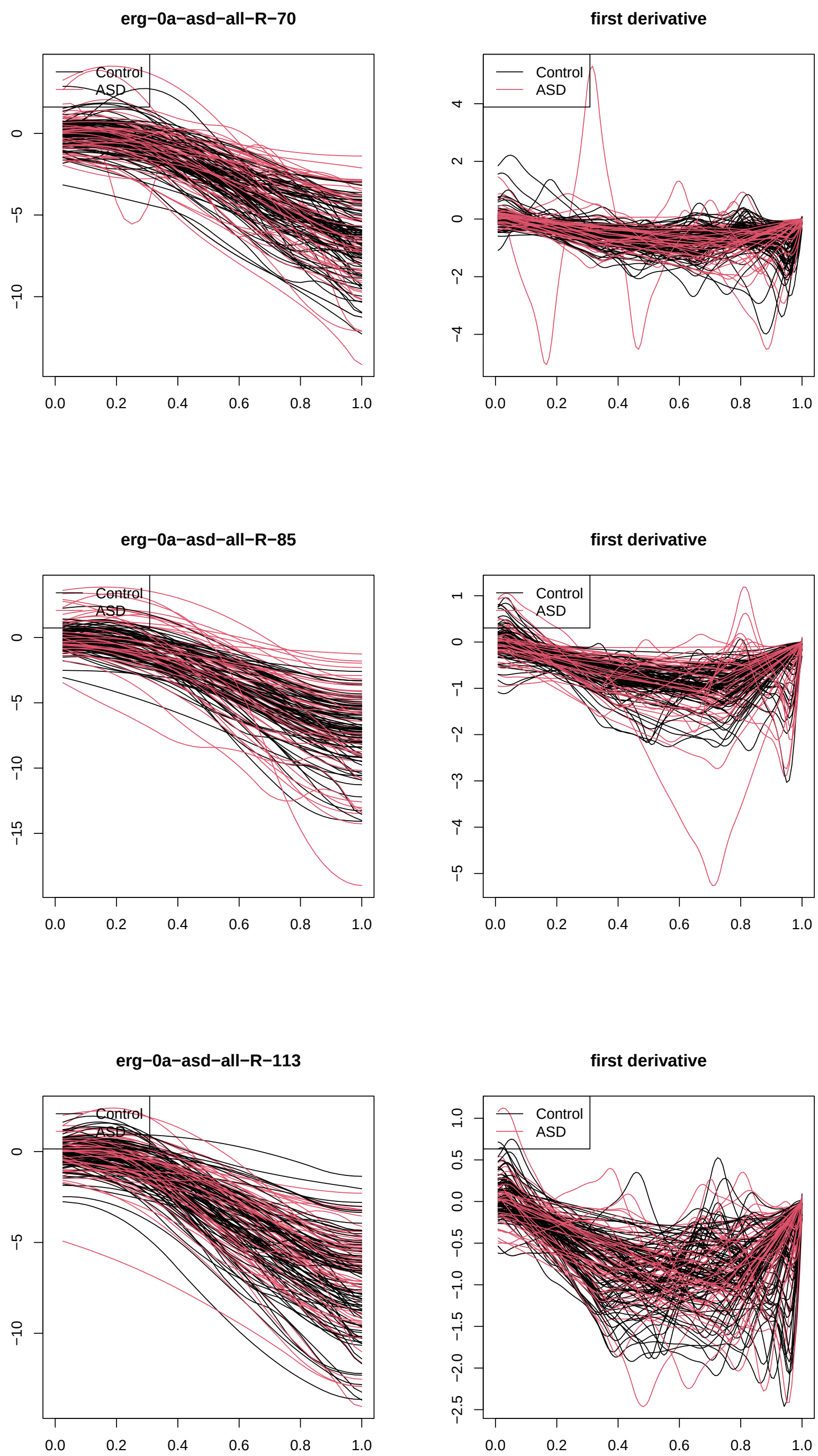

erg−0a−asd−all−R−70
first derivative
erg−0a−asd−all−R−85
first derivative
erg−0a−asd−all−R−113
first derivative
Control
ASD
0.0
0.2
0.4
0.6
0.8
1.0

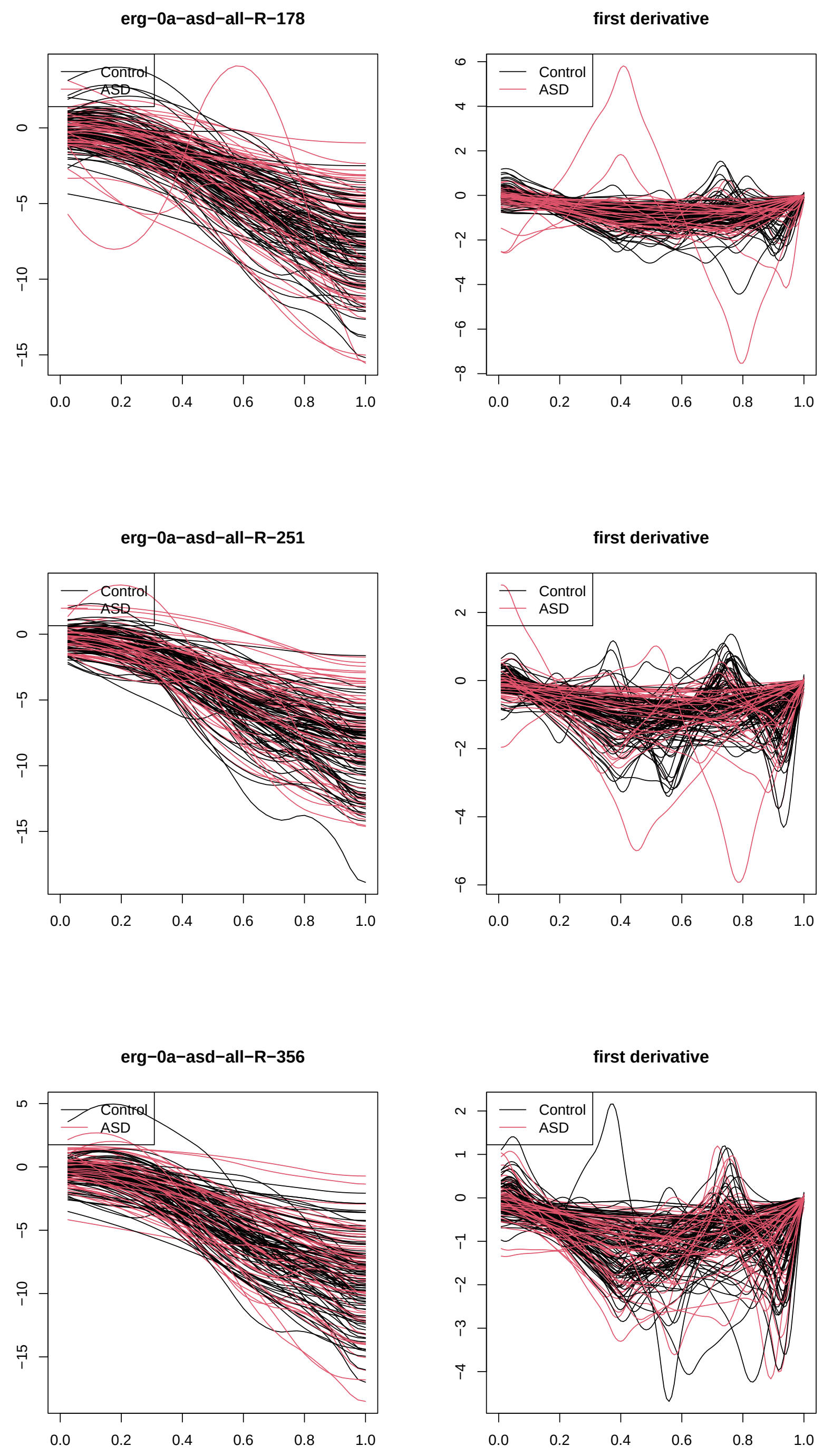
erg−0a−asd−all−R−178
first derivative
Control
ASD
erg−0a−asd−all−R−251
first derivative
Control
ASD
erg−0a−asd−all−R−356
first derivative
Control
ASD

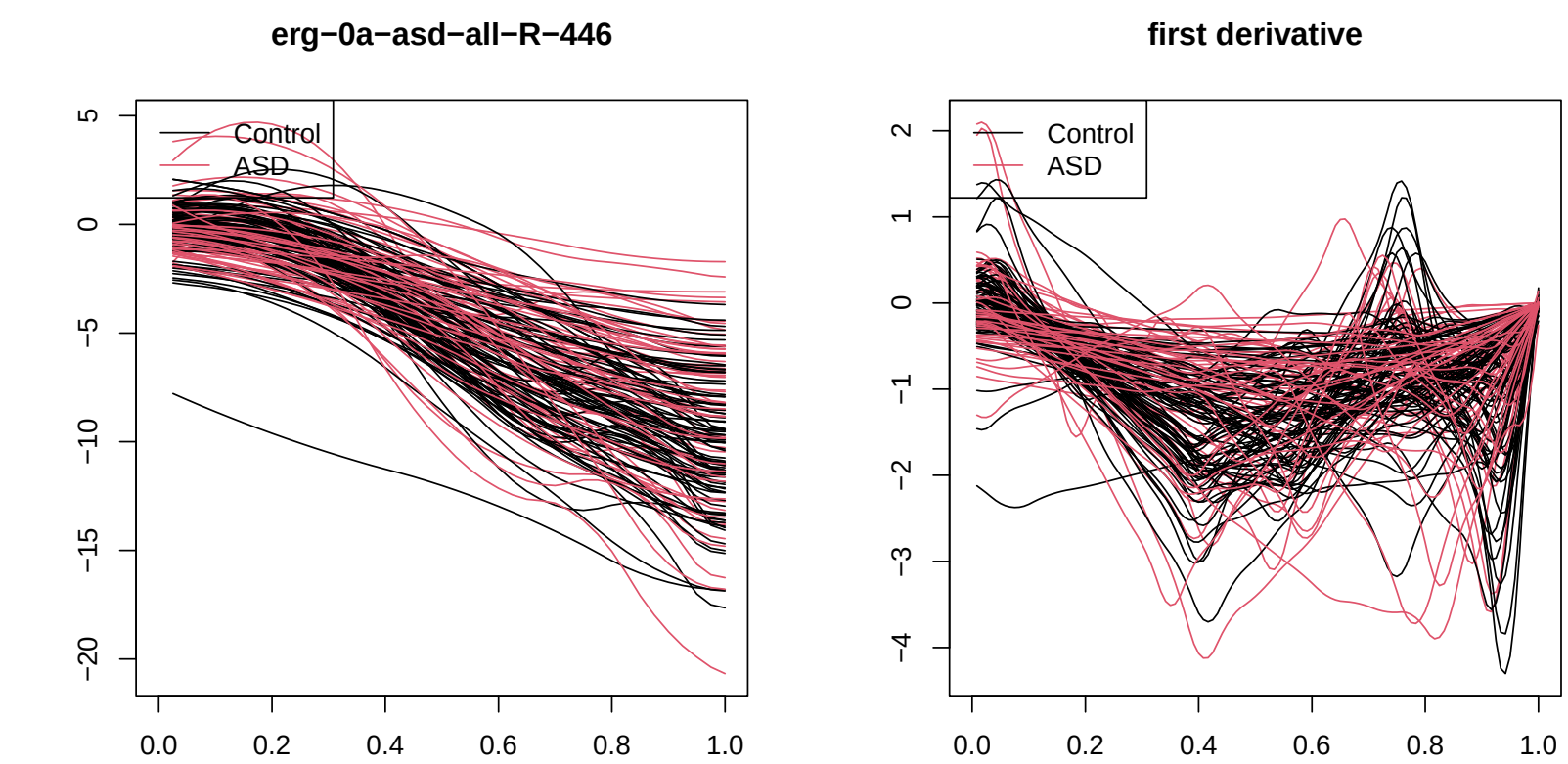

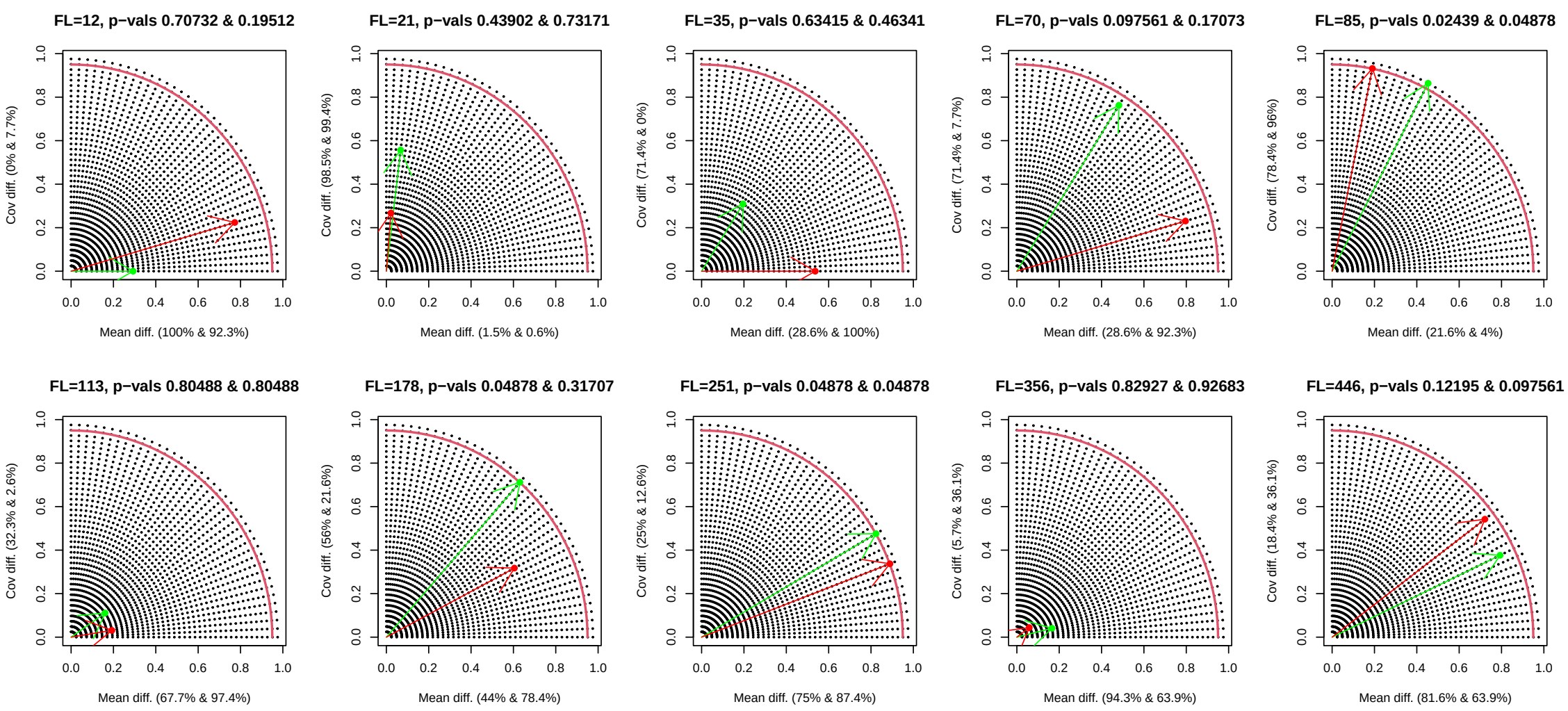

Table for ERG wave by asd in 0–atime – all obs.

	pval-erg-0a	mean perc	cov perc	pval-derivative	mean perc	cov perc
12	0.7073			0.1951		
21	0.4390			0.7317		
35	0.6341			0.4634		
70	0.0976			0.1707		
85	0.0244	21.6	78.4	0.0488	4	96
113	0.8049			0.8049		
178	0.0488	44	56	0.3171		
251	0.0488	75	25	0.0488	87.4	12.6
356	0.8293			0.9268		
446	0.1220			0.0976		

2.3 ERG wave by asd in atime–btime – all obs.

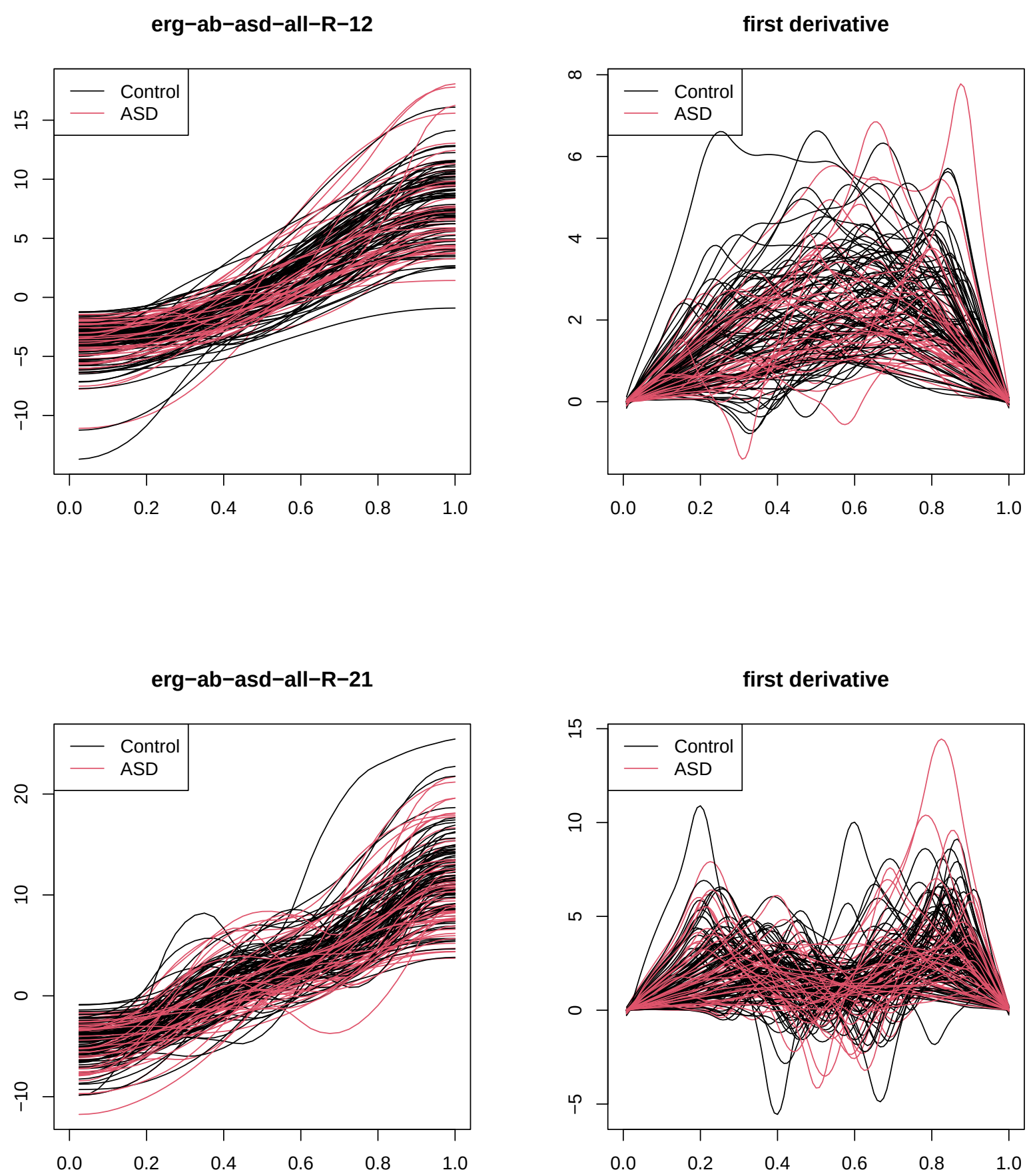

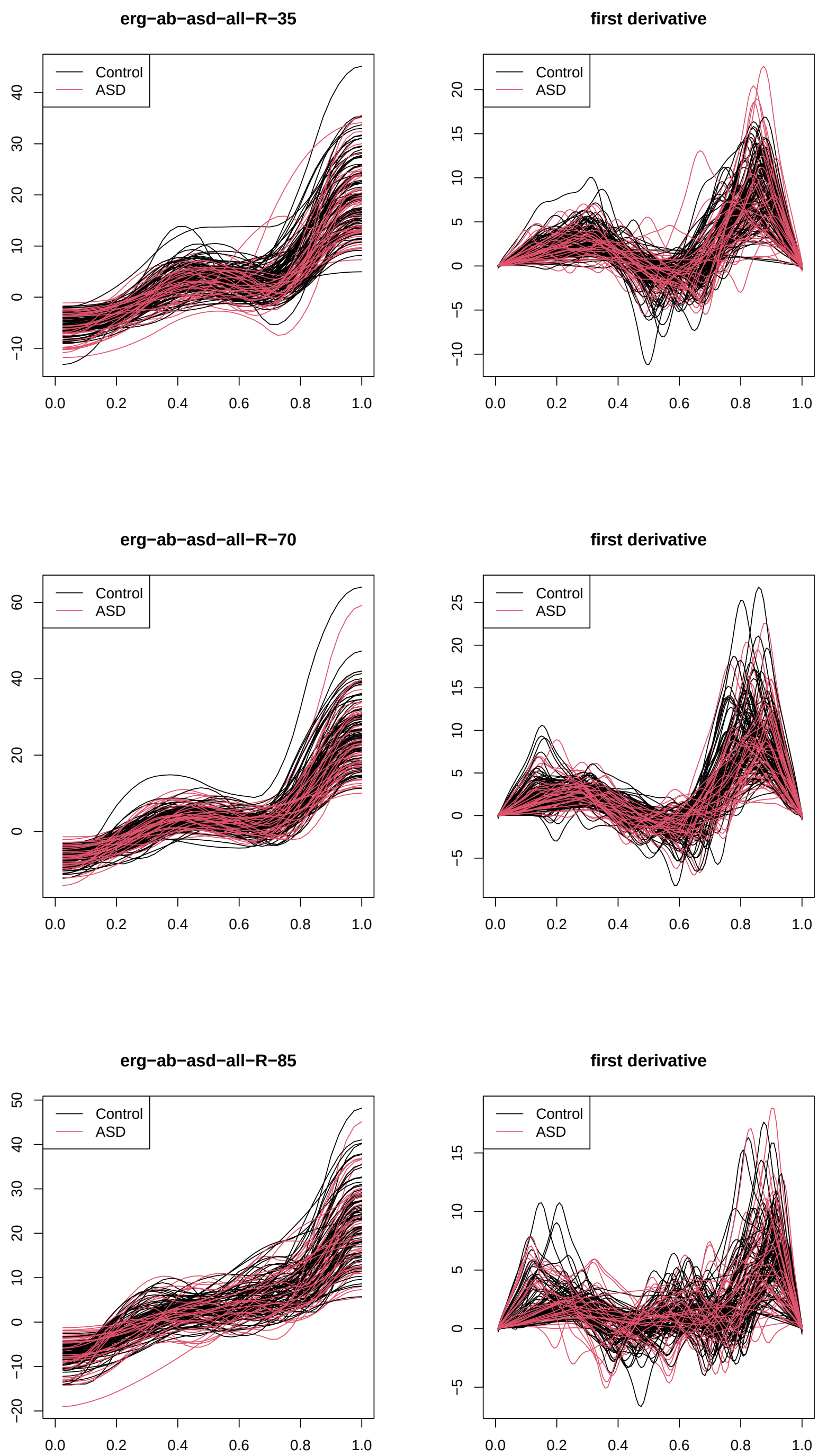
erg−ab−asd−all−R−35
first derivative
erg−ab−asd−all−R−70
first derivative
erg−ab−asd−all−R−85
first derivative
Control
ASD

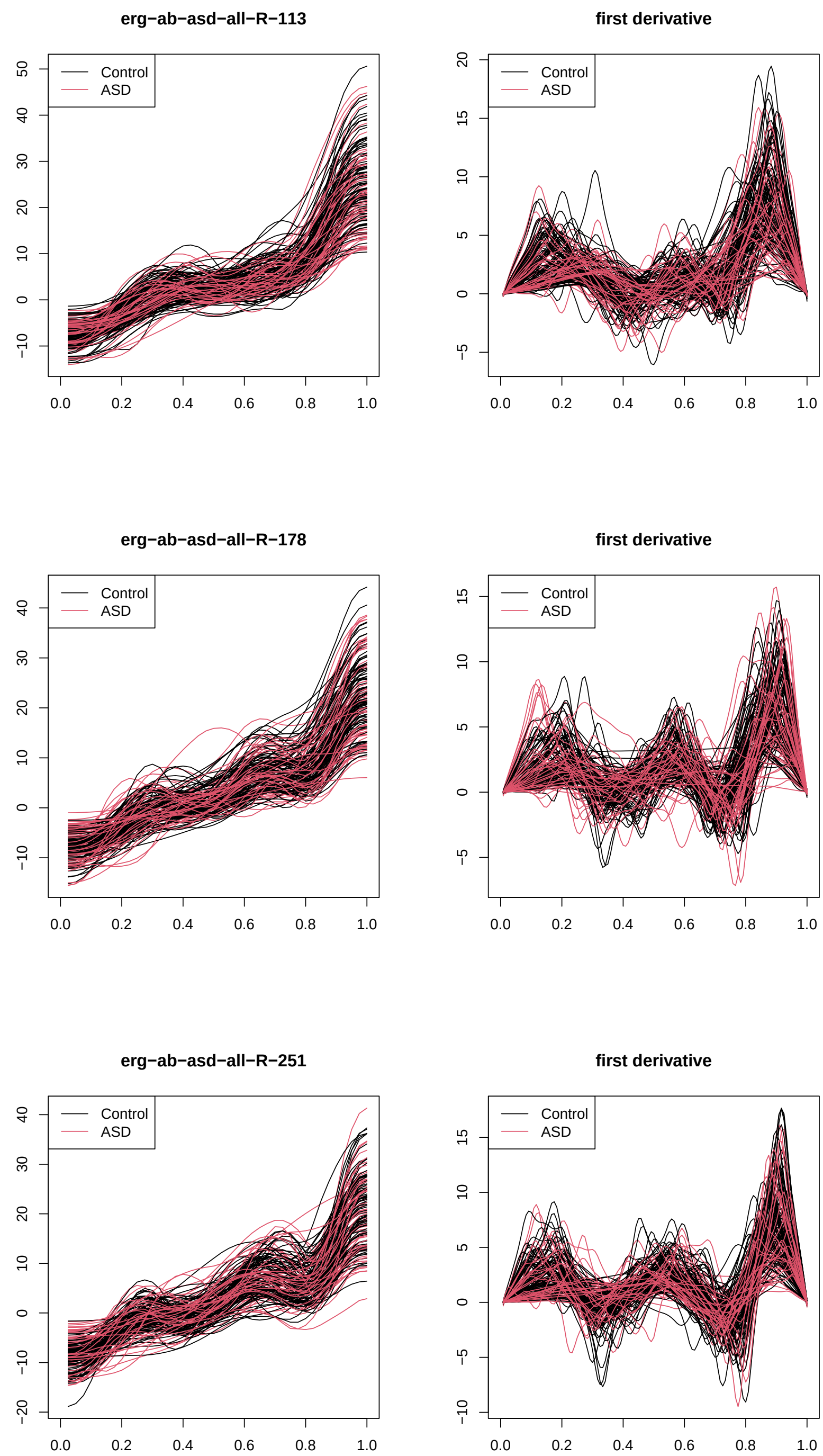
erg−ab−asd−all−R−113
first derivative
Control
ASD
erg−ab−asd−all−R−178
first derivative
Control
ASD
erg−ab−asd−all−R−251
first derivative
Control
ASD

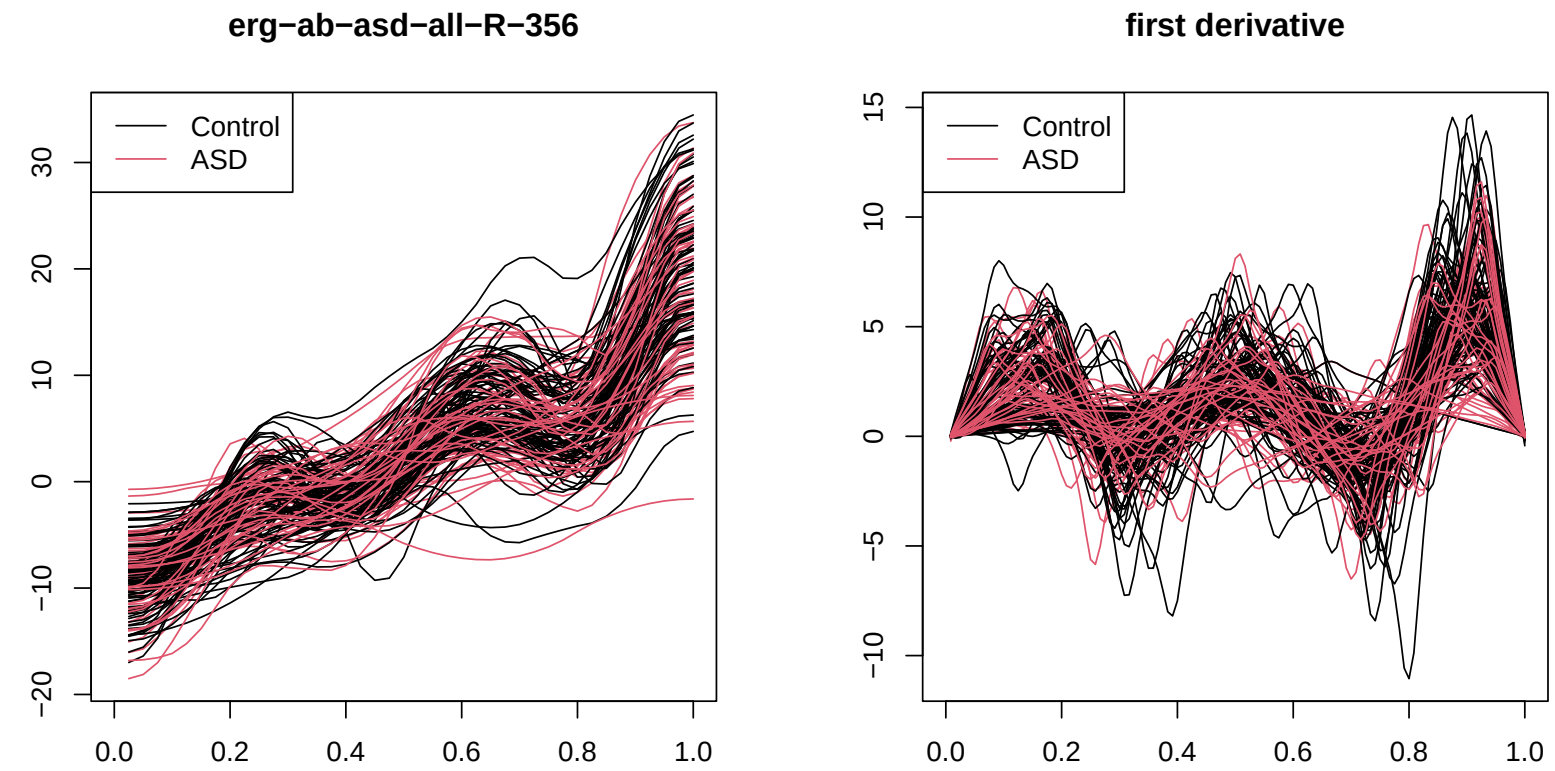
erg−ab−asd−all−R−356
first derivative
Control
ASD

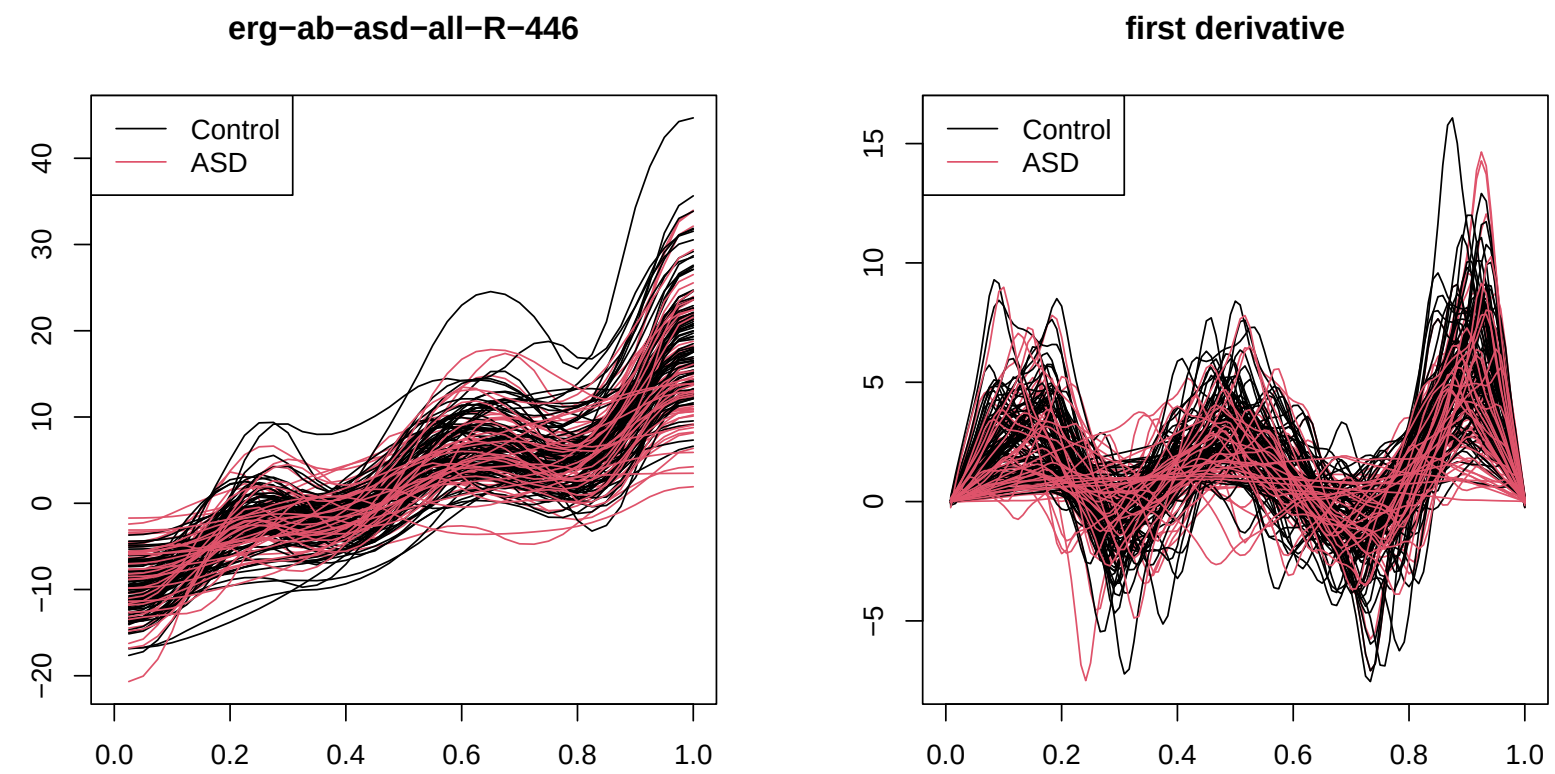
erg−ab−asd−all−R−446
first derivative
Control
ASD

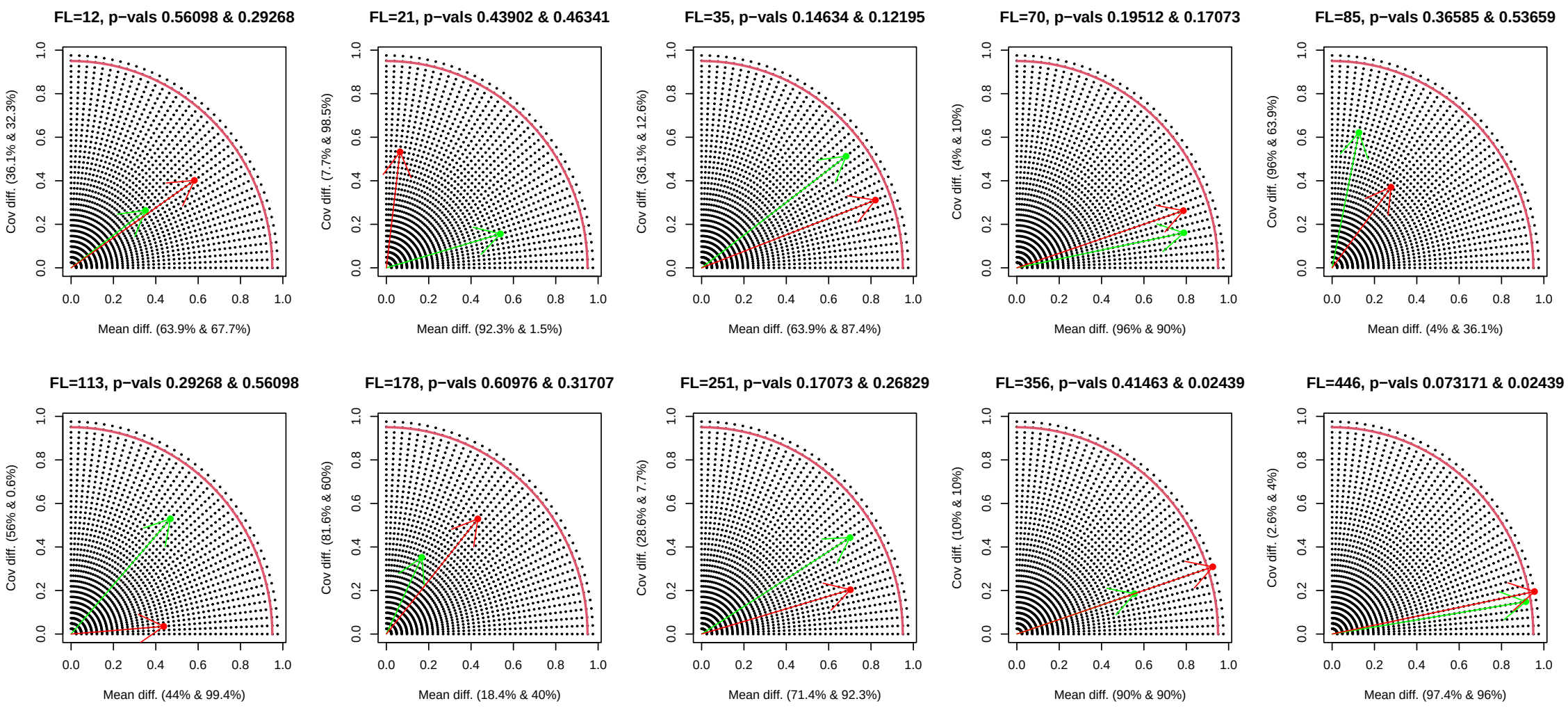
FL=12, p−vals 0.56098 & 0.29268
Mean diff. (63.9% & 67.7%)
FL=21, p−vals 0.43902 & 0.46341
Mean diff. (92.3% & 1.5%)
FL=35, p−vals 0.14634 & 0.12195
Mean diff. (63.9% & 87.4%)
FL=70, p−vals 0.19512 & 0.17073
Mean diff. (96% & 90%)
FL=85, p−vals 0.36585 & 0.53659
Mean diff. (4% & 36.1%)
FL=113, p−vals 0.29268 & 0.56098
Mean diff. (44% & 99.4%)
FL=178, p−vals 0.60976 & 0.31707
Mean diff. (18.4% & 40%)
FL=251, p−vals 0.17073 & 0.26829
Mean diff. (71.4% & 92.3%)
FL=356, p−vals 0.41463 & 0.02439
Mean diff. (90% & 90%)
FL=446, p−vals 0.073171 & 0.02439
Mean diff. (97.4% & 96%)

Table for ERG wave by asd in atime–btime – all obs.

	pval-erg-ab	mean perc	cov perc	pval-derivative	mean perc	cov perc
12	0.5610			0.2927		
21	0.4390			0.4634		
35	0.1463			0.1220		
70	0.1951			0.1707		
85	0.3659			0.5366		
113	0.2927			0.5610		
178	0.6098			0.3171		
251	0.1707			0.2683		
356	0.4146			0.0244	90	10
446	0.0732			0.0244	96	4

2.4 ERG wave by asd in btime–72 – all obs.

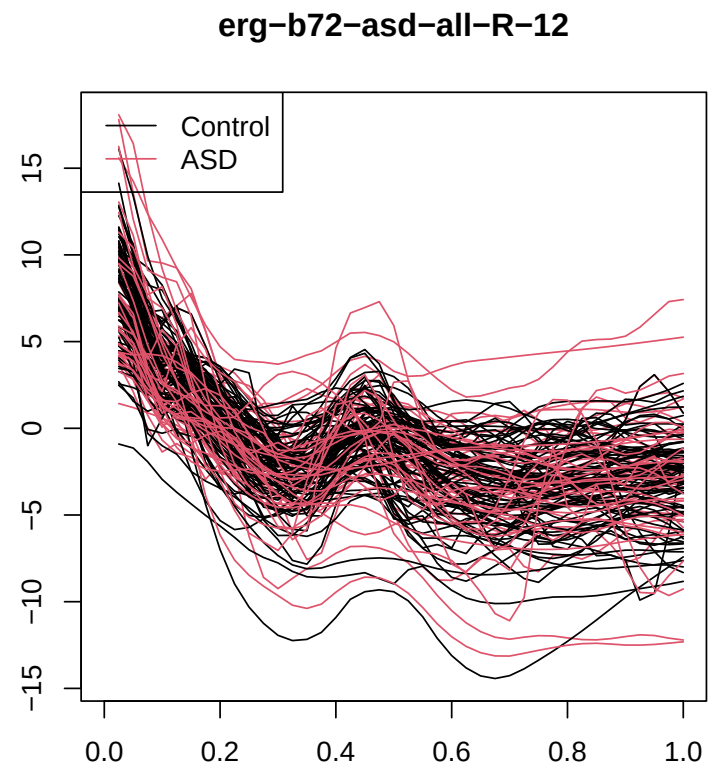

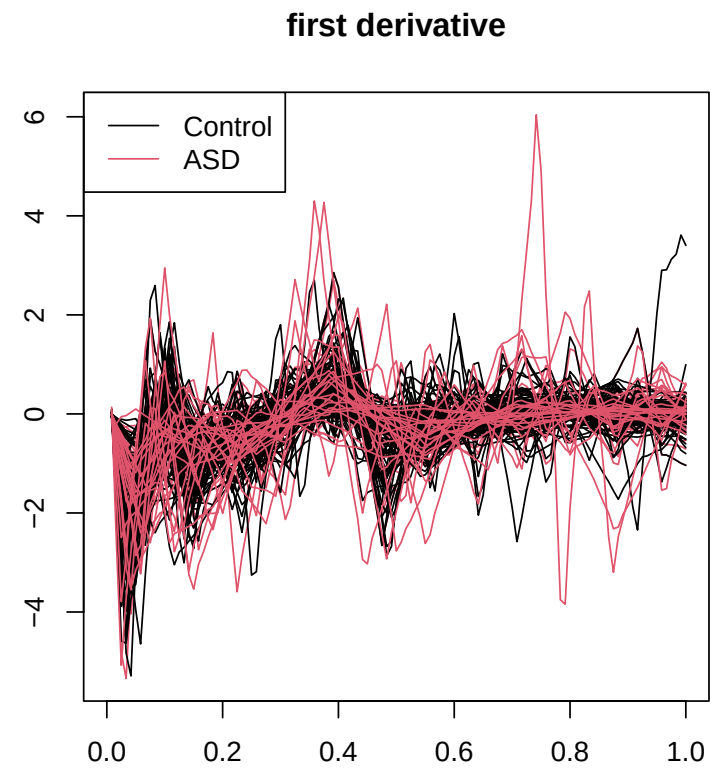

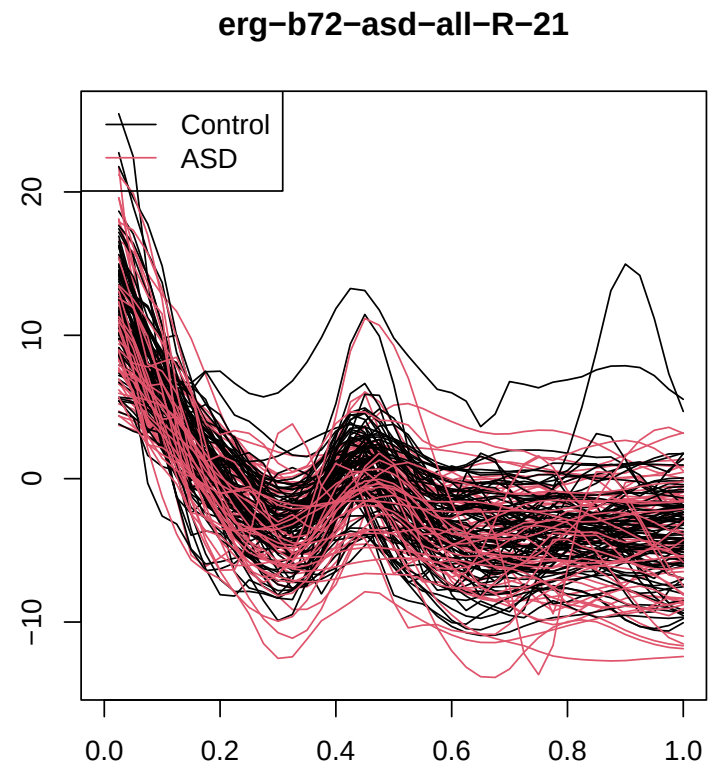

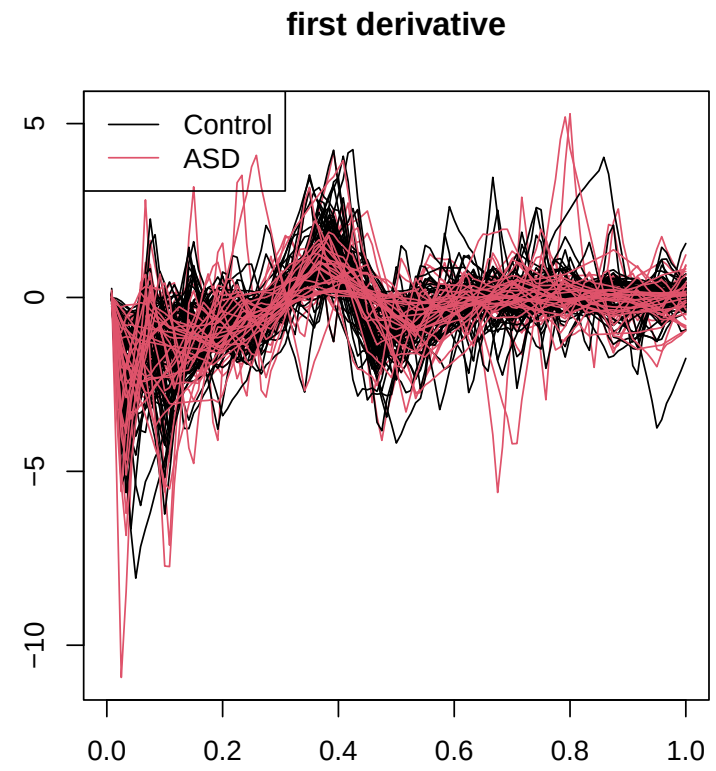

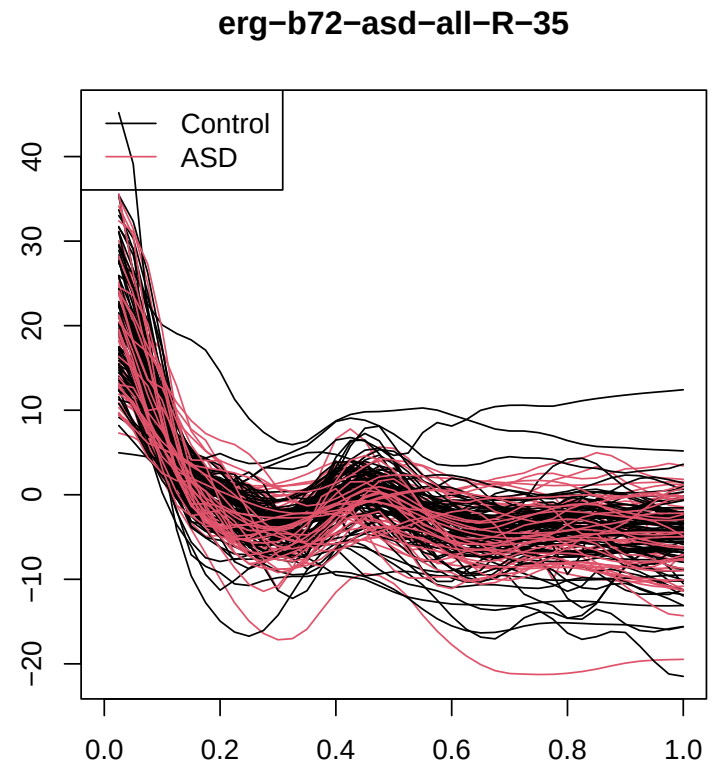

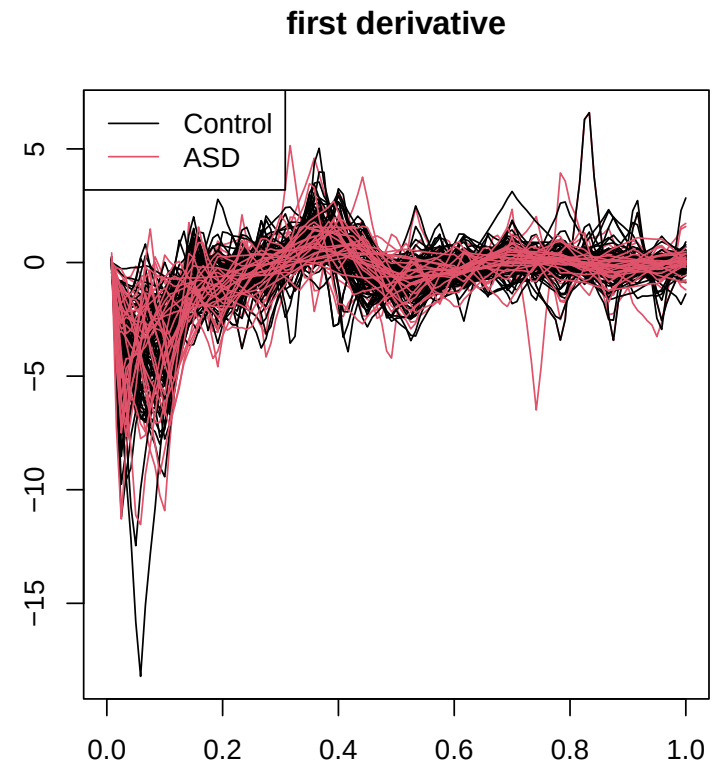

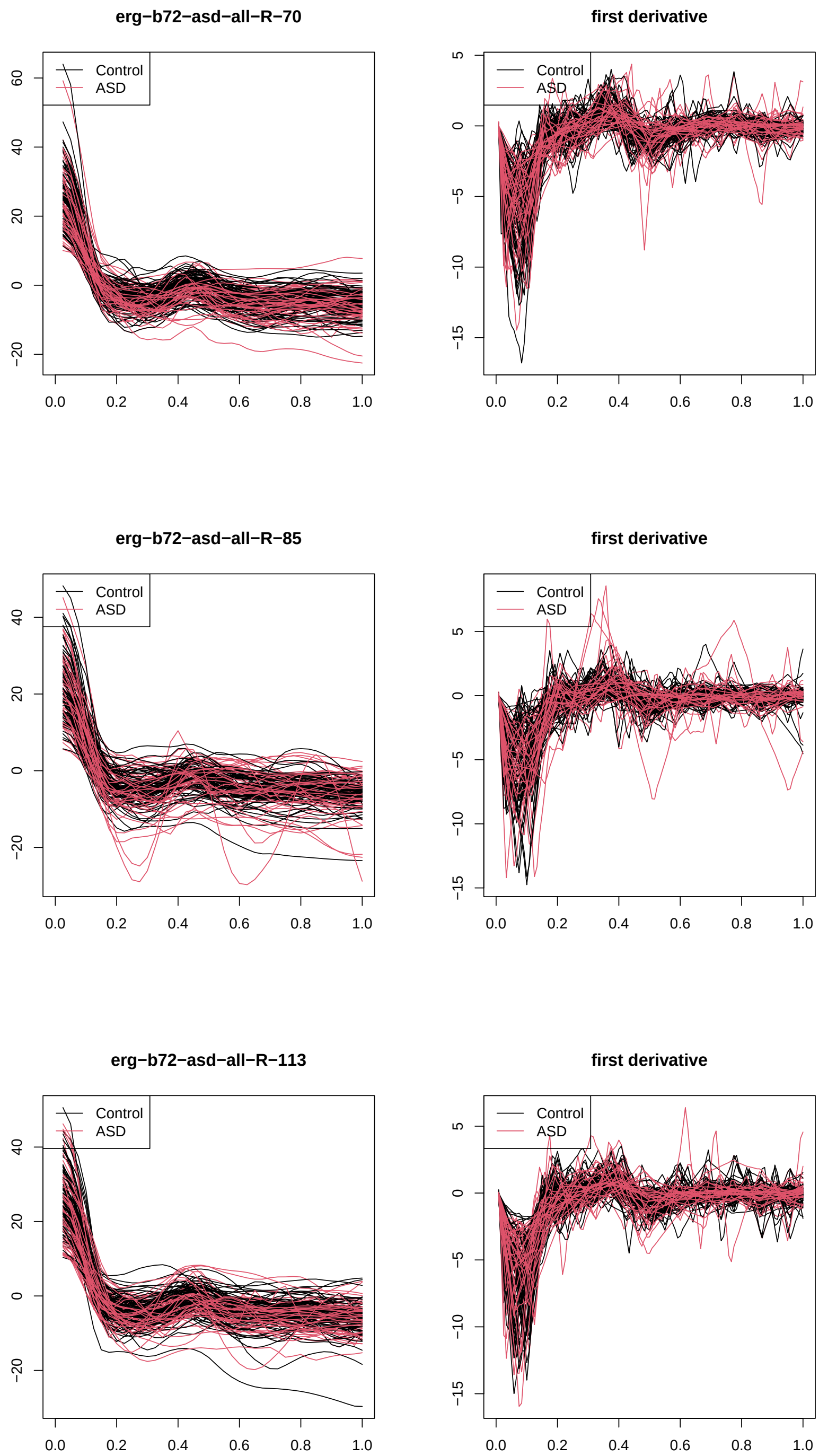
erg−b72−asd−all−R−70
Control
ASD
first derivative
Control
ASD
erg−b72−asd−all−R−85
Control
ASD
first derivative
Control
ASD
erg−b72−asd−all−R−113
Control
ASD
first derivative
Control
ASD

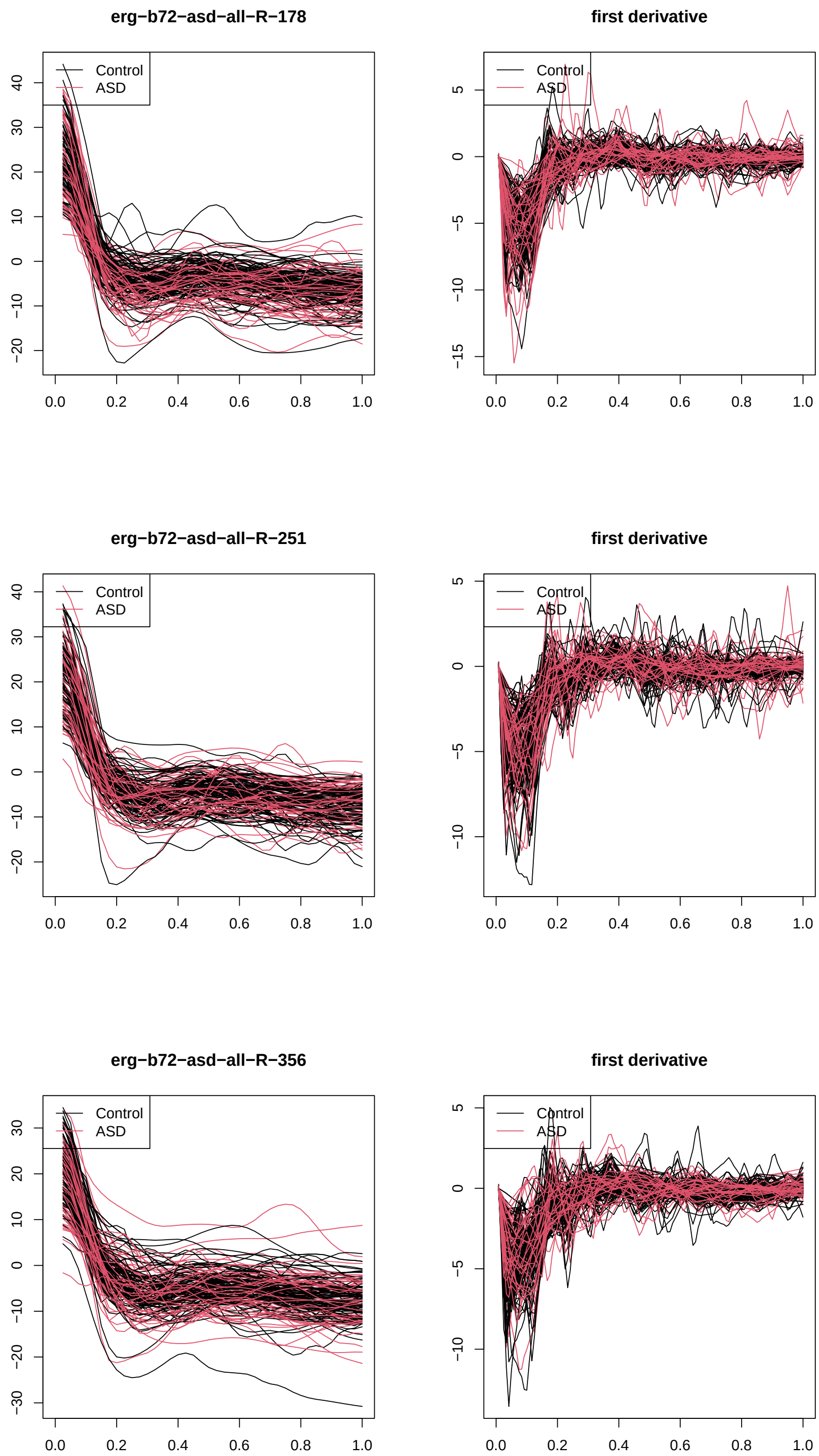
erg−b72−asd−all−R−178
first derivative
erg−b72−asd−all−R−251
first derivative
erg−b72−asd−all−R−356
first derivative
Control
ASD

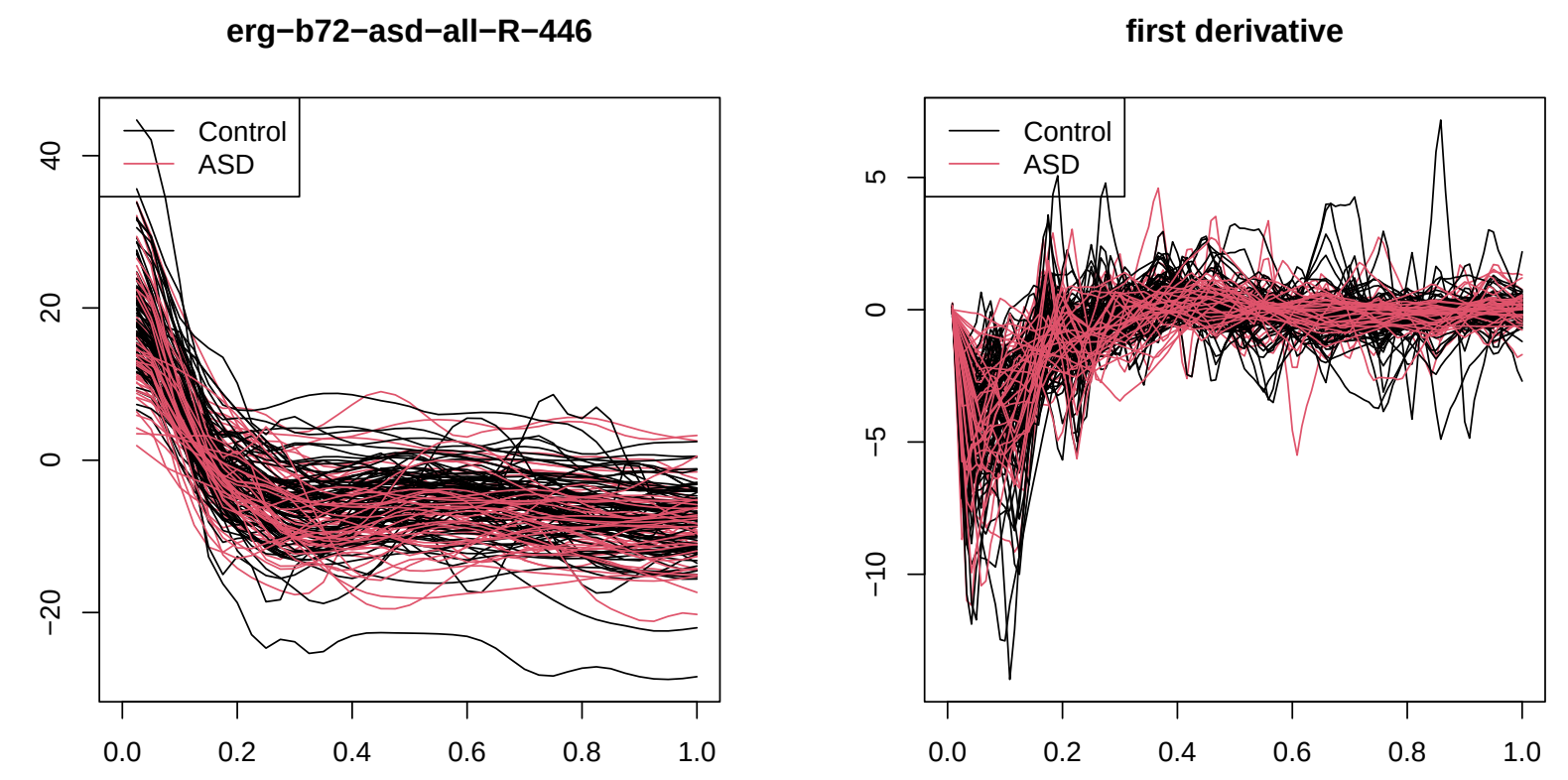

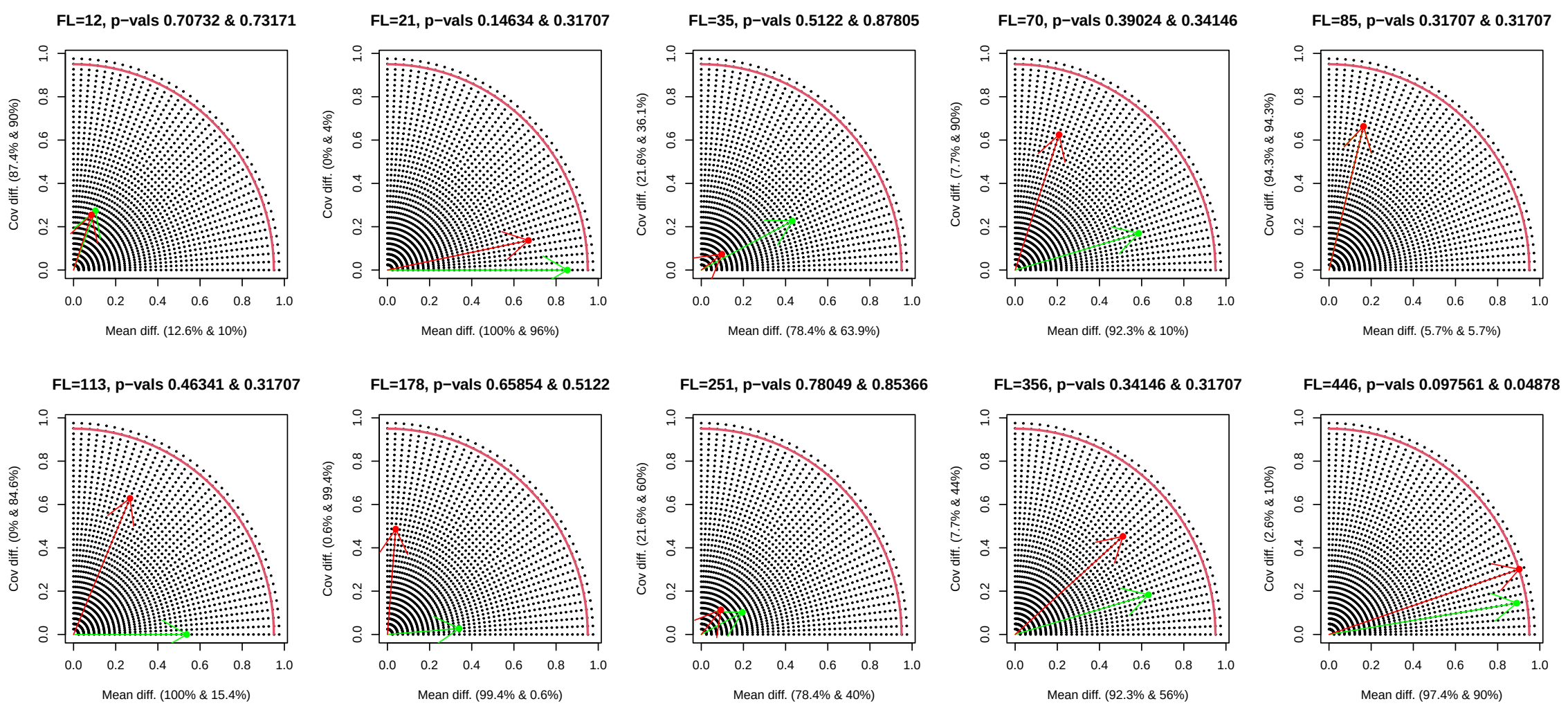

Table for ERG wave by asd in btime–72 – all obs.

	pval-erg-b72	mean perc	cov perc	pval-derivative	mean perc	cov perc
12	0.7073			0.7317		
21	0.1463			0.3171		
35	0.5122			0.8780		
70	0.3902			0.3415		
85	0.3171			0.3171		
113	0.4634			0.3171		
178	0.6585			0.5122		
251	0.7805			0.8537		
356	0.3415			0.3171		
446	0.0976			0.0488	90	10

2.5 OP wave by sex in 0–60 – ctr subgroup

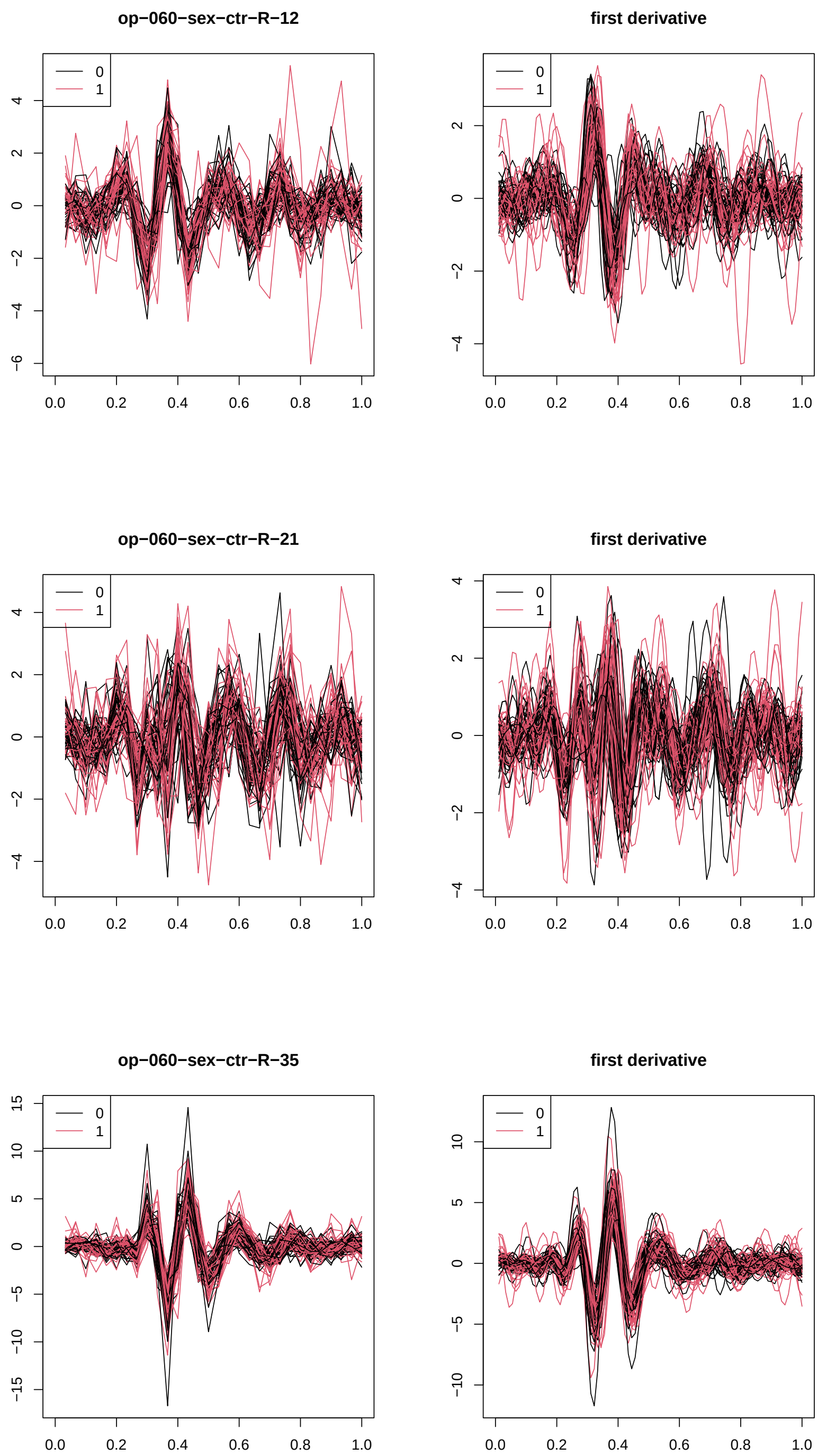

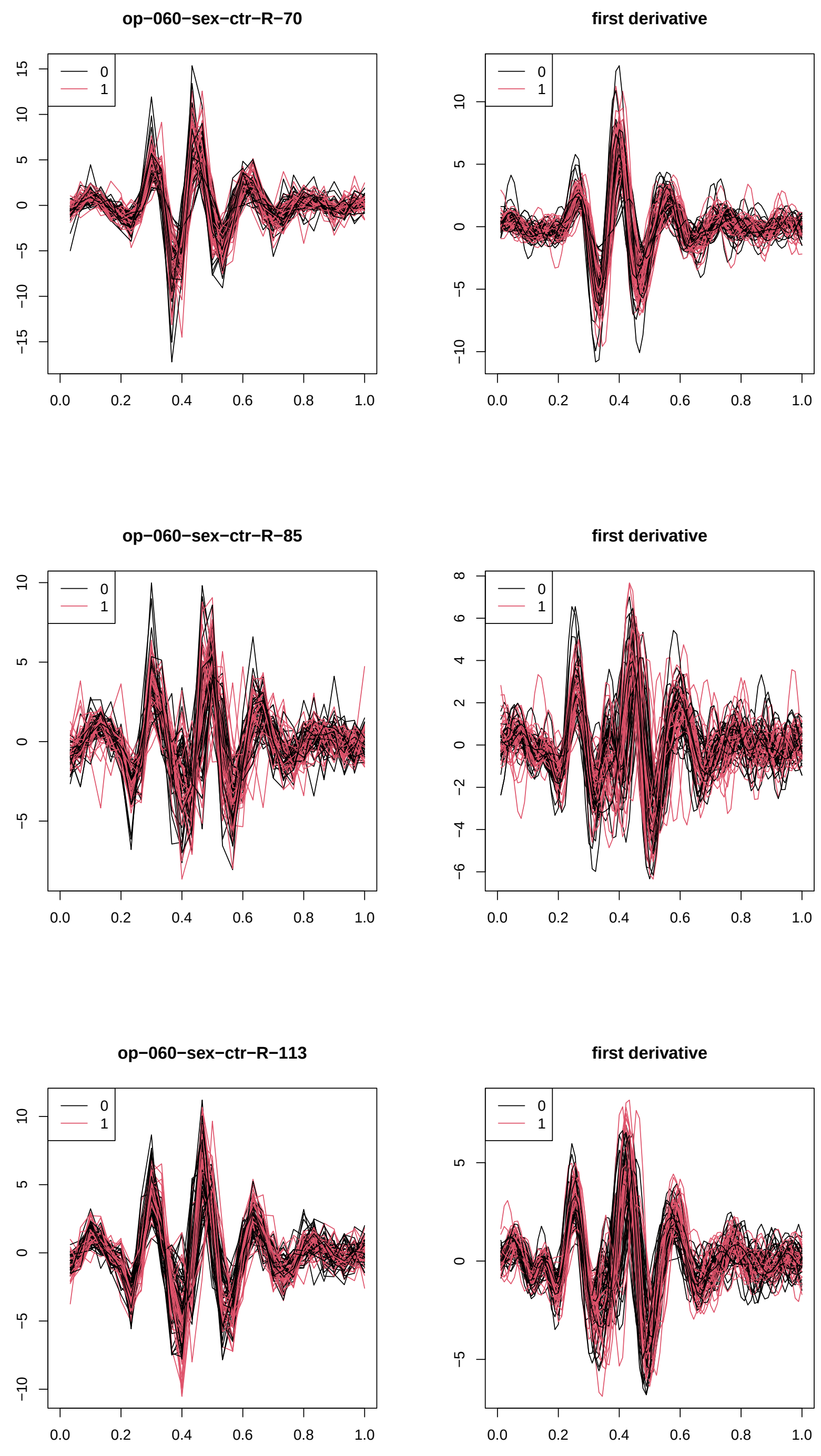
op−060−sex−ctr−R−70
first derivative
op−060−sex−ctr−R−85
first derivative
op−060−sex−ctr−R−113
first derivative
0
1

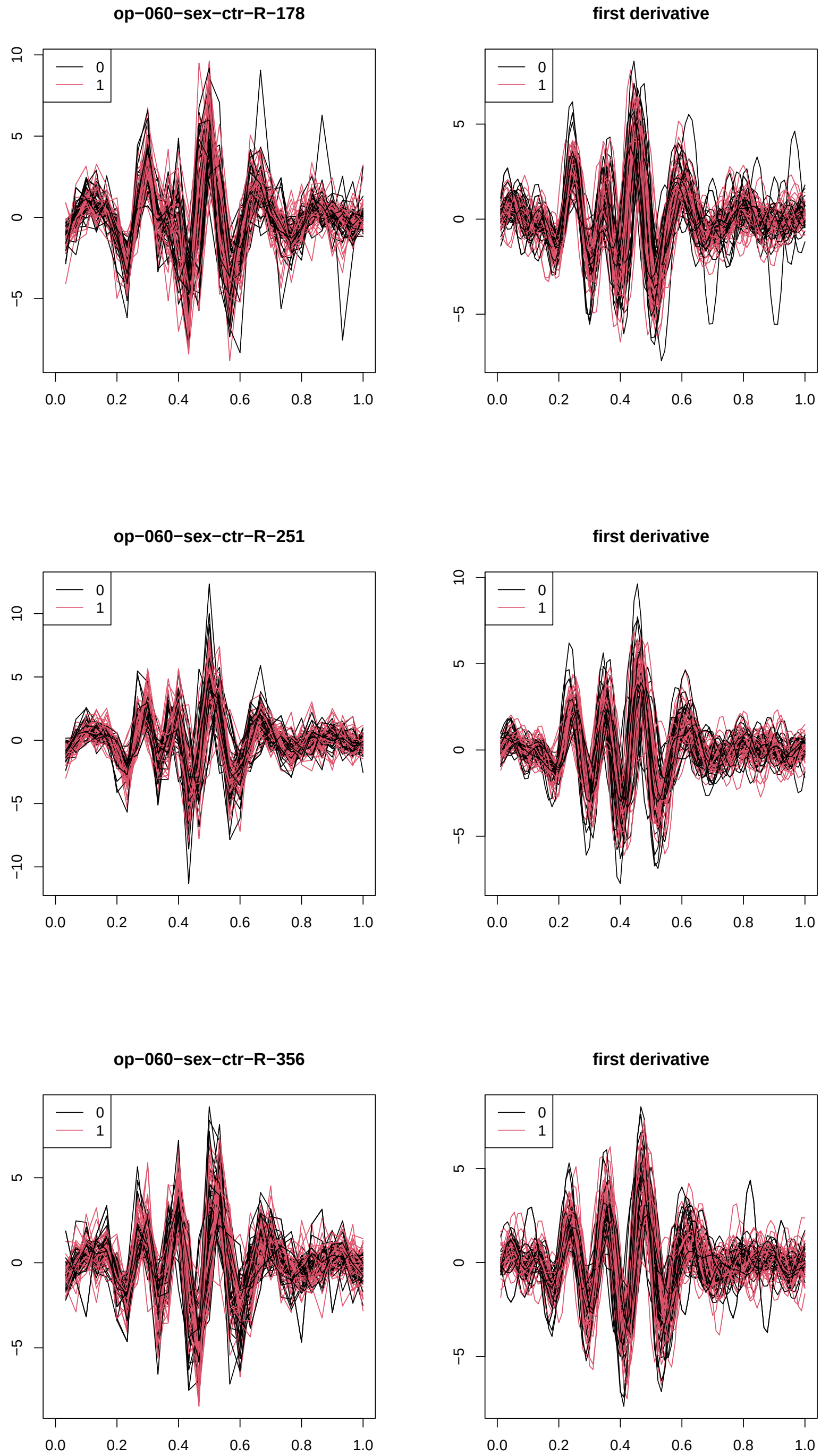
op−060−sex−ctr−R−178
first derivative
op−060−sex−ctr−R−251
first derivative
op−060−sex−ctr−R−356
first derivative
0
1

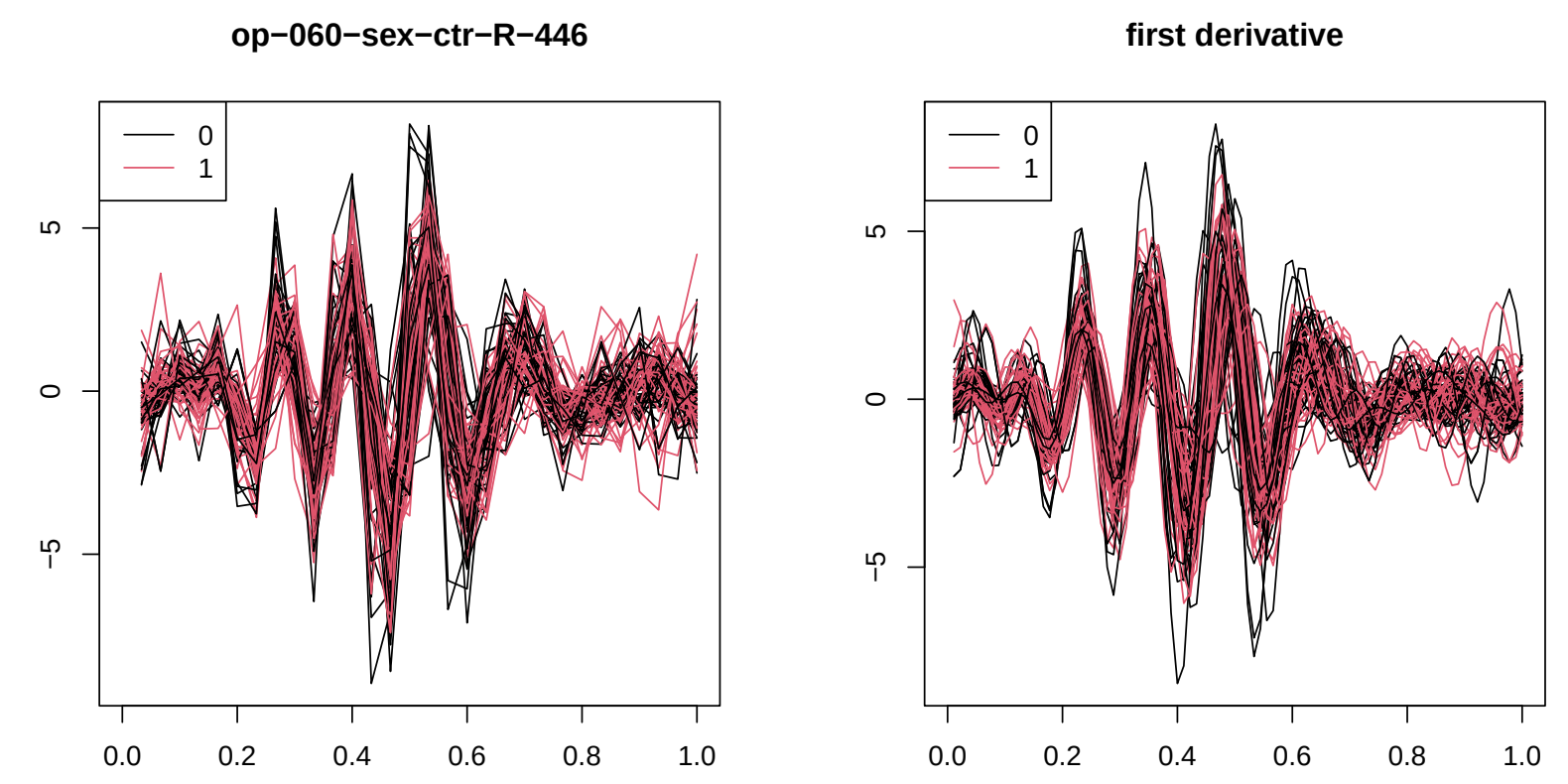

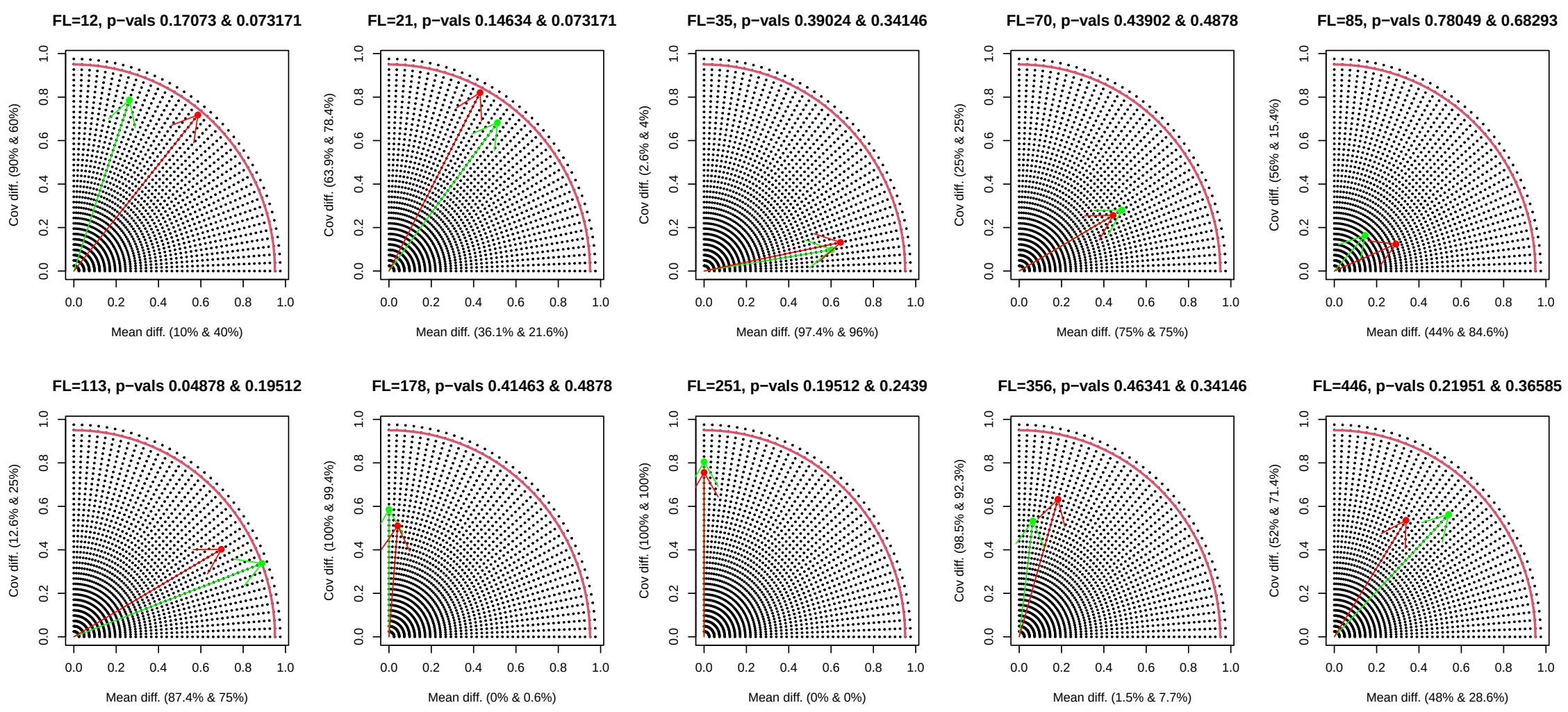

Table for OP wave by sex in 0–60 – ctr subgroup

	pval-op-060	mean perc	cov perc	pval-derivative	mean perc	cov perc
12	0.1707			0.0732		
21	0.1463			0.0732		
35	0.3902			0.3415		
70	0.4390			0.4878		
85	0.7805			0.6829		
113	0.0488	87.4	12.6	0.1951		
178	0.4146			0.4878		
251	0.1951			0.2439		
356	0.4634			0.3415		
446	0.2195			0.3659		

2.6 OP wave by sex in 0–60 – asd subgroup

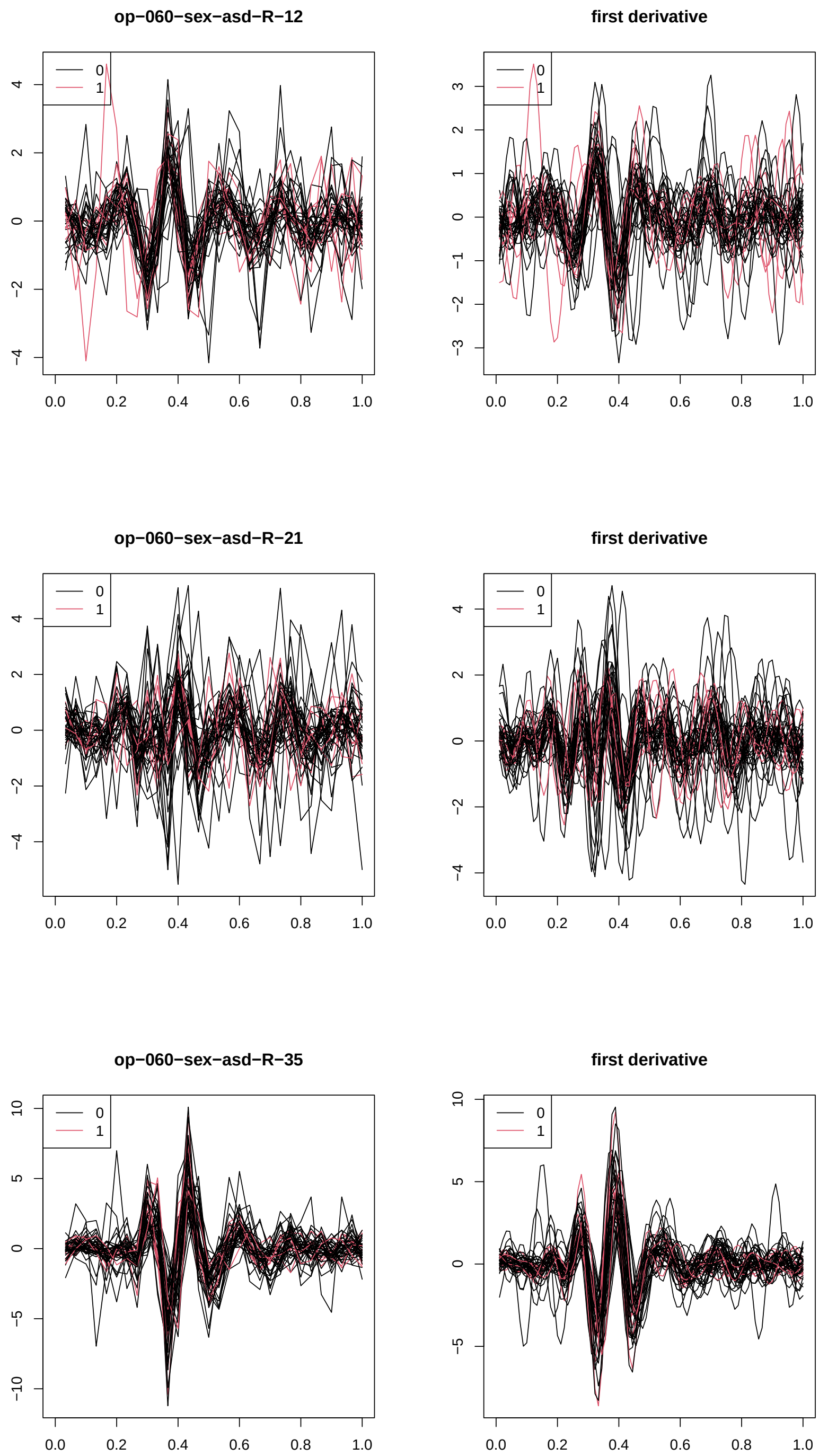

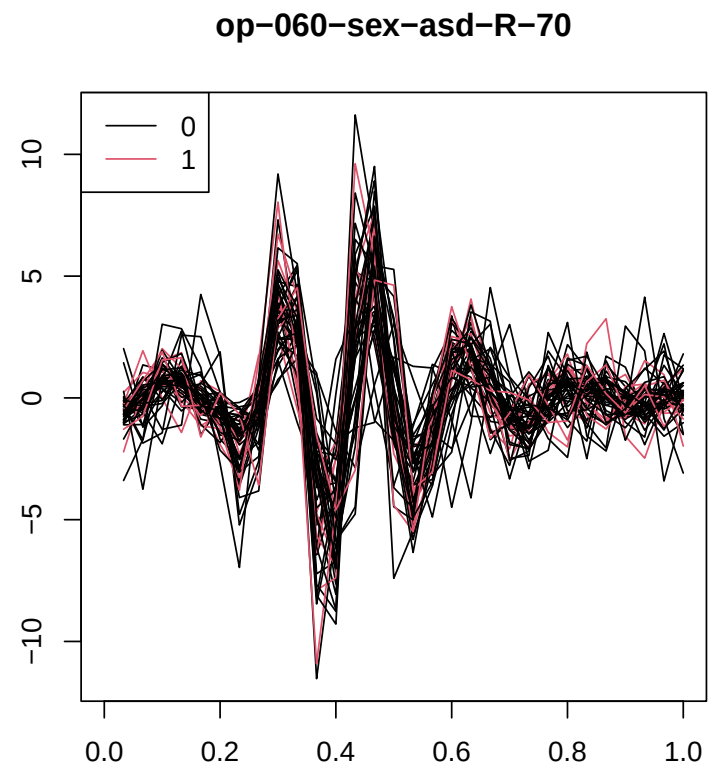
op−060−sex−asd−R−70
0
1

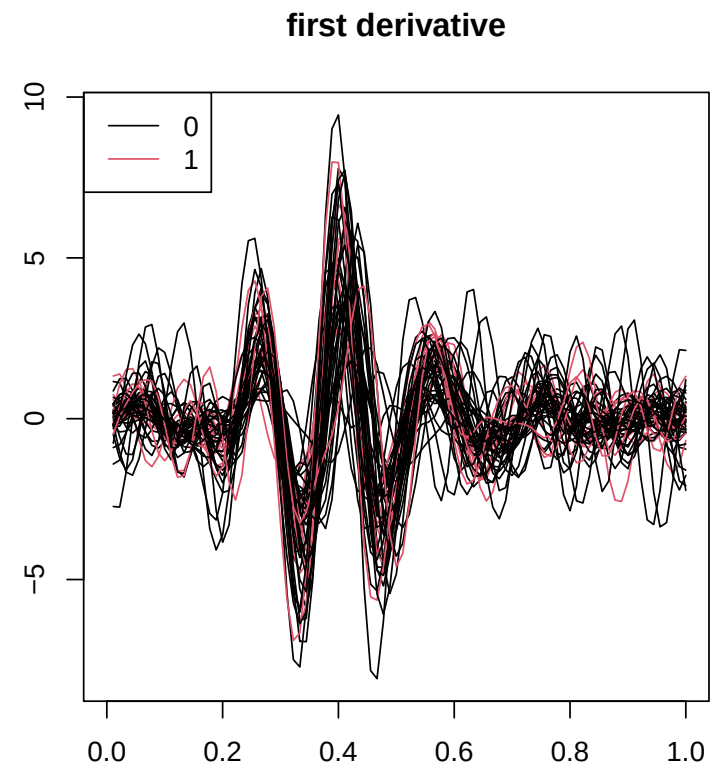
first derivative
0
1

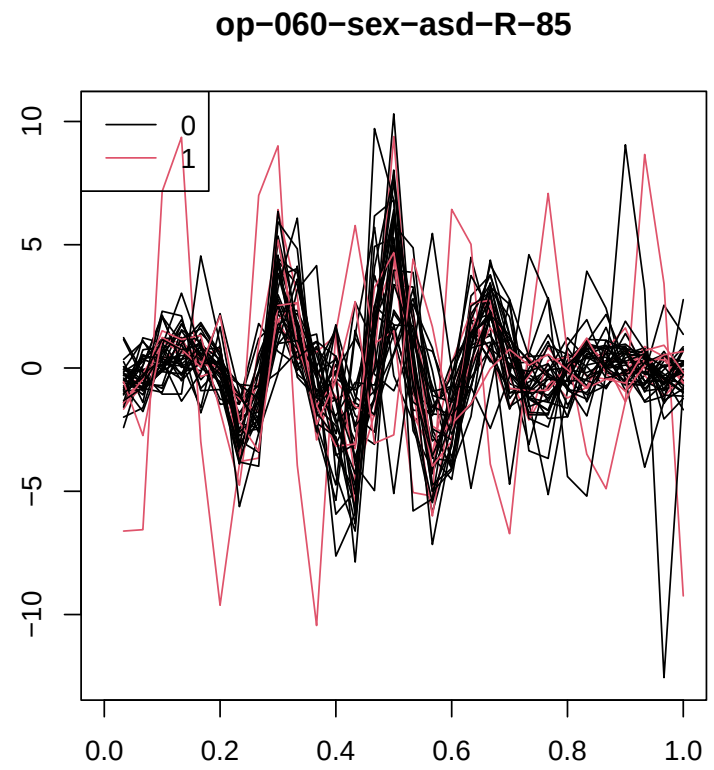
op−060−sex−asd−R−85
0
1

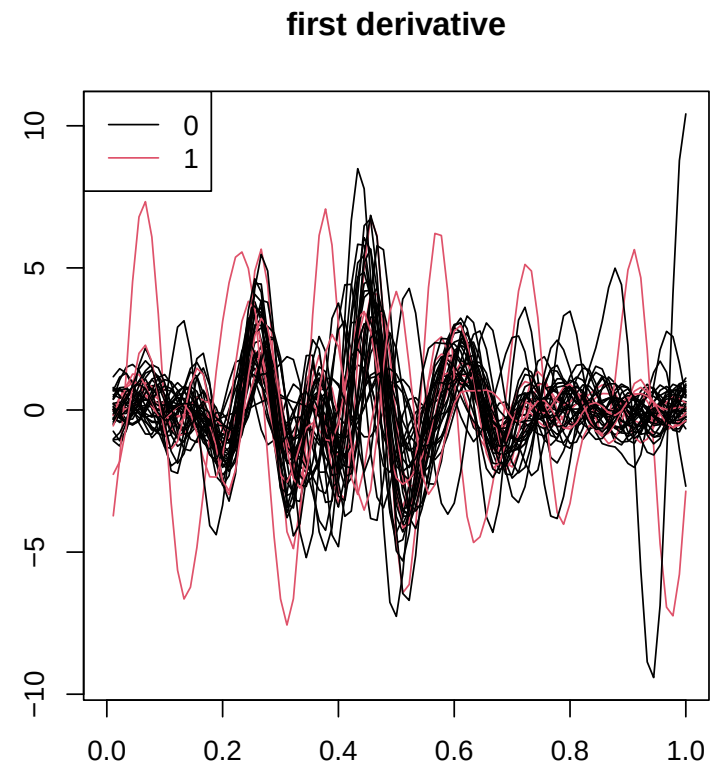
first derivative
0
1

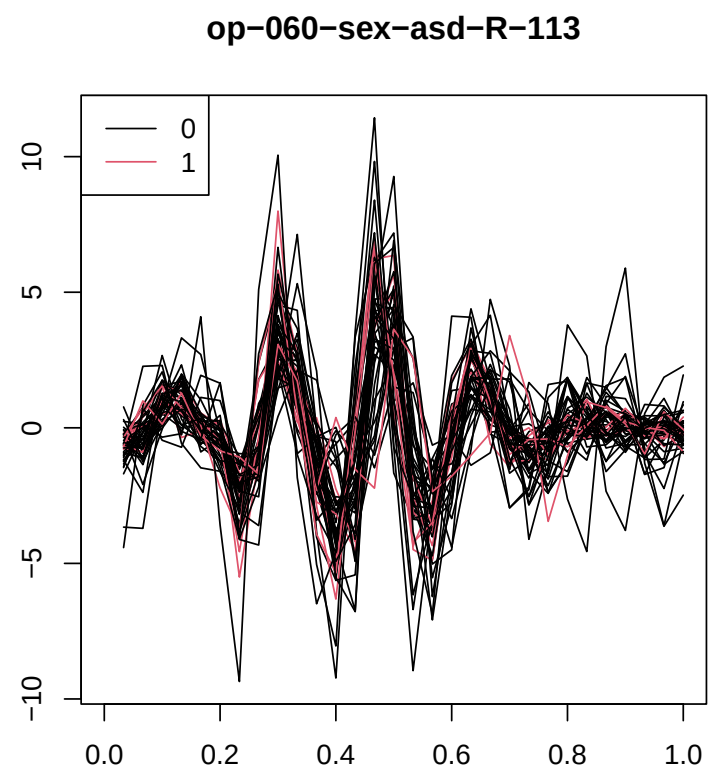
op−060−sex−asd−R−113
0
1

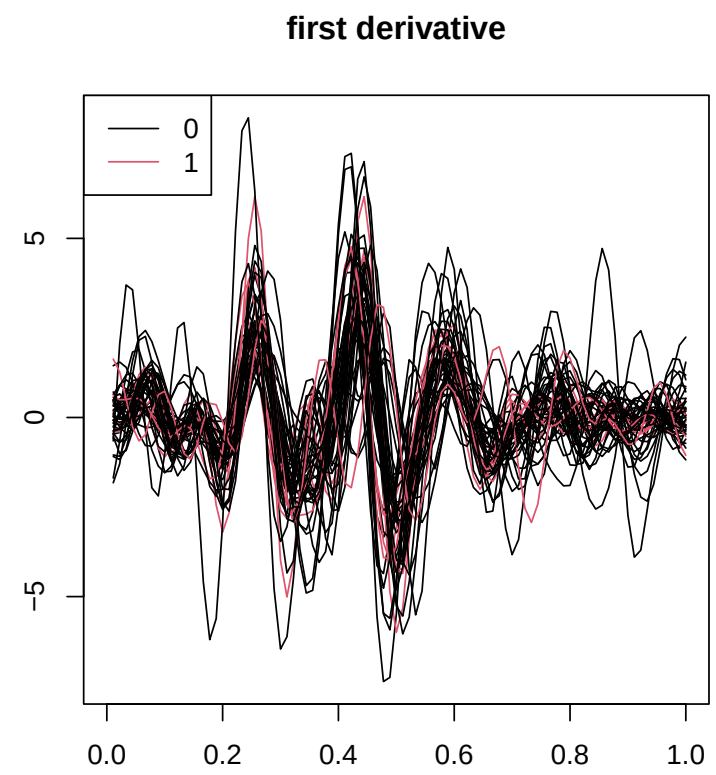
first derivative
0
1

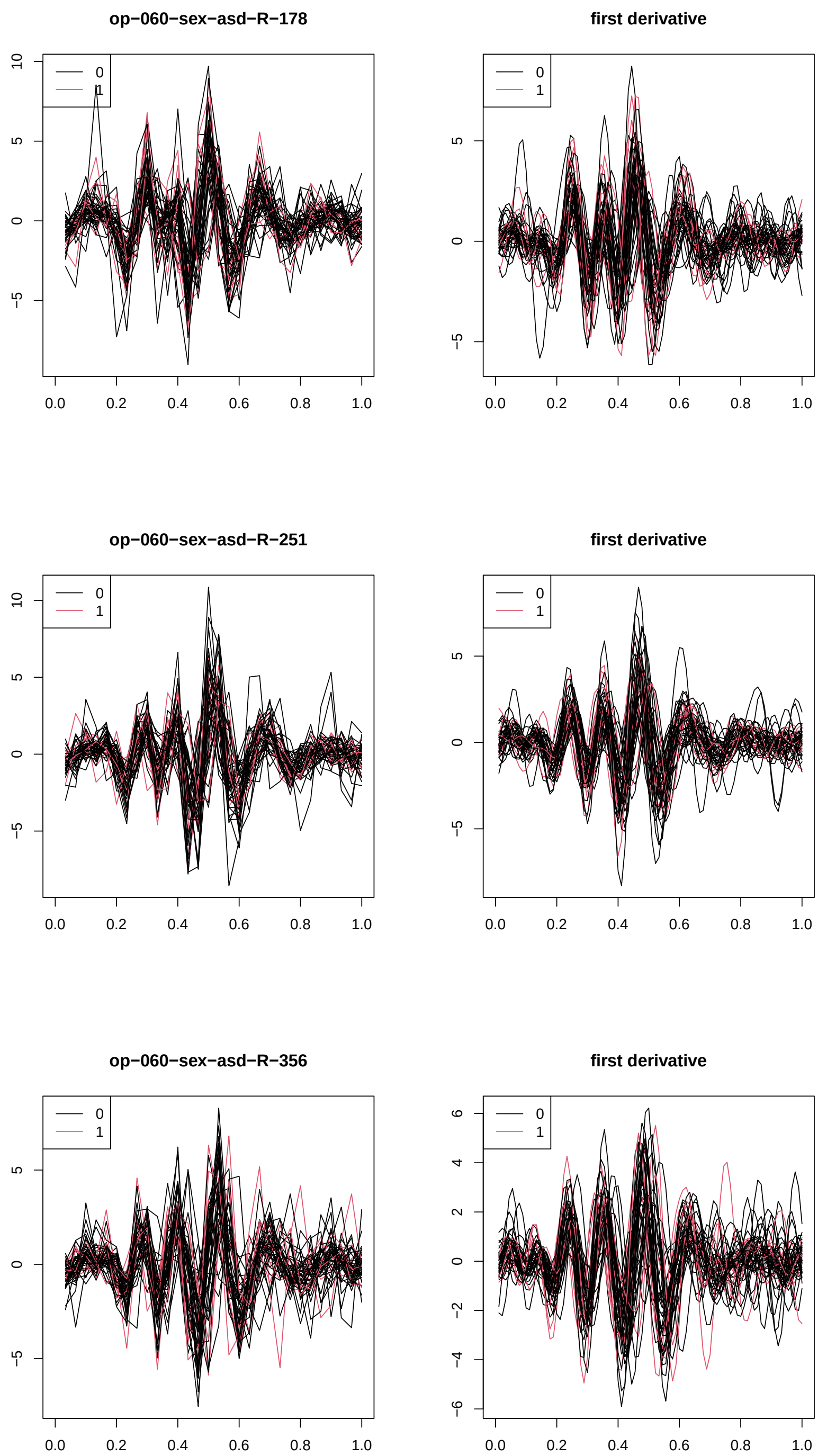
op−060−sex−asd−R−178
first derivative
op−060−sex−asd−R−251
first derivative
op−060−sex−asd−R−356
first derivative
0
1
0.0
0.2
0.4
0.6
0.8
1.0

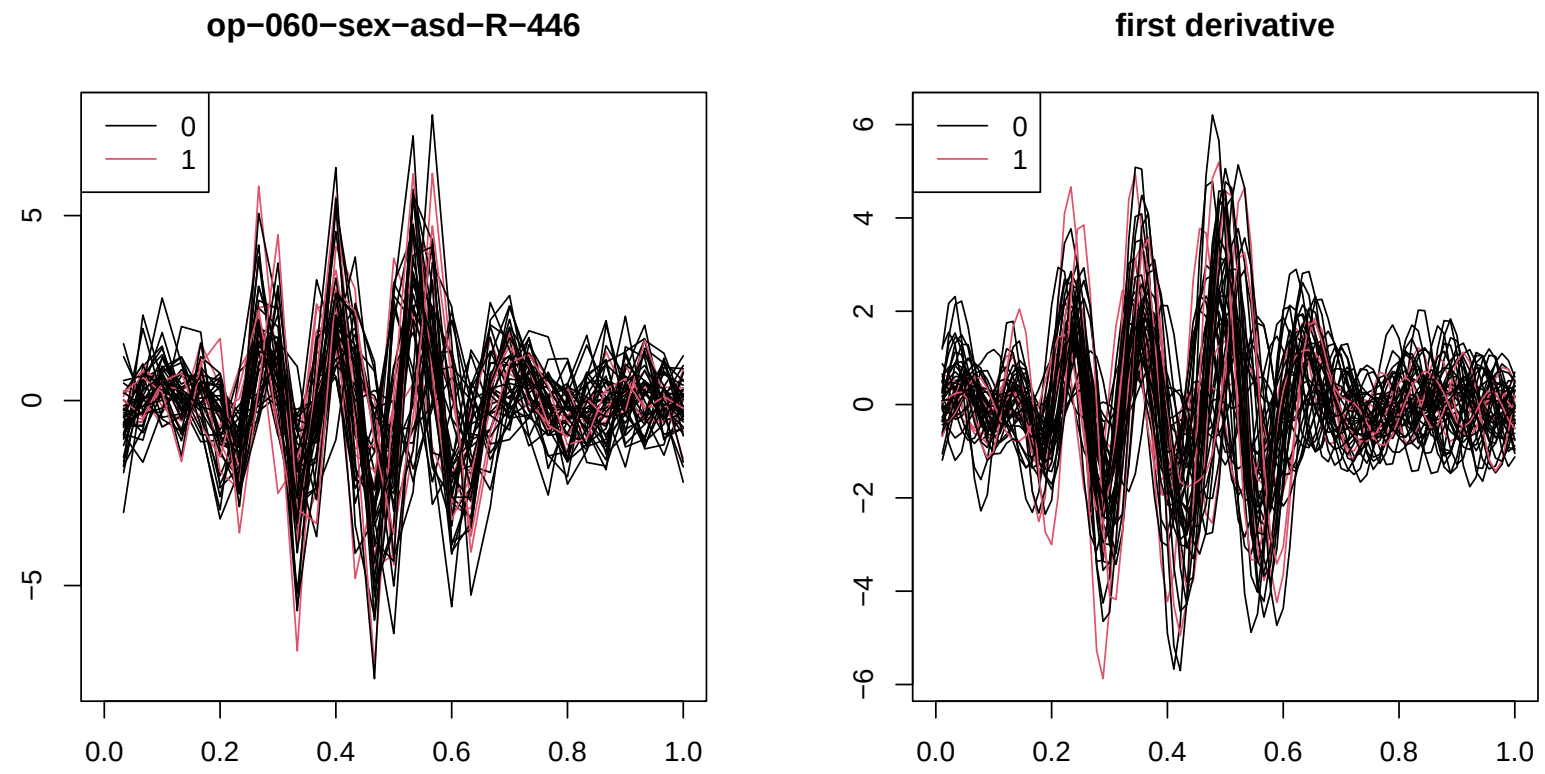

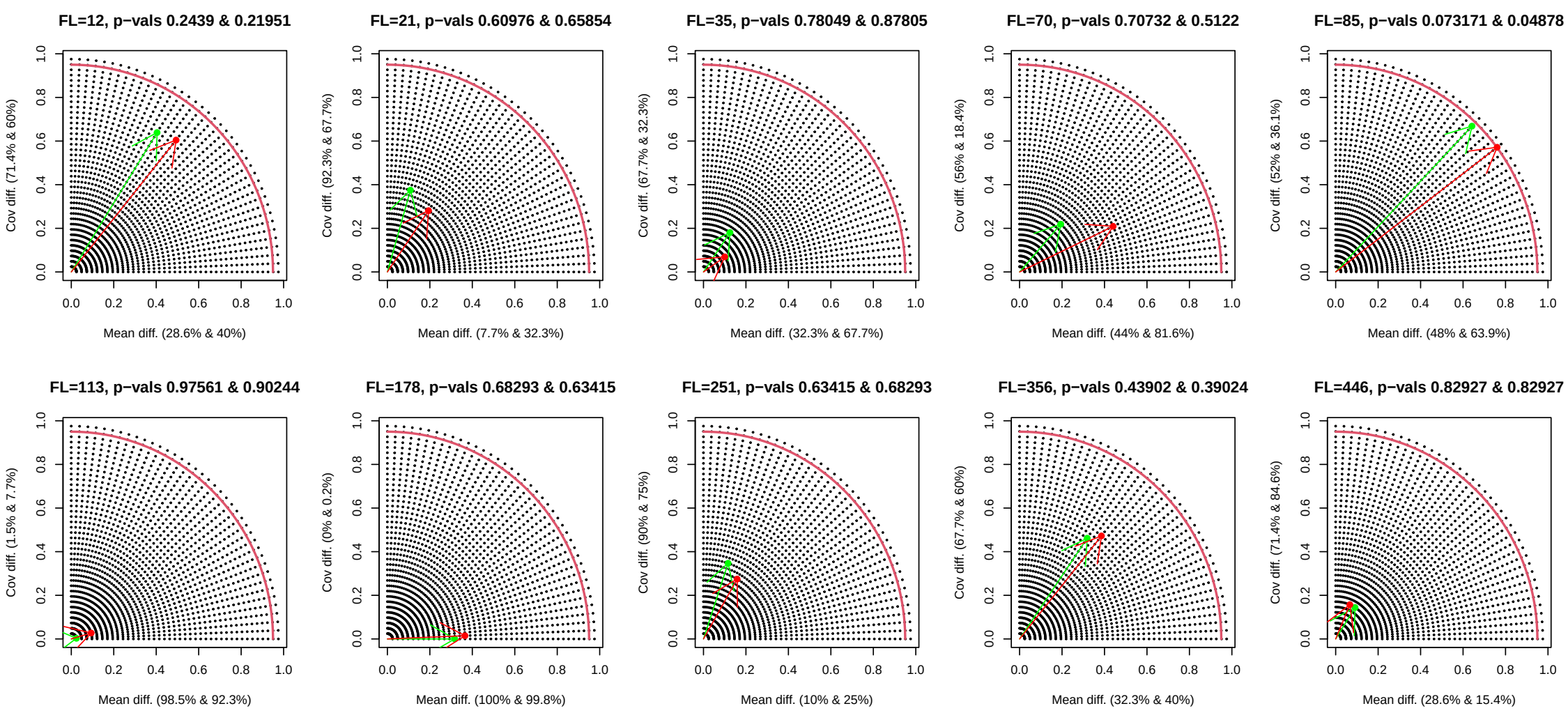

Table for OP wave by sex in 0–60 – asd subgroup

	pval-op-060	mean perc	cov perc	pval-derivative	mean perc	cov perc
12	0.2439			0.2195		
21	0.6098			0.6585		
35	0.7805			0.8780		
70	0.7073			0.5122		
85	0.0732			0.0488	63.9	36.1
113	0.9756			0.9024		
178	0.6829			0.6341		
251	0.6341			0.6829		
356	0.4390			0.3902		
446	0.8293			0.8293		

2.7 ERG wave by sex in 0–atime – ctr subgroup

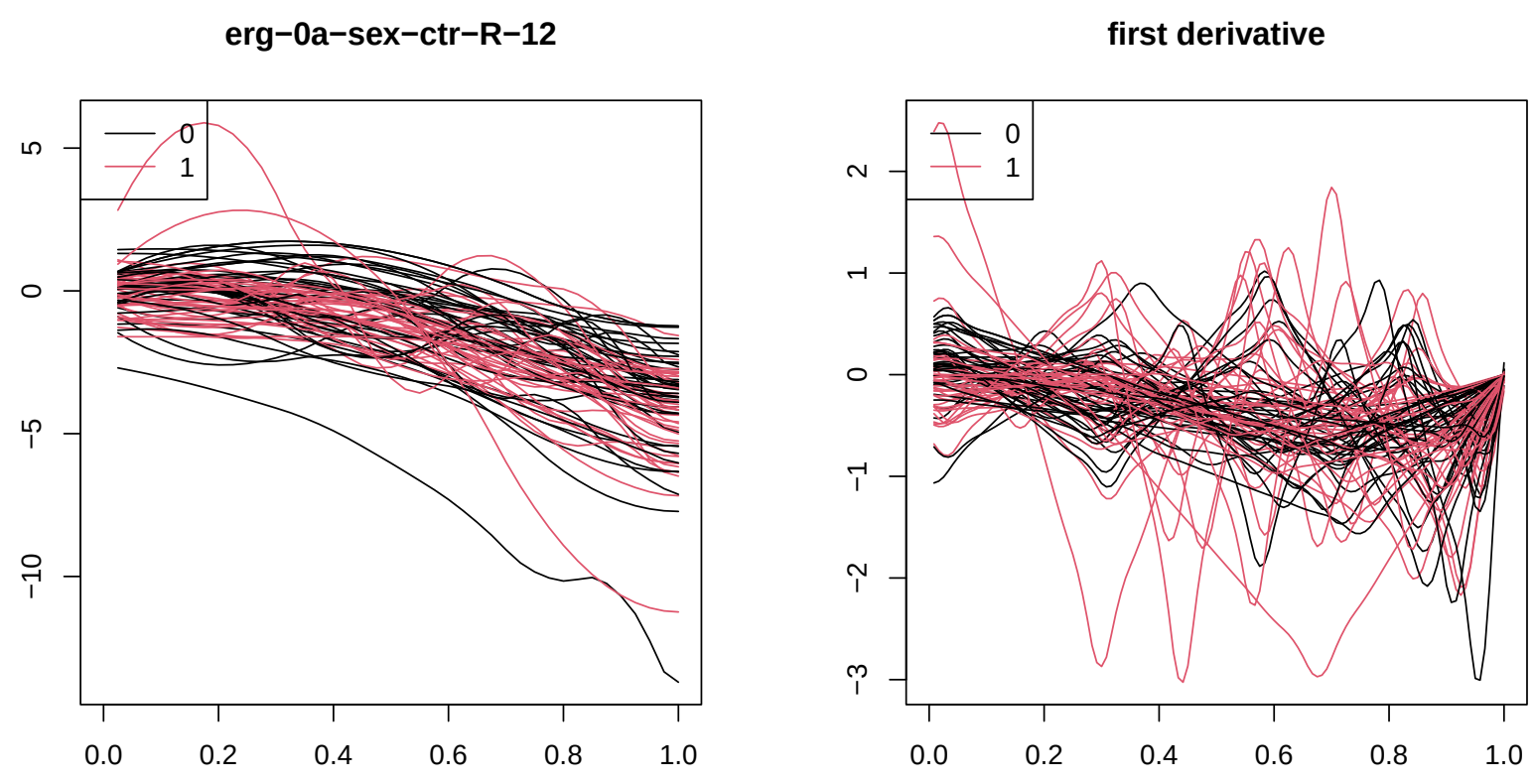

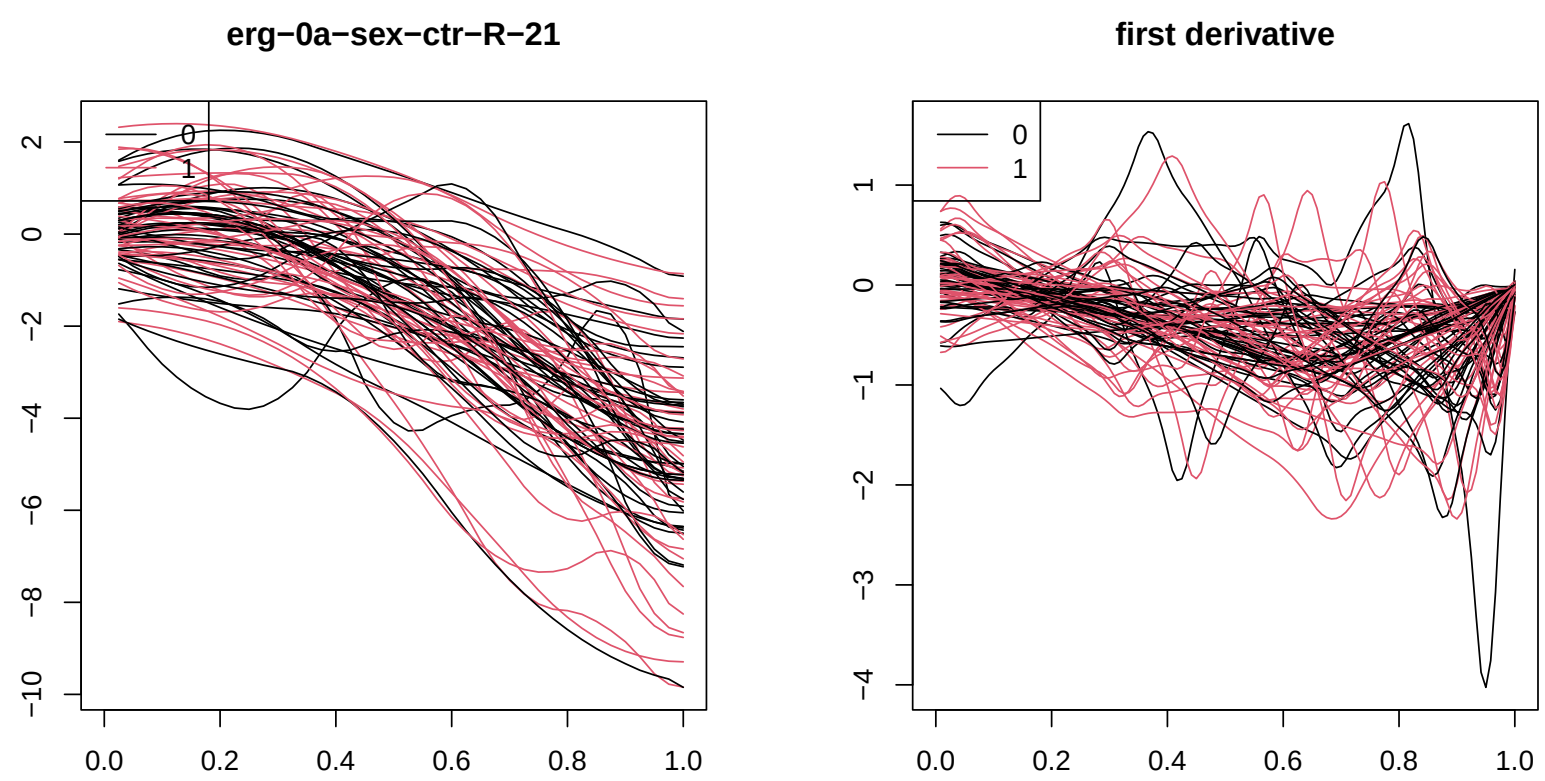

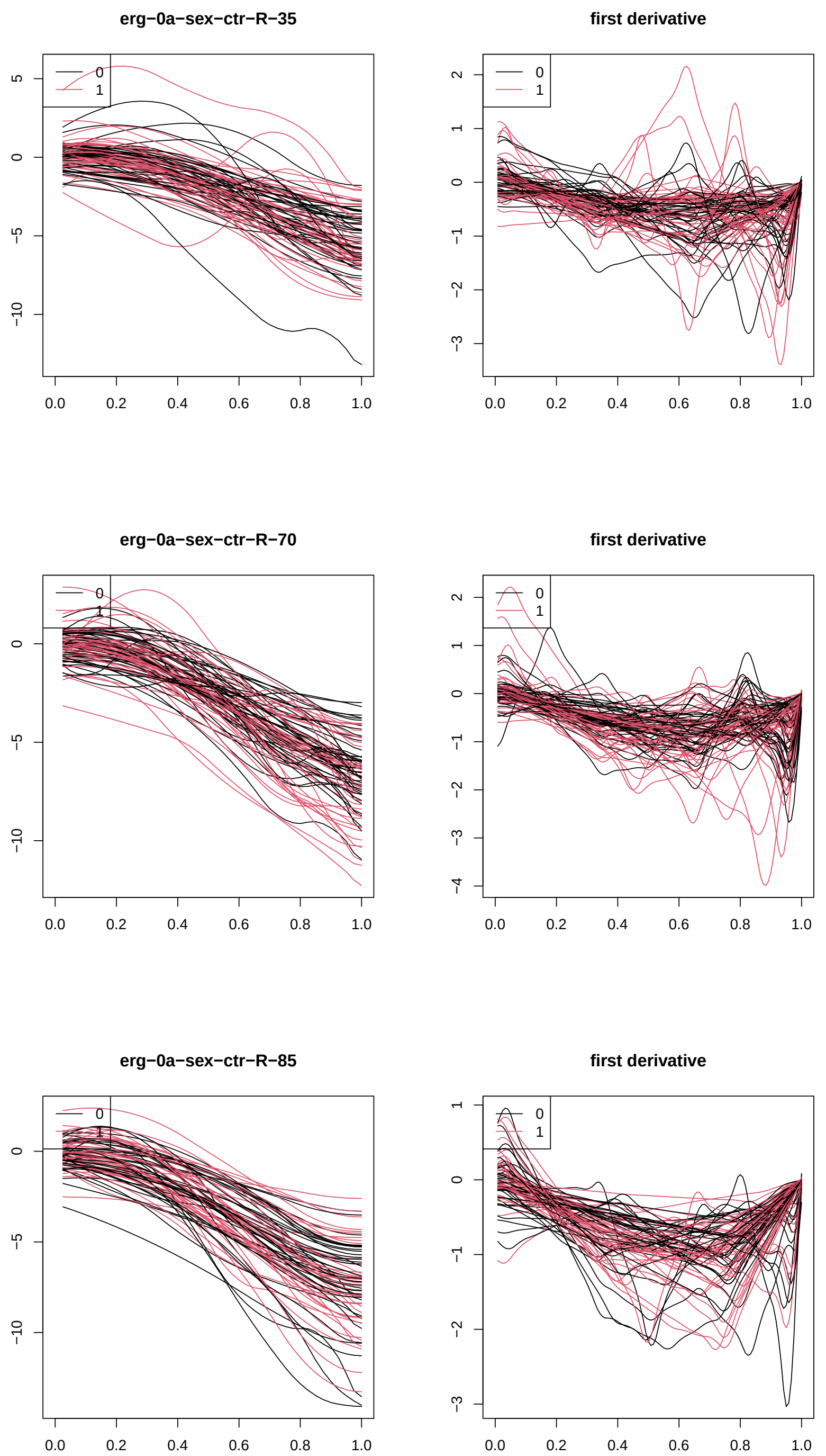
erg−0a−sex−ctr−R−35
first derivative
erg−0a−sex−ctr−R−70
first derivative
erg−0a−sex−ctr−R−85
first derivative
0
1

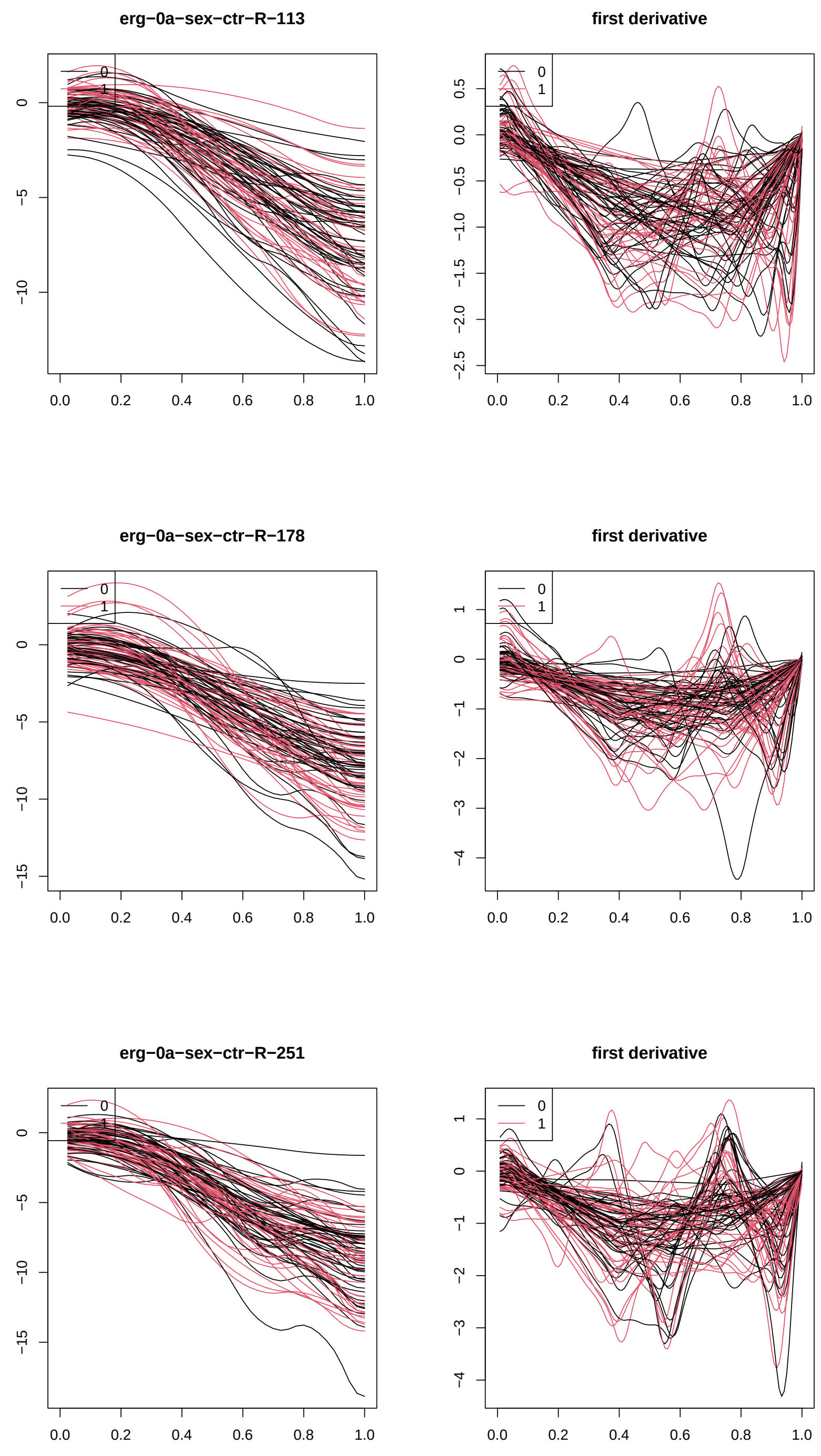
erg−0a−sex−ctr−R−113
first derivative
erg−0a−sex−ctr−R−178
first derivative
erg−0a−sex−ctr−R−251
first derivative
0
1

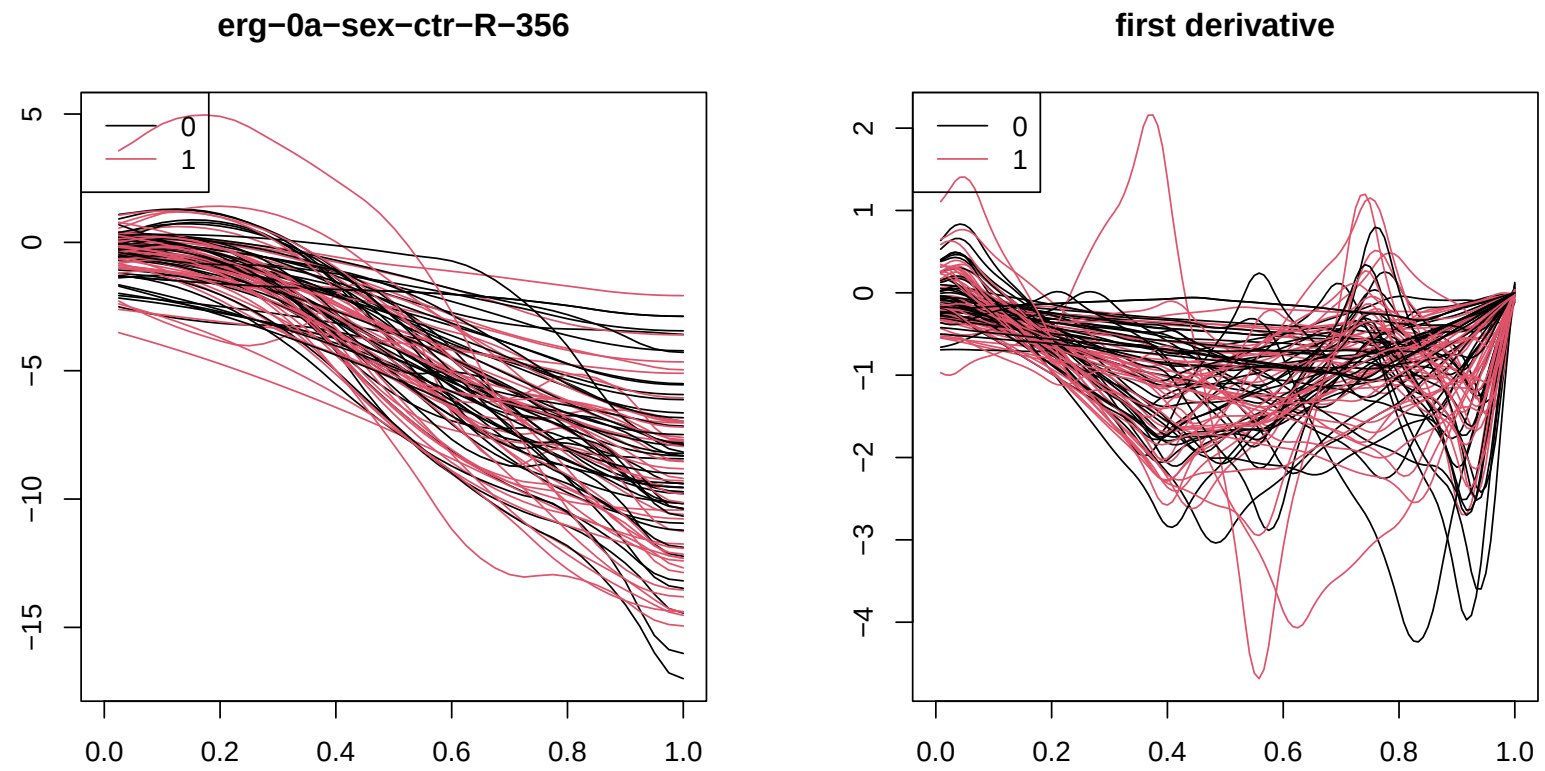
erg−0a−sex−ctr−R−356
first derivative
0
1

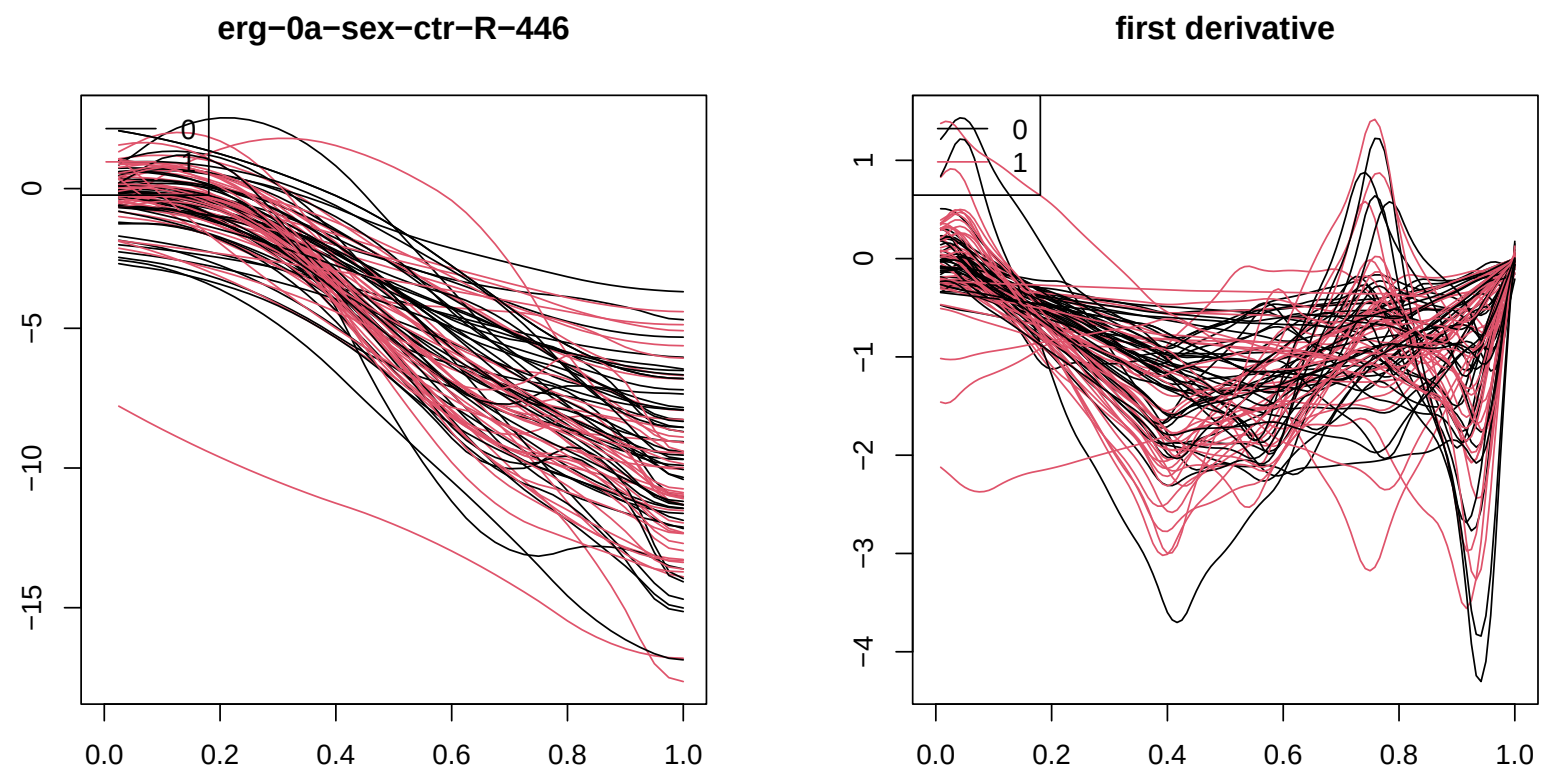
erg−0a−sex−ctr−R−446
first derivative
0
1

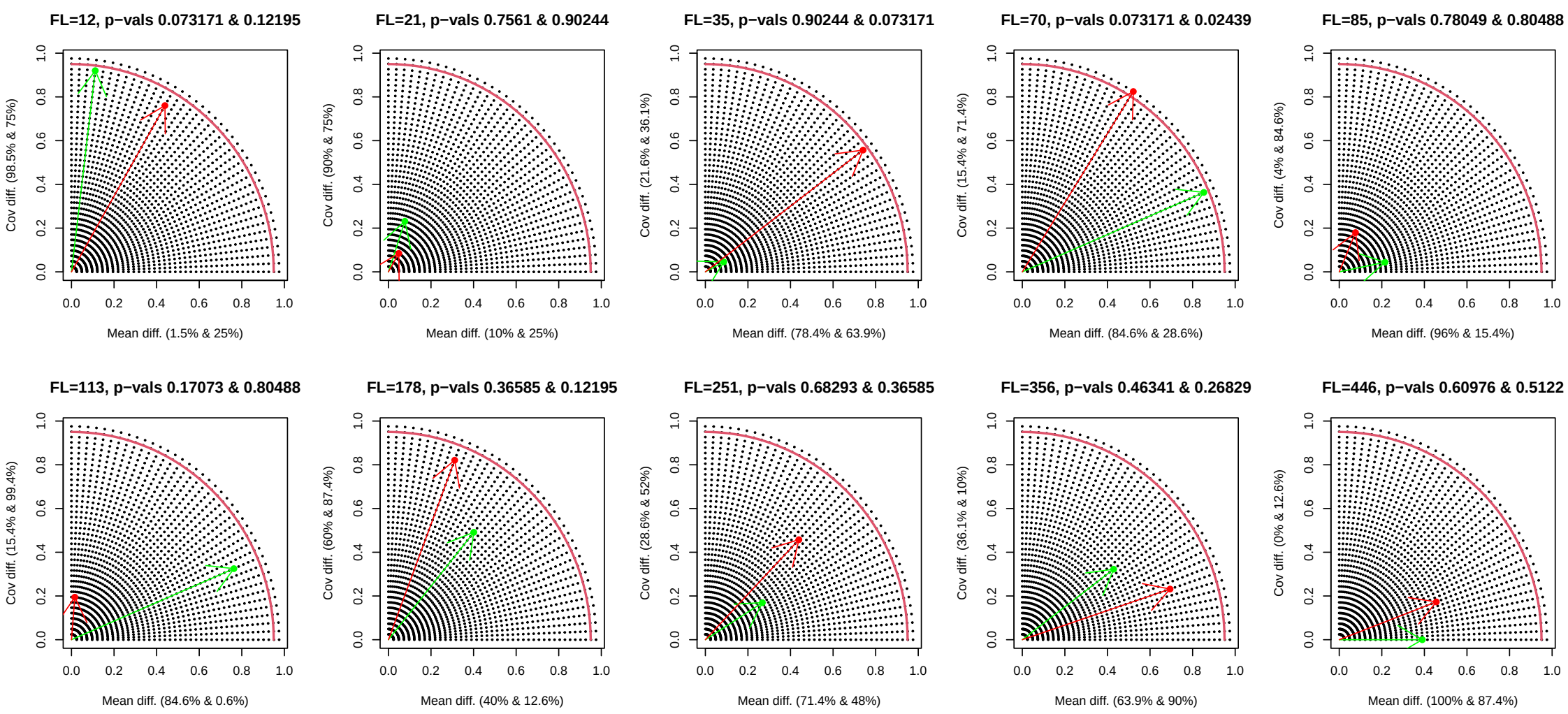
FL=12, p−vals 0.073171 & 0.12195
Mean diff. (1.5% & 25%)
Cov diff. (98.5% & 75%)
FL=21, p−vals 0.7561 & 0.90244
Mean diff. (10% & 25%)
Cov diff. (90% & 75%)
FL=35, p−vals 0.90244 & 0.073171
Mean diff. (78.4% & 63.9%)
Cov diff. (21.6% & 36.1%)
FL=70, p−vals 0.073171 & 0.02439
Mean diff. (84.6% & 28.6%)
Cov diff. (15.4% & 71.4%)
FL=85, p−vals 0.78049 & 0.80488
Mean diff. (96% & 15.4%)
Cov diff. (4% & 84.6%)
FL=113, p−vals 0.17073 & 0.80488
Mean diff. (84.6% & 0.6%)
Cov diff. (15.4% & 99.4%)
FL=178, p−vals 0.36585 & 0.12195
Mean diff. (40% & 12.6%)
Cov diff. (60% & 87.4%)
FL=251, p−vals 0.68293 & 0.36585
Mean diff. (71.4% & 48%)
Cov diff. (28.6% & 52%)
FL=356, p−vals 0.46341 & 0.26829
Mean diff. (63.9% & 90%)
Cov diff. (36.1% & 10%)
FL=446, p−vals 0.60976 & 0.5122
Mean diff. (100% & 87.4%)
Cov diff. (0% & 12.6%)

Table for ERG wave by sex in 0–atime – ctr subgroup

	pval-erg-0a	mean perc	cov perc	pval-derivative	mean perc	cov perc
12	0.0732			0.1220		
21	0.7561			0.9024		
35	0.9024			0.0732		
70	0.0732			0.0244	28.6	71.4
85	0.7805			0.8049		
113	0.1707			0.8049		
178	0.3659			0.1220		
251	0.6829			0.3659		
356	0.4634			0.2683		
446	0.6098			0.5122		

2.8 ERG wave by sex in 0–atime – asd subgroup

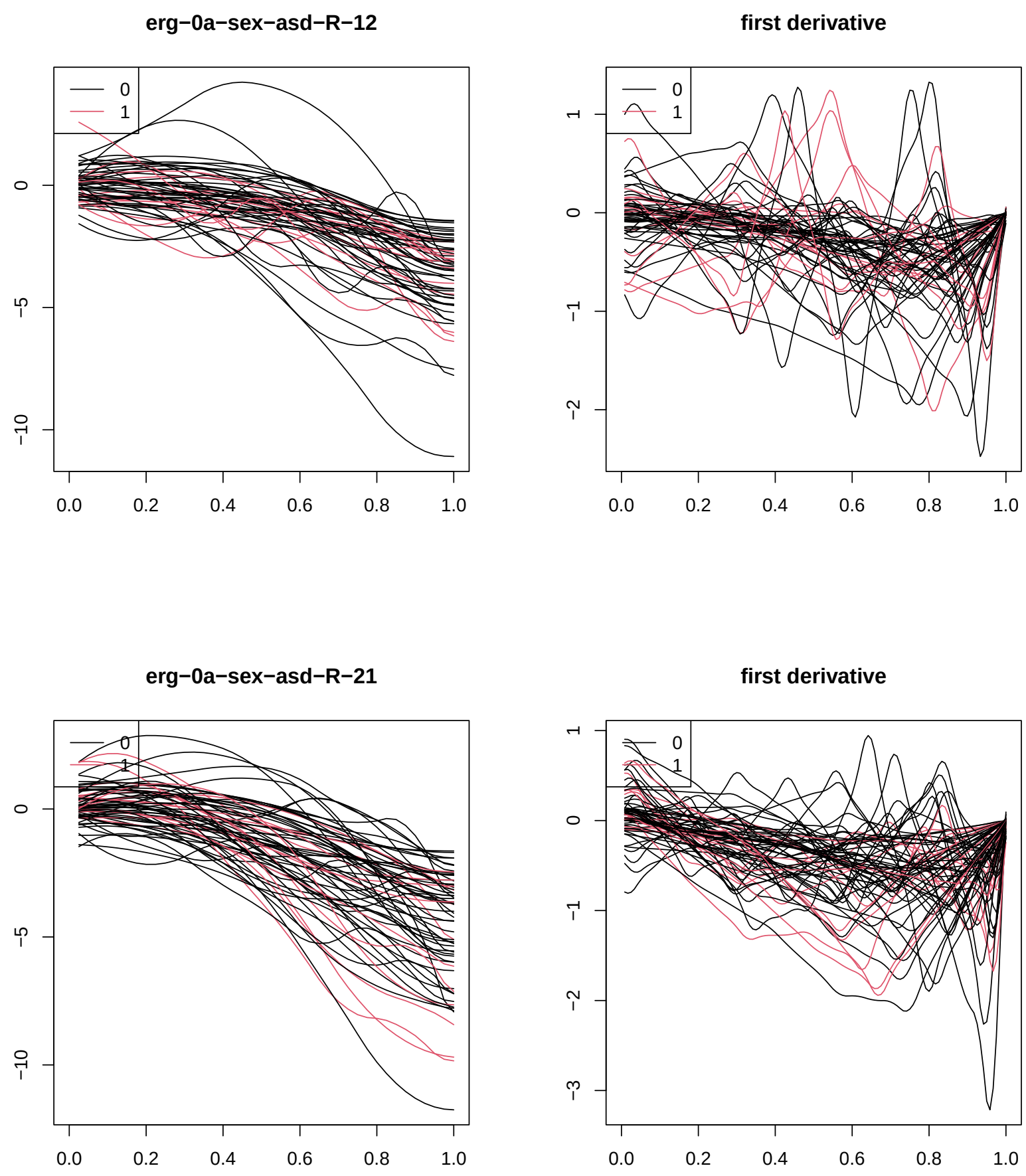

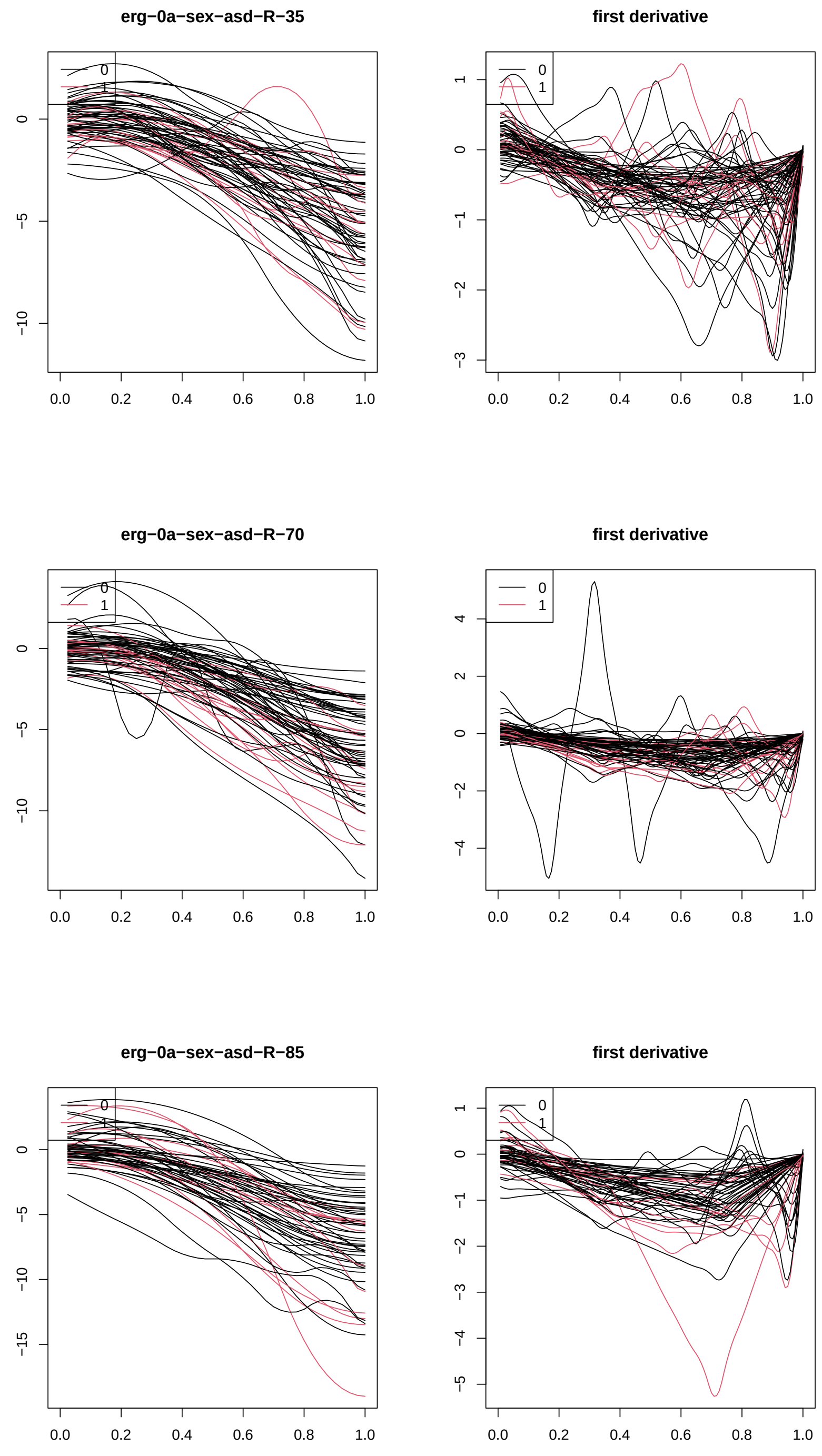
erg−0a−sex−asd−R−35
first derivative
erg−0a−sex−asd−R−70
first derivative
erg−0a−sex−asd−R−85
first derivative

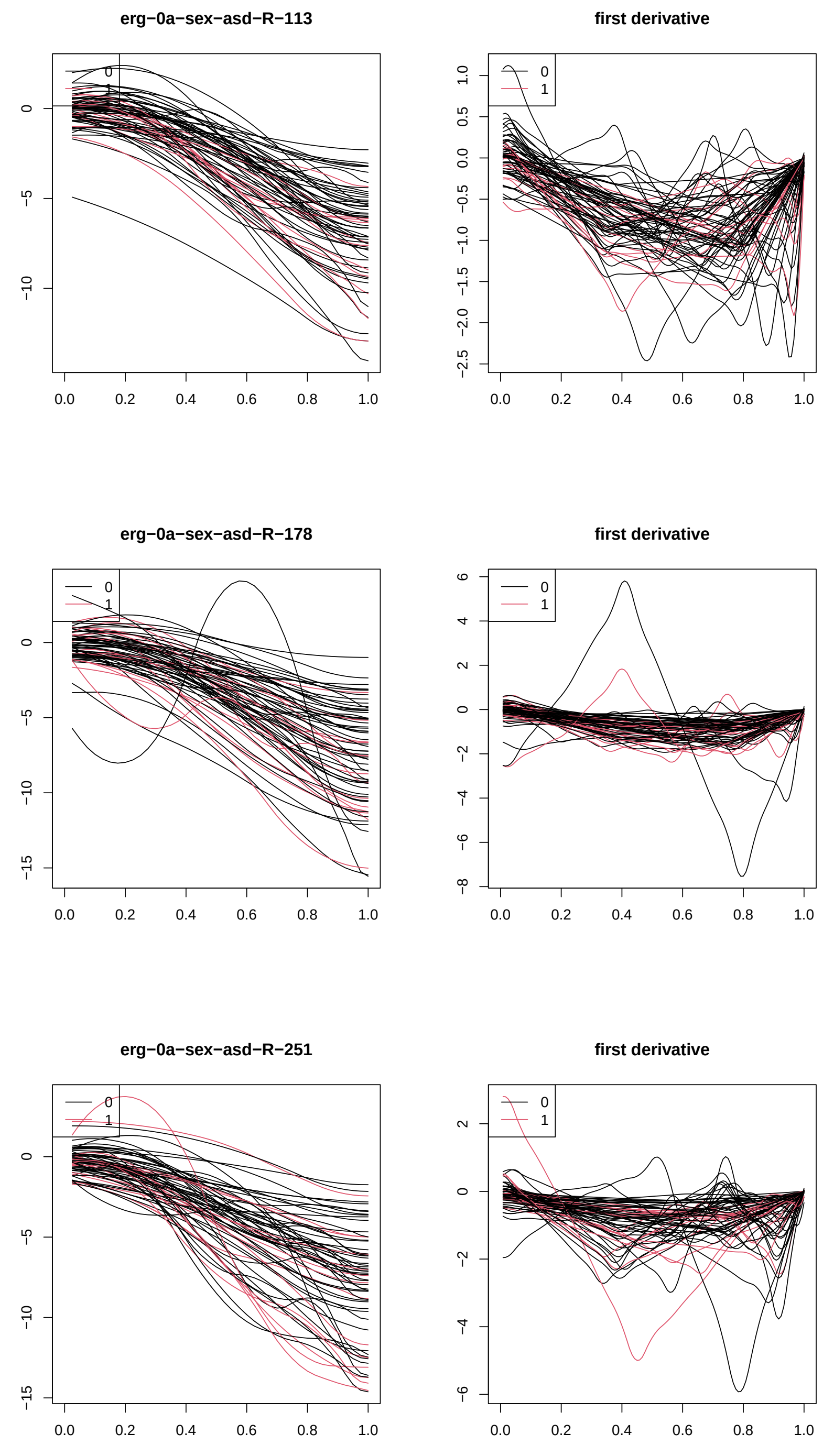

erg−0a−sex−asd−R−113
first derivative
erg−0a−sex−asd−R−178
first derivative
erg−0a−sex−asd−R−251
first derivative
0
1

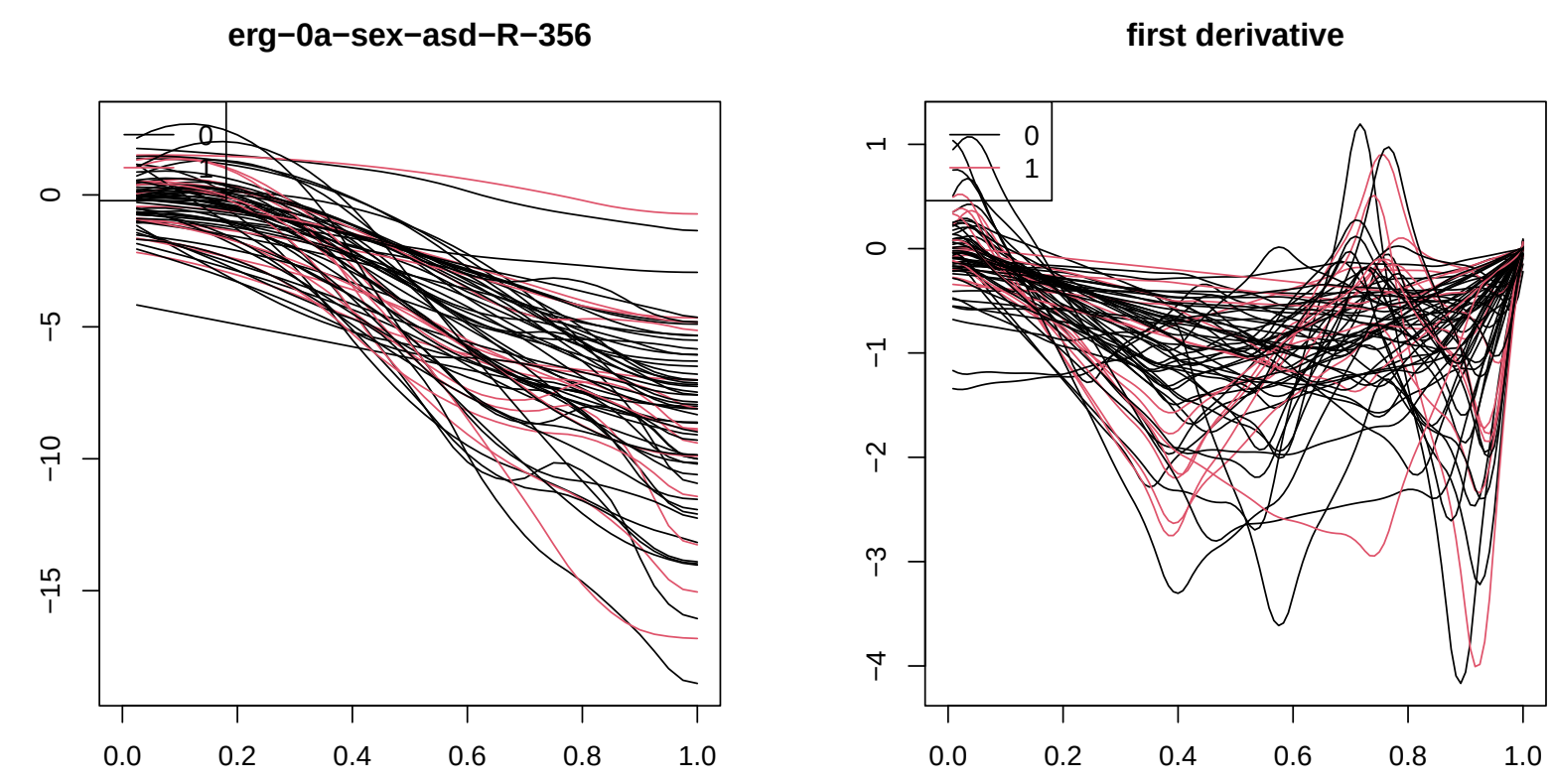
erg−0a−sex−asd−R−356
first derivative
0
1

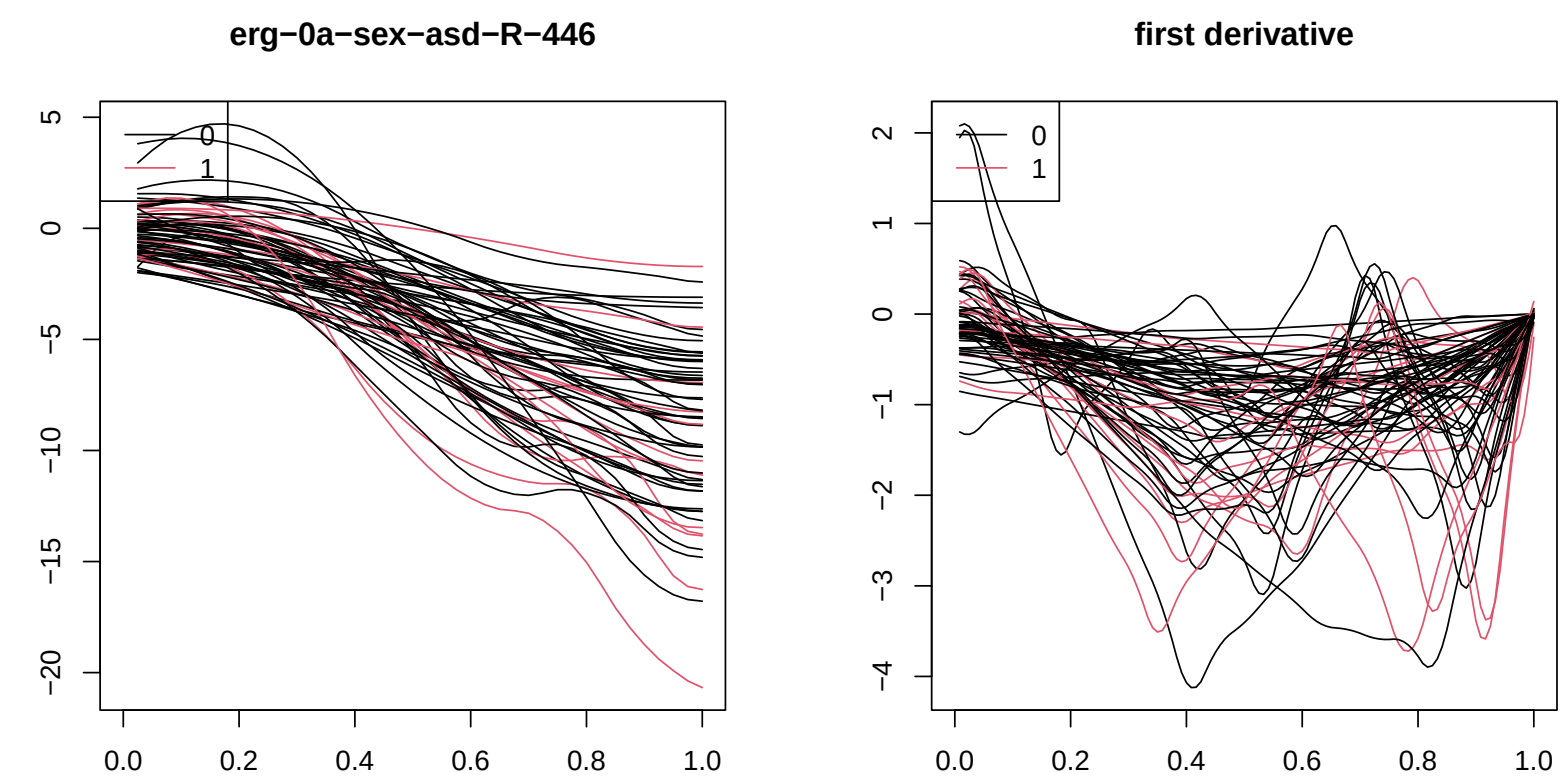
erg−0a−sex−asd−R−446
first derivative
0
1

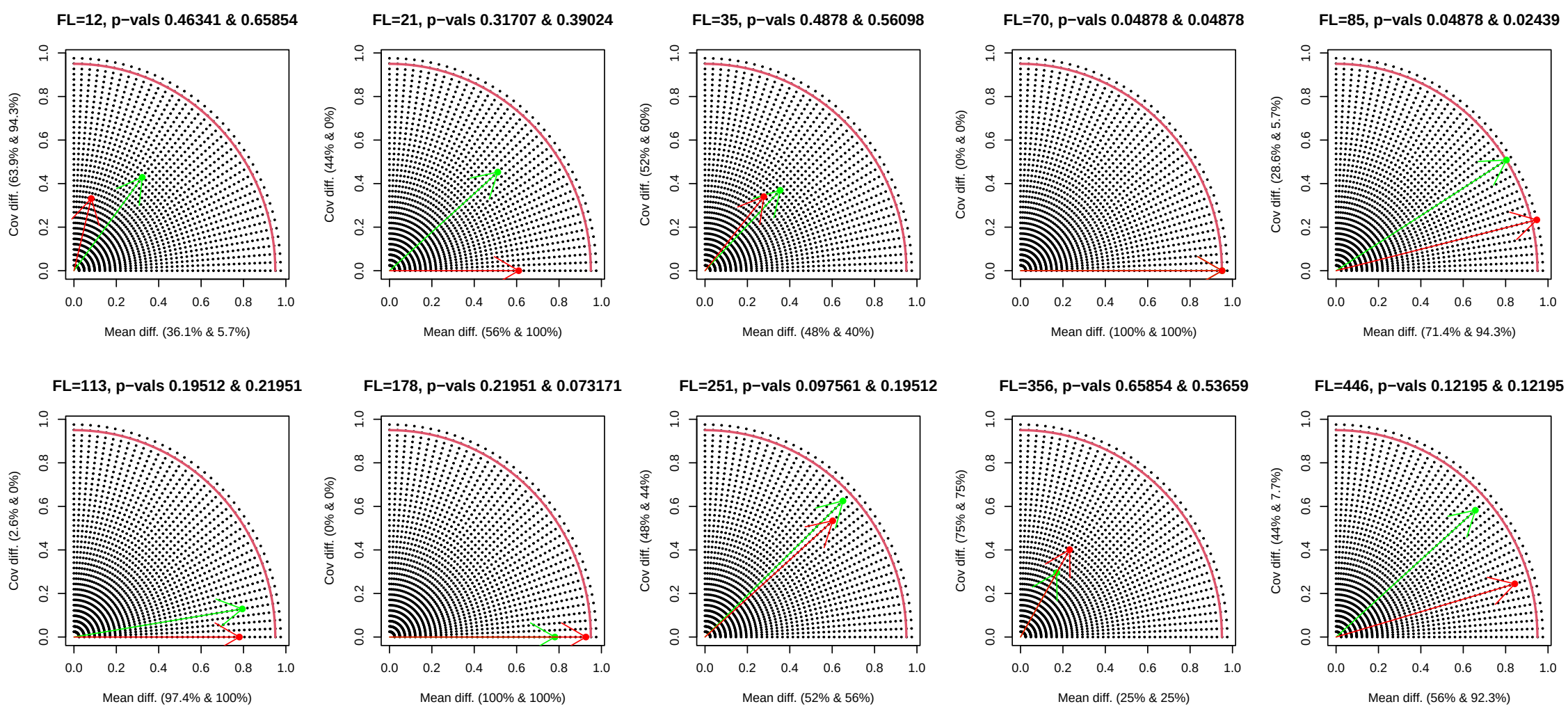
FL=12, p−vals 0.46341 & 0.65854
Cov diff. (63.9% & 94.3%)
Mean diff. (36.1% & 5.7%)
FL=21, p−vals 0.31707 & 0.39024
Cov diff. (44% & 0%)
Mean diff. (56% & 100%)
FL=35, p−vals 0.4878 & 0.56098
Cov diff. (52% & 60%)
Mean diff. (48% & 40%)
FL=70, p−vals 0.04878 & 0.04878
Cov diff. (0% & 0%)
Mean diff. (100% & 100%)
FL=85, p−vals 0.04878 & 0.02439
Cov diff. (28.6% & 5.7%)
Mean diff. (71.4% & 94.3%)
FL=113, p−vals 0.19512 & 0.21951
Cov diff. (2.6% & 0%)
Mean diff. (97.4% & 100%)
FL=178, p−vals 0.21951 & 0.073171
Cov diff. (0% & 0%)
Mean diff. (100% & 100%)
FL=251, p−vals 0.097561 & 0.19512
Cov diff. (48% & 44%)
Mean diff. (52% & 56%)
FL=356, p−vals 0.65854 & 0.53659
Cov diff. (75% & 75%)
Mean diff. (25% & 25%)
FL=446, p−vals 0.12195 & 0.12195
Cov diff. (44% & 7.7%)
Mean diff. (56% & 92.3%)

Table for ERG wave by sex in 0–atime – asd subgroup

	pval-erg-0a	mean perc	cov perc	pval-derivative	mean perc	cov perc
12	0.4634			0.6585		
21	0.3171			0.3902		
35	0.4878			0.5610		
70	0.0488	100	0	0.0488	100	0
85	0.0488	71.4	28.6	0.0244	94.3	5.7
113	0.1951			0.2195		
178	0.2195			0.0732		
251	0.0976			0.1951		
356	0.6585			0.5366		
446	0.1220			0.1220		

2.9 ERG wave by sex in atime–btime – ctr subgroup

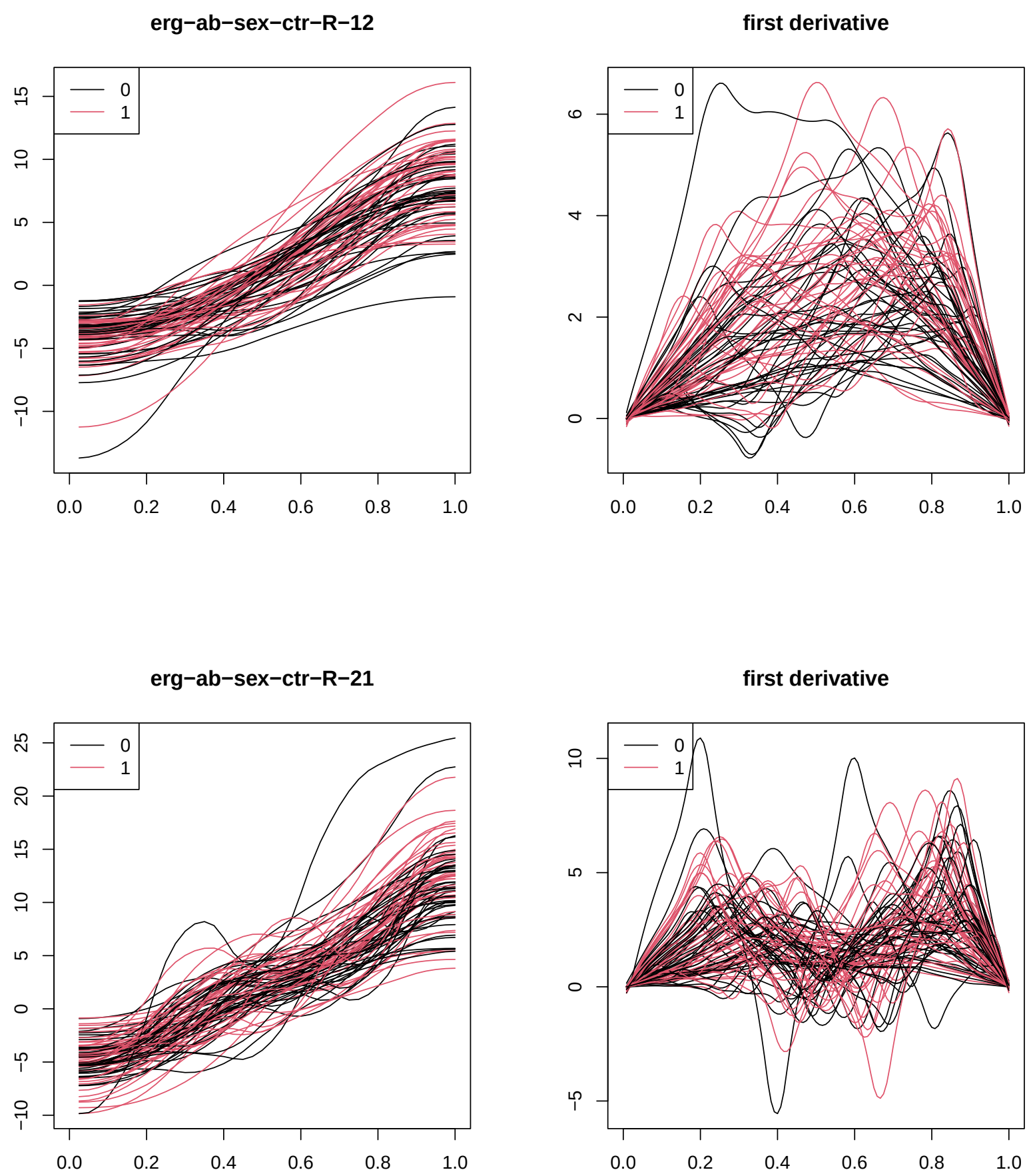

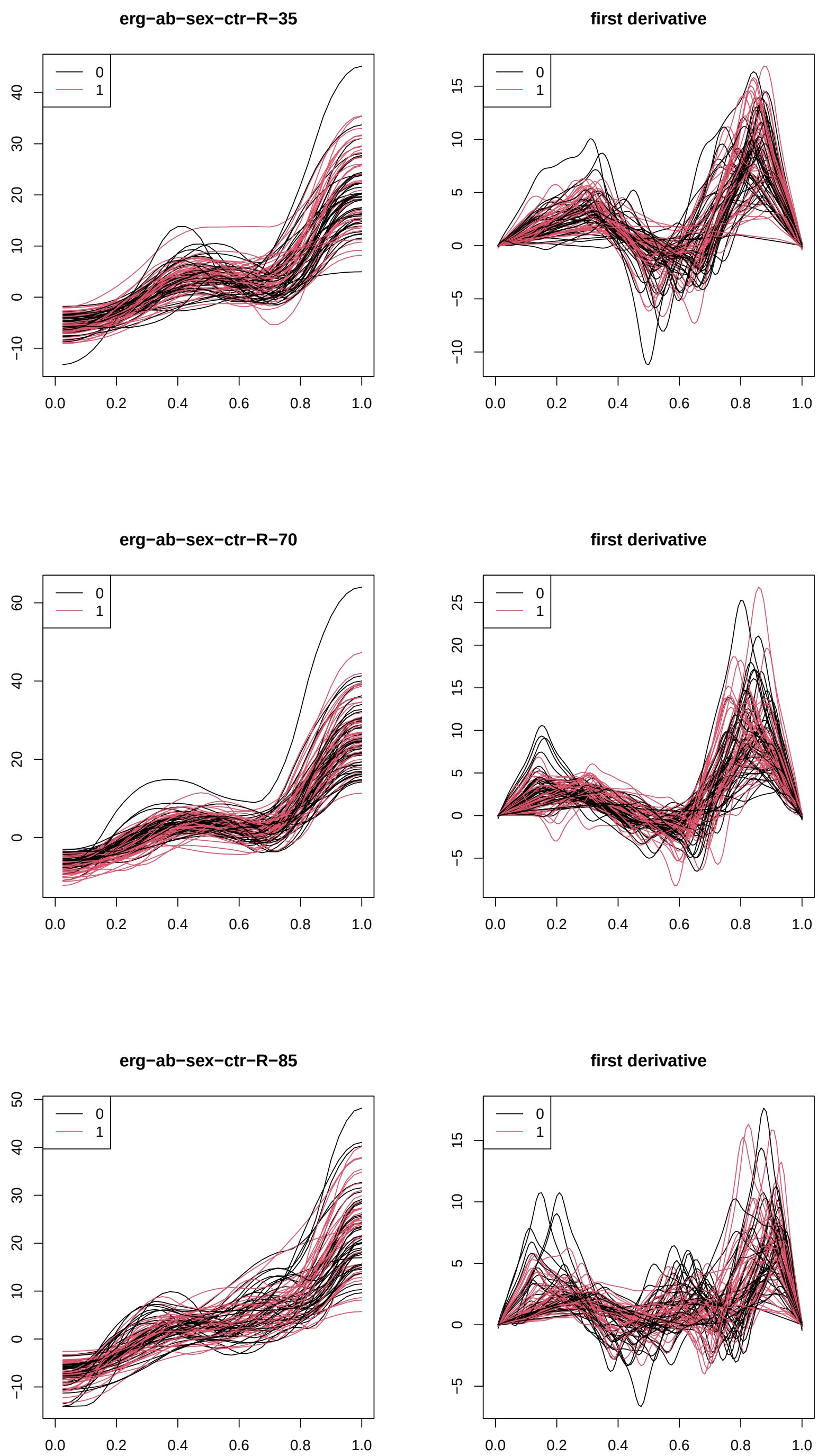
erg−ab−sex−ctr−R−35
first derivative
erg−ab−sex−ctr−R−70
first derivative
erg−ab−sex−ctr−R−85
first derivative
0
1

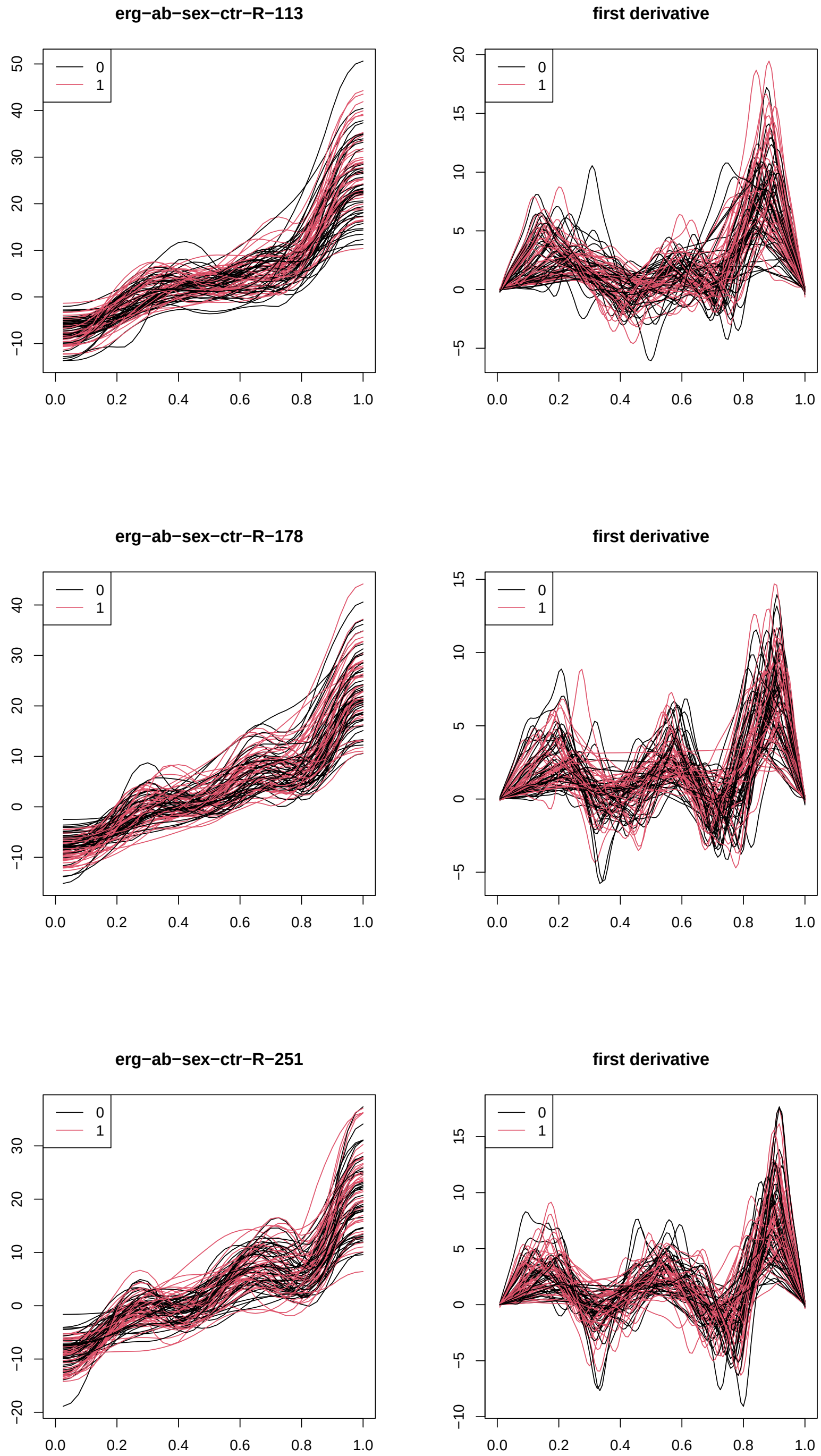
erg−ab−sex−ctr−R−113
first derivative
erg−ab−sex−ctr−R−178
first derivative
erg−ab−sex−ctr−R−251
first derivative
0
1
0.0
0.2
0.4
0.6
0.8
1.0

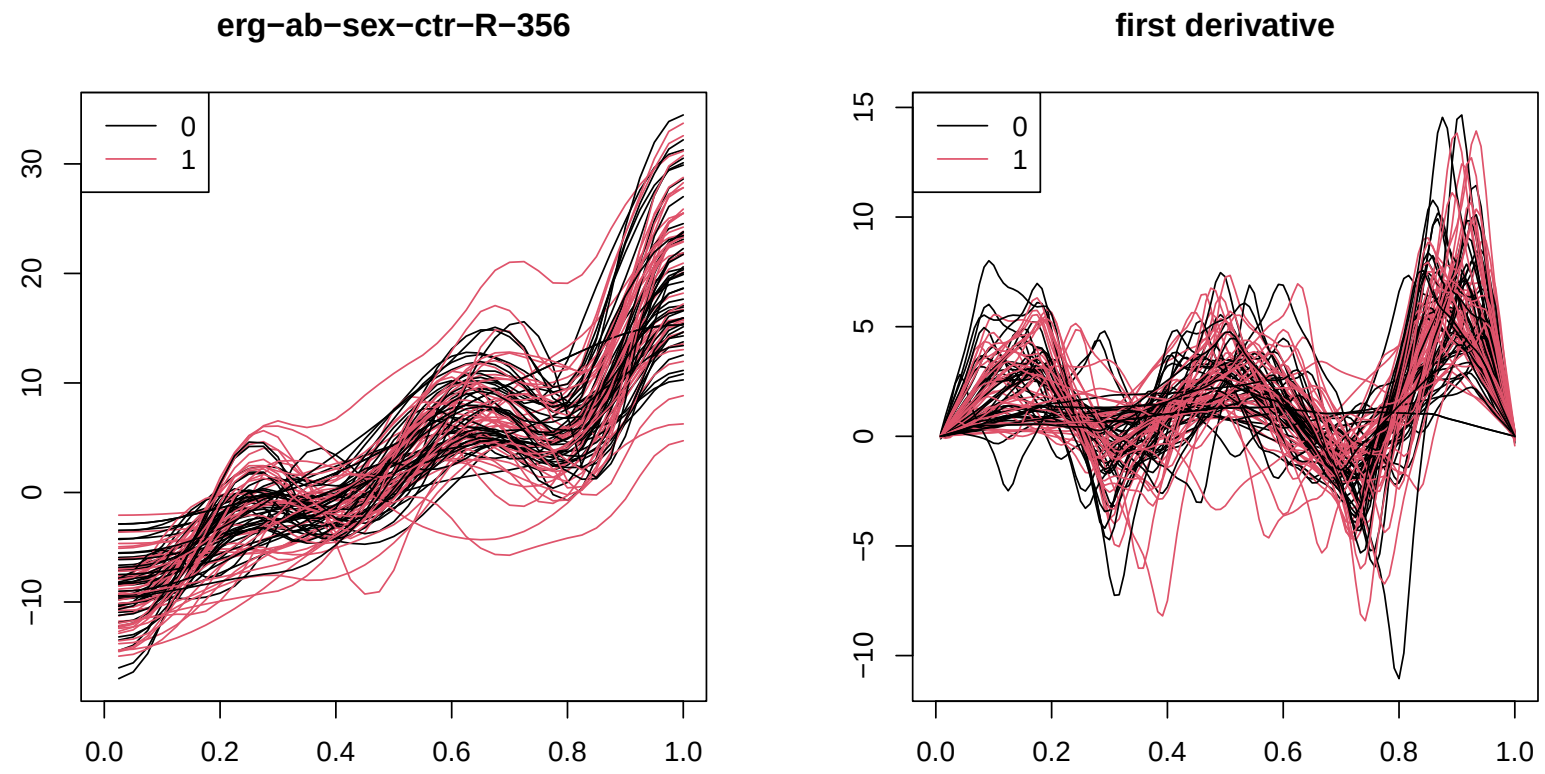
erg−ab−sex−ctr−R−356
first derivative
0
1

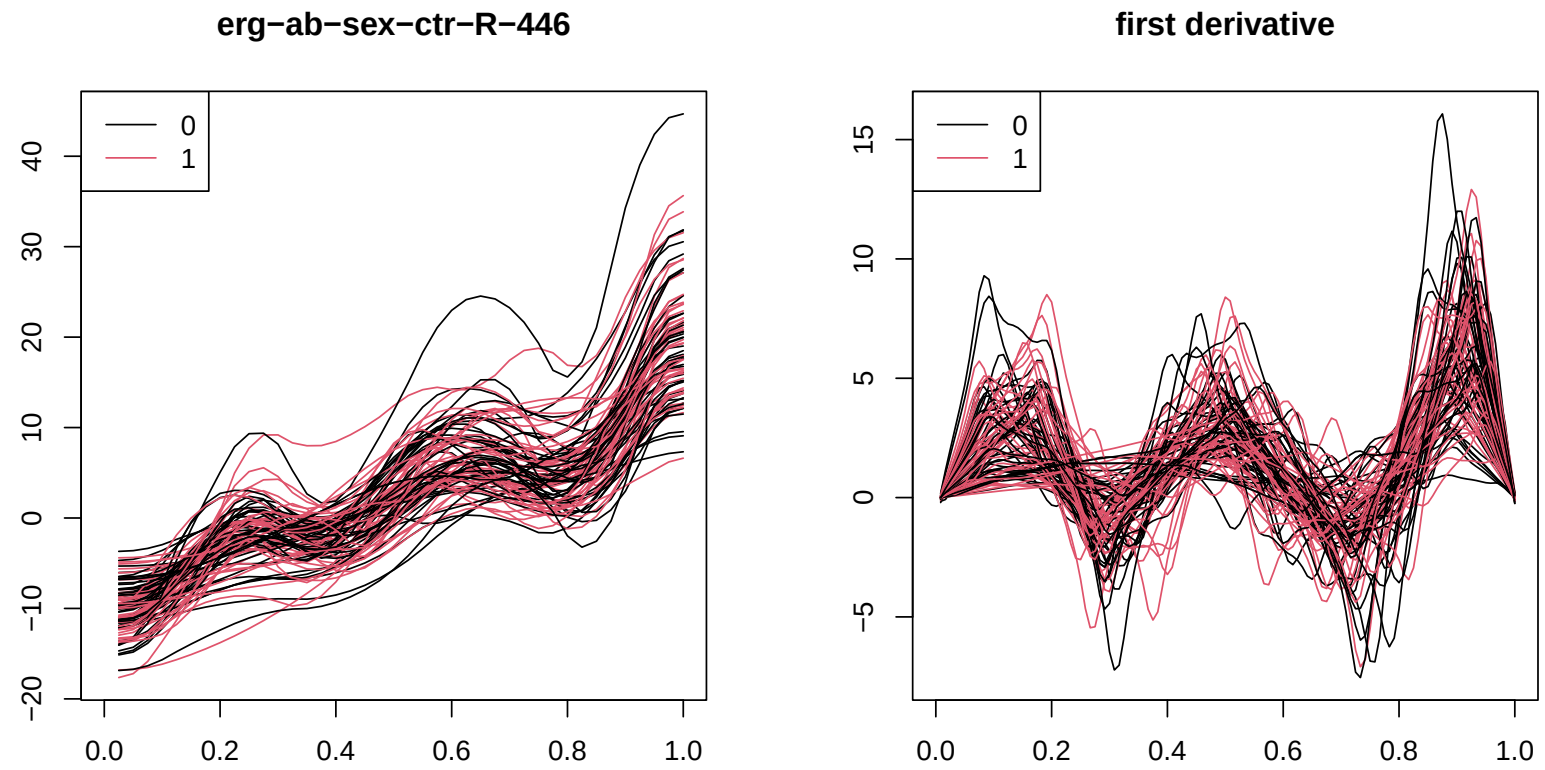
erg−ab−sex−ctr−R−446
first derivative
0
1

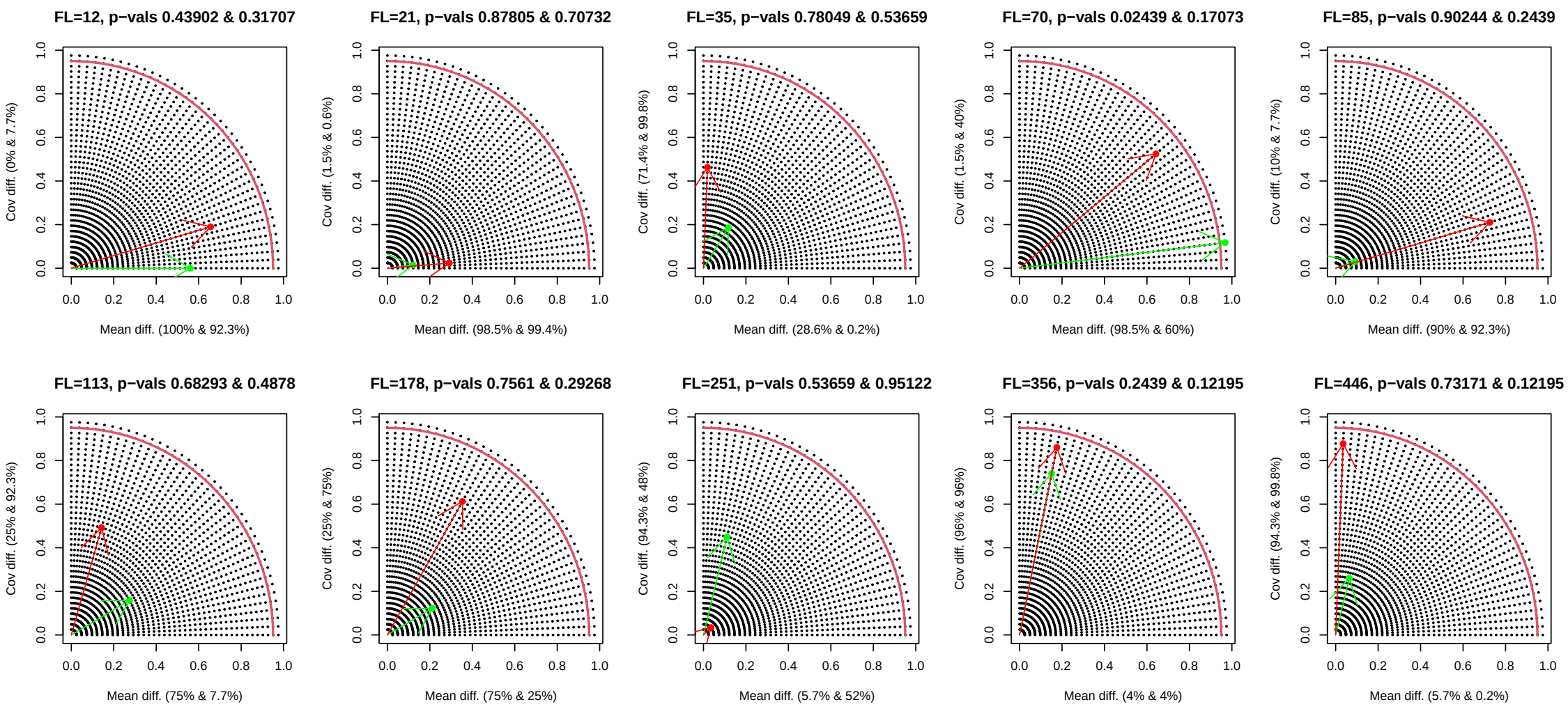
FL=12, p−vals 0.43902 & 0.31707
Cov diff. (0% & 7.7%)
Mean diff. (100% & 92.3%)
FL=21, p−vals 0.87805 & 0.70732
Cov diff. (1.5% & 0.6%)
Mean diff. (98.5% & 99.4%)
FL=35, p−vals 0.78049 & 0.53659
Cov diff. (71.4% & 99.8%)
Mean diff. (28.6% & 0.2%)
FL=70, p−vals 0.02439 & 0.17073
Cov diff. (1.5% & 40%)
Mean diff. (98.5% & 60%)
FL=85, p−vals 0.90244 & 0.2439
Cov diff. (10% & 7.7%)
Mean diff. (90% & 92.3%)
FL=113, p−vals 0.68293 & 0.4878
Cov diff. (25% & 92.3%)
Mean diff. (75% & 7.7%)
FL=178, p−vals 0.7561 & 0.29268
Cov diff. (25% & 75%)
Mean diff. (75% & 25%)
FL=251, p−vals 0.53659 & 0.95122
Cov diff. (94.3% & 48%)
Mean diff. (5.7% & 52%)
FL=356, p−vals 0.2439 & 0.12195
Cov diff. (96% & 96%)
Mean diff. (4% & 4%)
FL=446, p−vals 0.73171 & 0.12195
Cov diff. (94.3% & 99.8%)
Mean diff. (5.7% & 0.2%)

Table for ERG wave by sex in atime–btime – ctr subgroup

	pval-erg-ab	mean perc	cov perc	pval-derivative	mean perc	cov perc
12	0.4390			0.3171		
21	0.8780			0.7073		
35	0.7805			0.5366		
70	0.0244	98.5	1.5	0.1707		
85	0.9024			0.2439		
113	0.6829			0.4878		
178	0.7561			0.2927		
251	0.5366			0.9512		
356	0.2439			0.1220		
446	0.7317			0.1220		

2.10 ERG wave by sex in atime–btime – asd subgroup

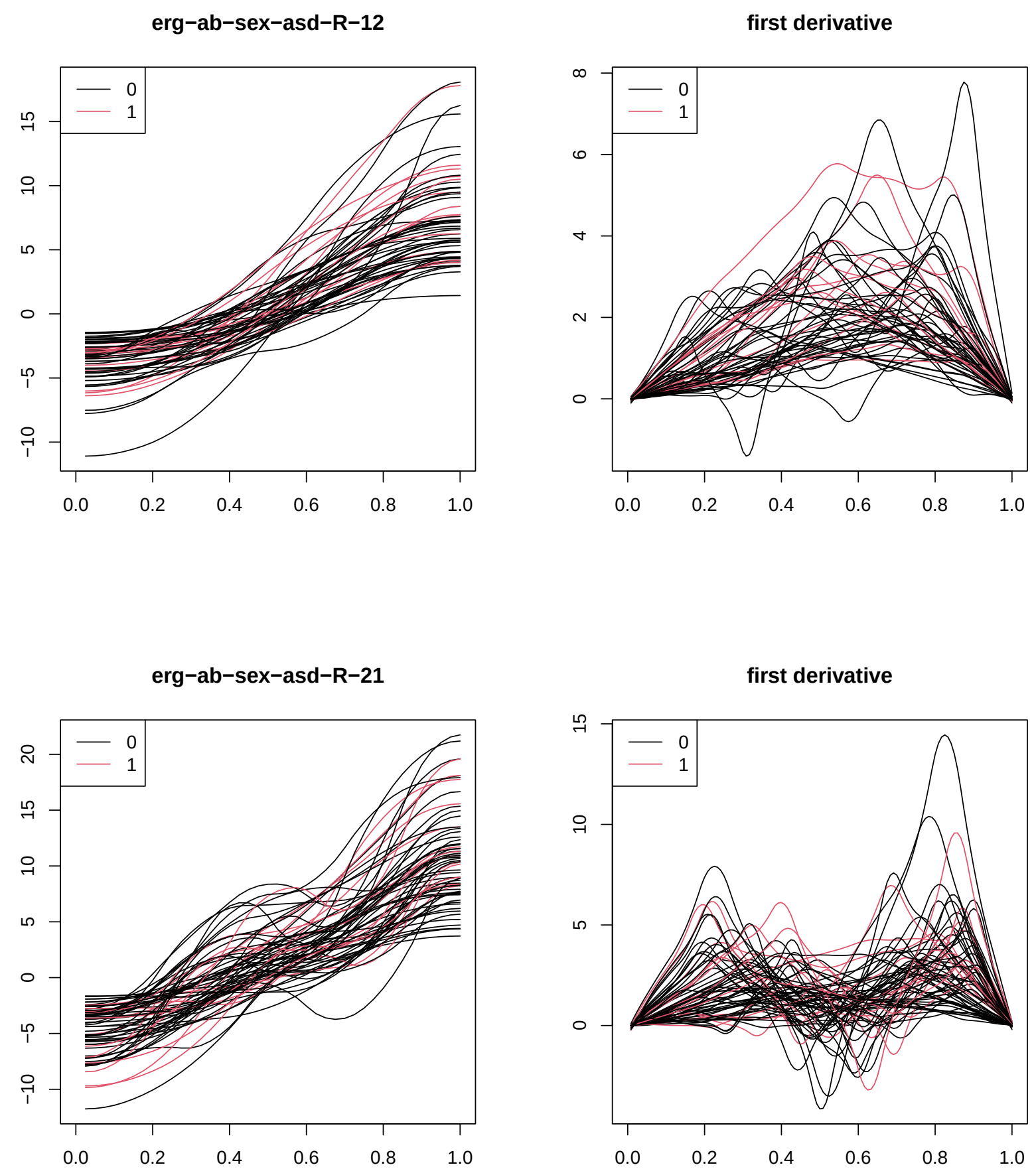

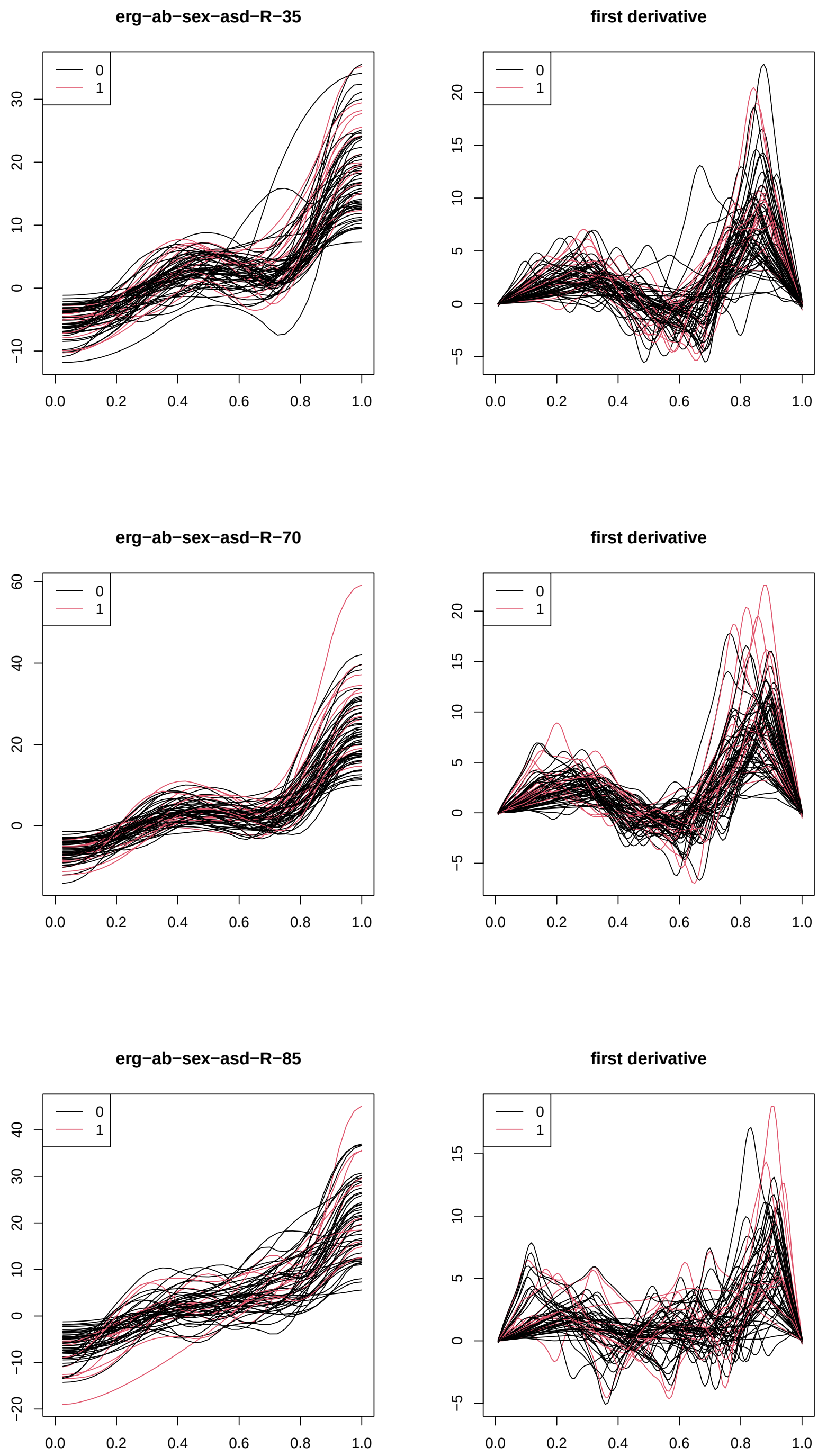
erg−ab−sex−asd−R−35
first derivative
erg−ab−sex−asd−R−70
first derivative
erg−ab−sex−asd−R−85
first derivative
0
1
0.0 0.2 0.4 0.6 0.8 1.0

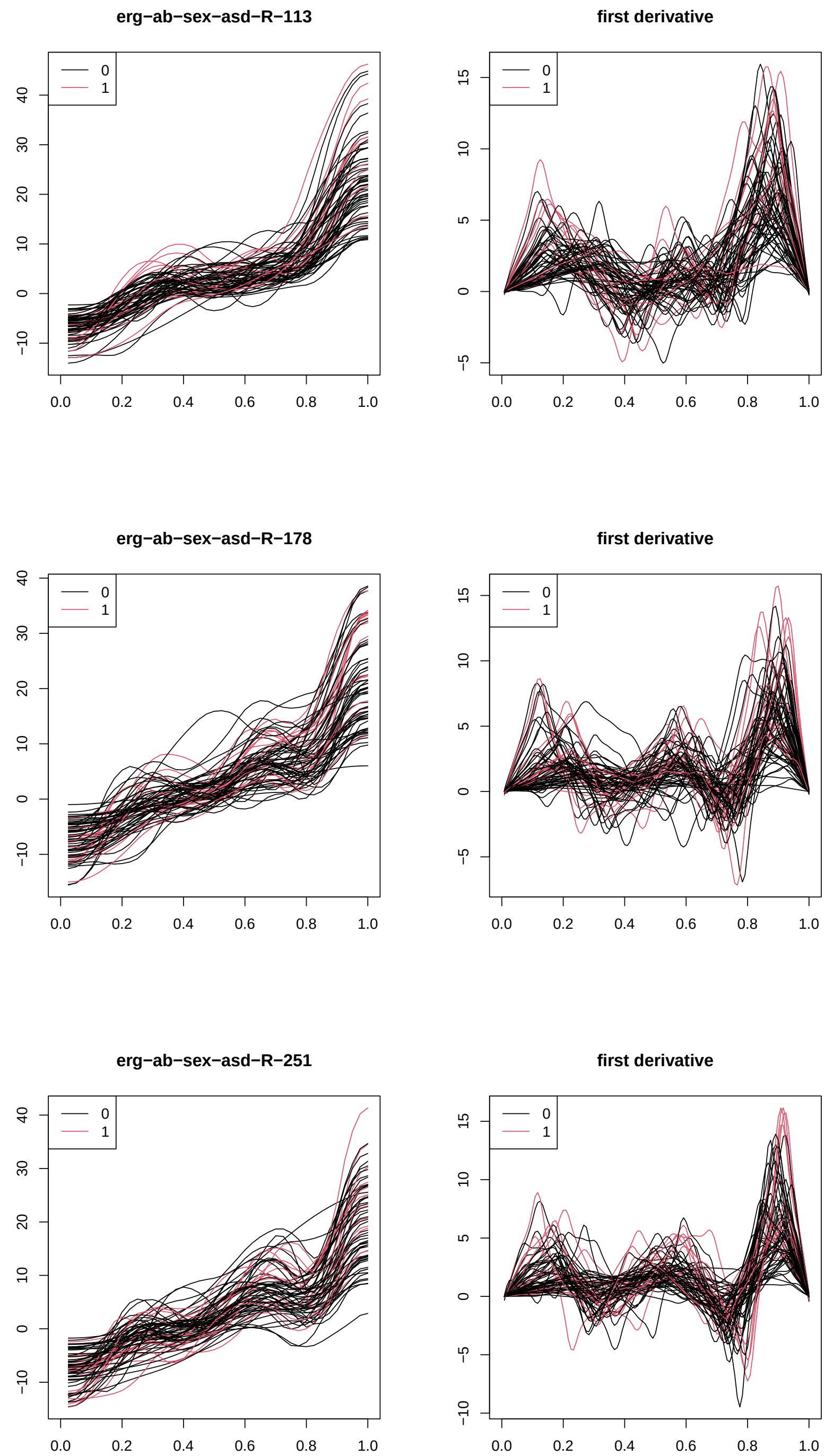
erg−ab−sex−asd−R−113
first derivative
erg−ab−sex−asd−R−178
first derivative
erg−ab−sex−asd−R−251
first derivative
0
1

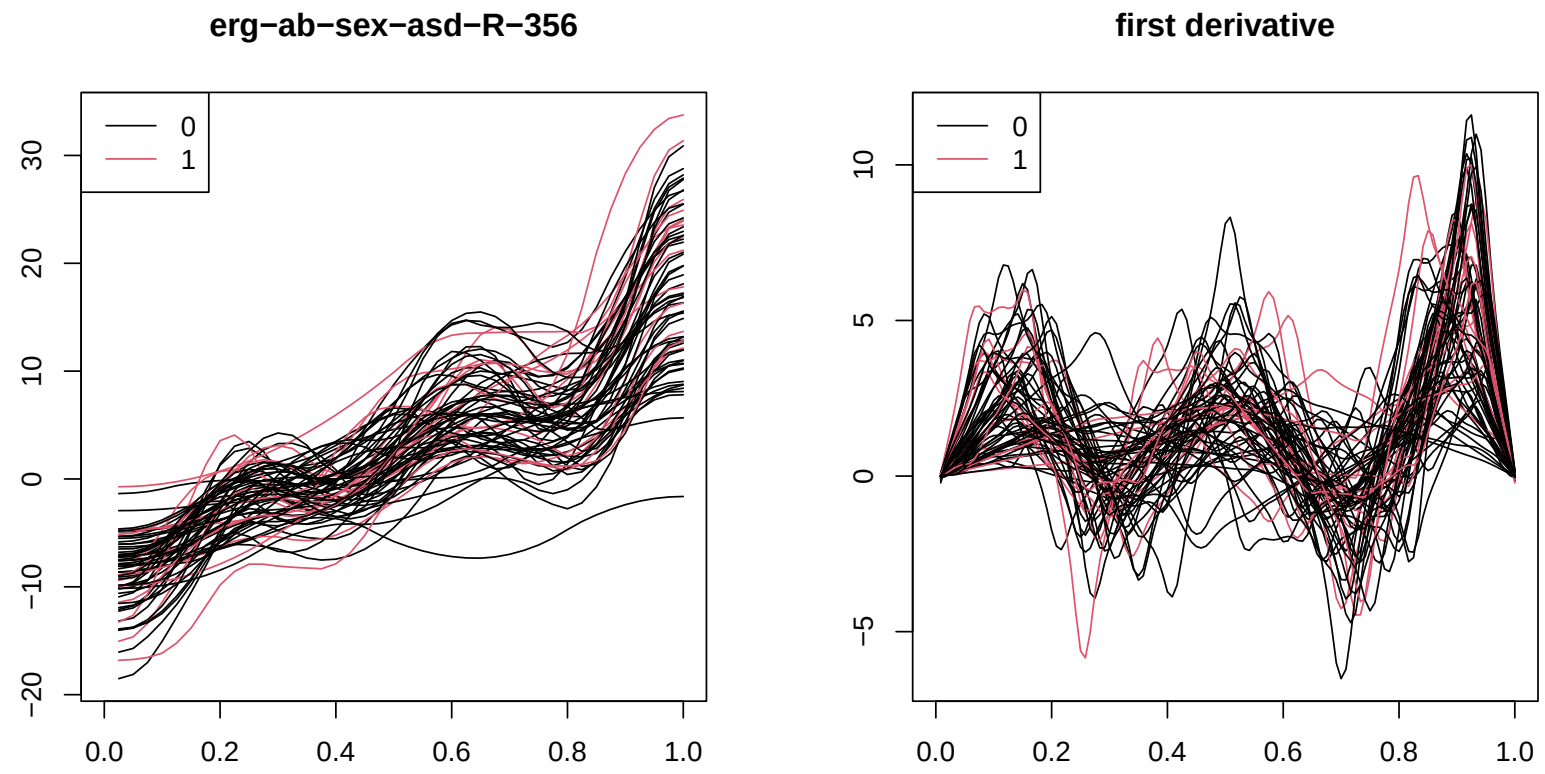
erg−ab−sex−asd−R−356
first derivative
0
1

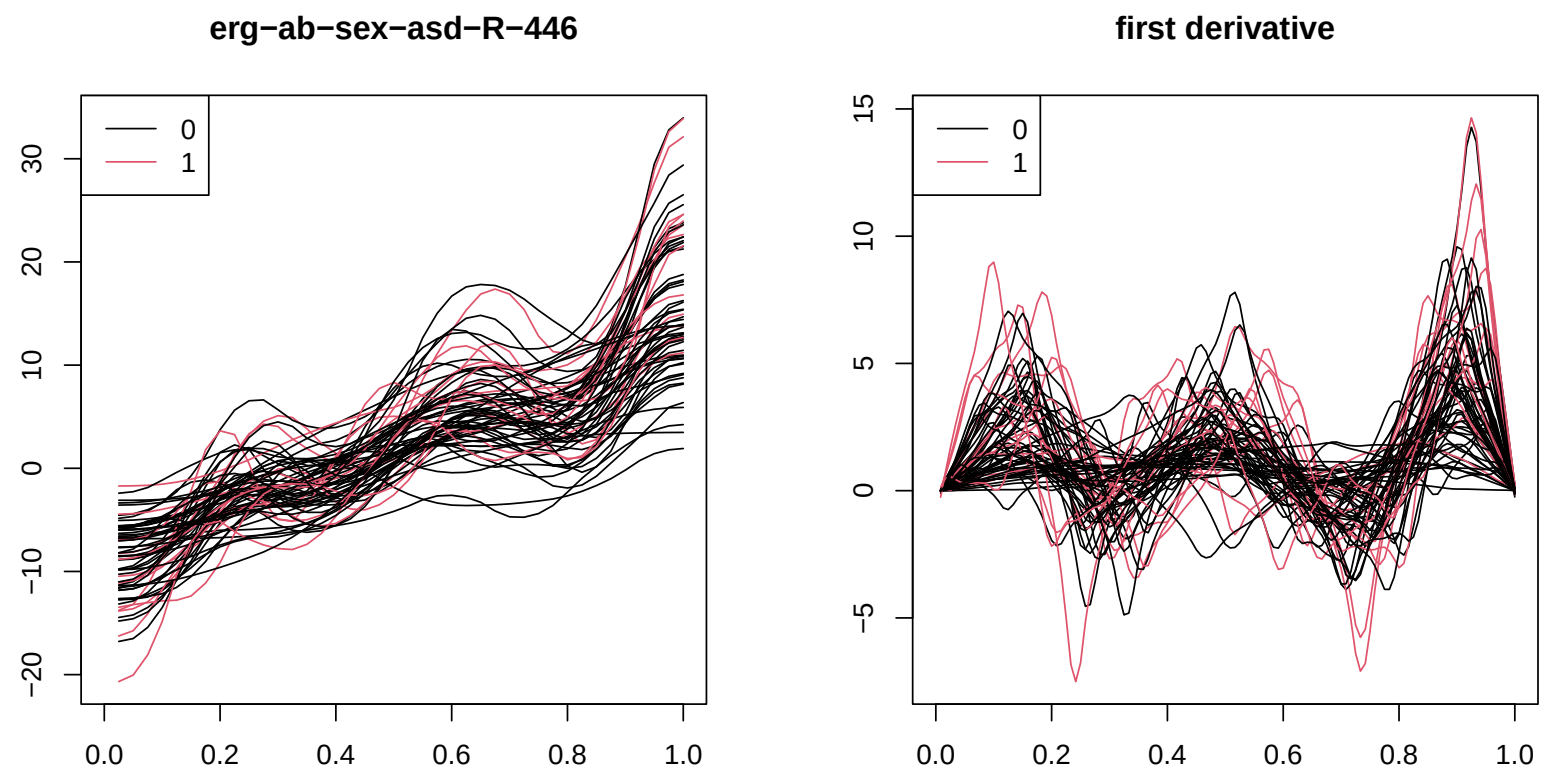
erg−ab−sex−asd−R−446
first derivative
0
1

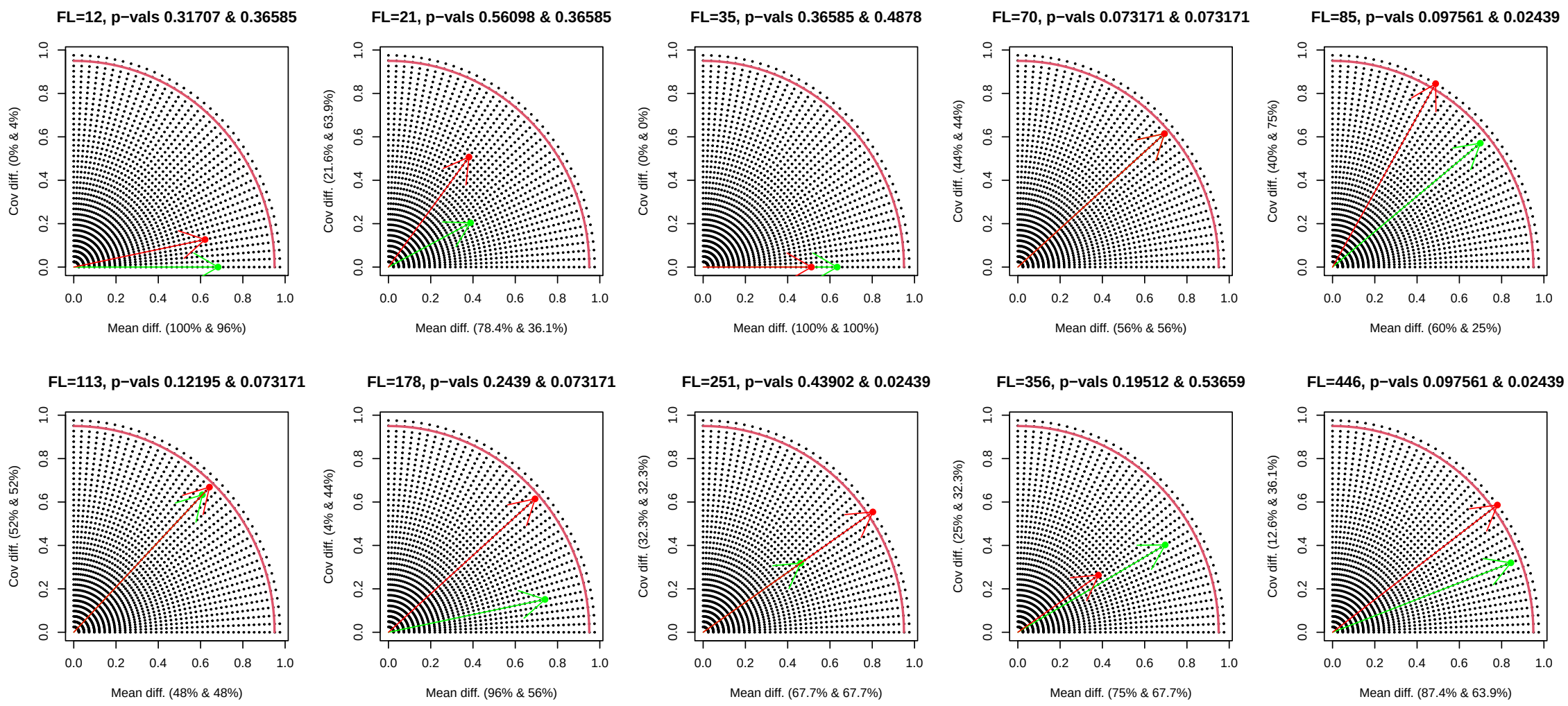
FL=12, p−vals 0.31707 & 0.36585
Cov diff. (0% & 4%)
Mean diff. (100% & 96%)
FL=21, p−vals 0.56098 & 0.36585
Cov diff. (21.6% & 63.9%)
Mean diff. (78.4% & 36.1%)
FL=35, p−vals 0.36585 & 0.4878
Cov diff. (0% & 0%)
Mean diff. (100% & 100%)
FL=70, p−vals 0.073171 & 0.073171
Cov diff. (44% & 44%)
Mean diff. (56% & 56%)
FL=85, p−vals 0.097561 & 0.02439
Cov diff. (40% & 75%)
Mean diff. (60% & 25%)
FL=113, p−vals 0.12195 & 0.073171
Cov diff. (52% & 52%)
Mean diff. (48% & 48%)
FL=178, p−vals 0.2439 & 0.073171
Cov diff. (4% & 44%)
Mean diff. (96% & 56%)
FL=251, p−vals 0.43902 & 0.02439
Cov diff. (32.3% & 32.3%)
Mean diff. (67.7% & 67.7%)
FL=356, p−vals 0.19512 & 0.53659
Cov diff. (25% & 32.3%)
Mean diff. (75% & 67.7%)
FL=446, p−vals 0.097561 & 0.02439
Cov diff. (12.6% & 36.1%)
Mean diff. (87.4% & 63.9%)

Table for ERG wave by sex in atime–btime – asd subgroup

	pval-erg-ab	mean perc	cov perc	pval-derivative	mean perc	cov perc
12	0.3171			0.3659		
21	0.5610			0.3659		
35	0.3659			0.4878		
70	0.0732			0.0732		
85	0.0976			0.0244	25	75
113	0.1220			0.0732		
178	0.2439			0.0732		
251	0.4390			0.0244	67.7	32.3
356	0.1951			0.5366		
446	0.0976			0.0244	63.9	36.1

2.11 ERG wave by sex in btime–72 – ctr subgroup

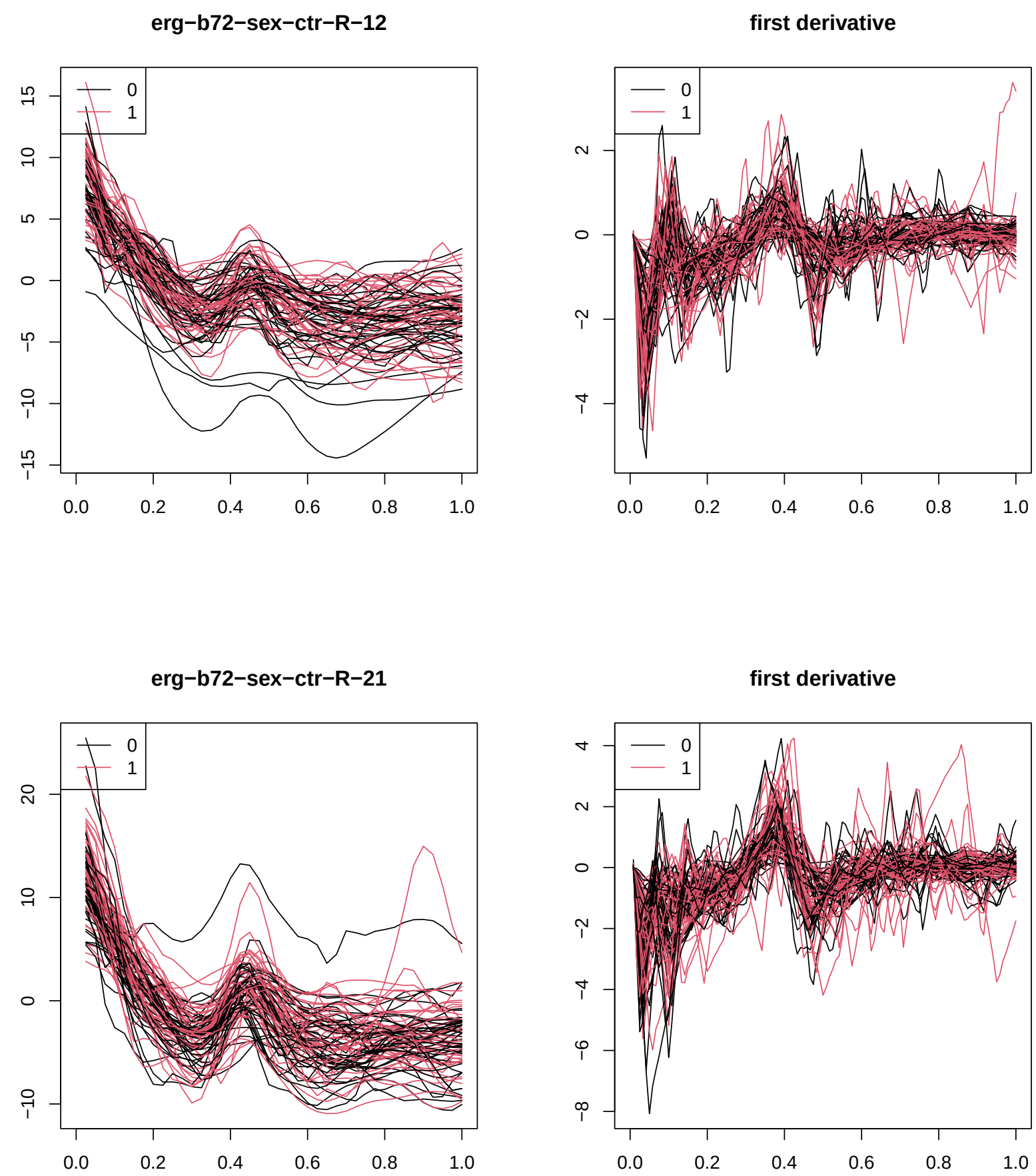

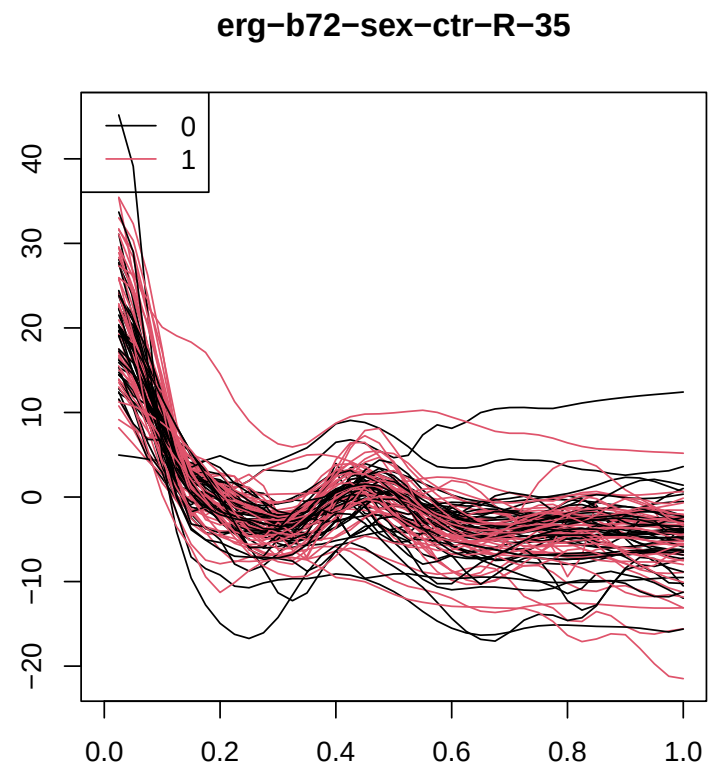
erg−b72−sex−ctr−R−35
0
1
40
30
20
10
0
−10
−20
0.0
0.2
0.4
0.6
0.8
1.0

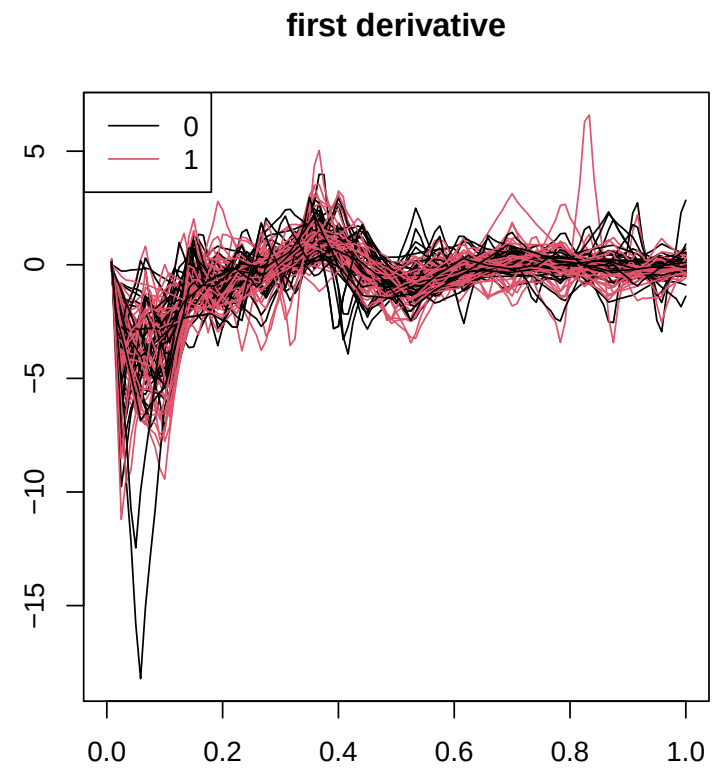
first derivative
0
1
5
0
−5
−10
−15
0.0
0.2
0.4
0.6
0.8
1.0

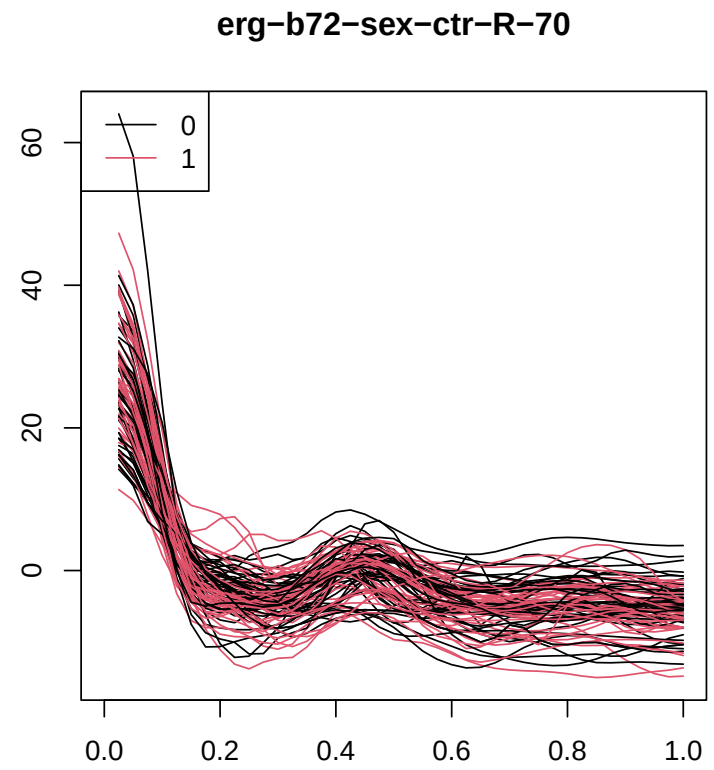
erg−b72−sex−ctr−R−70
0
1
60
40
20
0
0.0
0.2
0.4
0.6
0.8
1.0

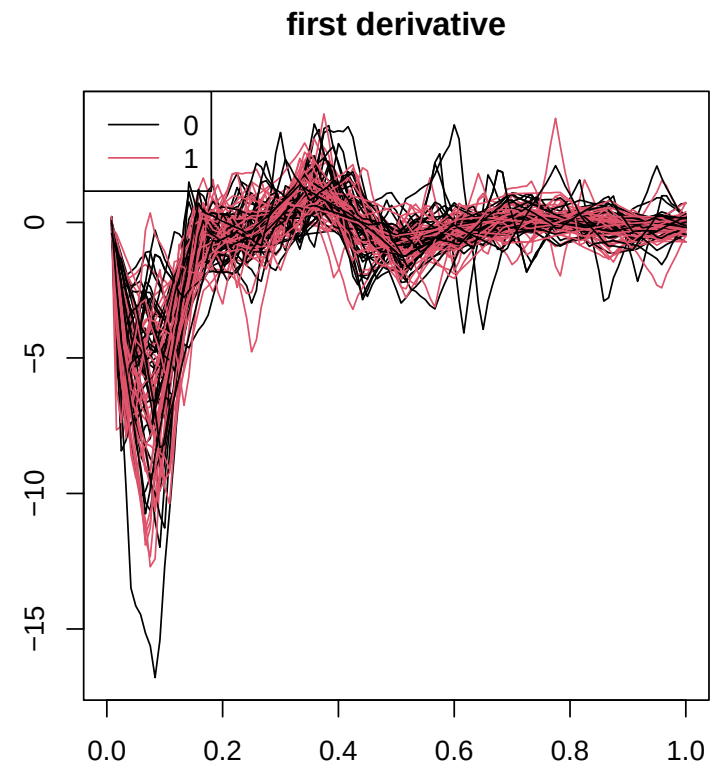
first derivative
0
1
0
−5
−10
−15
0.0
0.2
0.4
0.6
0.8
1.0

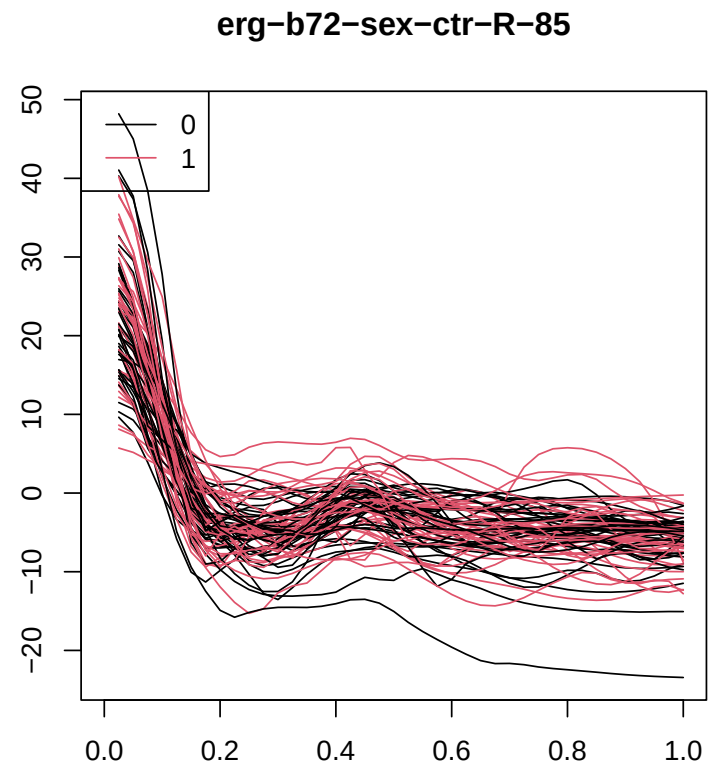
erg−b72−sex−ctr−R−85
0
1
50
40
30
20
10
0
−10
−20
0.0
0.2
0.4
0.6
0.8
1.0

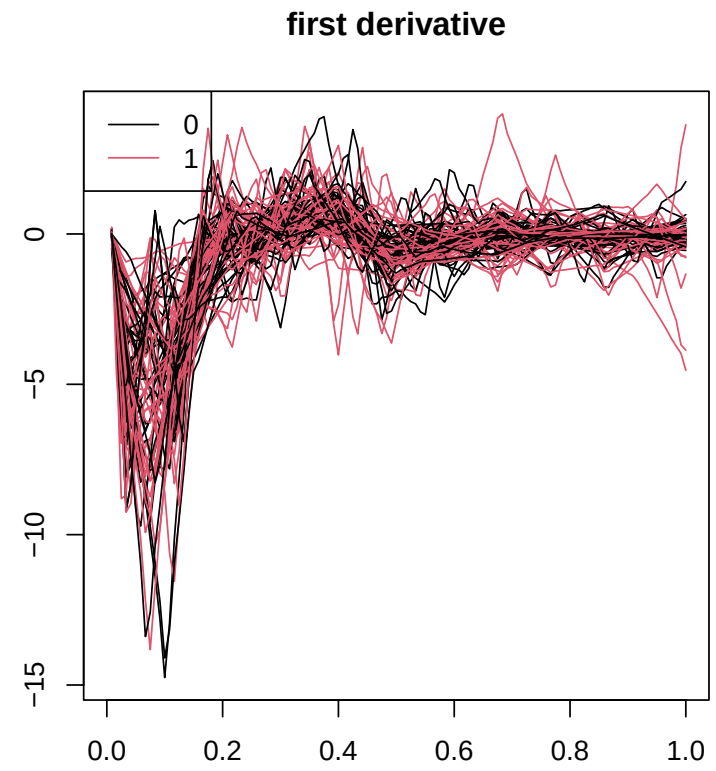
first derivative
0
1
0
−5
−10
−15
0.0
0.2
0.4
0.6
0.8
1.0

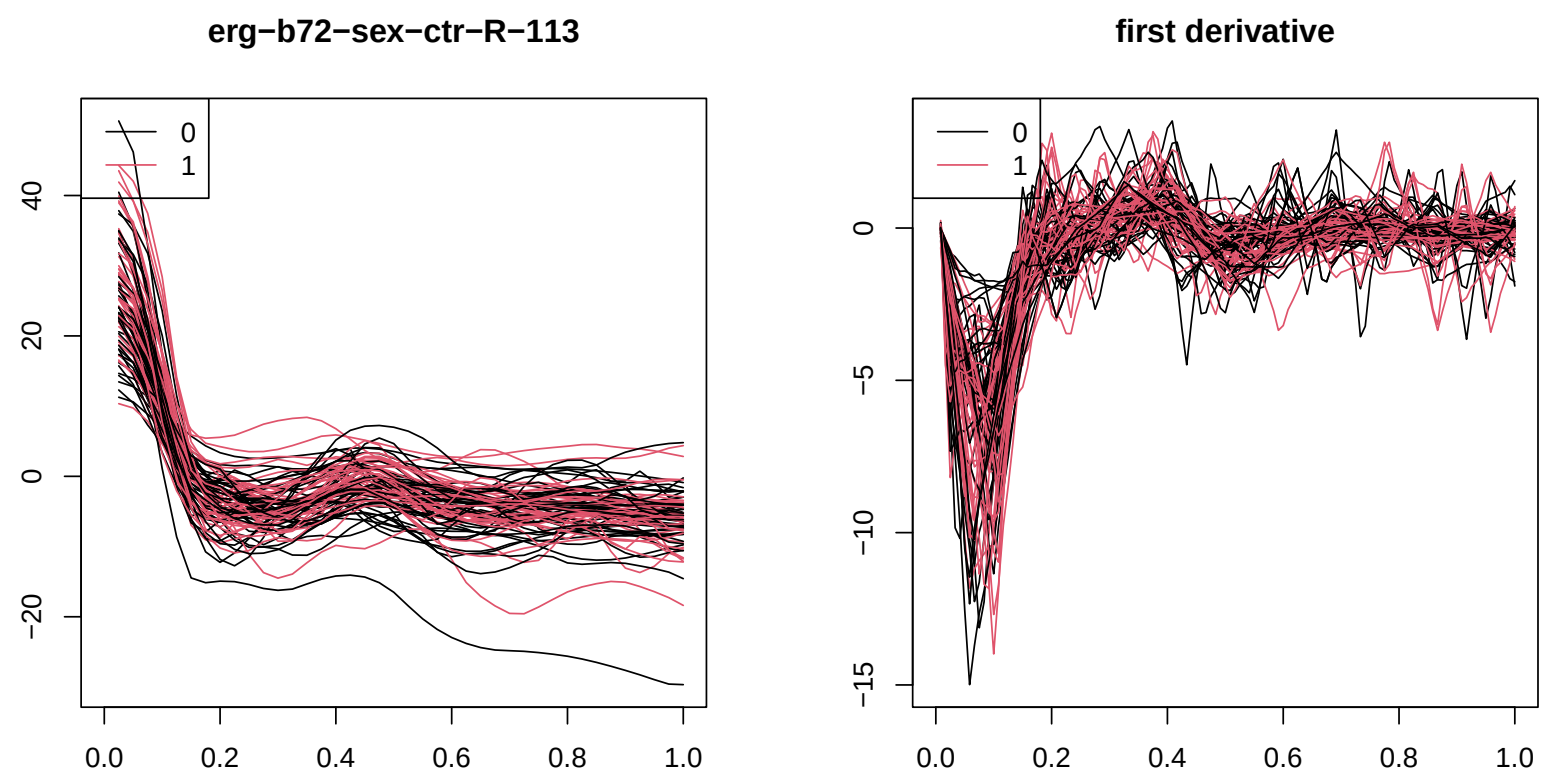
erg−b72−sex−ctr−R−113
first derivative
0
1

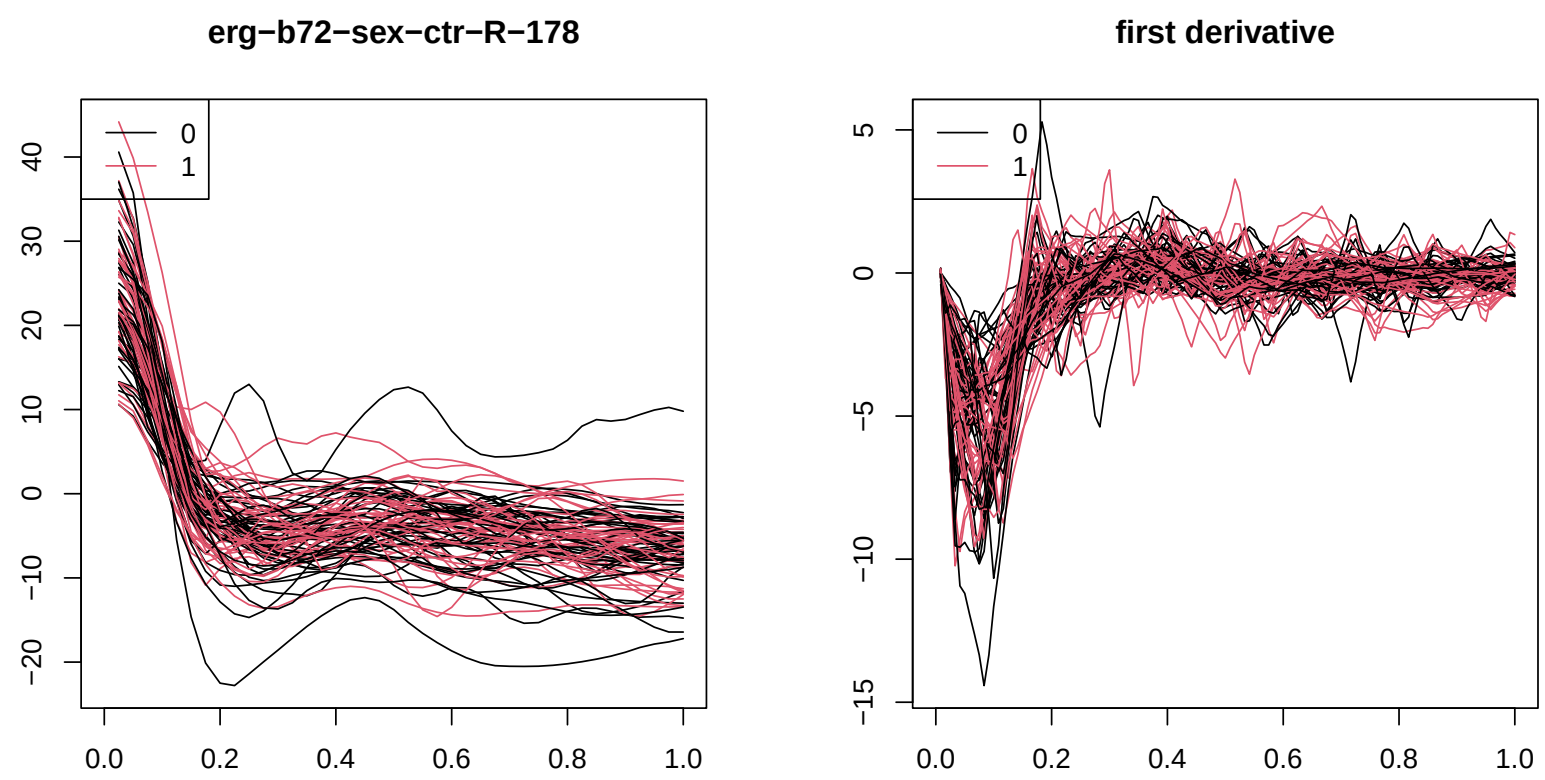
erg−b72−sex−ctr−R−178
first derivative
0
1

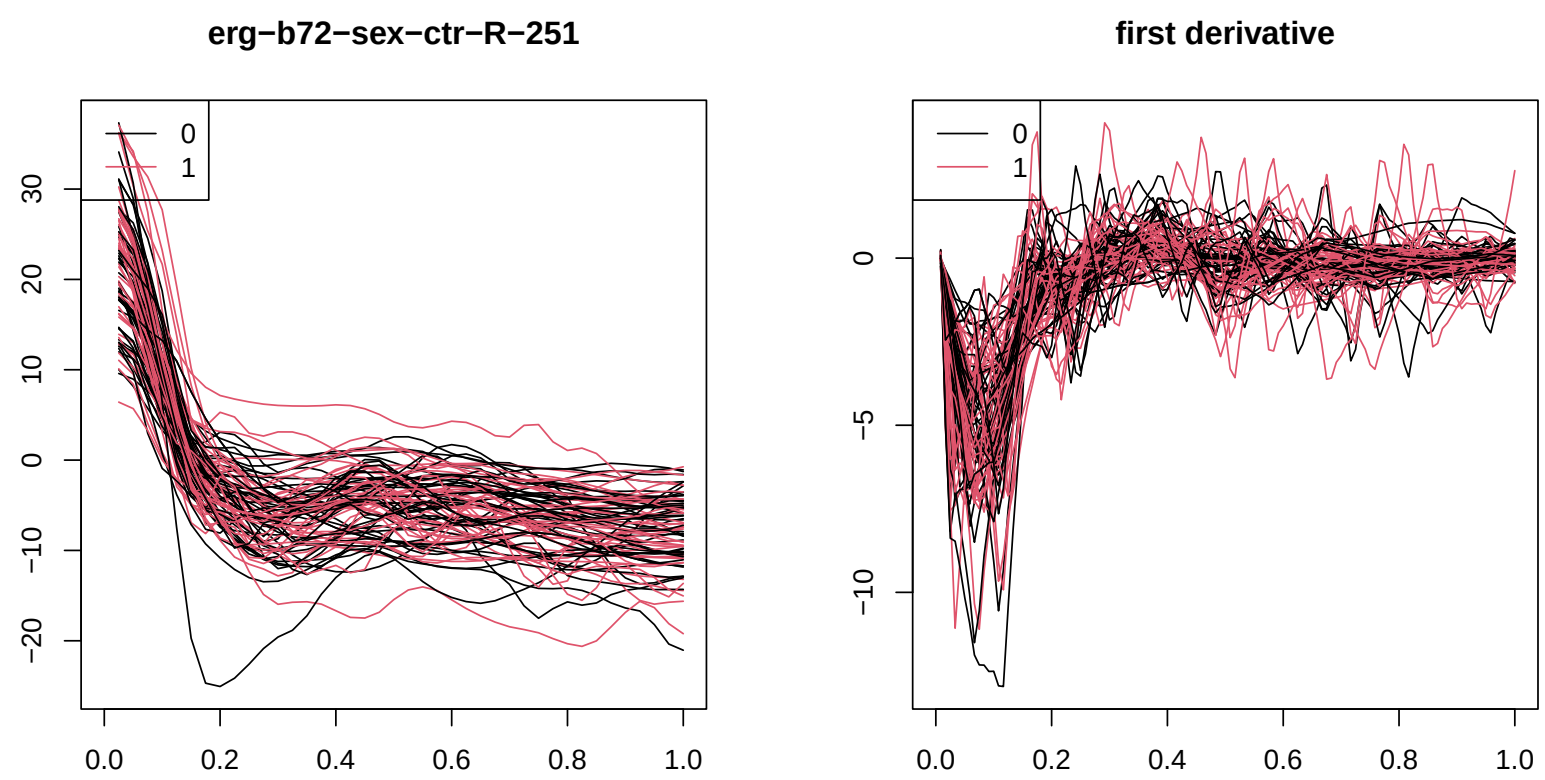
erg−b72−sex−ctr−R−251
first derivative
0
1

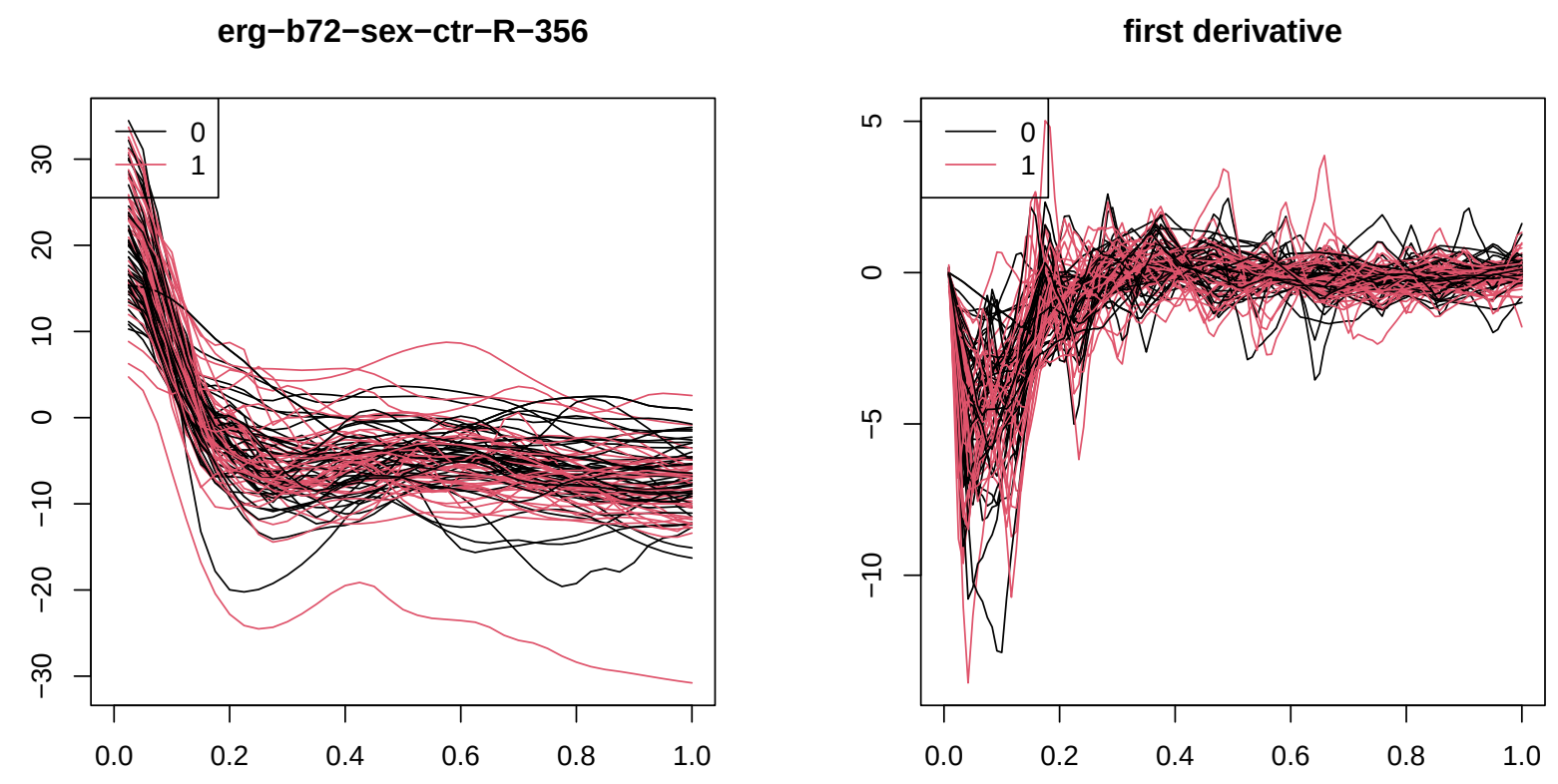
erg−b72−sex−ctr−R−356
first derivative
0
1

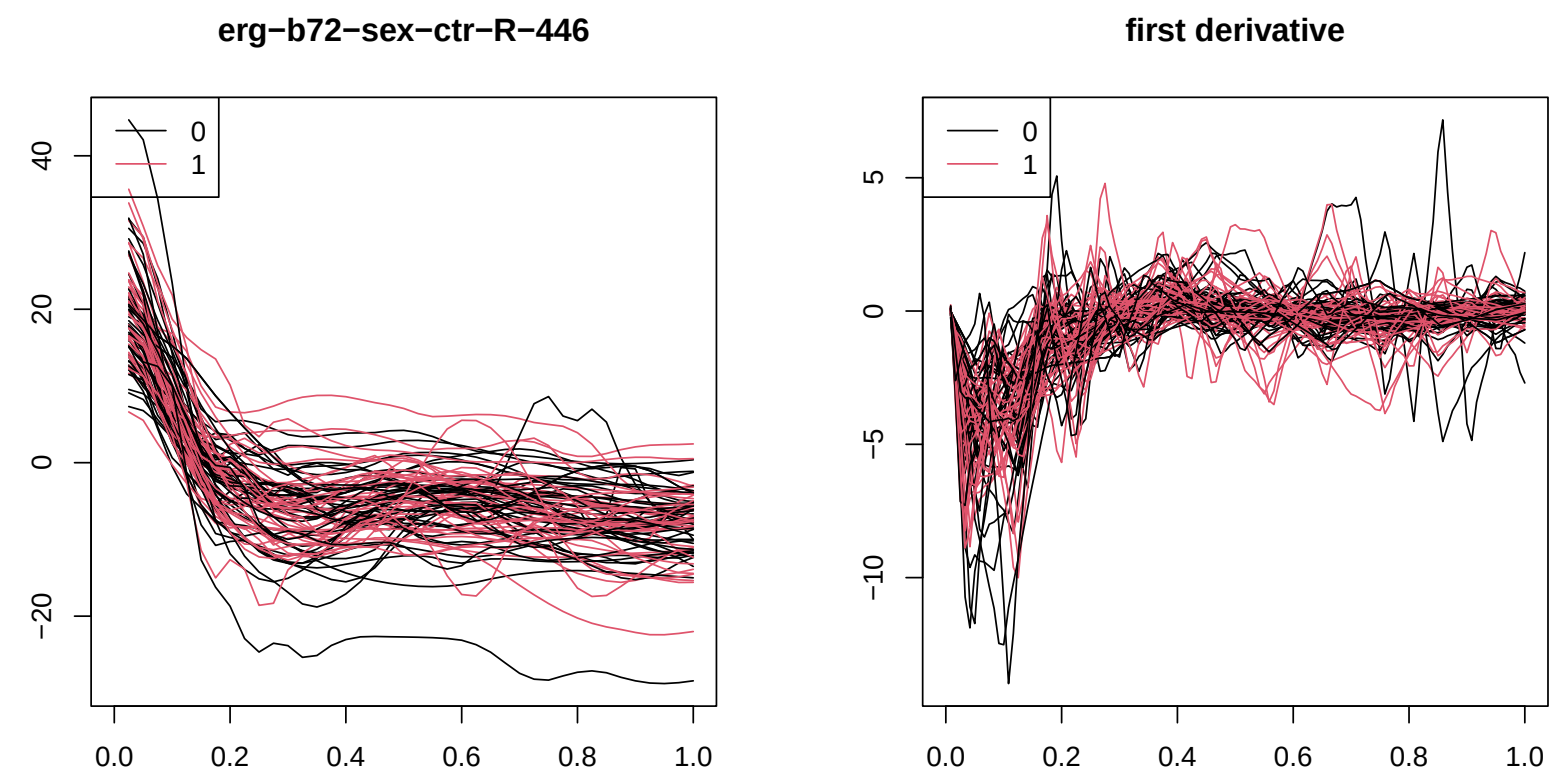
erg−b72−sex−ctr−R−446
first derivative
0
1

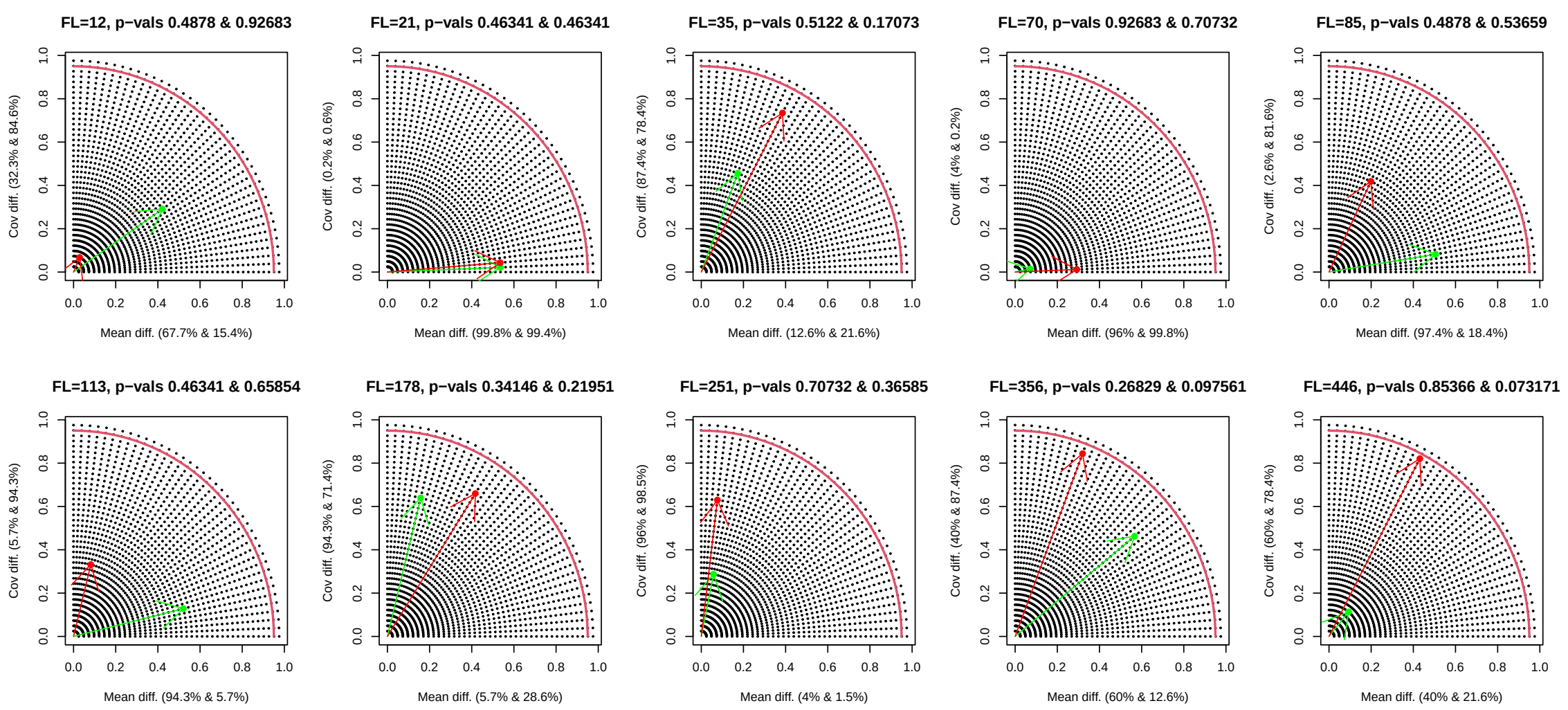
FL=12, p−vals 0.4878 & 0.92683
Mean diff. (67.7% & 15.4%)
Cov diff. (32.3% & 84.6%)
FL=21, p−vals 0.46341 & 0.46341
Mean diff. (99.8% & 99.4%)
Cov diff. (0.2% & 0.6%)
FL=35, p−vals 0.5122 & 0.17073
Mean diff. (12.6% & 21.6%)
Cov diff. (87.4% & 78.4%)
FL=70, p−vals 0.92683 & 0.70732
Mean diff. (96% & 99.8%)
Cov diff. (4% & 0.2%)
FL=85, p−vals 0.4878 & 0.53659
Mean diff. (97.4% & 18.4%)
Cov diff. (2.6% & 81.6%)
FL=113, p−vals 0.46341 & 0.65854
Mean diff. (94.3% & 5.7%)
Cov diff. (5.7% & 94.3%)
FL=178, p−vals 0.34146 & 0.21951
Mean diff. (5.7% & 28.6%)
Cov diff. (94.3% & 71.4%)
FL=251, p−vals 0.70732 & 0.36585
Mean diff. (4% & 1.5%)
Cov diff. (96% & 98.5%)
FL=356, p−vals 0.26829 & 0.097561
Mean diff. (60% & 12.6%)
Cov diff. (40% & 87.4%)
FL=446, p−vals 0.85366 & 0.073171
Mean diff. (40% & 21.6%)
Cov diff. (60% & 78.4%)

Table for ERG wave by sex in btime–72 – ctr subgroup

	pval-erg-b72	mean perc	cov perc	pval-derivative	mean perc	cov perc
12	0.4878			0.9268		
21	0.4634			0.4634		
35	0.5122			0.1707		
70	0.9268			0.7073		
85	0.4878			0.5366		
113	0.4634			0.6585		
178	0.3415			0.2195		
251	0.7073			0.3659		
356	0.2683			0.0976		
446	0.8537			0.0732		

2.12 ERG wave by sex in btime–72 – asd subgroup

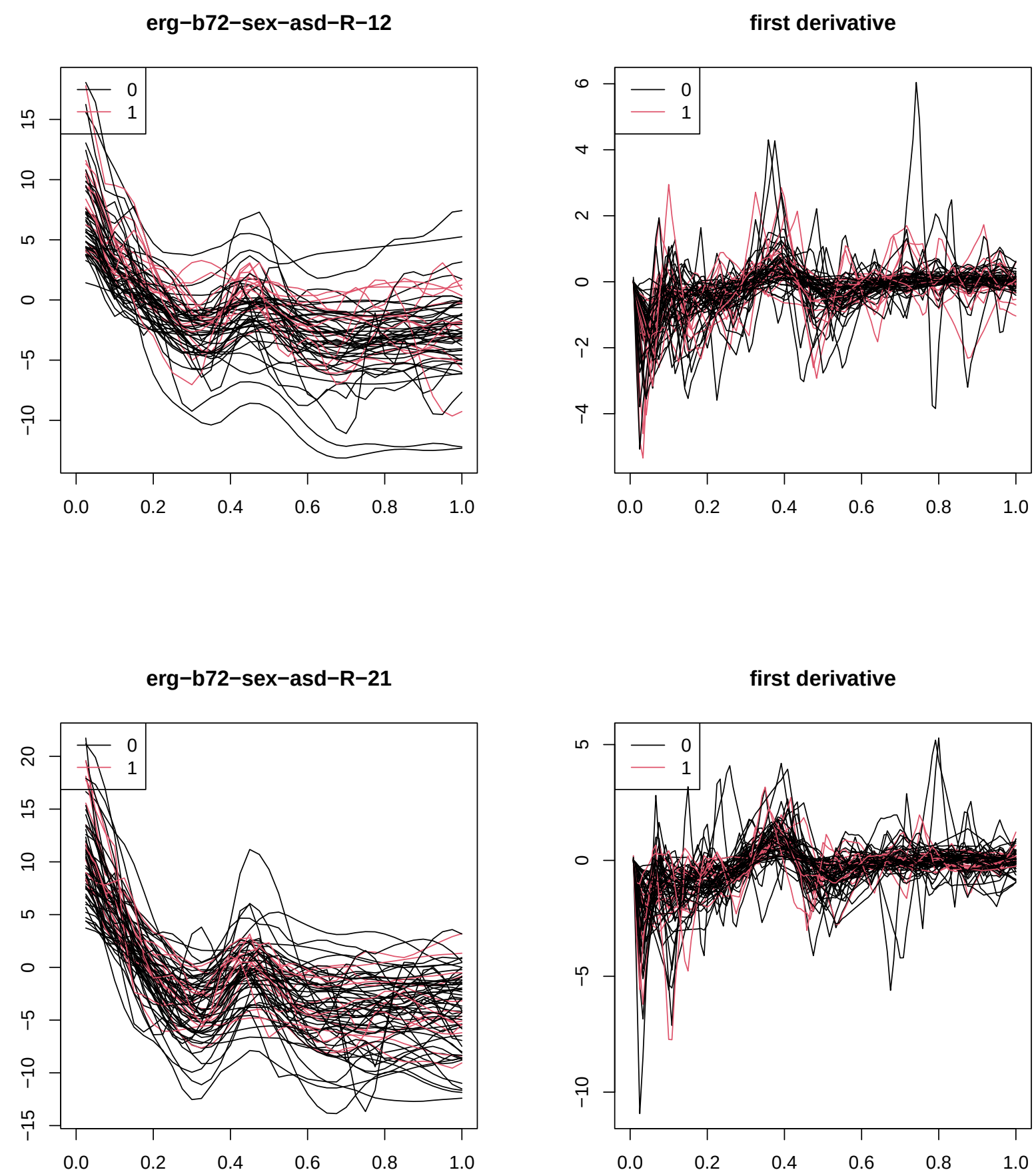

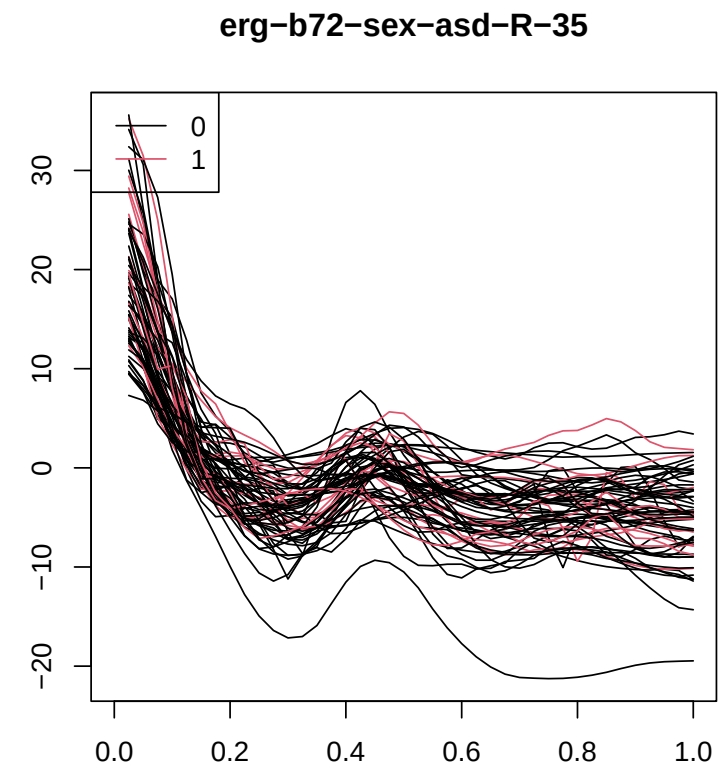
erg−b72−sex−asd−R−35
0
1

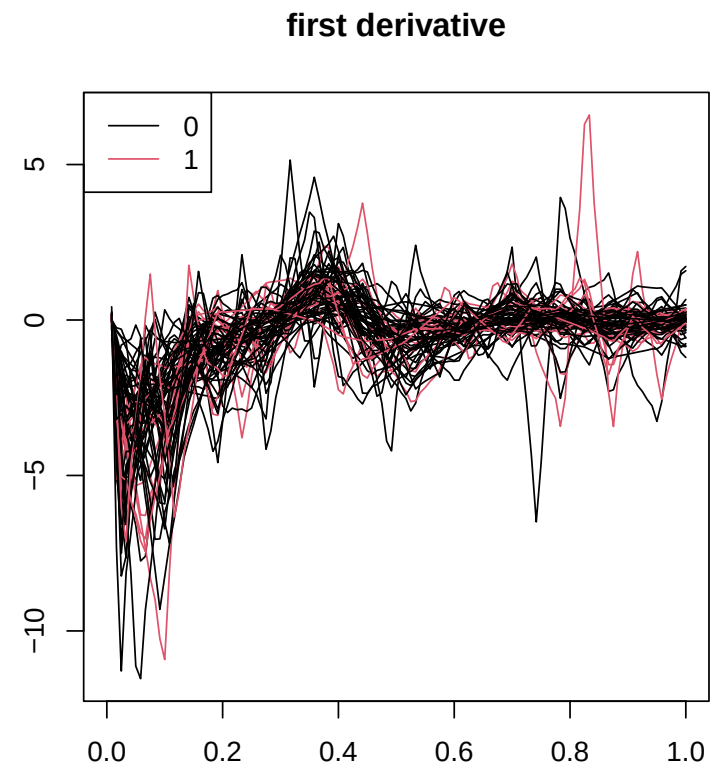
first derivative
0
1

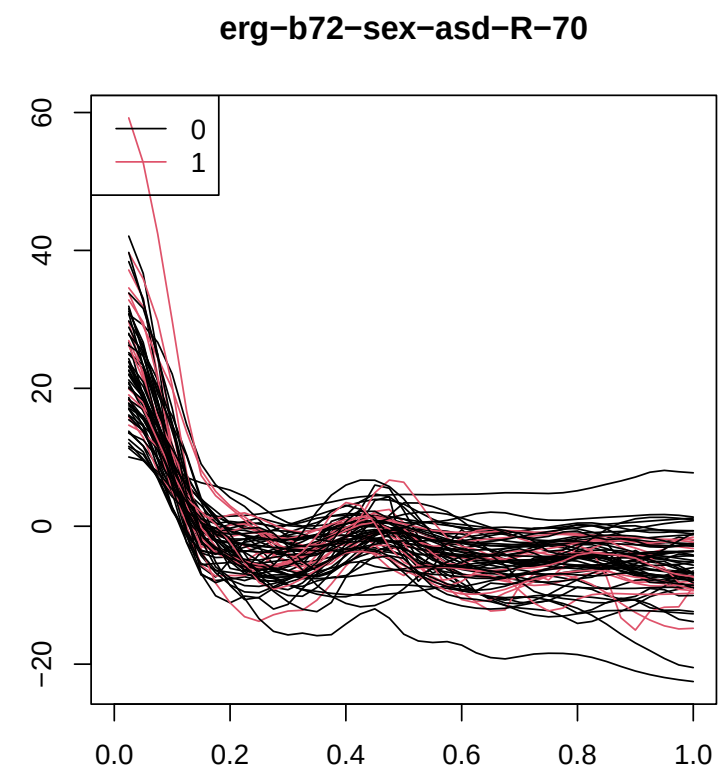
erg−b72−sex−asd−R−70
0
1

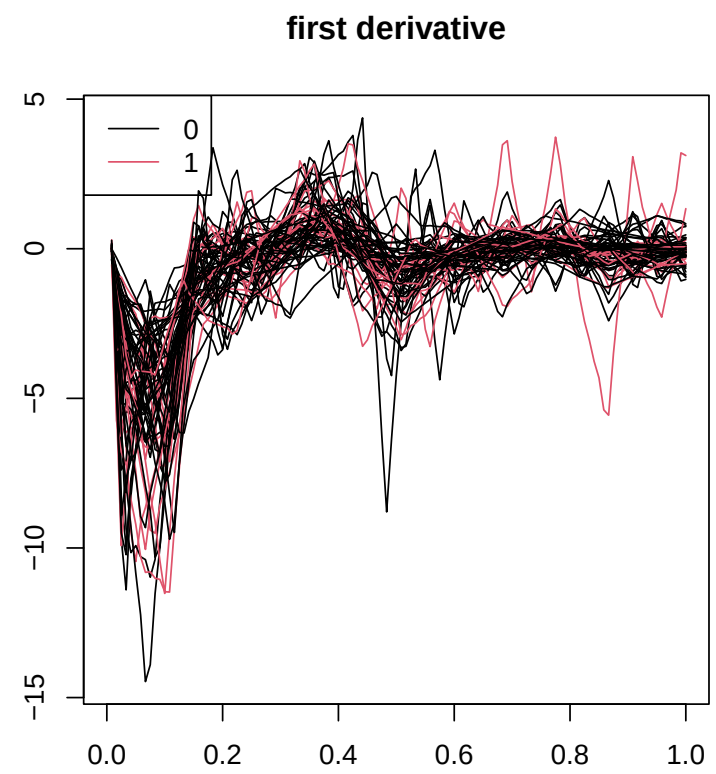
first derivative
0
1

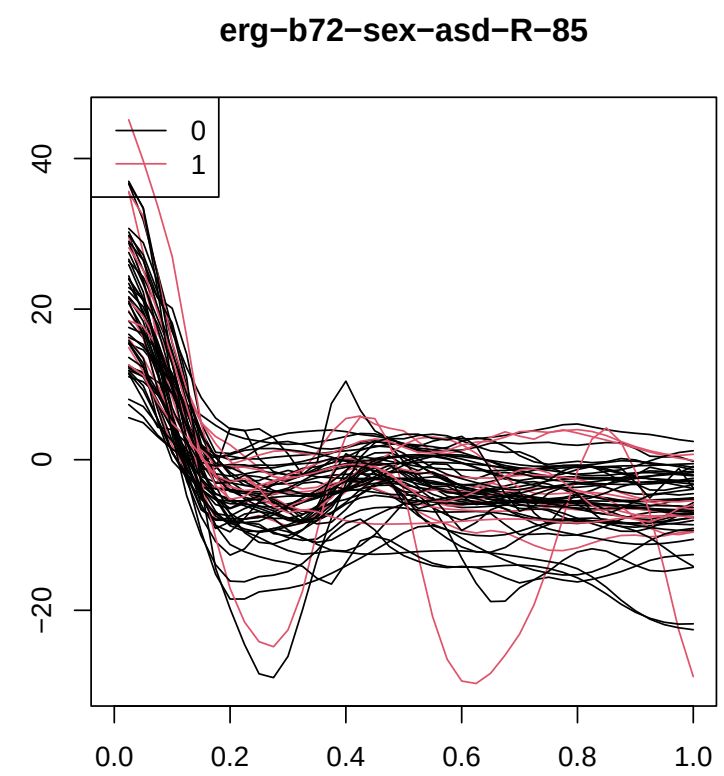
erg−b72−sex−asd−R−85
0
1

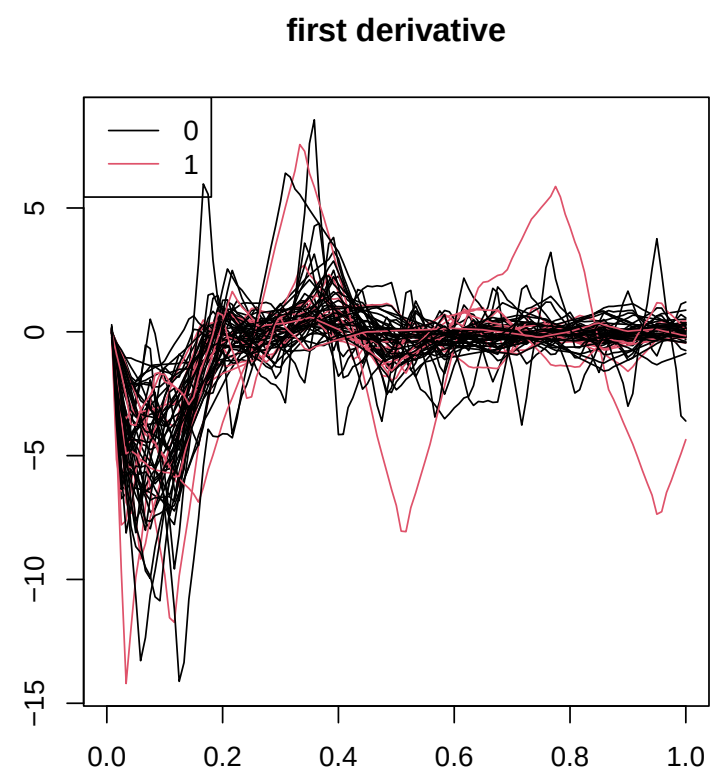
first derivative
0
1

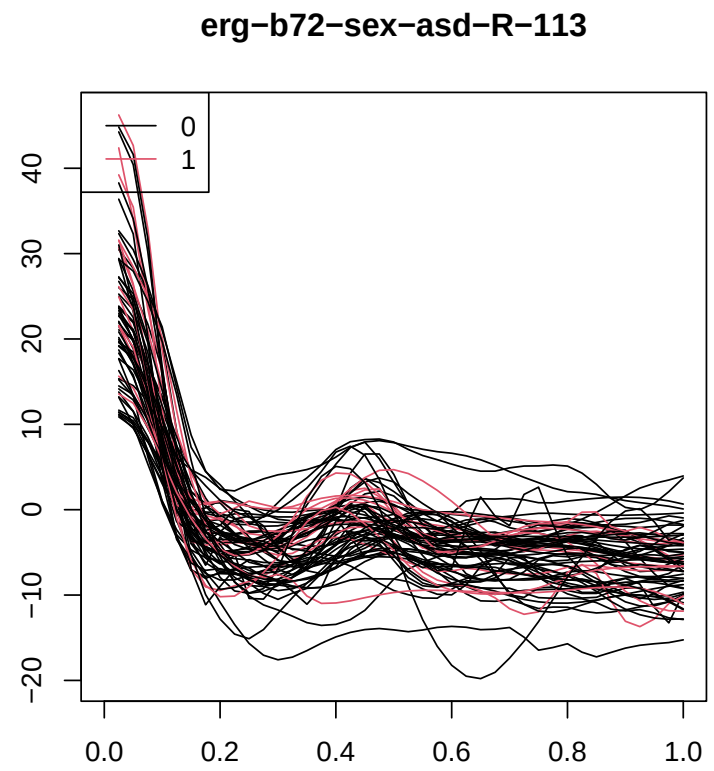
erg−b72−sex−asd−R−113
0
1
40
30
20
10
0
−10
−20
0.0
0.2
0.4
0.6
0.8
1.0
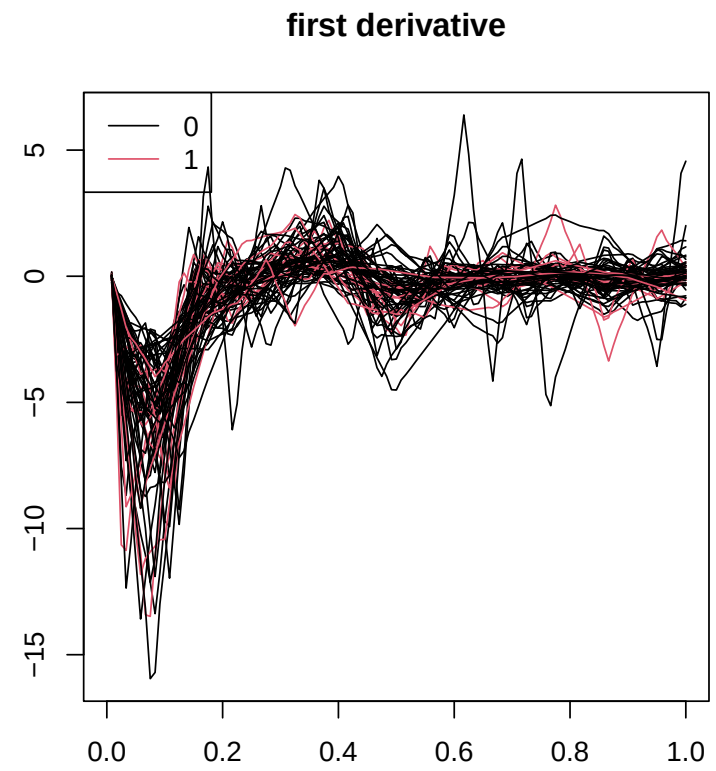
first derivative
0
1
5
0
−5
−10
−15
0.0
0.2
0.4
0.6
0.8
1.0
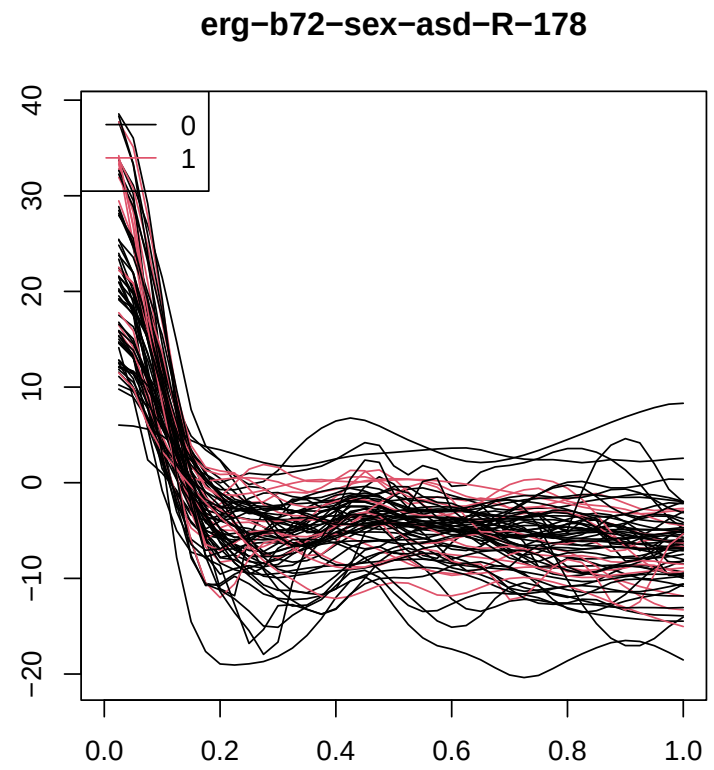
erg−b72−sex−asd−R−178
0
1
40
30
20
10
0
−10
−20
0.0
0.2
0.4
0.6
0.8
1.0
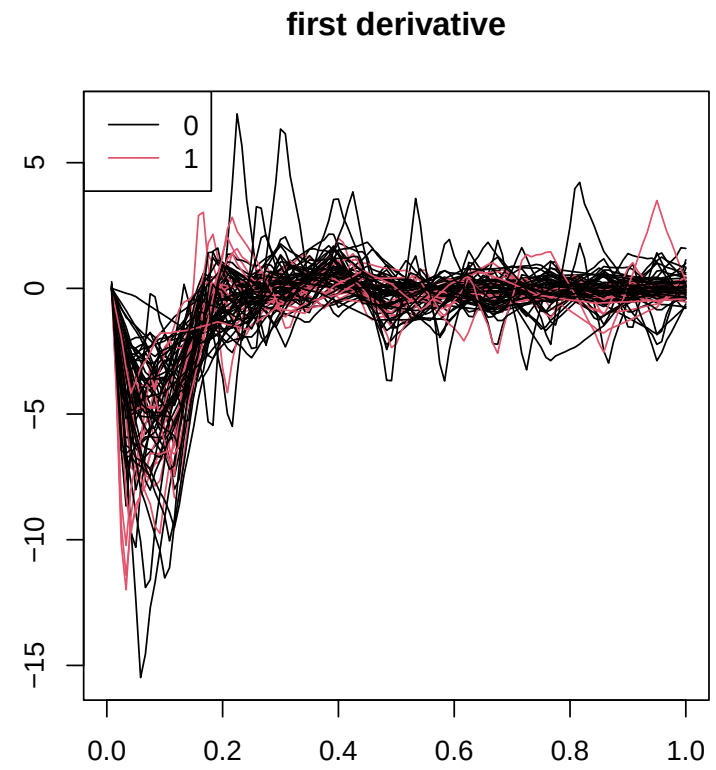
first derivative
0
1
5
0
−5
−10
−15
0.0
0.2
0.4
0.6
0.8
1.0
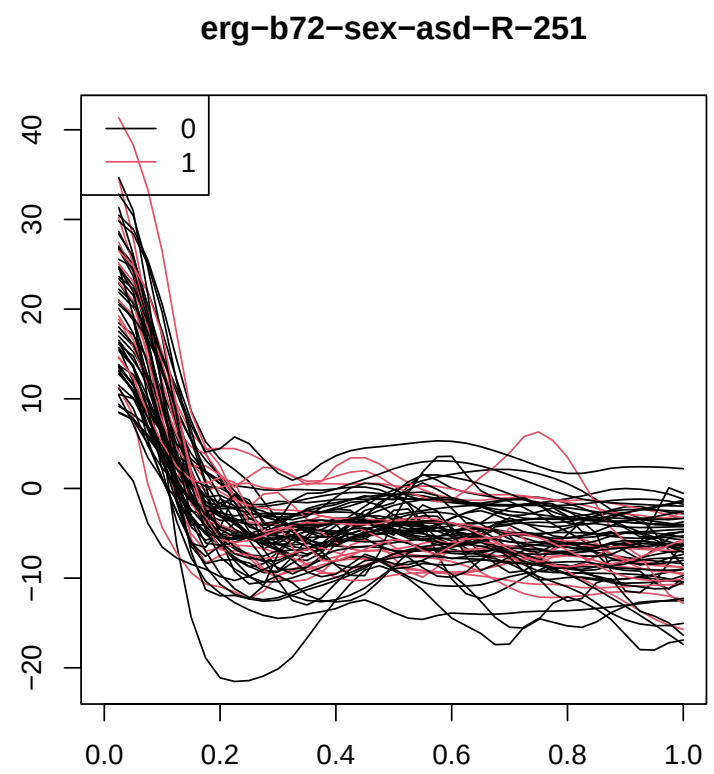
erg−b72−sex−asd−R−251
0
1
40
30
20
10
0
−10
−20
0.0
0.2
0.4
0.6
0.8
1.0
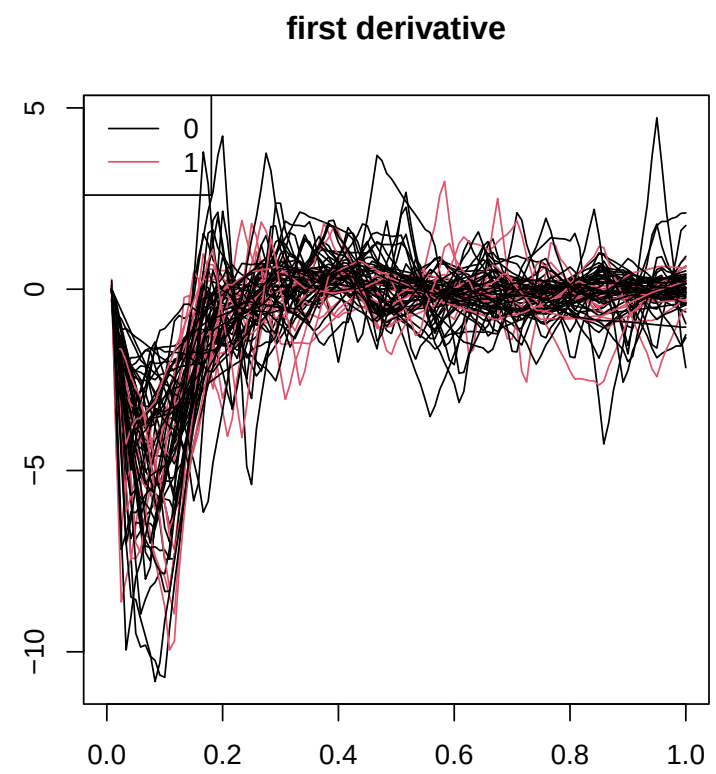
first derivative
0
1
5
0
−5
−10
0.0
0.2
0.4
0.6
0.8
1.0

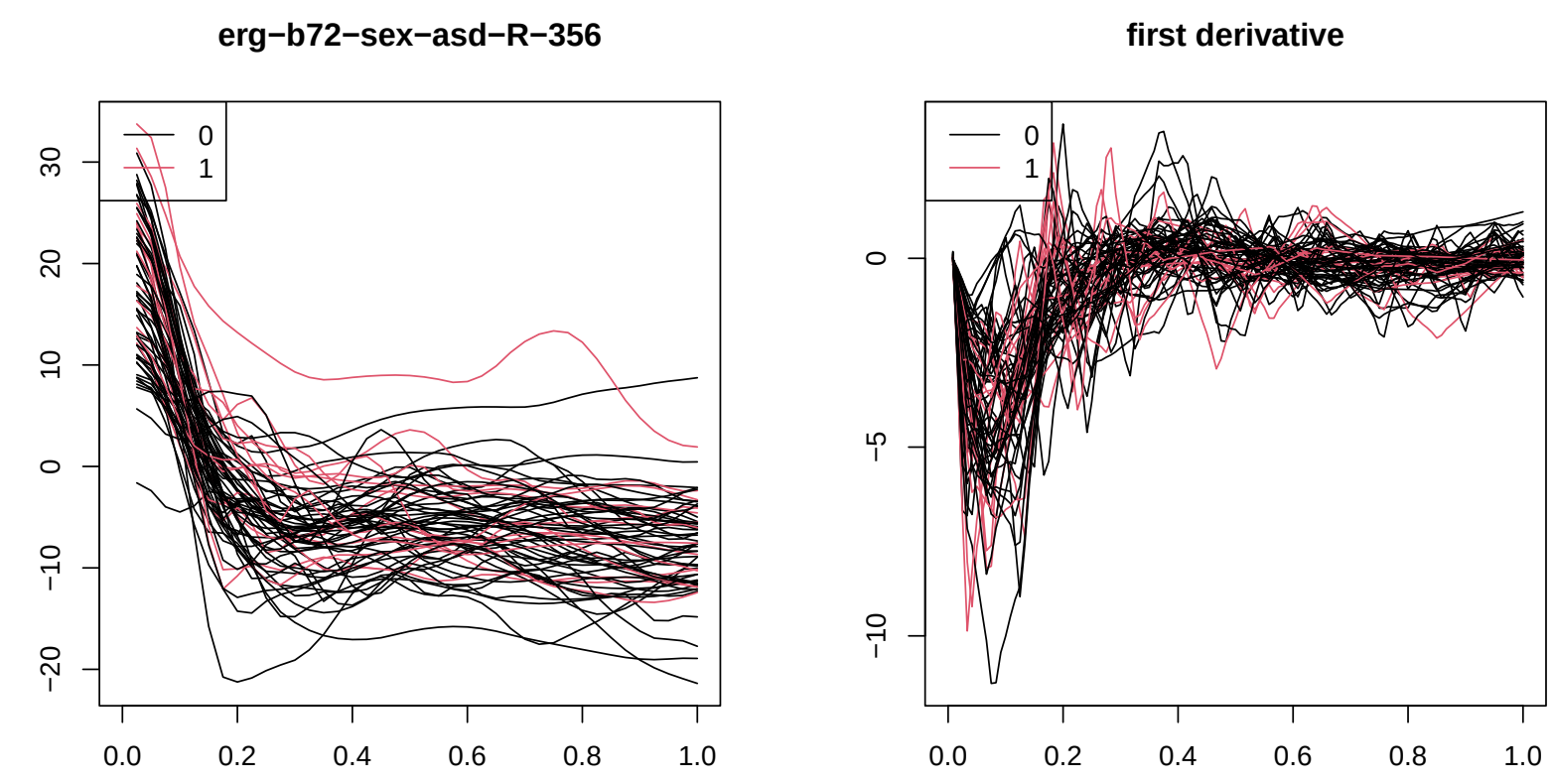
erg−b72−sex−asd−R−356
first derivative
0
1

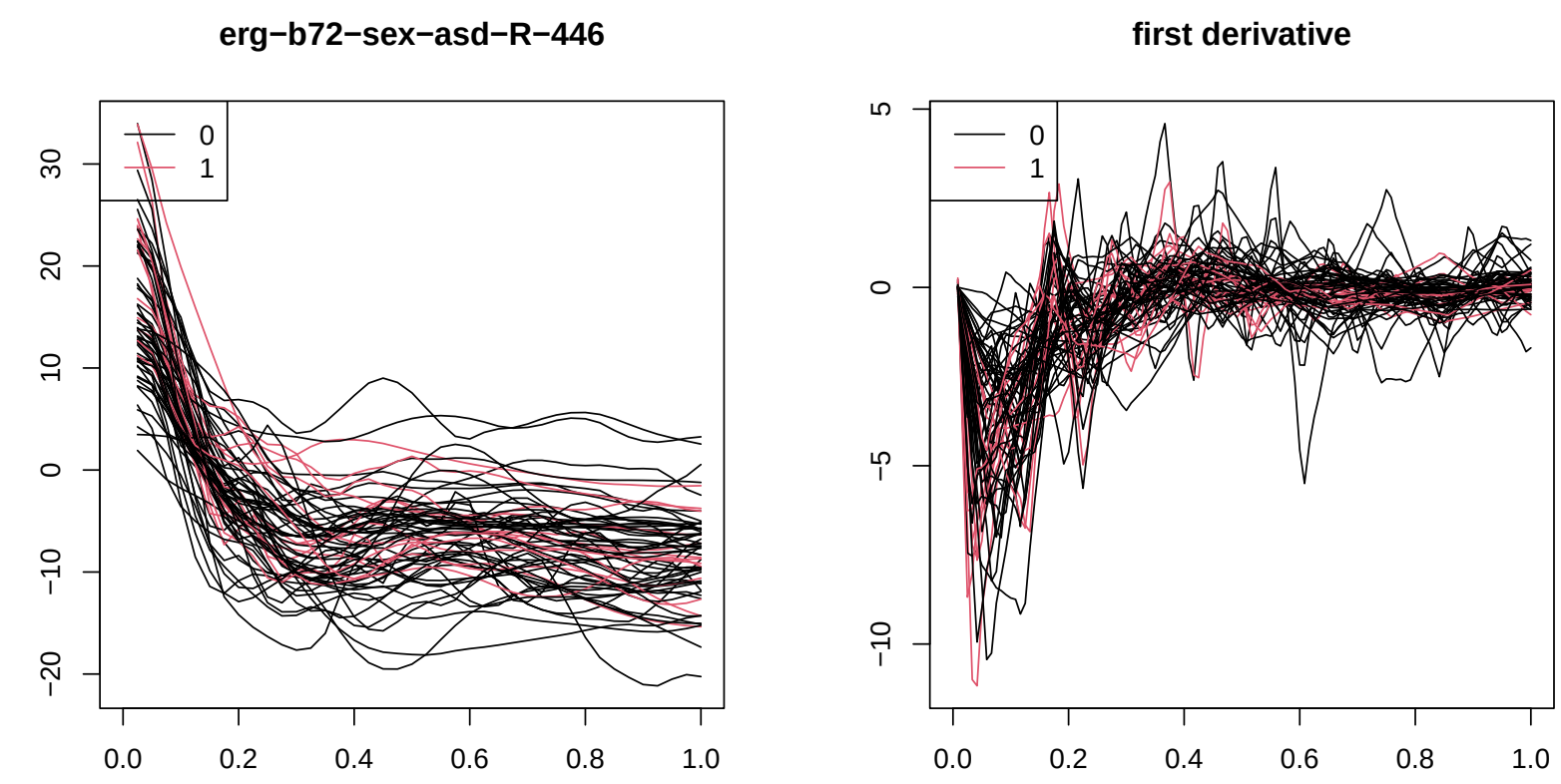
erg−b72−sex−asd−R−446
first derivative
0
1

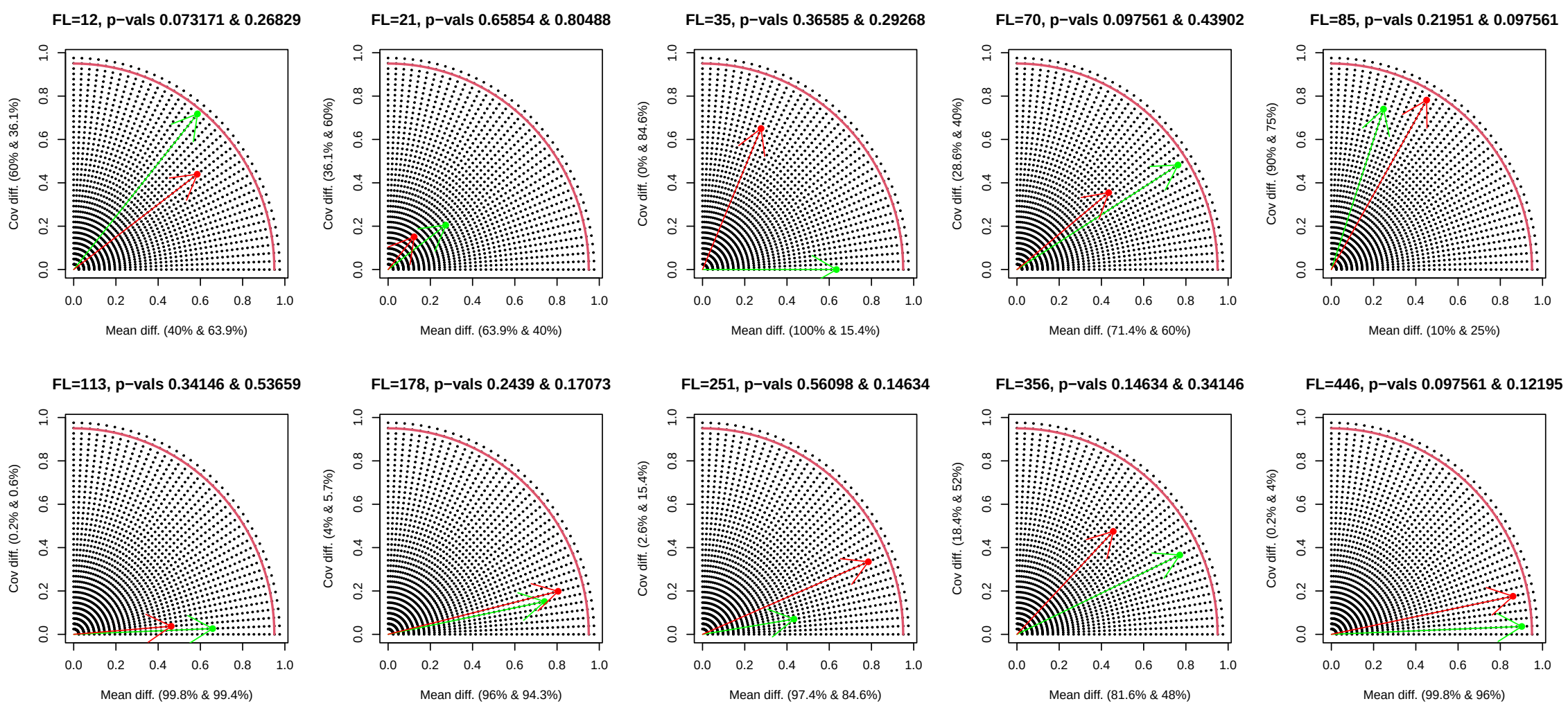
FL=12, p−vals 0.073171 & 0.26829
FL=21, p−vals 0.65854 & 0.80488
FL=35, p−vals 0.36585 & 0.29268
FL=70, p−vals 0.097561 & 0.43902
FL=85, p−vals 0.21951 & 0.097561
FL=113, p−vals 0.34146 & 0.53659
FL=178, p−vals 0.2439 & 0.17073
FL=251, p−vals 0.56098 & 0.14634
FL=356, p−vals 0.14634 & 0.34146
FL=446, p−vals 0.097561 & 0.12195

Table for ERG wave by sex in btime–72 – asd subgroup

	pval-erg-b72	mean perc	cov perc	pval-derivative	mean perc	cov perc
12	0.0732			0.2683		
21	0.6585			0.8049		
35	0.3659			0.2927		
70	0.0976			0.4390		
85	0.2195			0.0976		
113	0.3415			0.5366		
178	0.2439			0.1707		
251	0.5610			0.1463		
356	0.1463			0.3415		
446	0.0976			0.1220		

2.13 OP wave by age in 0–60 – ctr subgroup

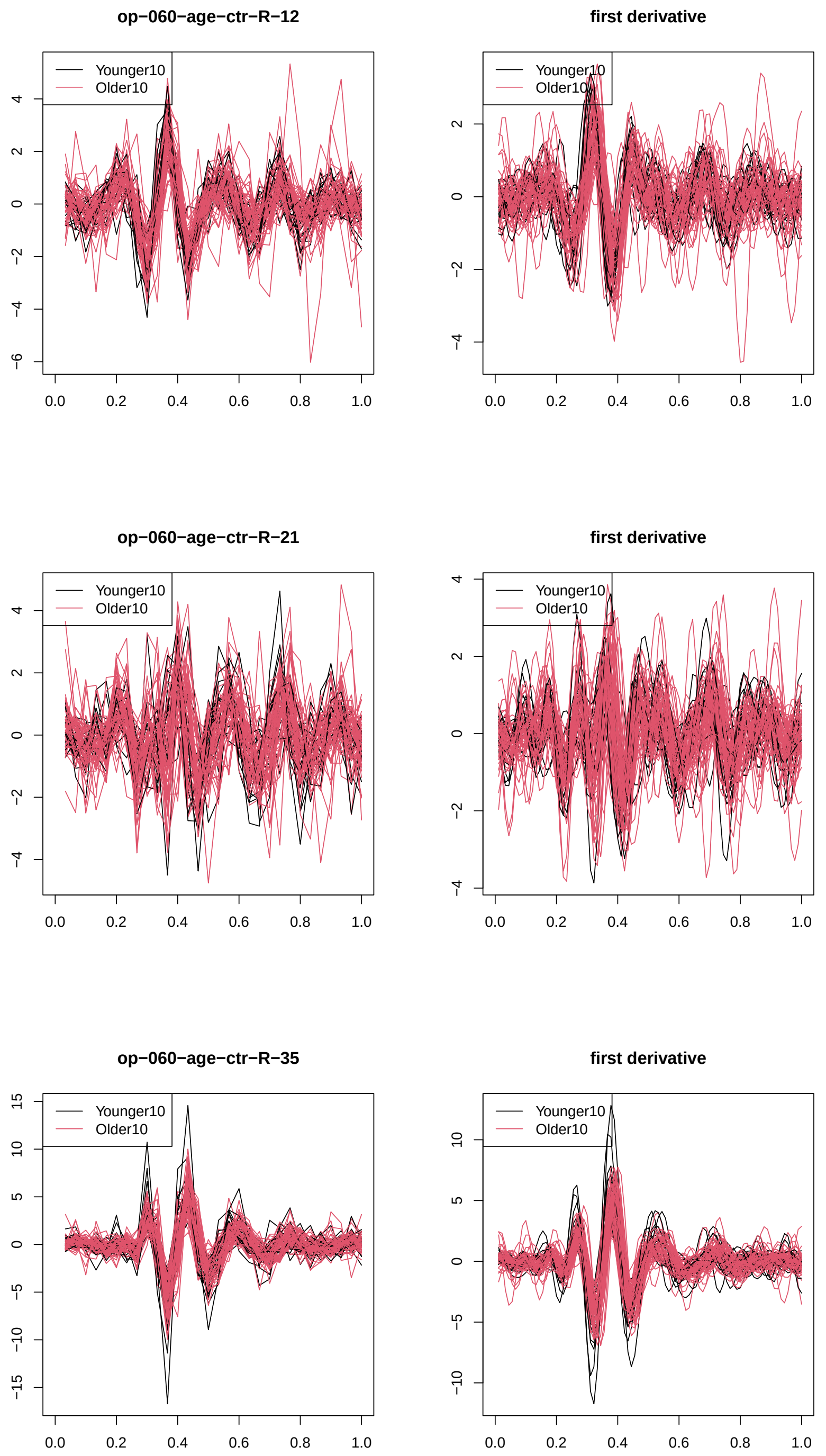

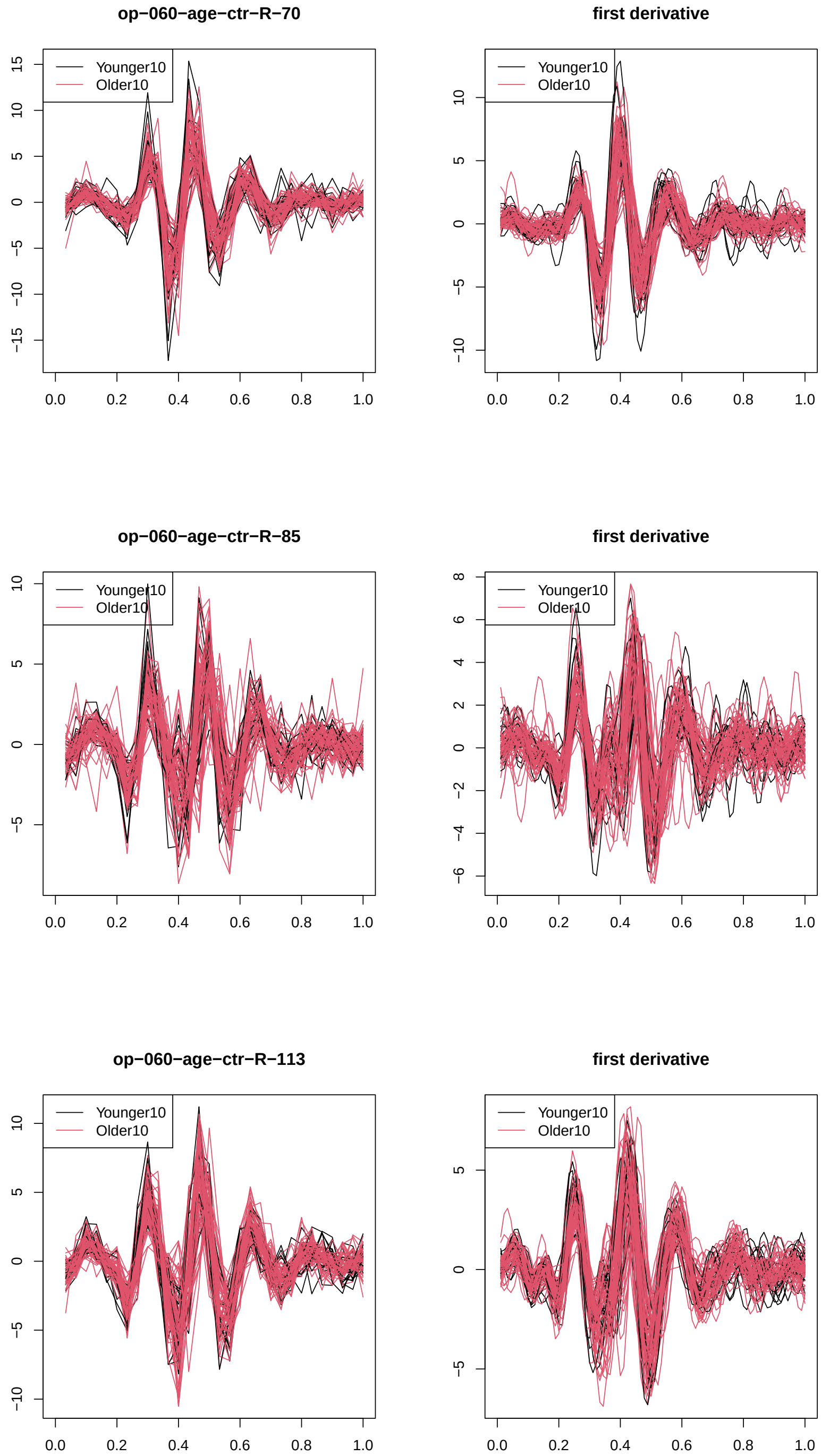
op−060−age−ctr−R−70
Younger10
Older10
15
10
5
0
−5
−10
−15
0.0
0.2
0.4
0.6
0.8
1.0
first derivative
Younger10
Older10
10
5
0
−5
−10
0.0
0.2
0.4
0.6
0.8
1.0
op−060−age−ctr−R−85
Younger10
Older10
10
5
0
−5
0.0
0.2
0.4
0.6
0.8
1.0
first derivative
Younger10
Older10
8
6
4
2
0
−2
−4
−6
0.0
0.2
0.4
0.6
0.8
1.0
op−060−age−ctr−R−113
Younger10
Older10
10
5
0
−5
−10
0.0
0.2
0.4
0.6
0.8
1.0
first derivative
Younger10
Older10
5
0
−5
0.0
0.2
0.4
0.6
0.8
1.0

op−060−age−ctr−R−178

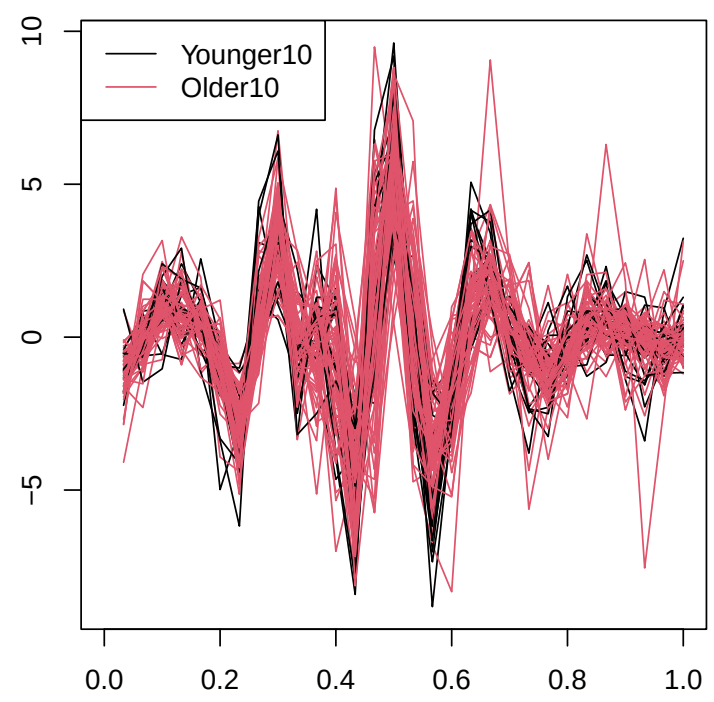

first derivative

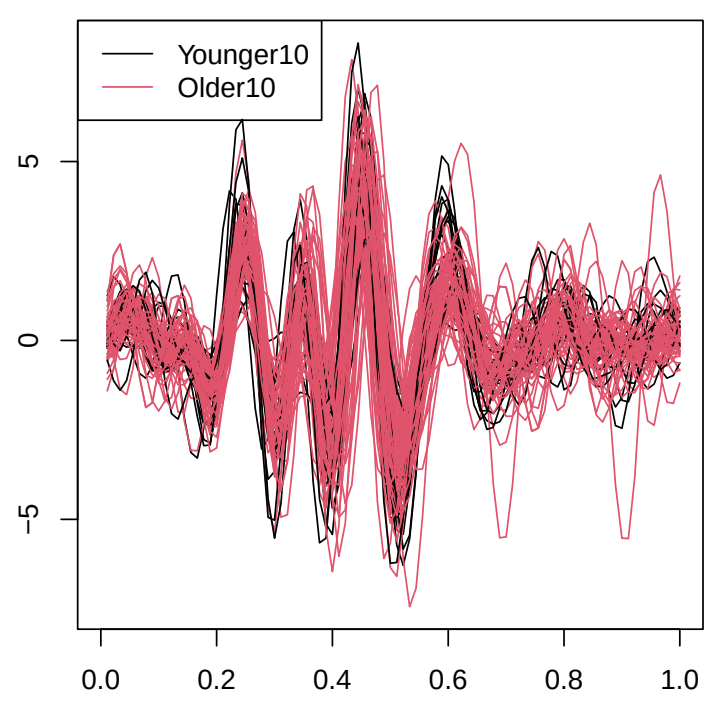

op−060−age−ctr−R−251

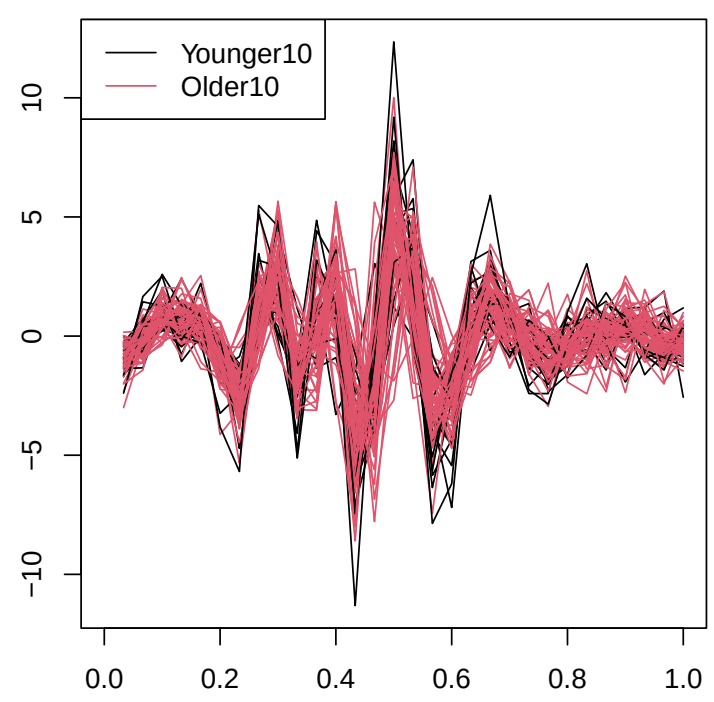

first derivative

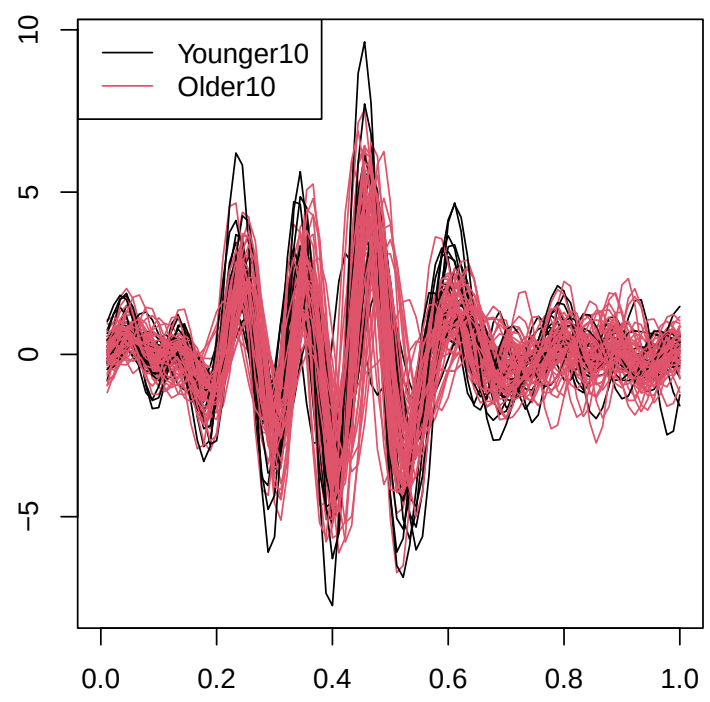

op−060−age−ctr−R−356

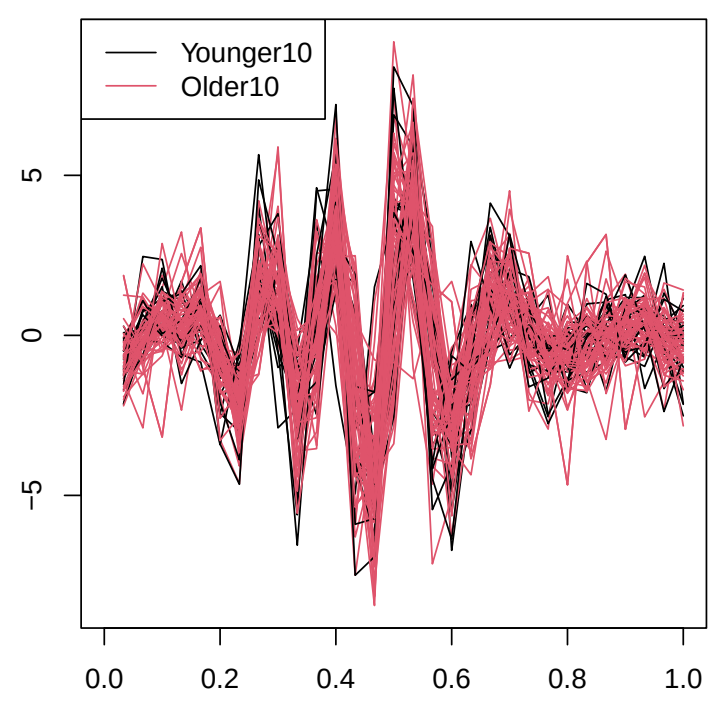

first derivative

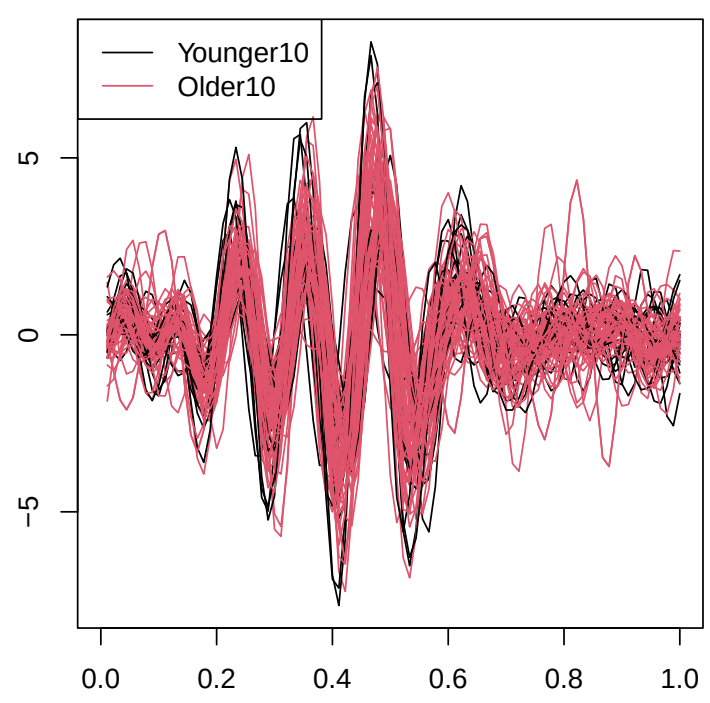

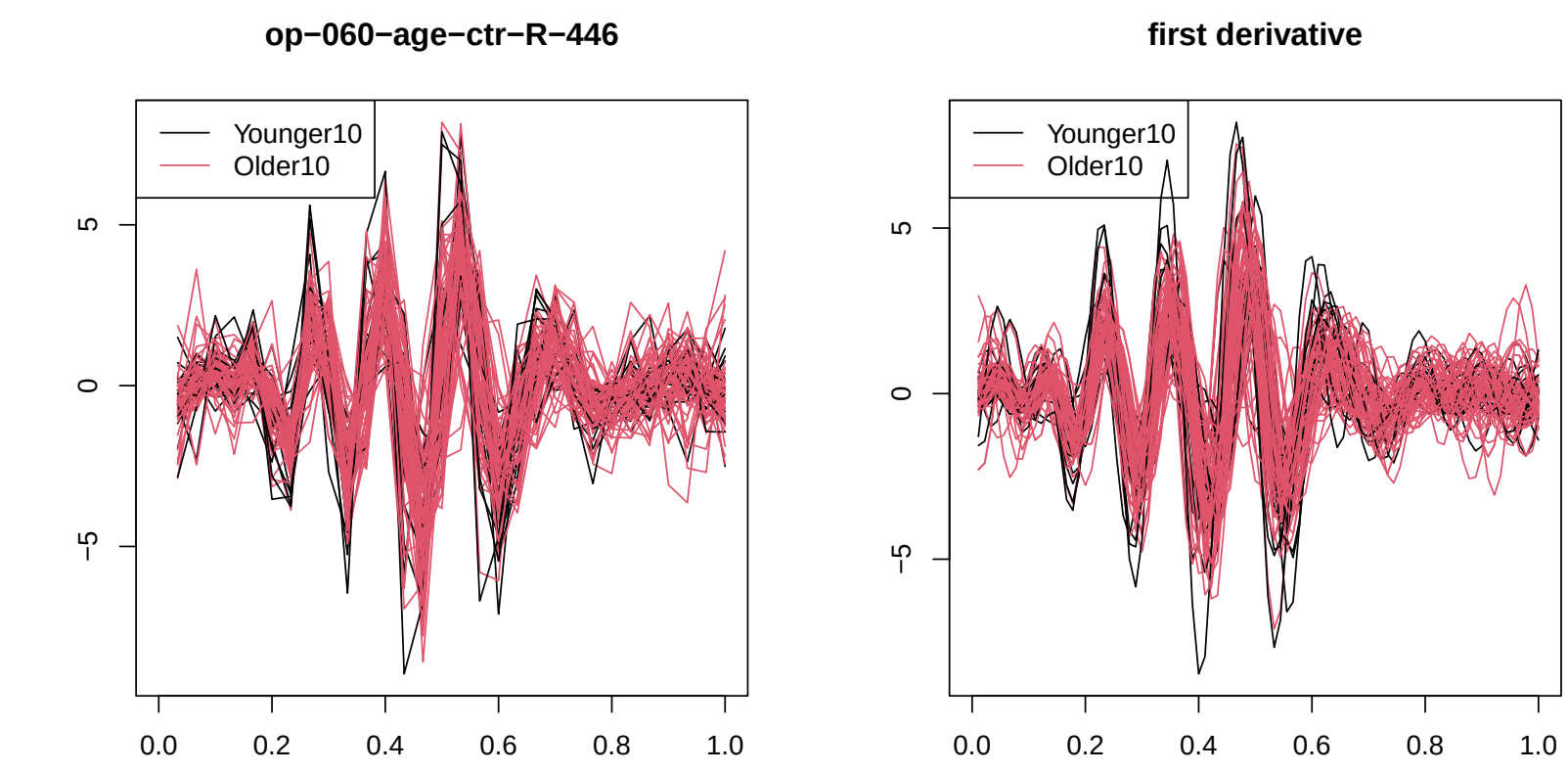

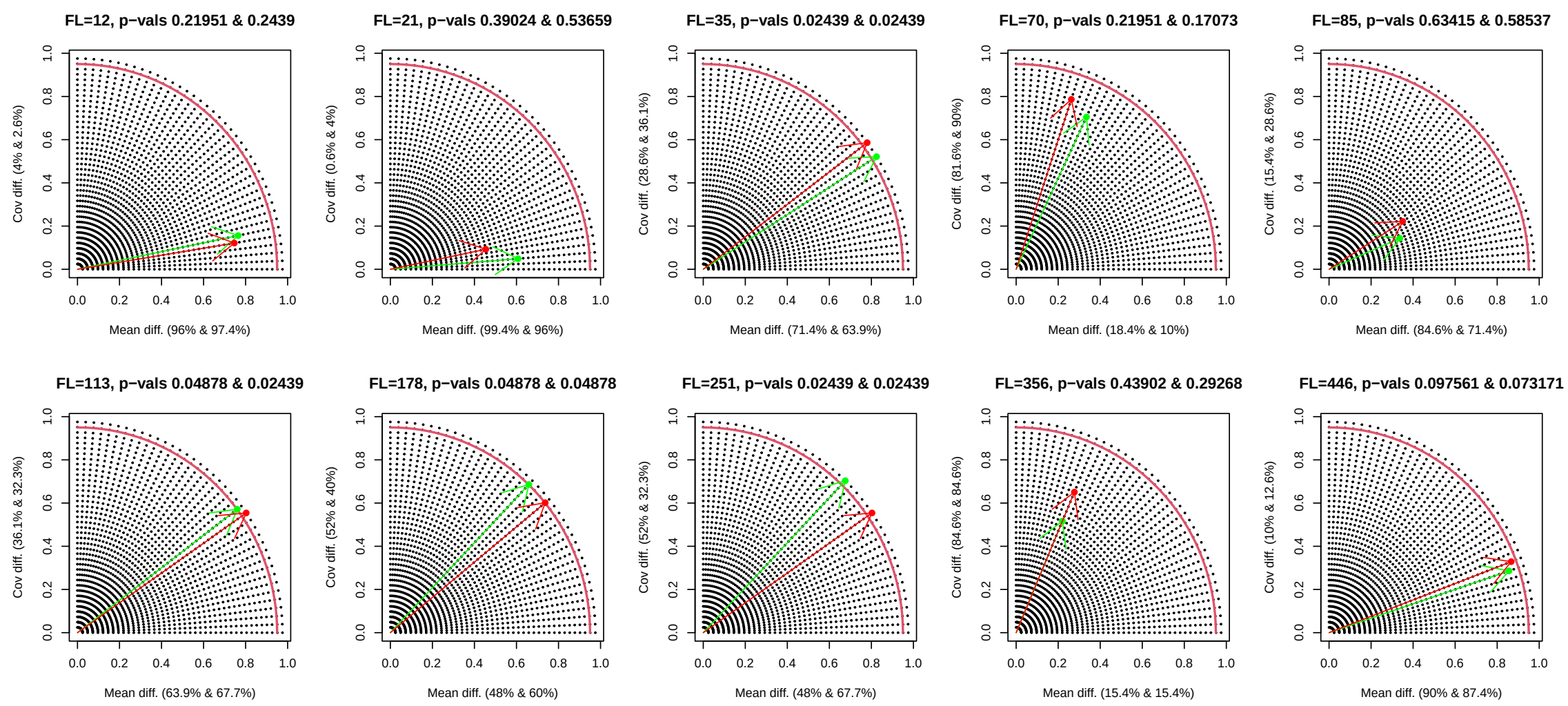

Table for OP wave by age in 0–60 – ctr subgroup

	pval-op-060	mean perc	cov perc	pval-derivative	mean perc	cov perc
12	0.2195			0.2439		
21	0.3902			0.5366		
35	0.0244	71.4	28.6	0.0244	63.9	36.1
70	0.2195			0.1707		
85	0.6341			0.5854		
113	0.0488	63.9	36.1	0.0244	67.7	32.3
178	0.0488	48	52	0.0488	60	40
251	0.0244	48	52	0.0244	67.7	32.3
356	0.4390			0.2927		
446	0.0976			0.0732		

2.14 OP wave by age in 0–60 – asd subgroup

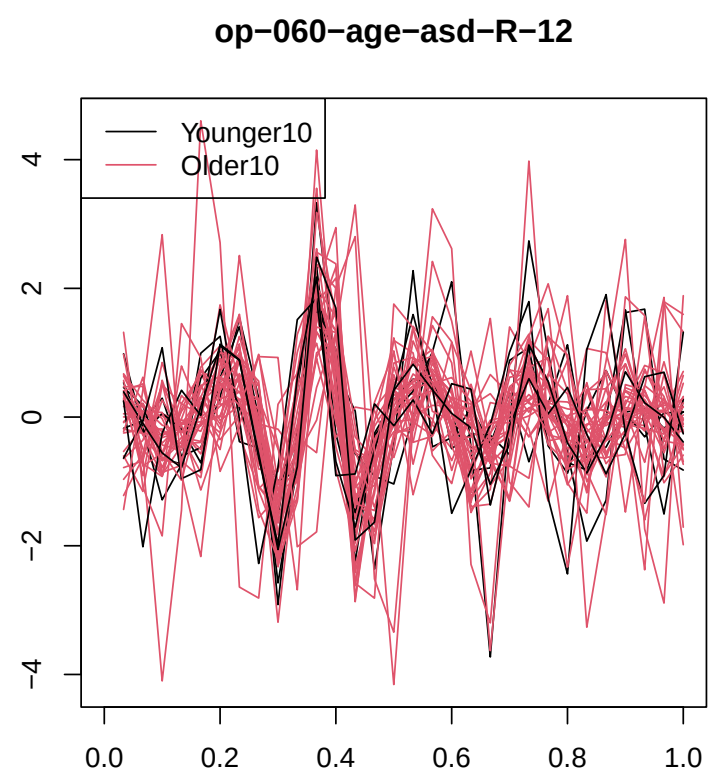

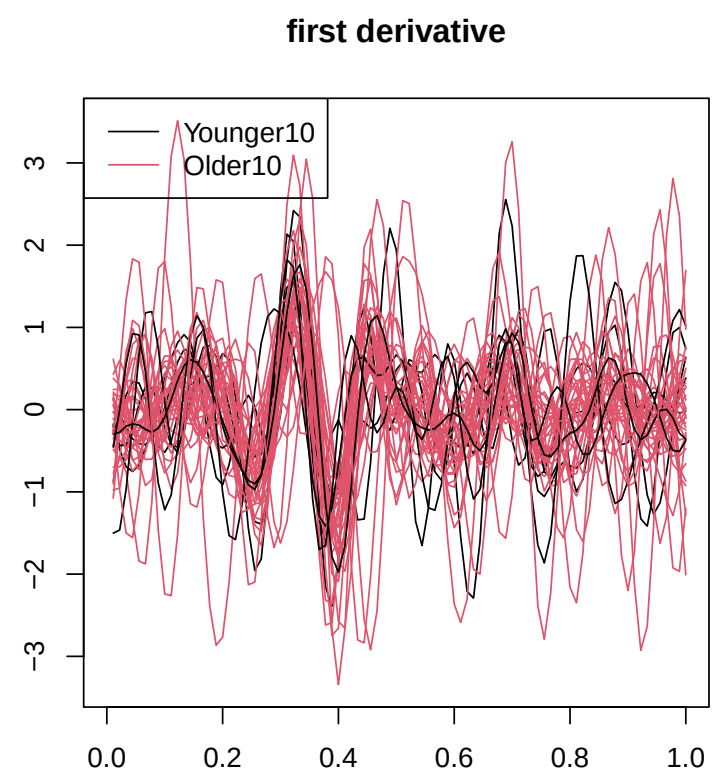

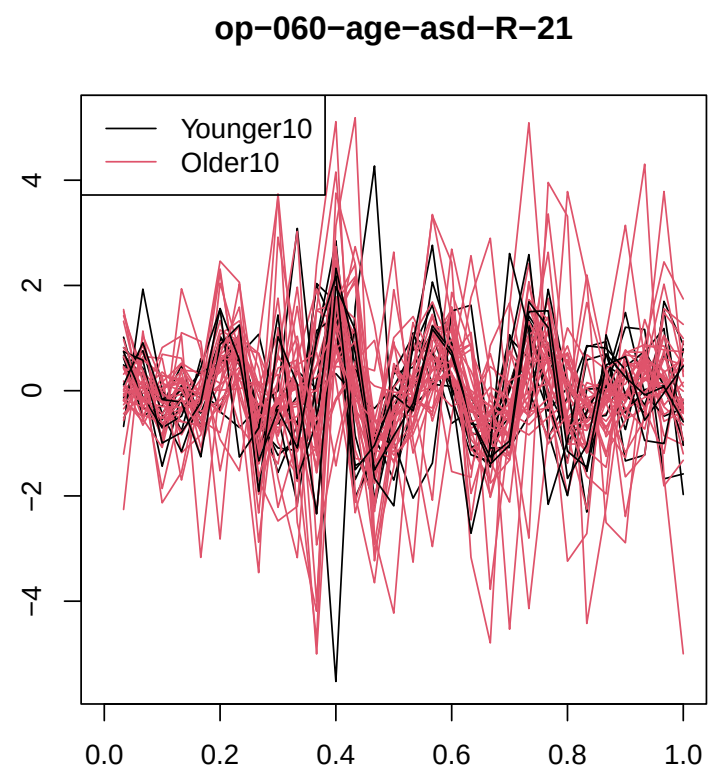

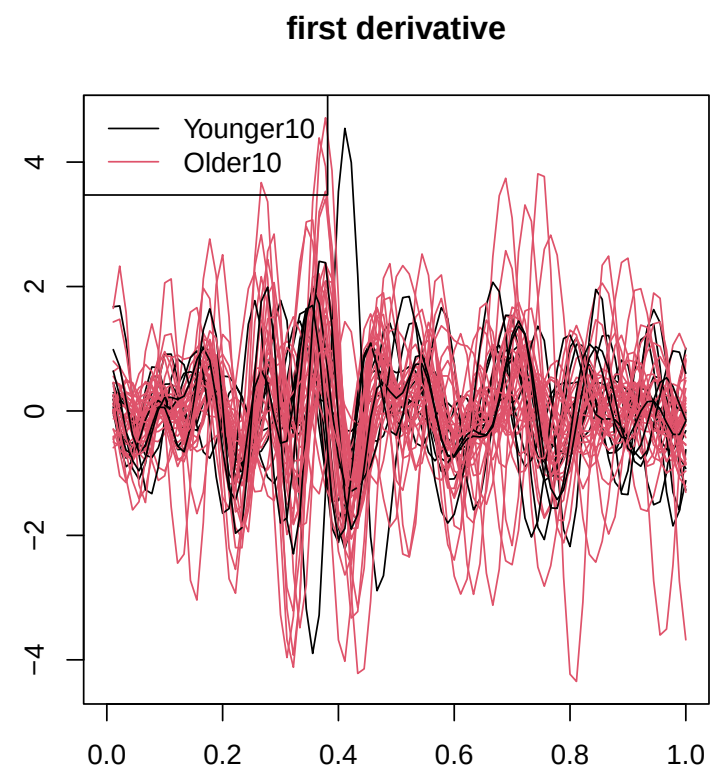

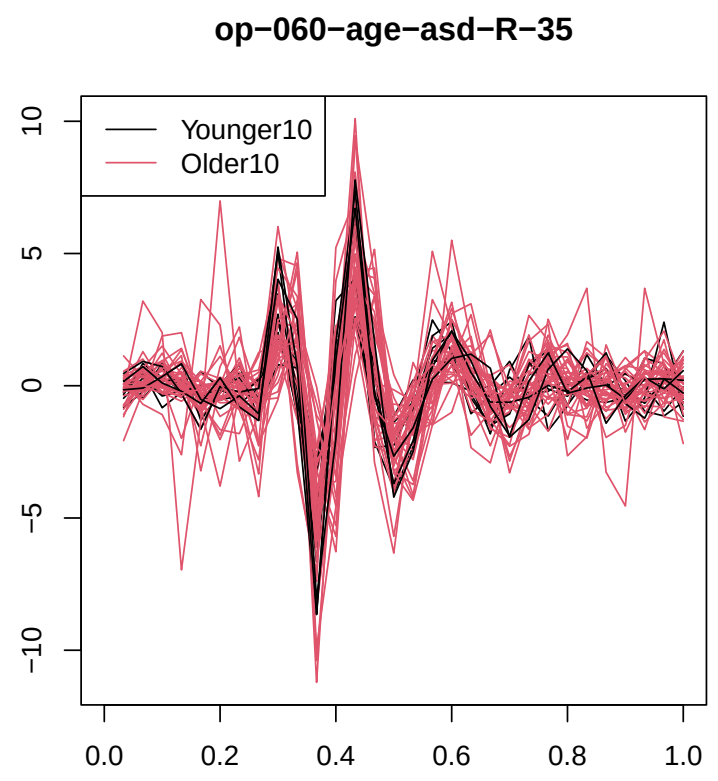

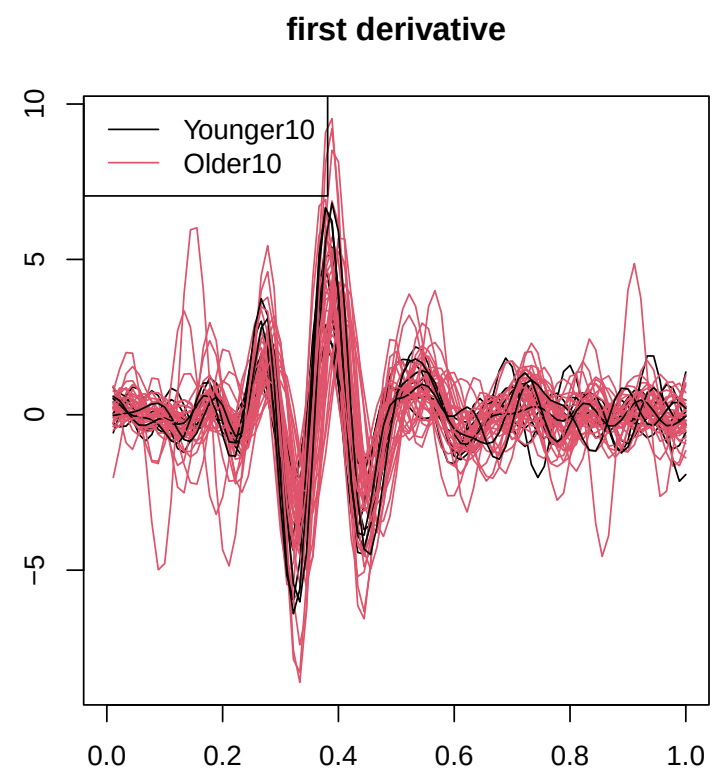

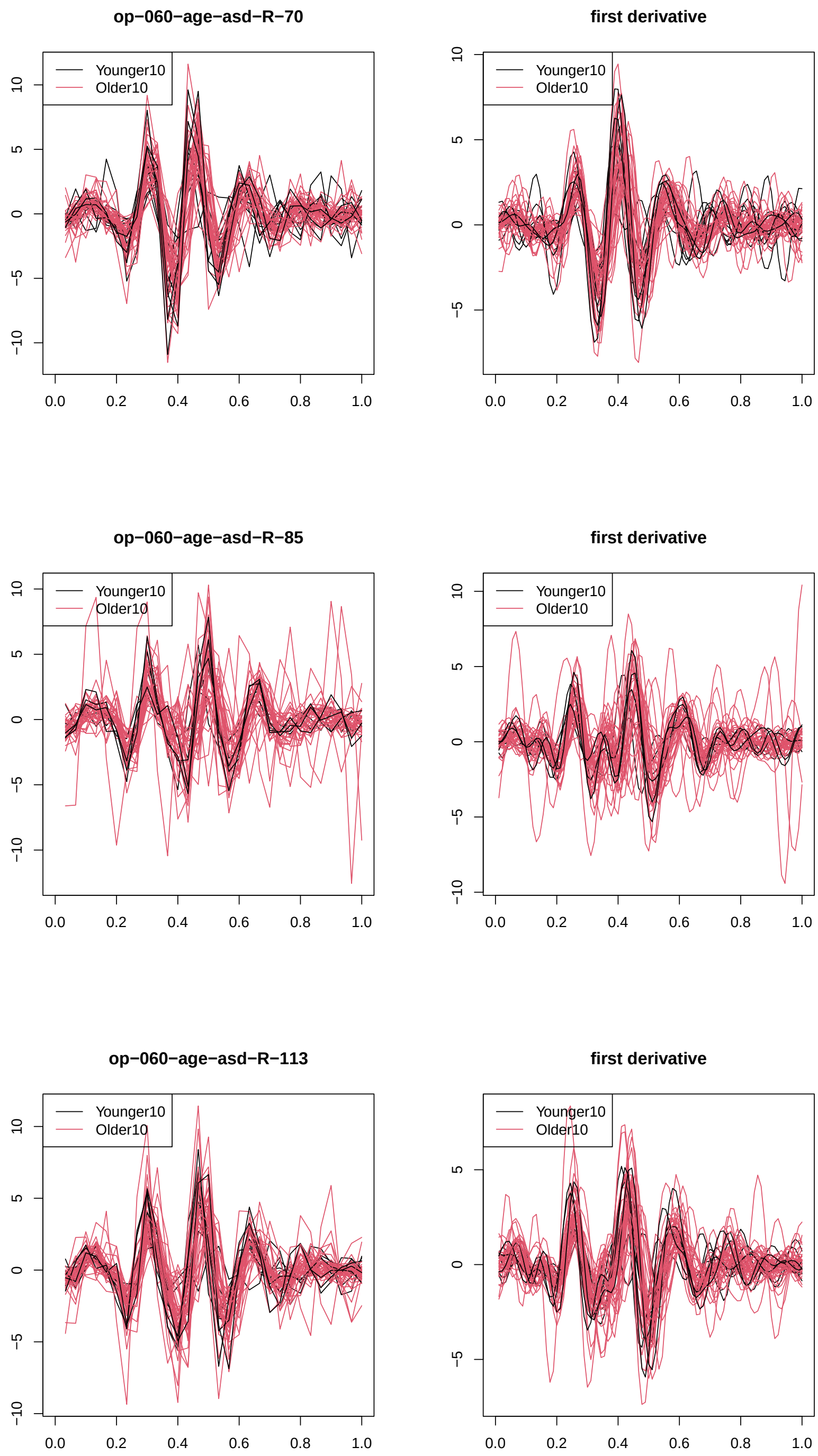
op−060−age−asd−R−70
first derivative
Younger10
Older10
op−060−age−asd−R−85
first derivative
Younger10
Older10
op−060−age−asd−R−113
first derivative
Younger10
Older10
0.0
0.2
0.4
0.6
0.8
1.0
−10
−5
0
5
10

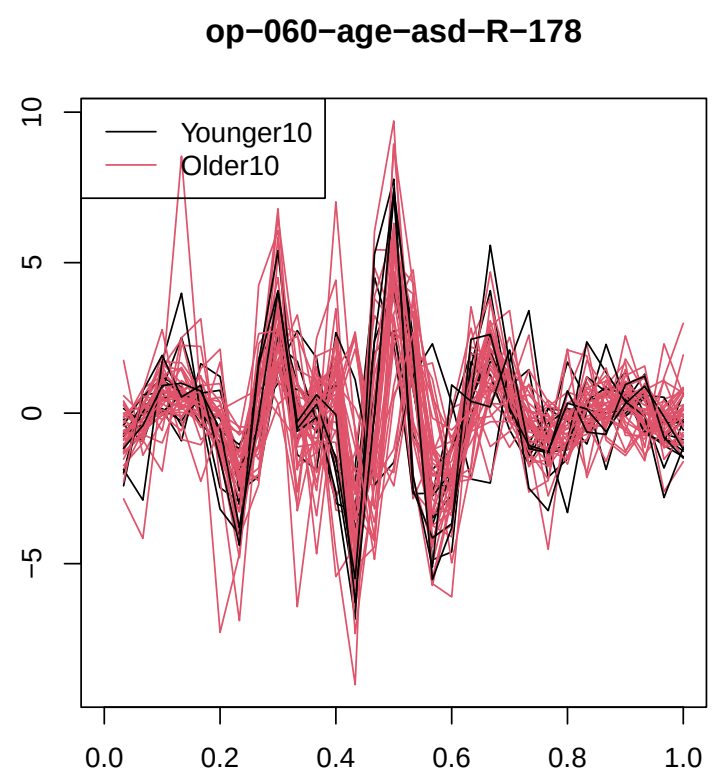
op−060−age−asd−R−178
Younger10
Older10

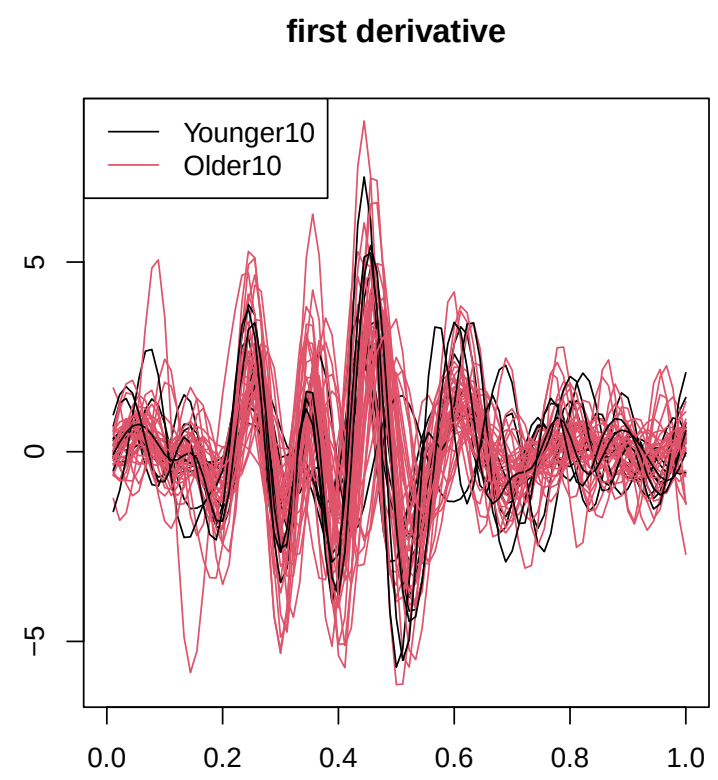
first derivative
Younger10
Older10

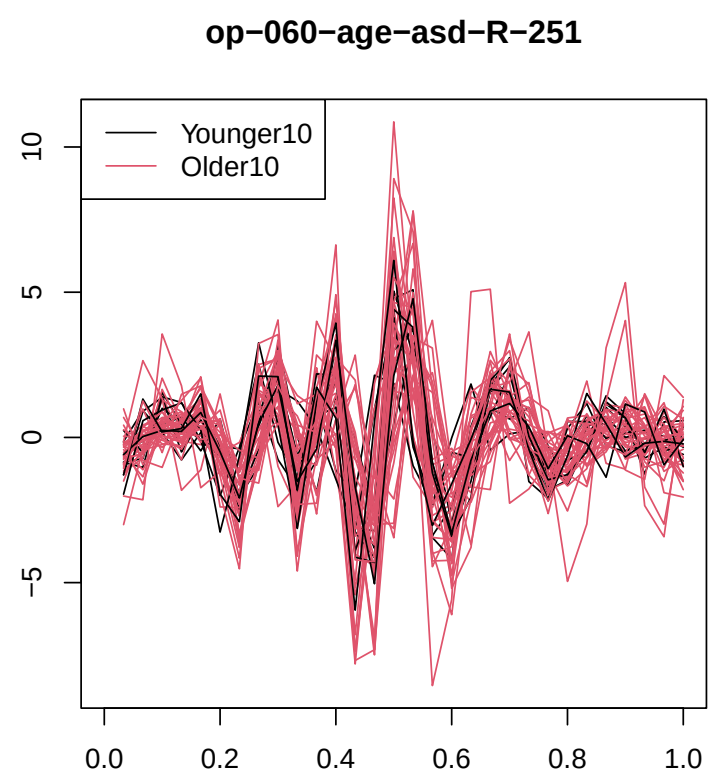
op−060−age−asd−R−251
Younger10
Older10

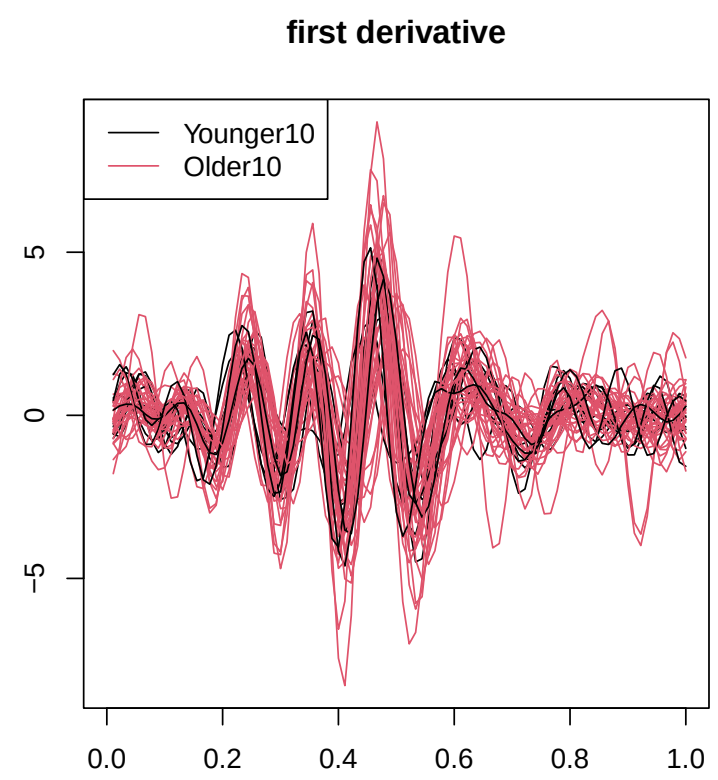
first derivative
Younger10
Older10

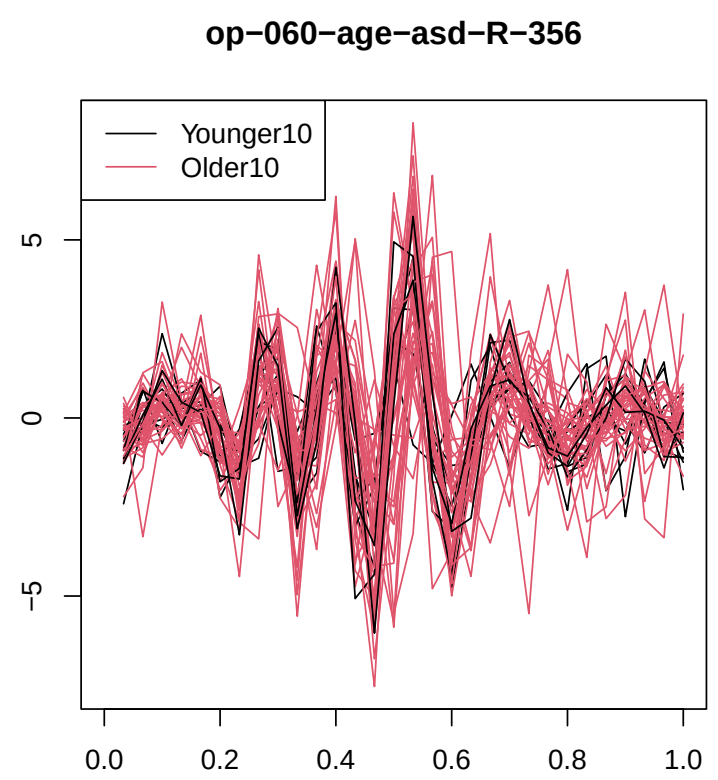
op−060−age−asd−R−356
Younger10
Older10

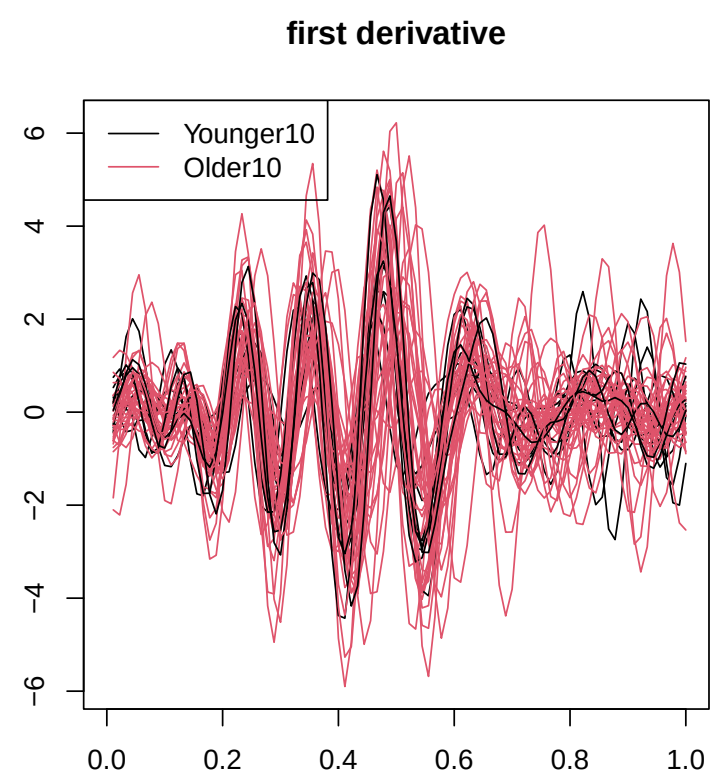
first derivative
Younger10
Older10

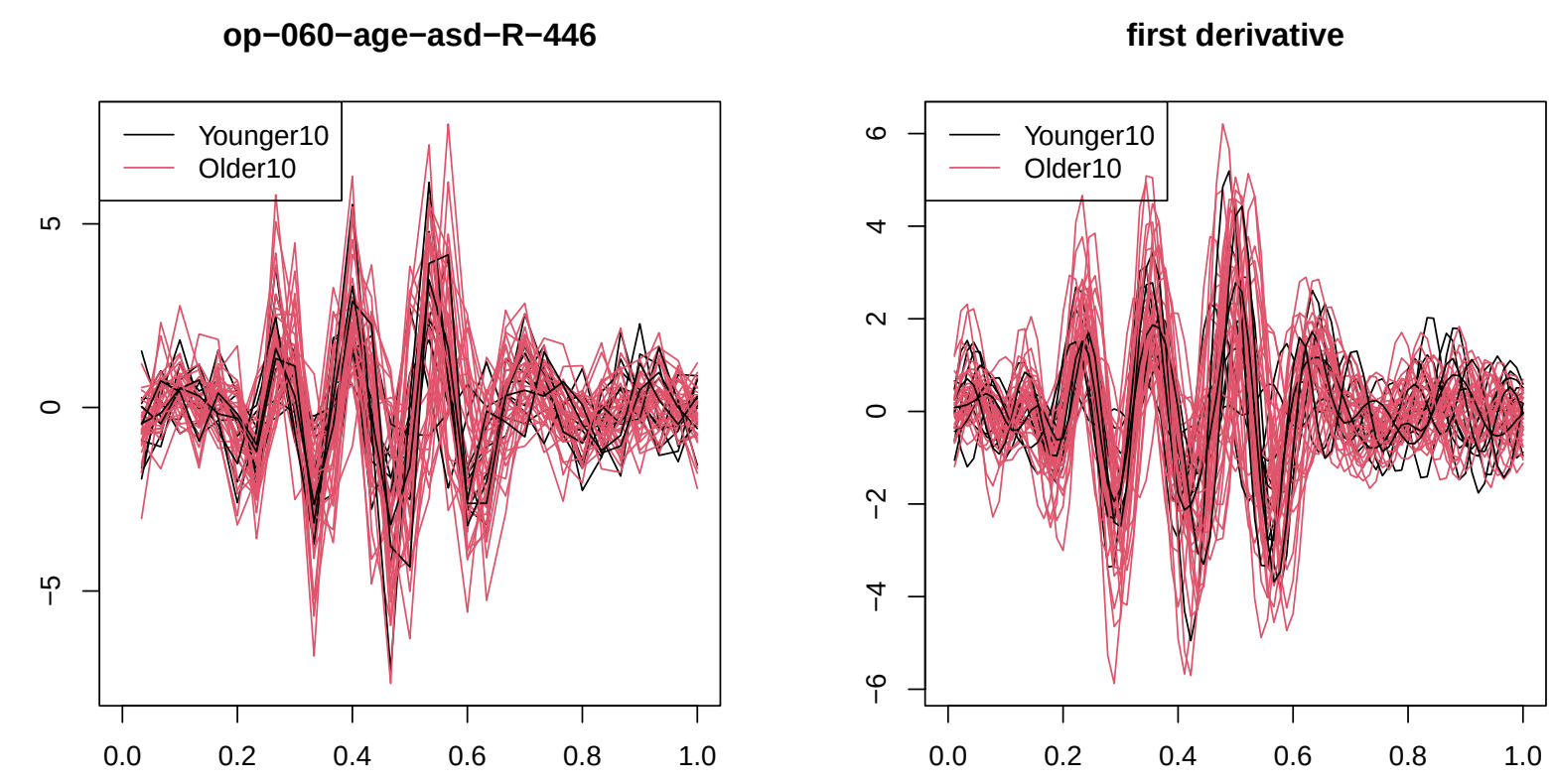

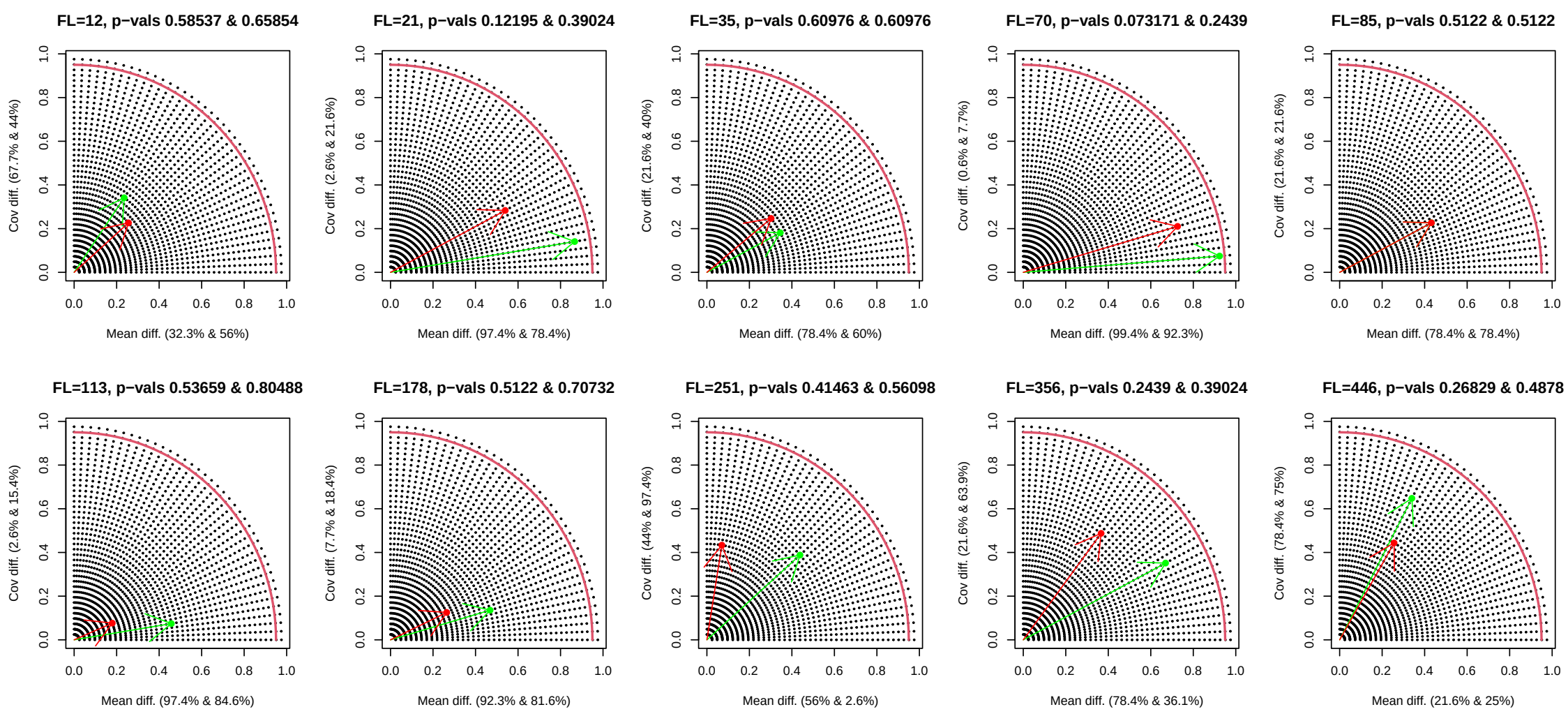

Table for OP wave by age in 0–60 – asd subgroup

	pval-op-060	mean perc	cov perc	pval-derivative	mean perc	cov perc
12	0.5854			0.6585		
21	0.1220			0.3902		
35	0.6098			0.6098		
70	0.0732			0.2439		
85	0.5122			0.5122		
113	0.5366			0.8049		
178	0.5122			0.7073		
251	0.4146			0.5610		
356	0.2439			0.3902		
446	0.2683			0.4878		

2.15 ERG wave by age in 0–atime – ctr subgroup

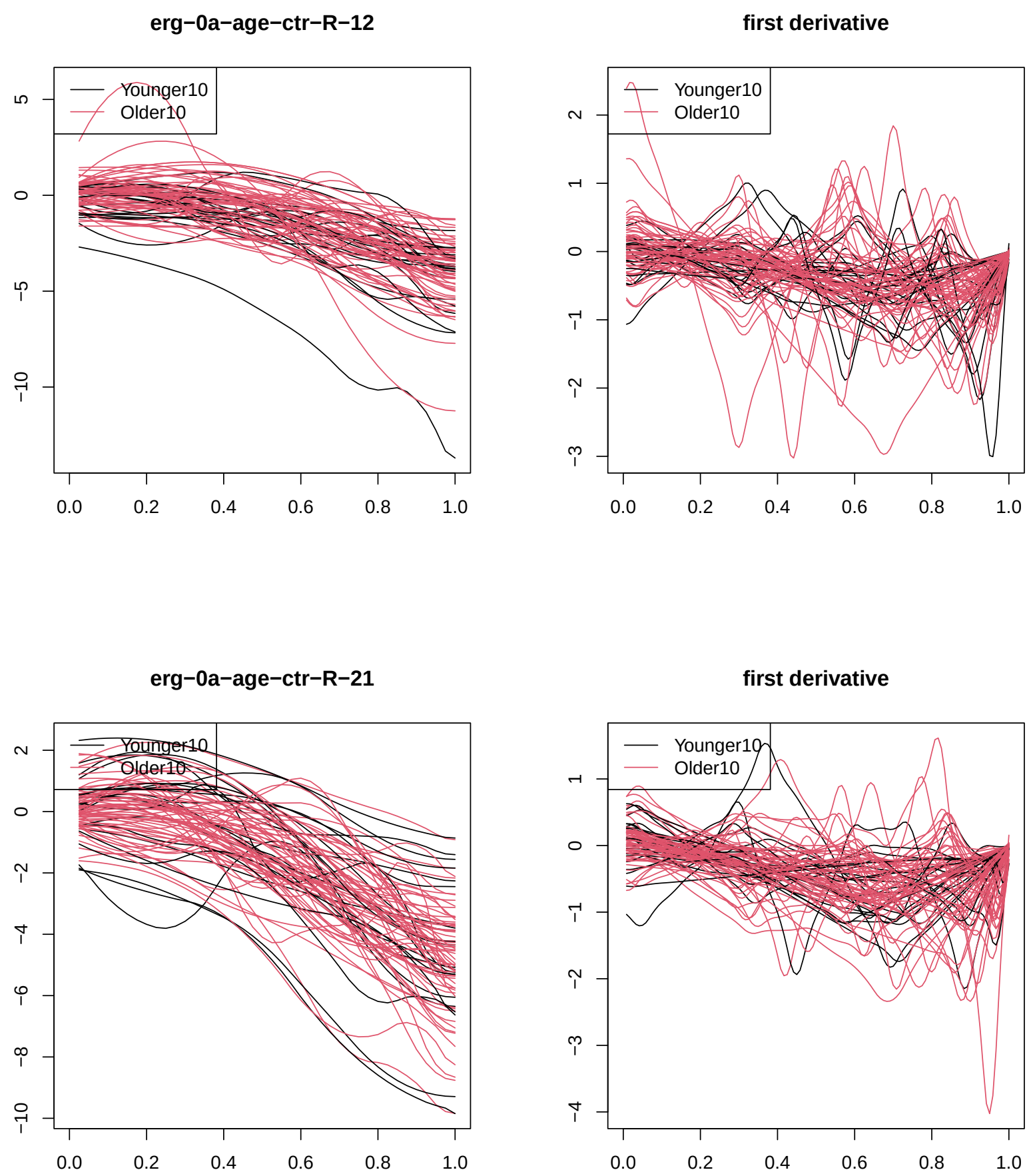

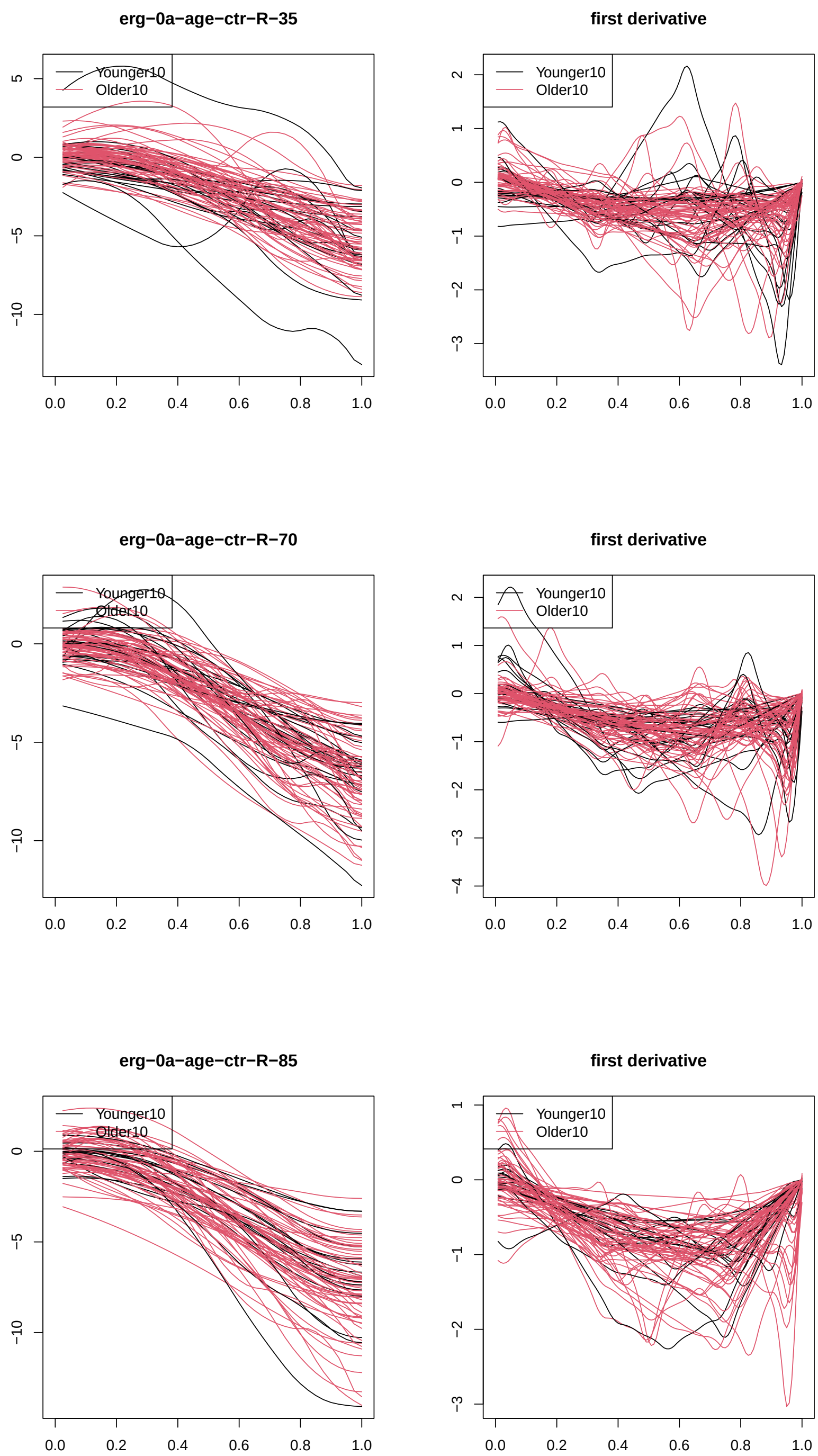
erg−0a−age−ctr−R−35
first derivative
erg−0a−age−ctr−R−70
first derivative
erg−0a−age−ctr−R−85
first derivative
Younger10
Older10
0.0
0.2
0.4
0.6
0.8
1.0
5
0
−5
−10
2
1
0
−1
−2
−3
−4

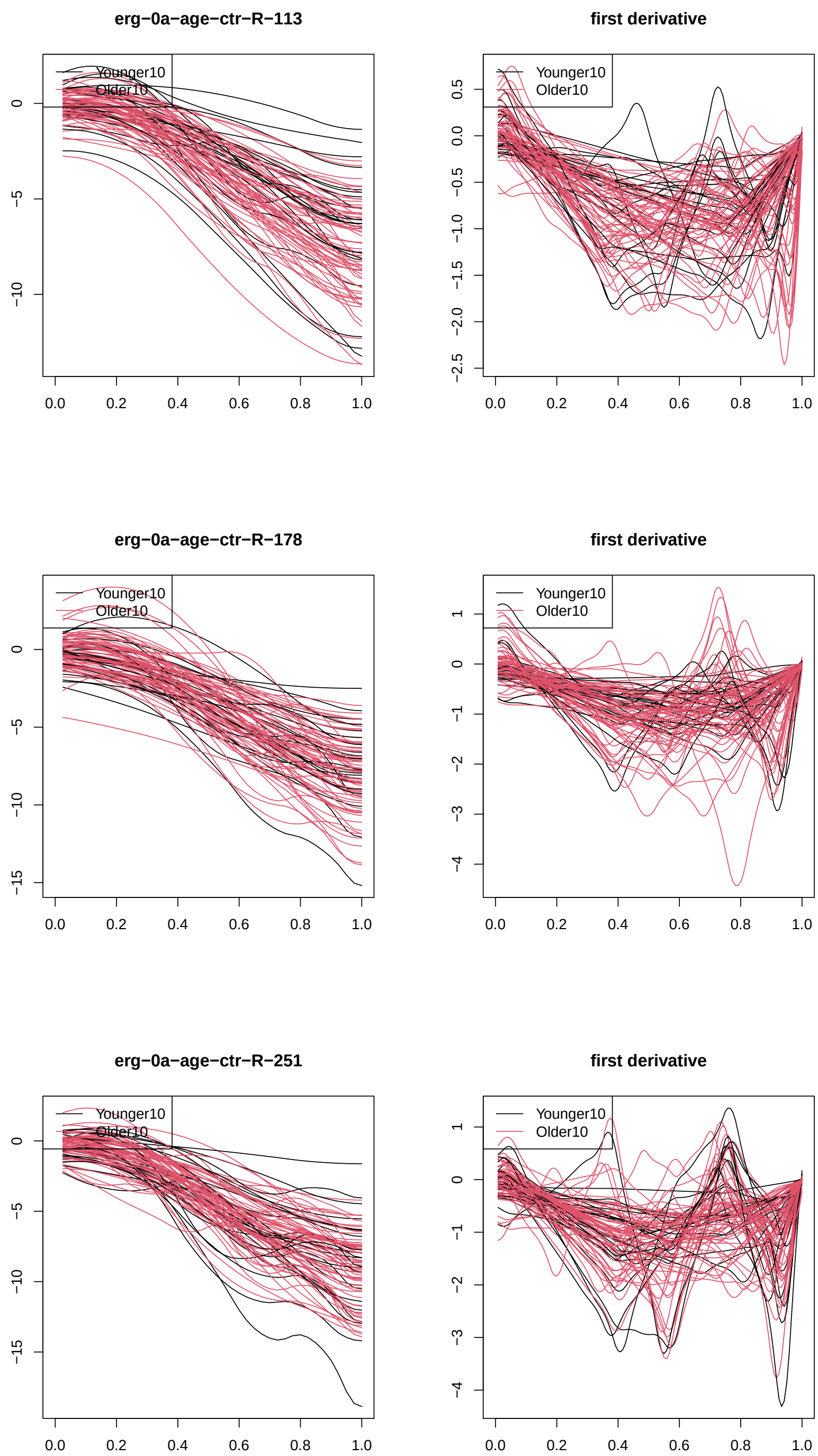
erg−0a−age−ctr−R−113
Younger10
Older10
first derivative
Younger10
Older10
erg−0a−age−ctr−R−178
Younger10
Older10
first derivative
Younger10
Older10
erg−0a−age−ctr−R−251
Younger10
Older10
first derivative
Younger10
Older10
0.0
0.2
0.4
0.6
0.8
1.0

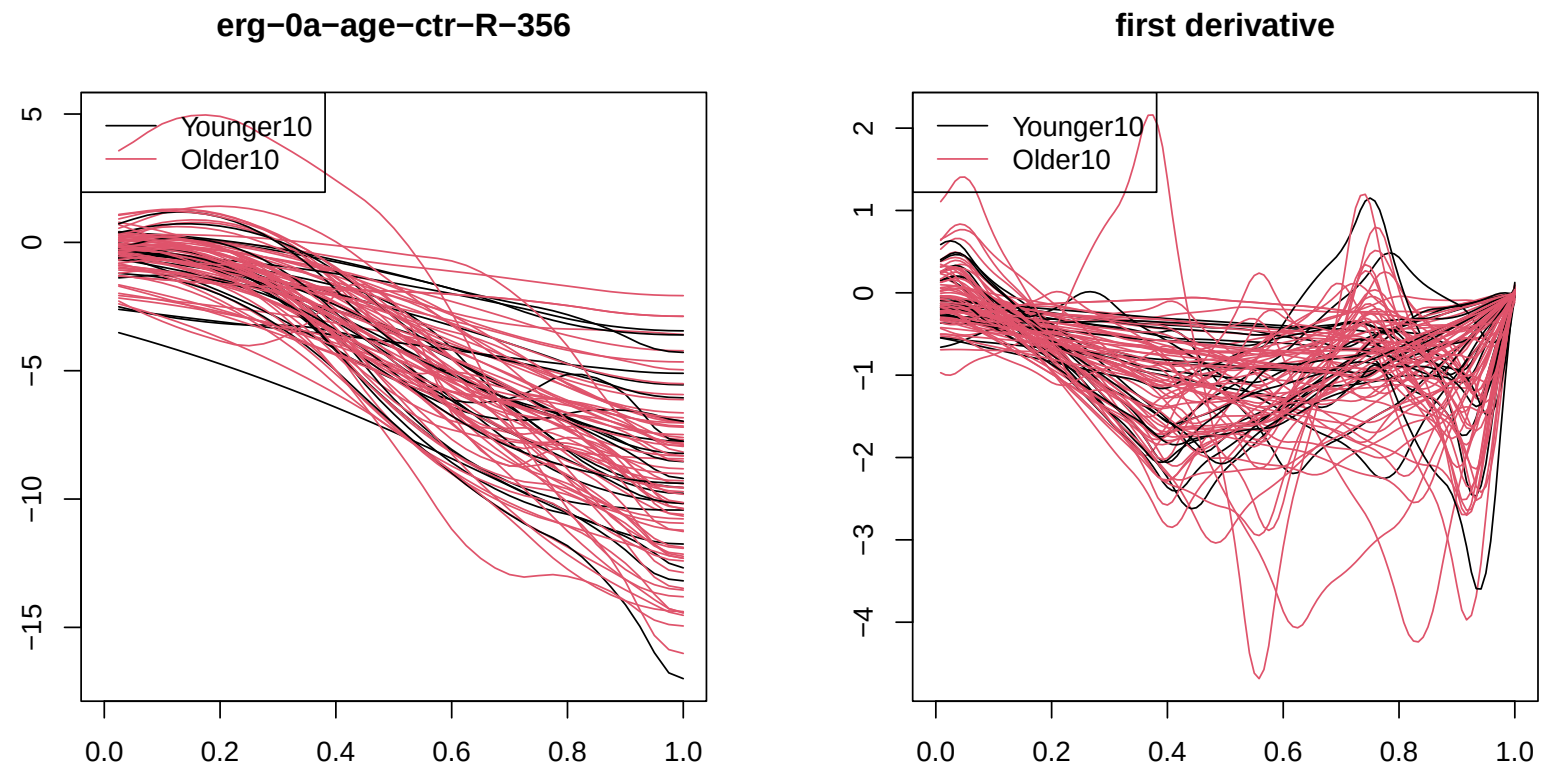
erg−0a−age−ctr−R−356
Younger10
Older10
first derivative
Younger10
Older10

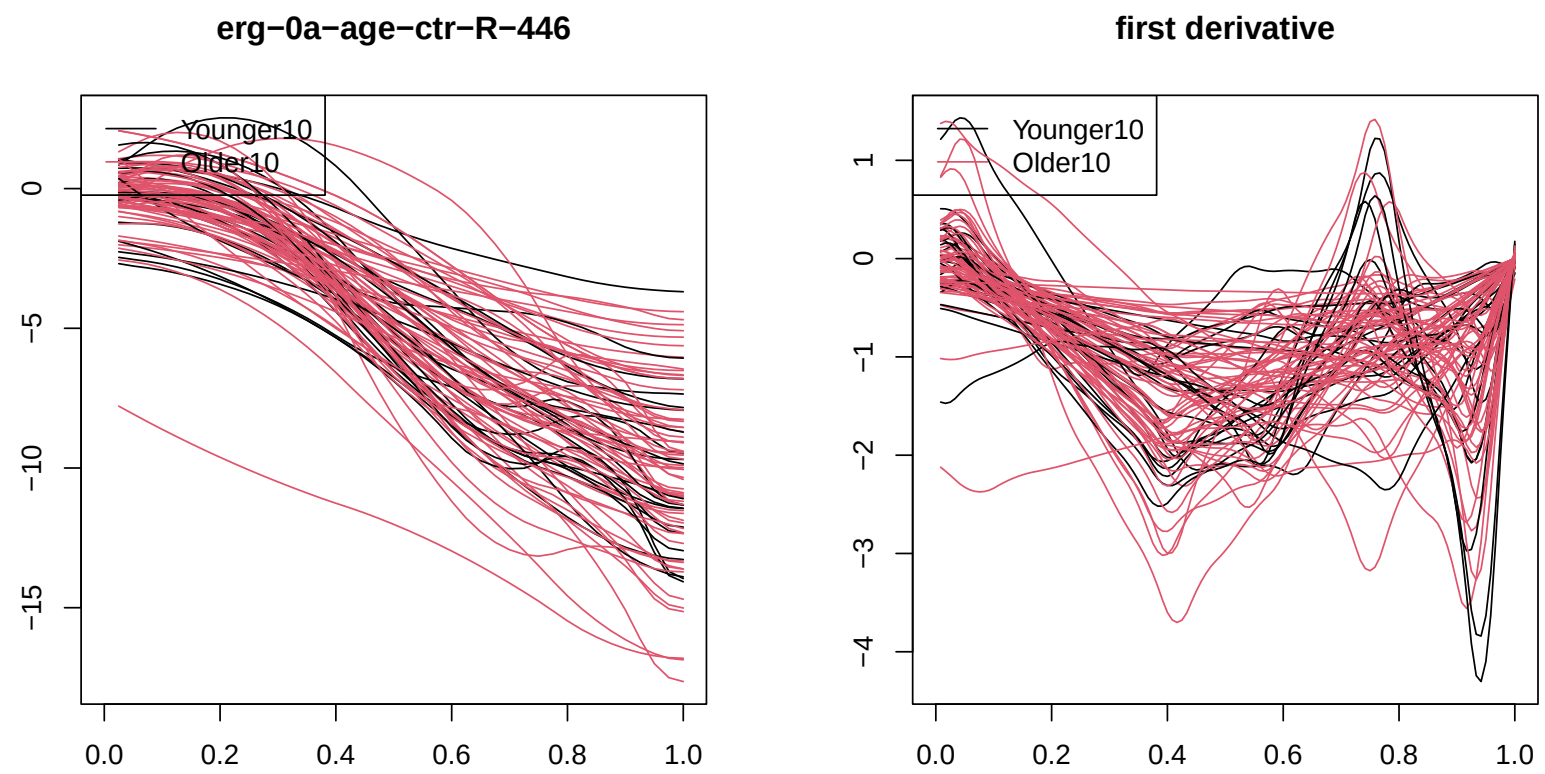
erg−0a−age−ctr−R−446
Younger10
Older10
first derivative
Younger10
Older10

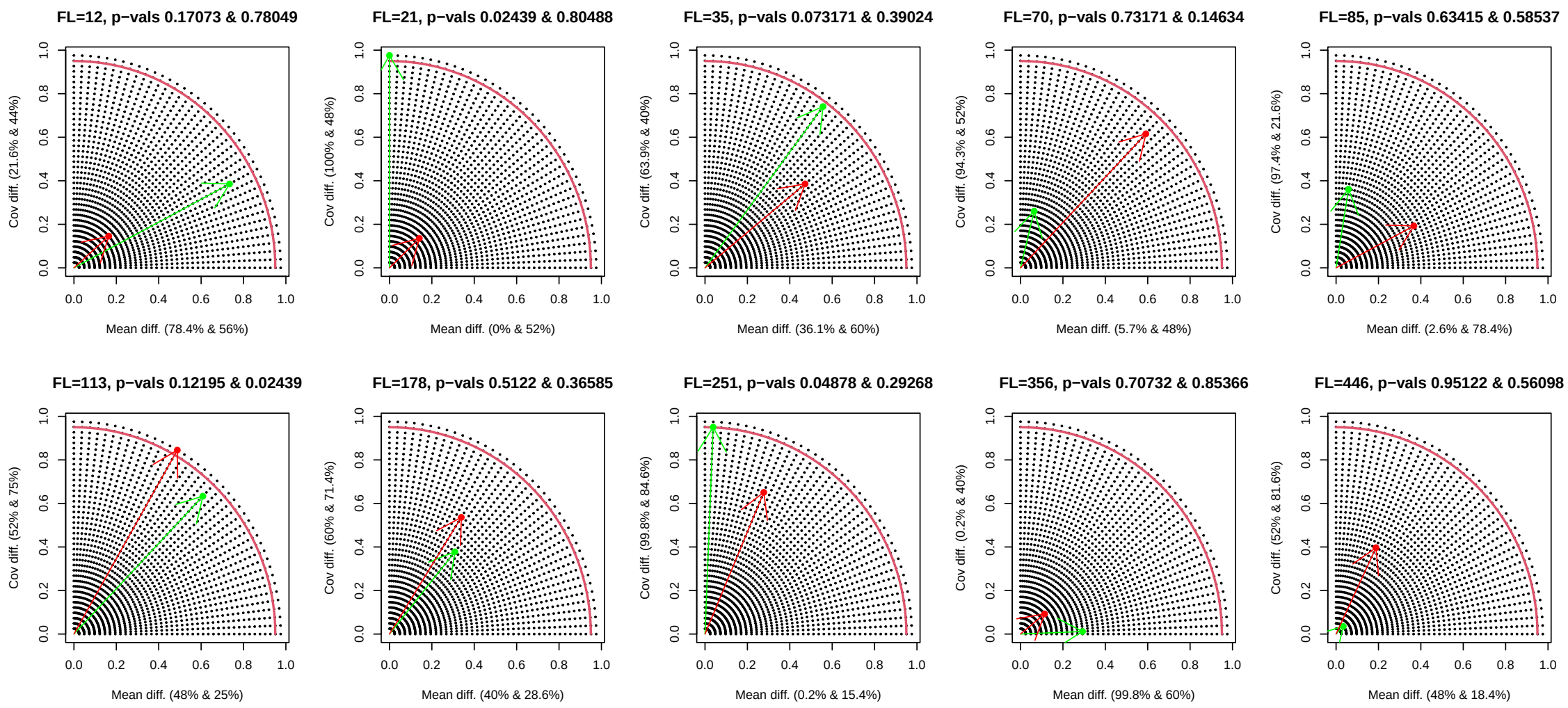
FL=12, p−vals 0.17073 & 0.78049
Cov diff. (21.6% & 44%)
Mean diff. (78.4% & 56%)
FL=21, p−vals 0.02439 & 0.80488
Cov diff. (100% & 48%)
Mean diff. (0% & 52%)
FL=35, p−vals 0.073171 & 0.39024
Cov diff. (63.9% & 40%)
Mean diff. (36.1% & 60%)
FL=70, p−vals 0.73171 & 0.14634
Cov diff. (94.3% & 52%)
Mean diff. (5.7% & 48%)
FL=85, p−vals 0.63415 & 0.58537
Cov diff. (97.4% & 21.6%)
Mean diff. (2.6% & 78.4%)
FL=113, p−vals 0.12195 & 0.02439
Cov diff. (52% & 75%)
Mean diff. (48% & 25%)
FL=178, p−vals 0.5122 & 0.36585
Cov diff. (60% & 71.4%)
Mean diff. (40% & 28.6%)
FL=251, p−vals 0.04878 & 0.29268
Cov diff. (99.8% & 84.6%)
Mean diff. (0.2% & 15.4%)
FL=356, p−vals 0.70732 & 0.85366
Cov diff. (0.2% & 40%)
Mean diff. (99.8% & 60%)
FL=446, p−vals 0.95122 & 0.56098
Cov diff. (52% & 81.6%)
Mean diff. (48% & 18.4%)

Table for ERG wave by age in 0–atime – ctr subgroup

	pval-erg-0a	mean perc	cov perc	pval-derivative	mean perc	cov perc
12	0.1707			0.7805		
21	0.0244	0	100	0.8049		
35	0.0732			0.3902		
70	0.7317			0.1463		
85	0.6341			0.5854		
113	0.1220			0.0244	25	75
178	0.5122			0.3659		
251	0.0488	0.2	99.8	0.2927		
356	0.7073			0.8537		
446	0.9512			0.5610		

2.16 ERG wave by age in 0–atime – asd subgroup

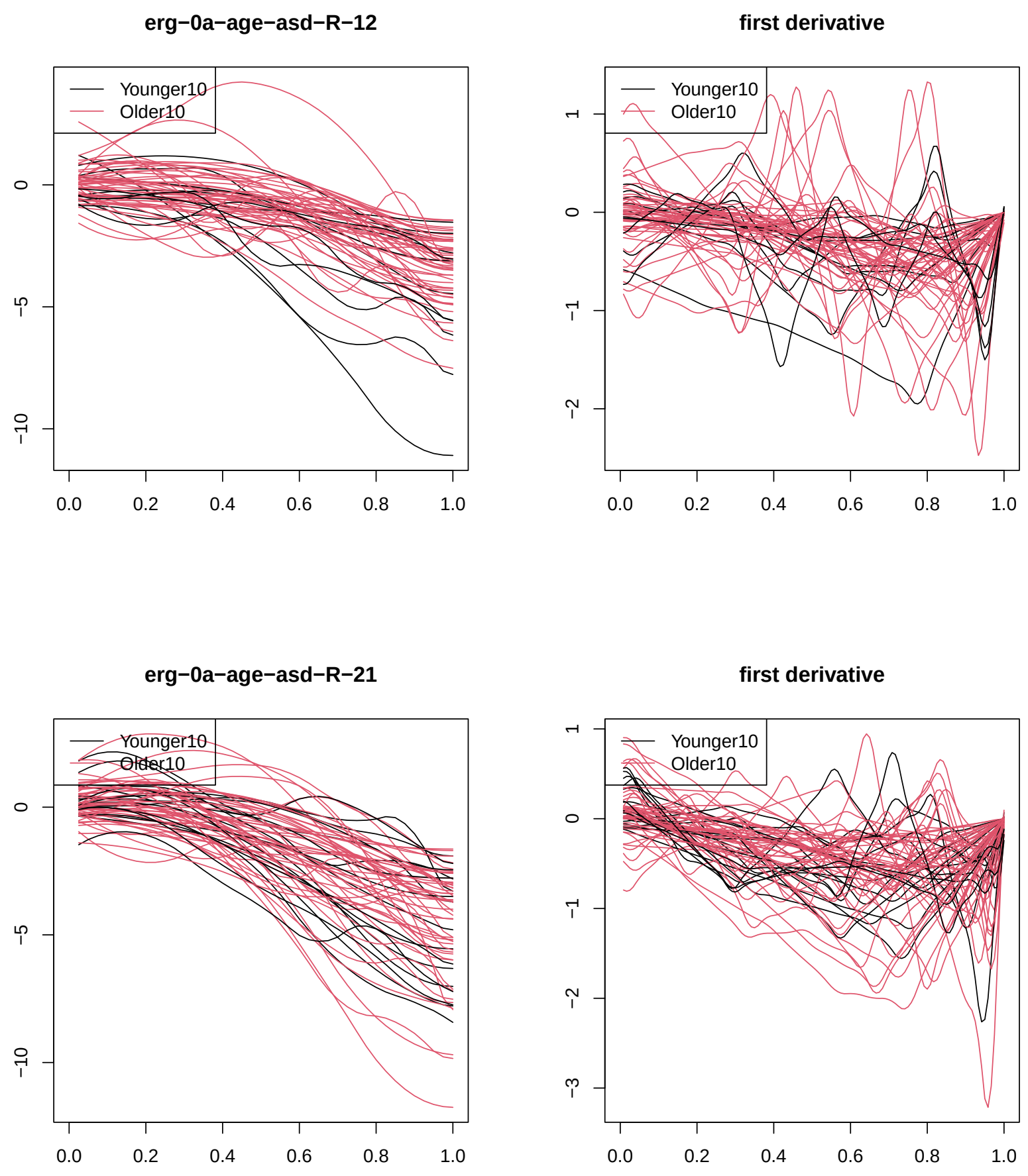

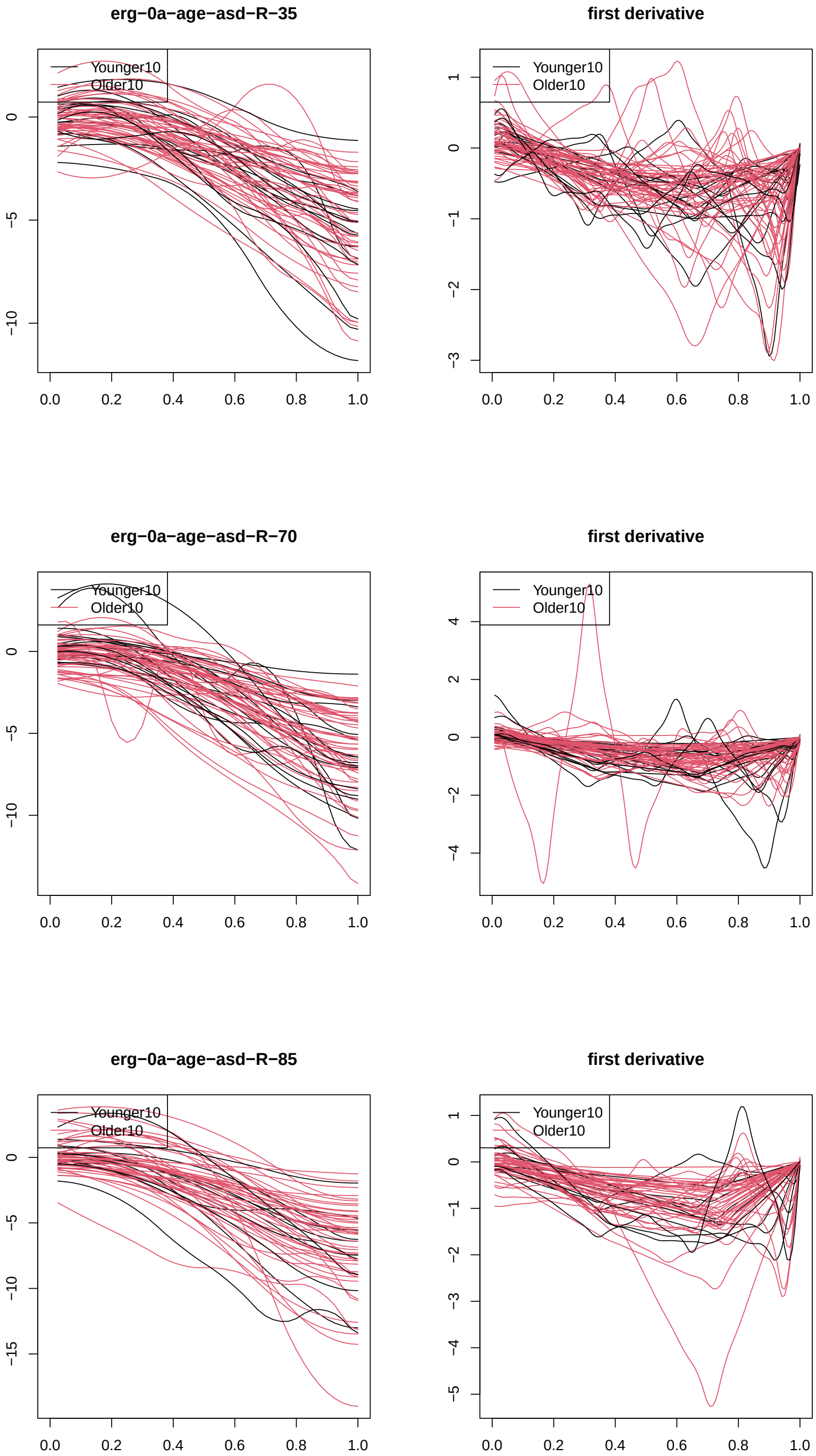
erg−0a−age−asd−R−35
Younger10
Older10
first derivative
Younger10
Older10
erg−0a−age−asd−R−70
Younger10
Older10
first derivative
Younger10
Older10
erg−0a−age−asd−R−85
Younger10
Older10
first derivative
Younger10
Older10

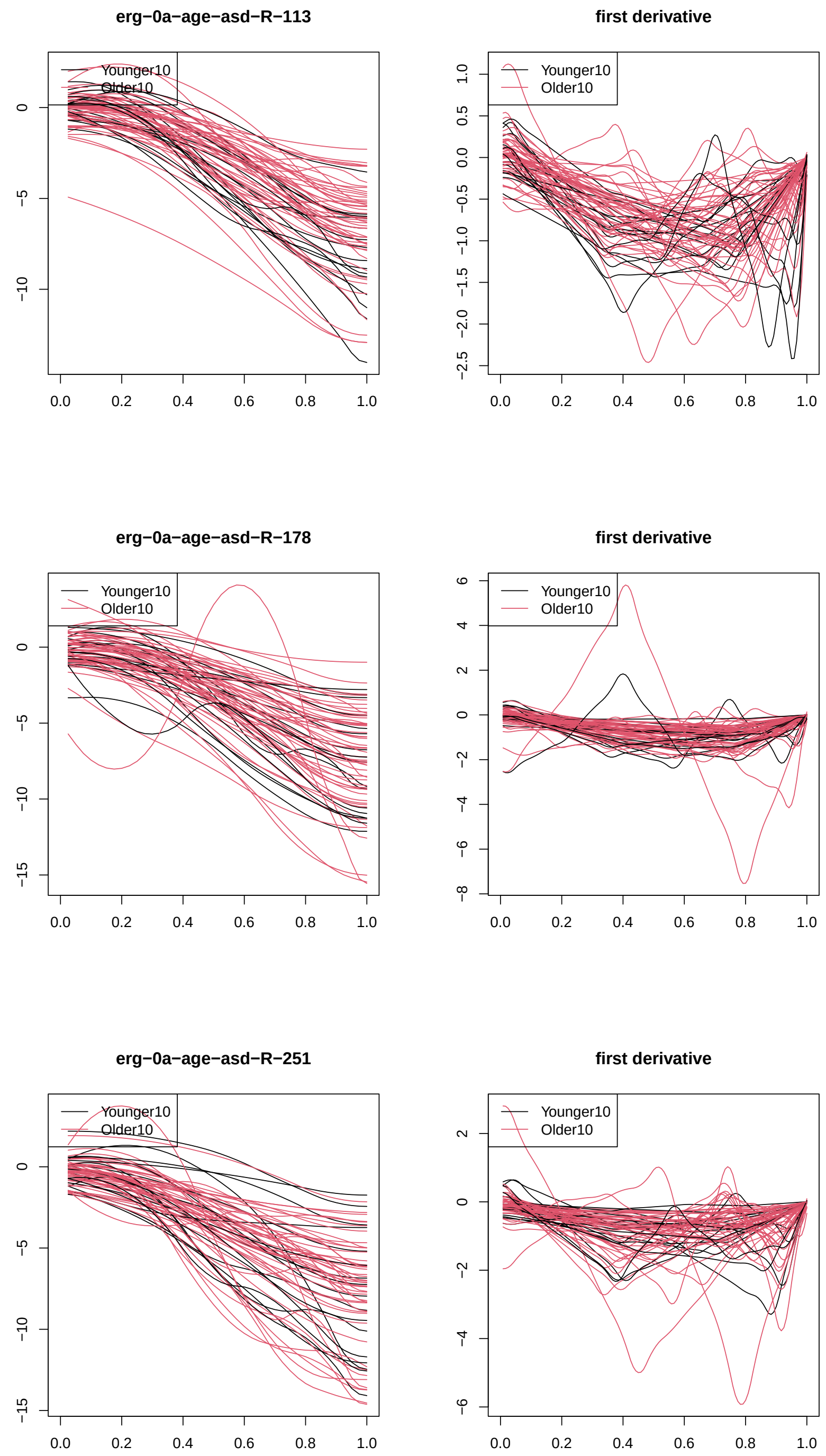
erg−0a−age−asd−R−113
first derivative
Younger10
Older10
erg−0a−age−asd−R−178
first derivative
Younger10
Older10
erg−0a−age−asd−R−251
first derivative
Younger10
Older10

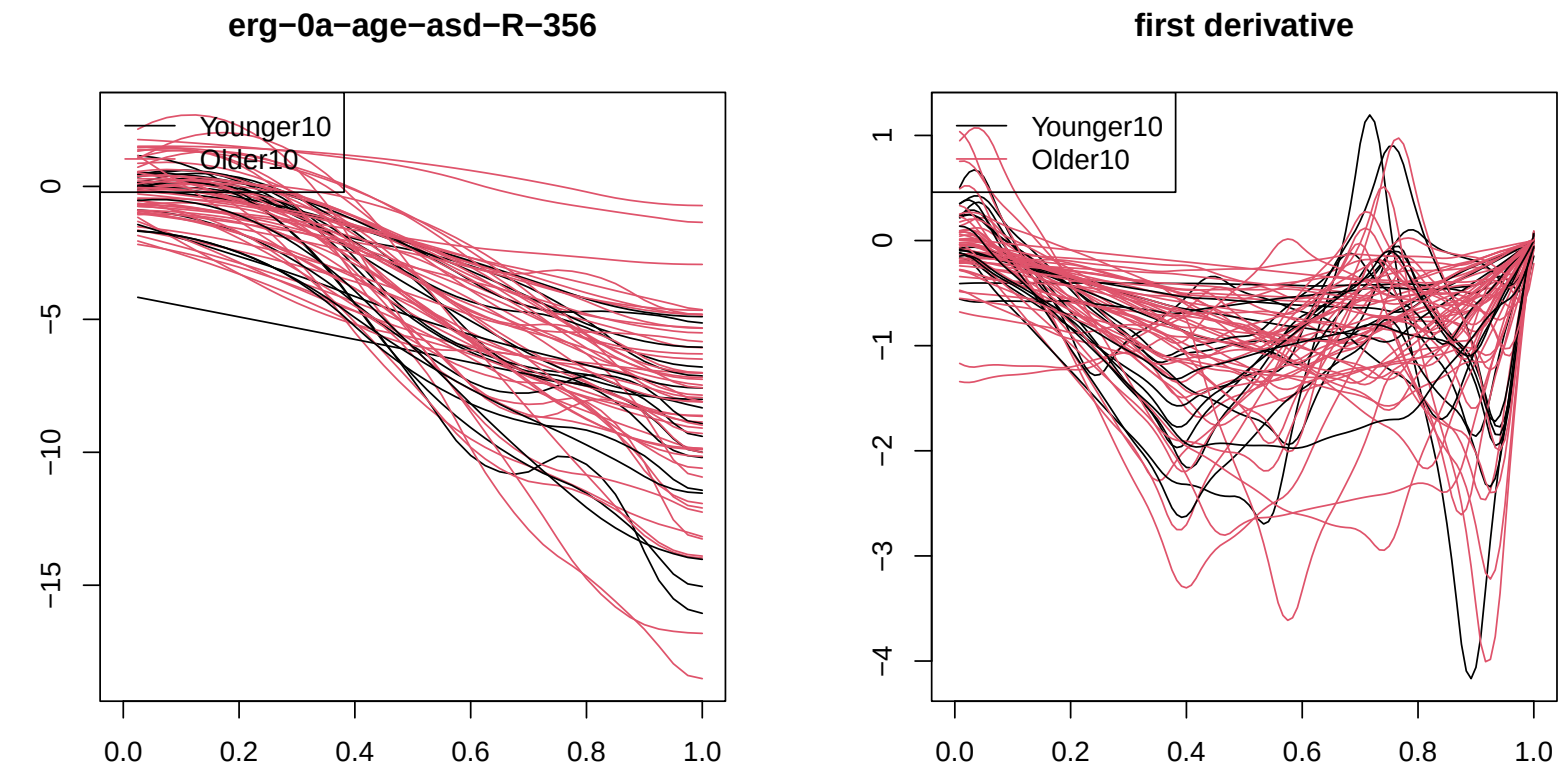
erg−0a−age−asd−R−356
first derivative
Younger10
Older10
Younger10
Older10

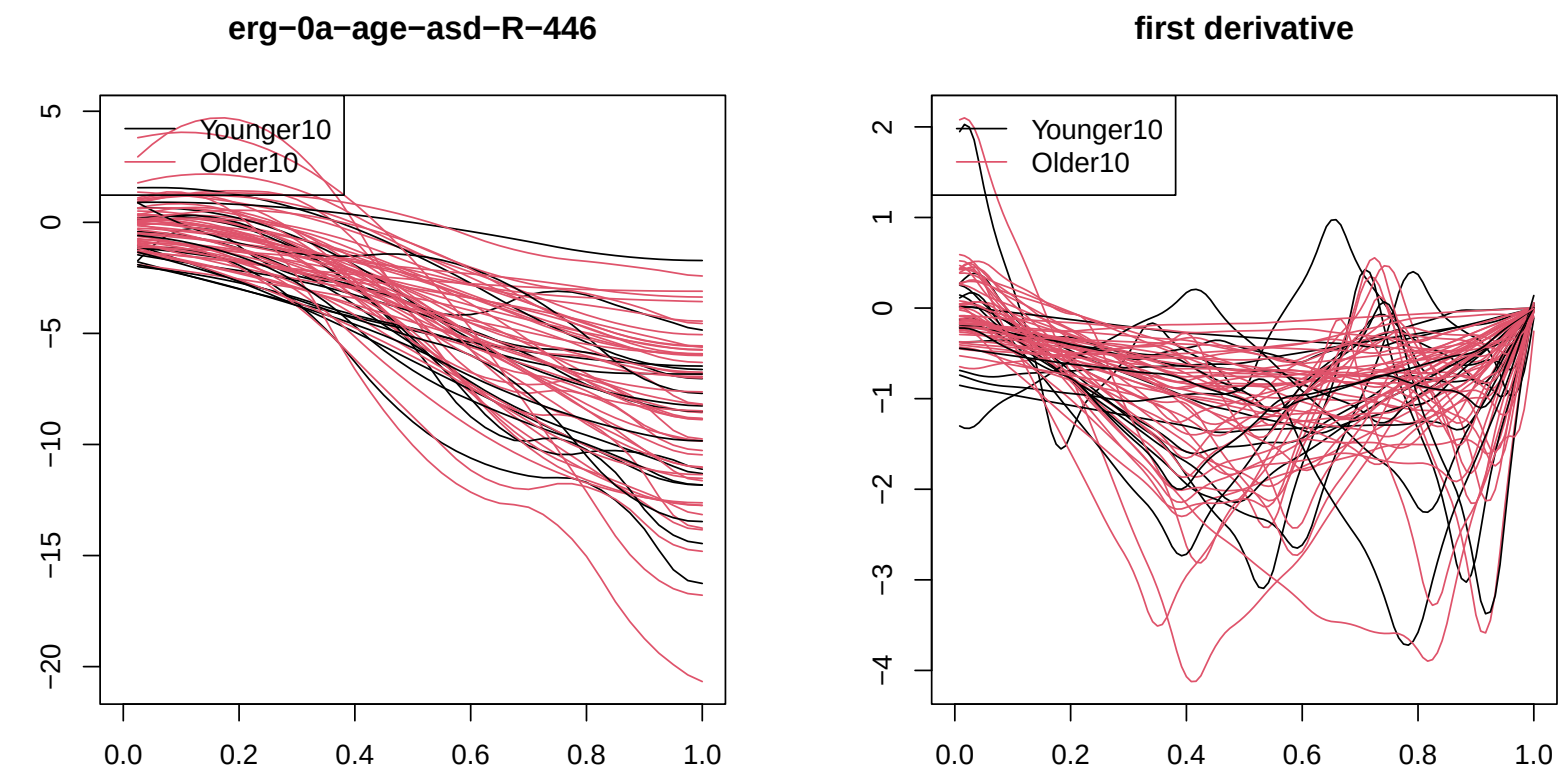
erg−0a−age−asd−R−446
first derivative
Younger10
Older10
Younger10
Older10

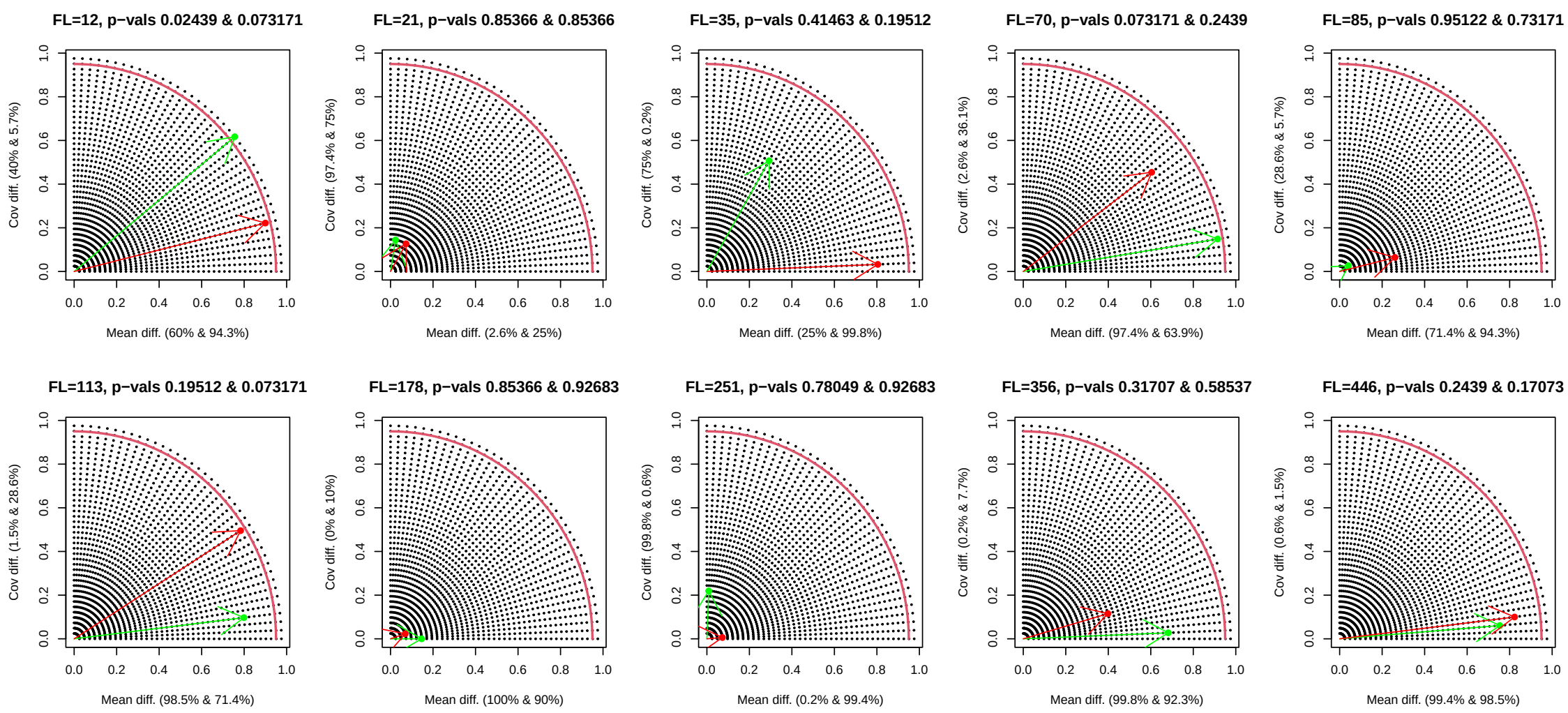
FL=12, p-vals 0.02439 & 0.073171
Cov diff. (40% & 5.7%)
Mean diff. (60% & 94.3%)
FL=21, p-vals 0.85366 & 0.85366
Cov diff. (97.4% & 75%)
Mean diff. (2.6% & 25%)
FL=35, p-vals 0.41463 & 0.19512
Cov diff. (75% & 0.2%)
Mean diff. (25% & 99.8%)
FL=70, p-vals 0.073171 & 0.2439
Cov diff. (2.6% & 36.1%)
Mean diff. (97.4% & 63.9%)
FL=85, p-vals 0.95122 & 0.73171
Cov diff. (28.6% & 5.7%)
Mean diff. (71.4% & 94.3%)
FL=113, p-vals 0.19512 & 0.073171
Cov diff. (1.5% & 28.6%)
Mean diff. (98.5% & 71.4%)
FL=178, p-vals 0.85366 & 0.92683
Cov diff. (0% & 10%)
Mean diff. (100% & 90%)
FL=251, p-vals 0.78049 & 0.92683
Cov diff. (99.8% & 0.6%)
Mean diff. (0.2% & 99.4%)
FL=356, p-vals 0.31707 & 0.58537
Cov diff. (0.2% & 7.7%)
Mean diff. (99.8% & 92.3%)
FL=446, p-vals 0.2439 & 0.17073
Cov diff. (0.6% & 1.5%)
Mean diff. (99.4% & 98.5%)

Table for ERG wave by age in 0–atime – asd subgroup

	pval-erg-0a	mean perc	cov perc	pval-derivative	mean perc	cov perc
12	0.0244	60	40	0.0732		
21	0.8537			0.8537		
35	0.4146			0.1951		
70	0.0732			0.2439		
85	0.9512			0.7317		
113	0.1951			0.0732		
178	0.8537			0.9268		
251	0.7805			0.9268		
356	0.3171			0.5854		
446	0.2439			0.1707		

2.17 ERG wave by age in atime–btime – ctr subgroup

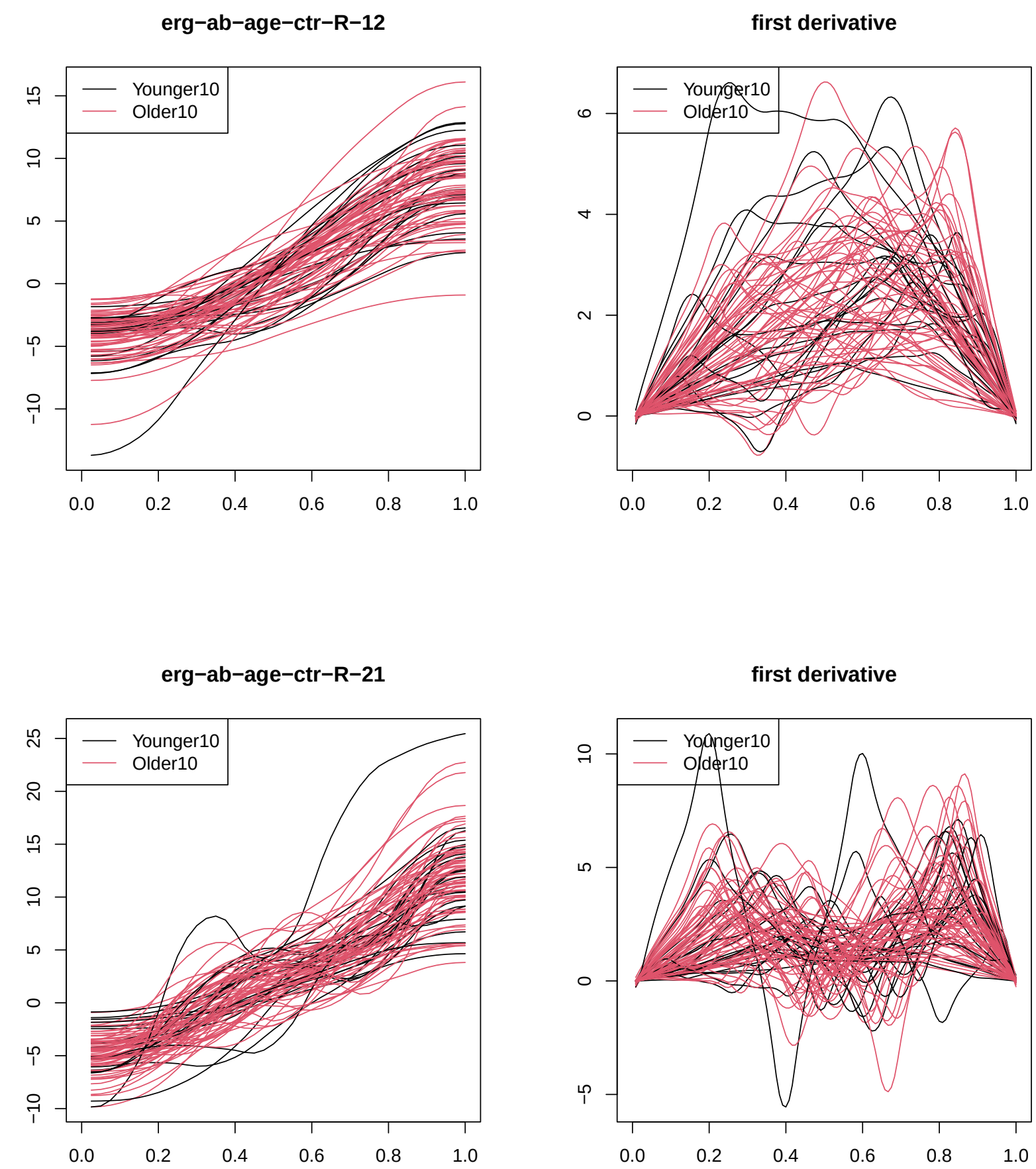

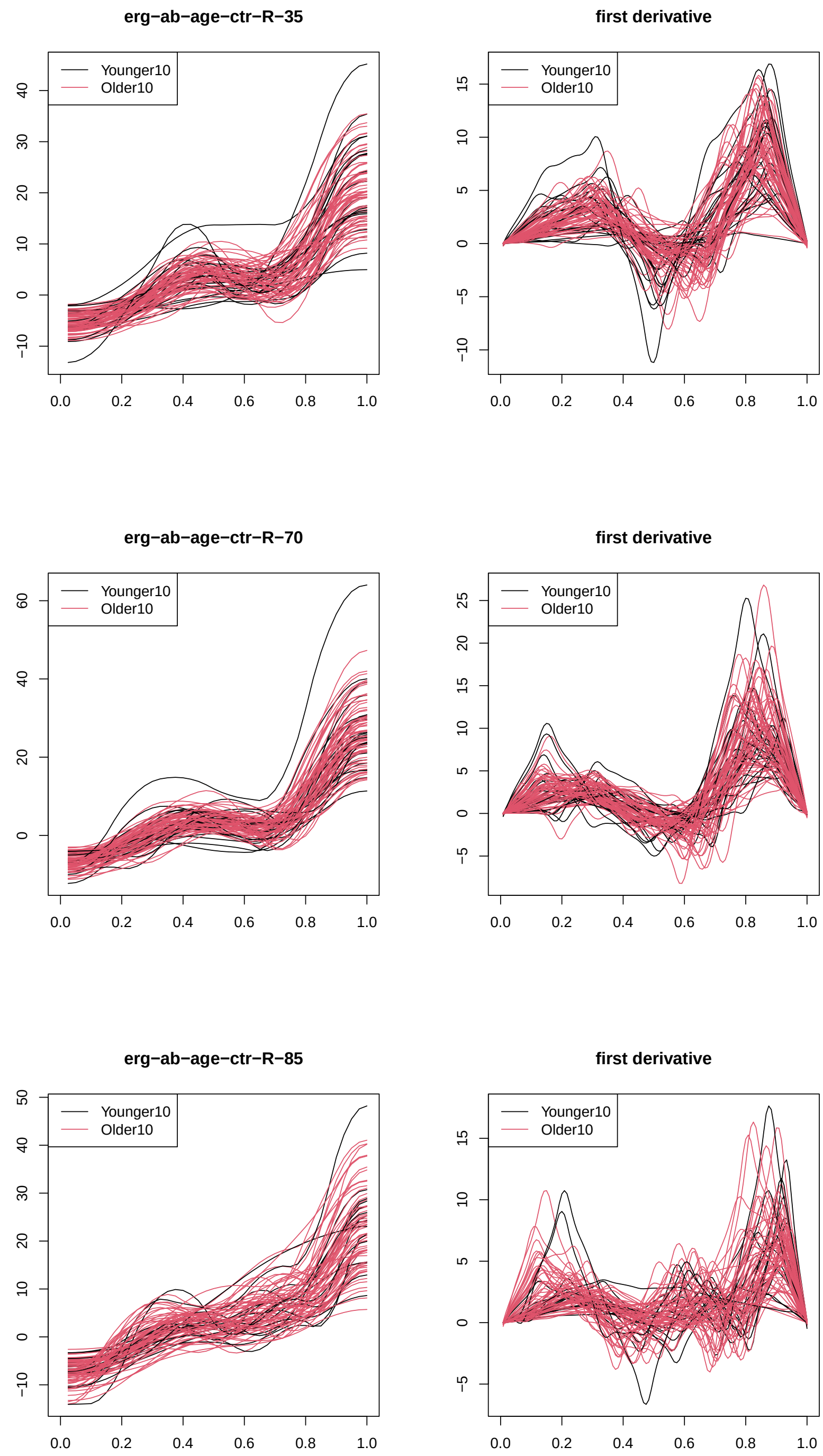
erg−ab−age−ctr−R−35
first derivative
erg−ab−age−ctr−R−70
first derivative
erg−ab−age−ctr−R−85
first derivative
Younger10
Older10

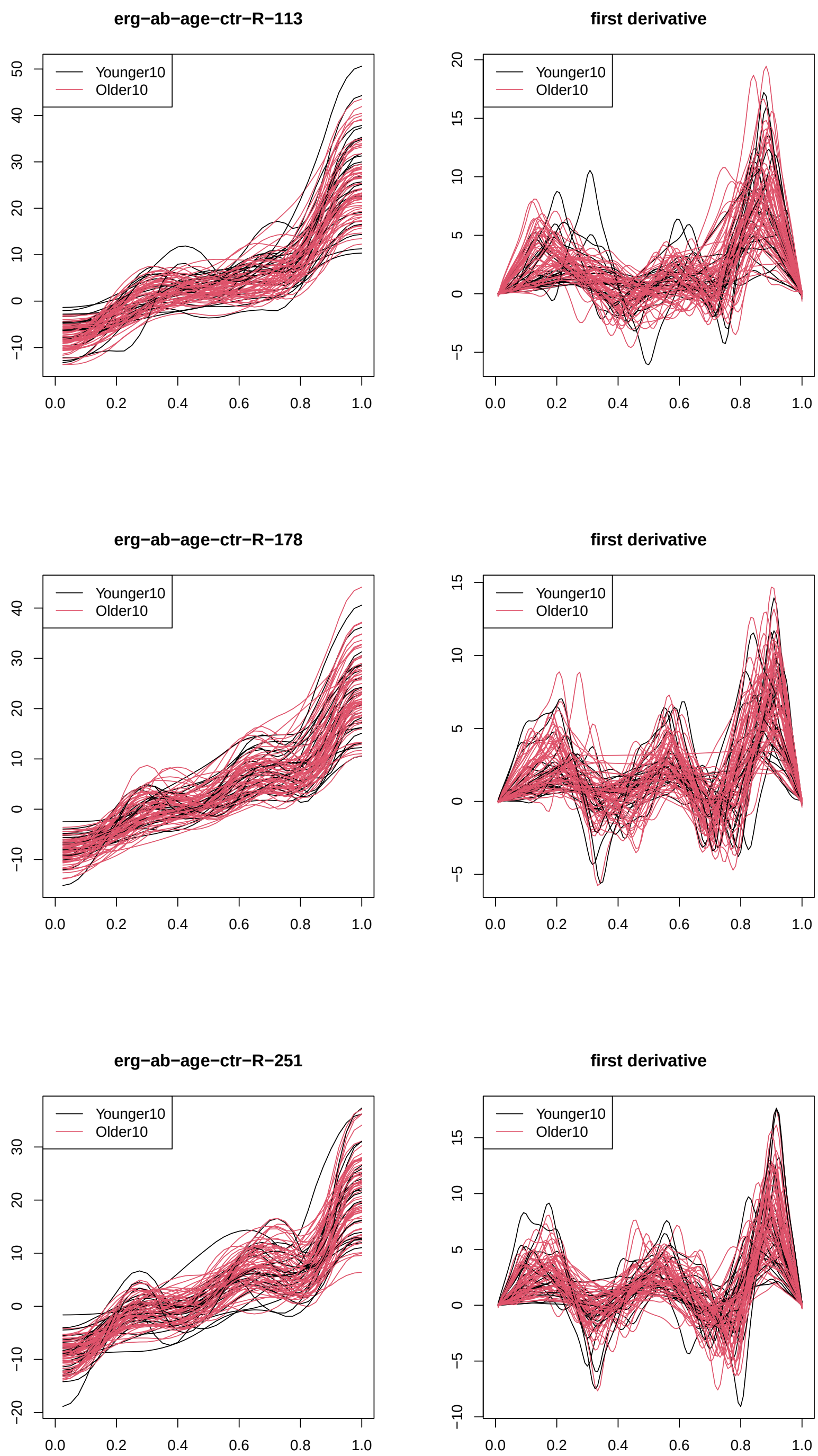
erg−ab−age−ctr−R−113
first derivative
erg−ab−age−ctr−R−178
first derivative
erg−ab−age−ctr−R−251
first derivative
Younger10
Older10

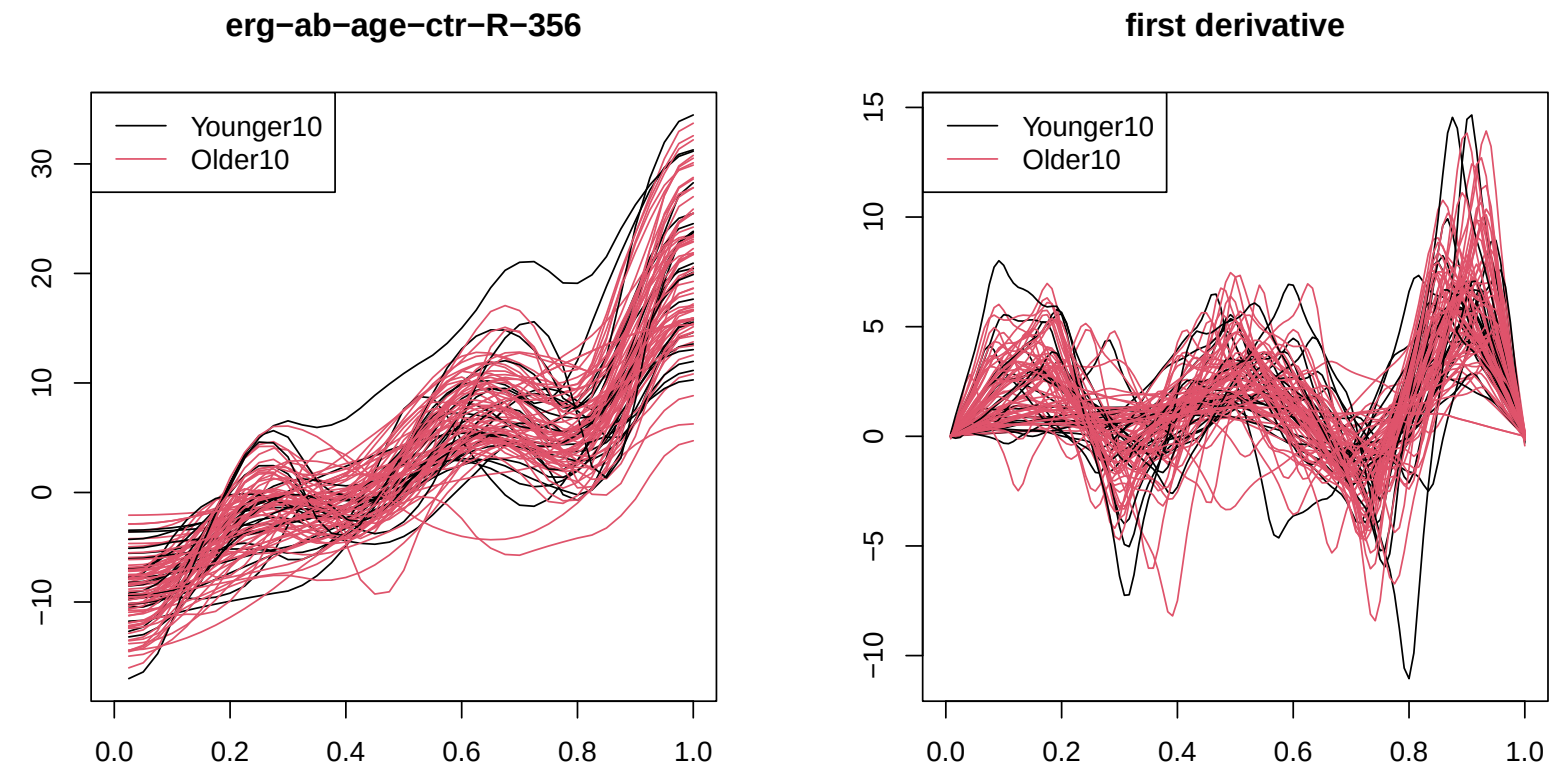
erg−ab−age−ctr−R−356
Younger10
Older10
first derivative
Younger10
Older10

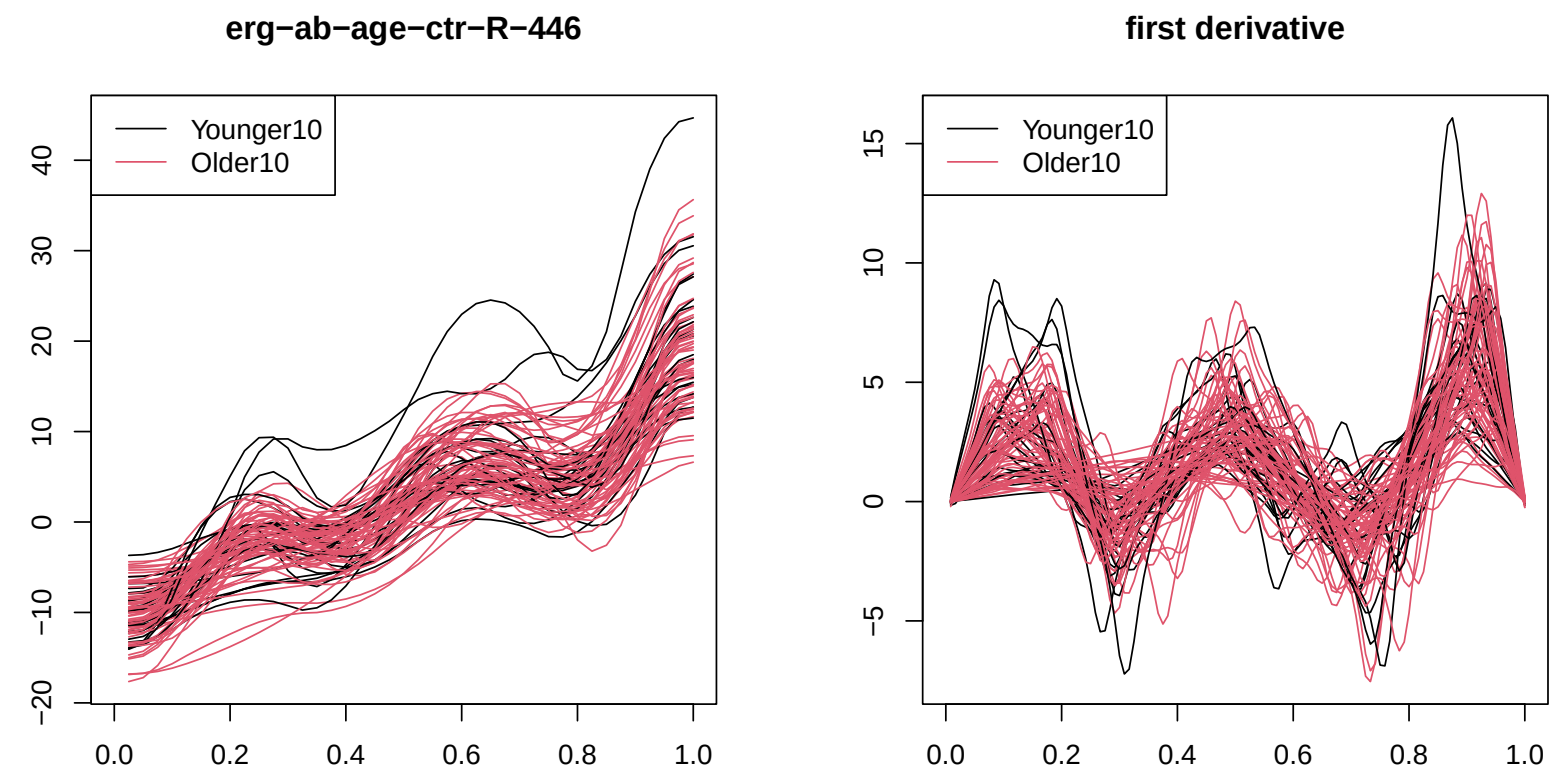
erg−ab−age−ctr−R−446
Younger10
Older10
first derivative
Younger10
Older10

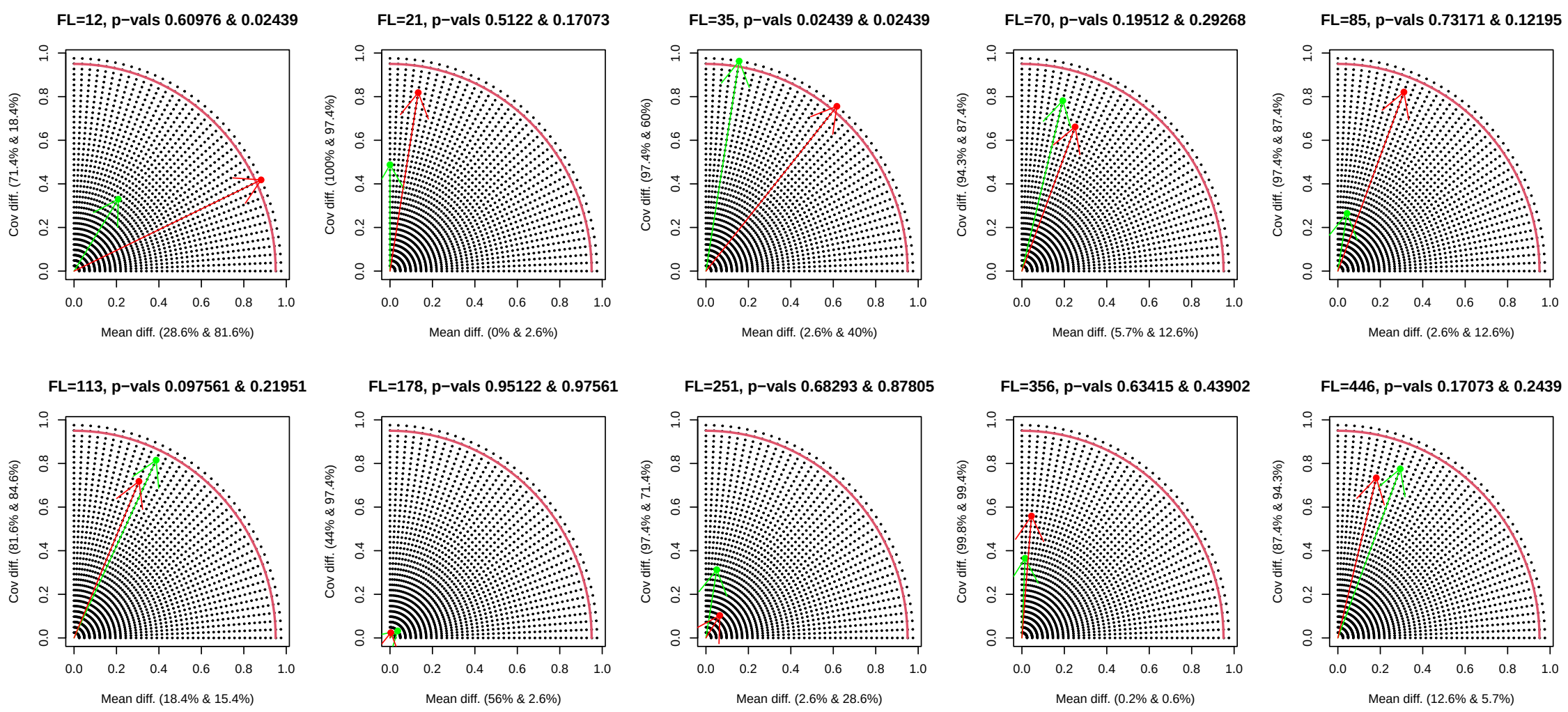
FL=12, p−vals 0.60976 & 0.02439
Cov diff. (71.4% & 18.4%)
Mean diff. (28.6% & 81.6%)
FL=21, p−vals 0.5122 & 0.17073
Cov diff. (100% & 97.4%)
Mean diff. (0% & 2.6%)
FL=35, p−vals 0.02439 & 0.02439
Cov diff. (97.4% & 60%)
Mean diff. (2.6% & 40%)
FL=70, p−vals 0.19512 & 0.29268
Cov diff. (94.3% & 87.4%)
Mean diff. (5.7% & 12.6%)
FL=85, p−vals 0.73171 & 0.12195
Cov diff. (97.4% & 87.4%)
Mean diff. (2.6% & 12.6%)
FL=113, p−vals 0.097561 & 0.21951
Cov diff. (81.6% & 84.6%)
Mean diff. (18.4% & 15.4%)
FL=178, p−vals 0.95122 & 0.97561
Cov diff. (44% & 97.4%)
Mean diff. (56% & 2.6%)
FL=251, p−vals 0.68293 & 0.87805
Cov diff. (97.4% & 71.4%)
Mean diff. (2.6% & 28.6%)
FL=356, p−vals 0.63415 & 0.43902
Cov diff. (99.8% & 99.4%)
Mean diff. (0.2% & 0.6%)
FL=446, p−vals 0.17073 & 0.2439
Cov diff. (87.4% & 94.3%)
Mean diff. (12.6% & 5.7%)

Table for ERG wave by age in atime–btime – ctr subgroup

	pval-erg-ab	mean perc	cov perc	pval-derivative	mean perc	cov perc
12	0.6098			0.0244	81.6	18.4
21	0.5122			0.1707		
35	0.0244	2.6	97.4	0.0244	40	60
70	0.1951			0.2927		
85	0.7317			0.1220		
113	0.0976			0.2195		
178	0.9512			0.9756		
251	0.6829			0.8780		
356	0.6341			0.4390		
446	0.1707			0.2439		

2.18 ERG wave by age in atime–btime – asd subgroup

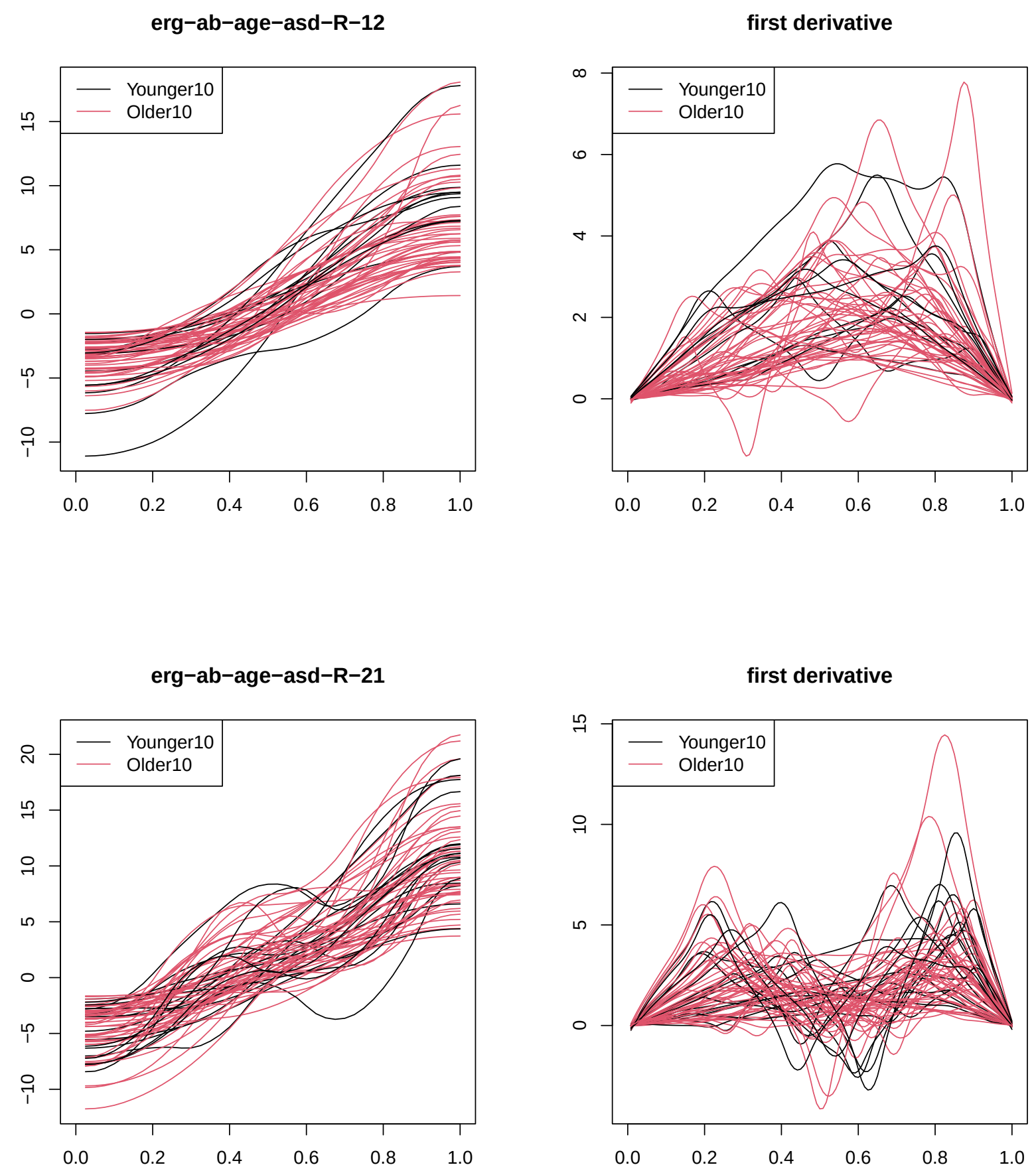

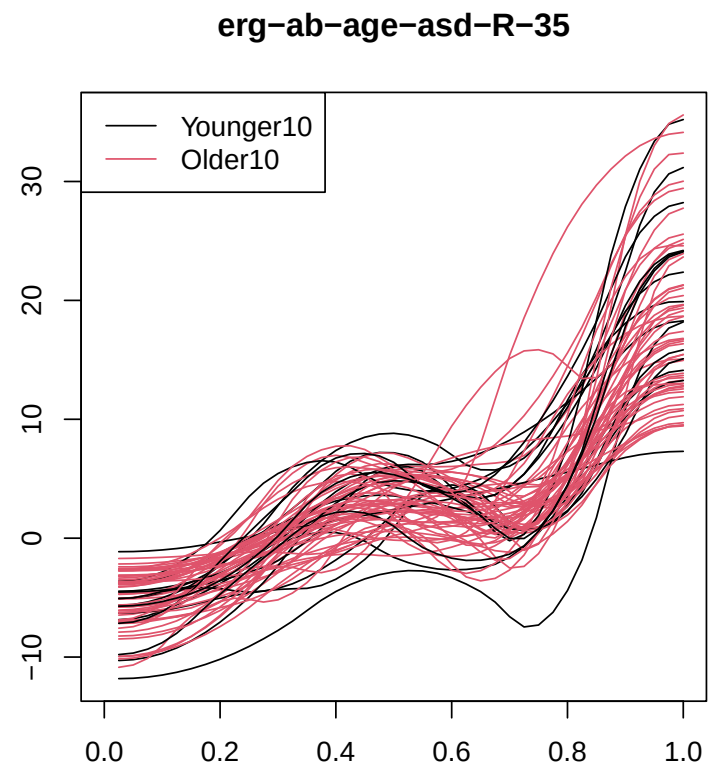
erg−ab−age−asd−R−35
Younger10
Older10

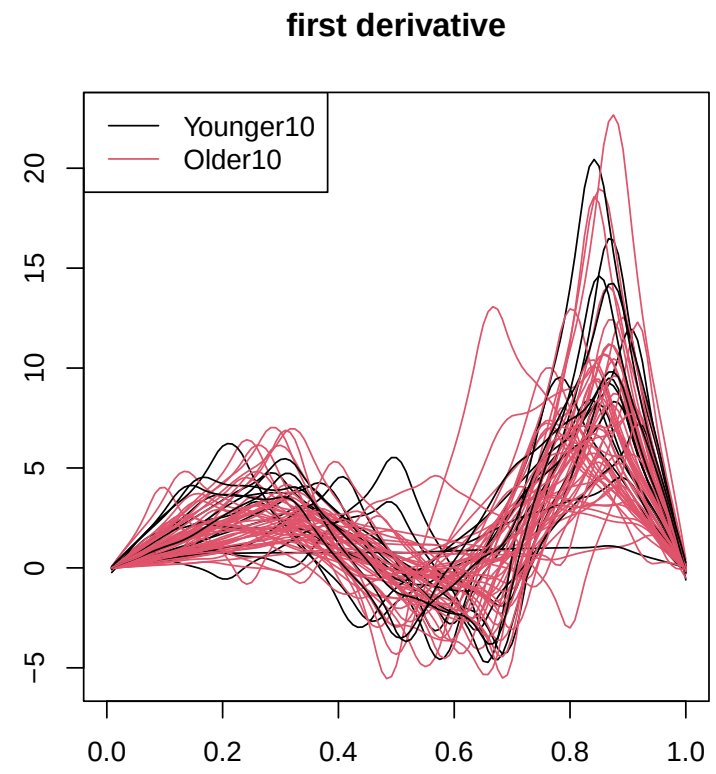
first derivative
Younger10
Older10

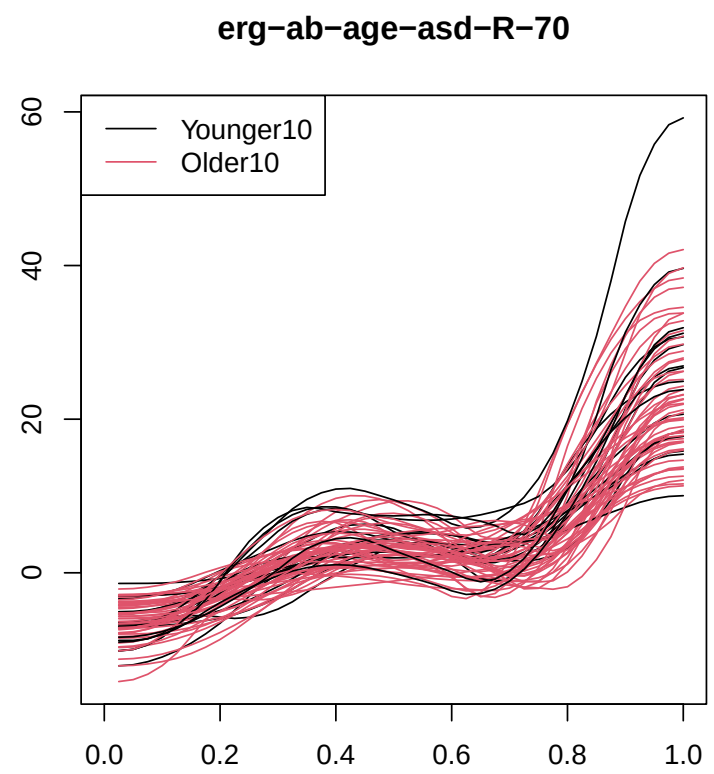
erg−ab−age−asd−R−70
Younger10
Older10

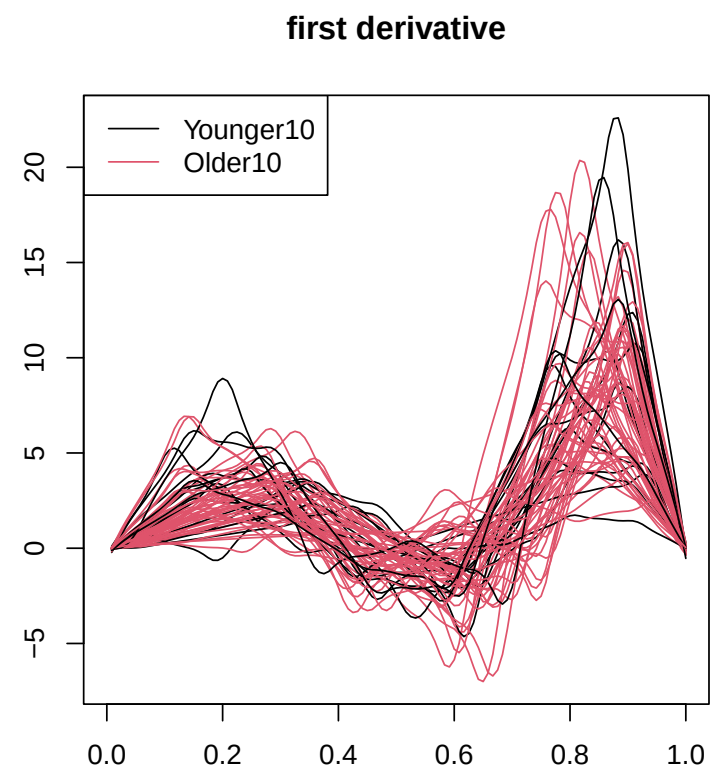
first derivative
Younger10
Older10

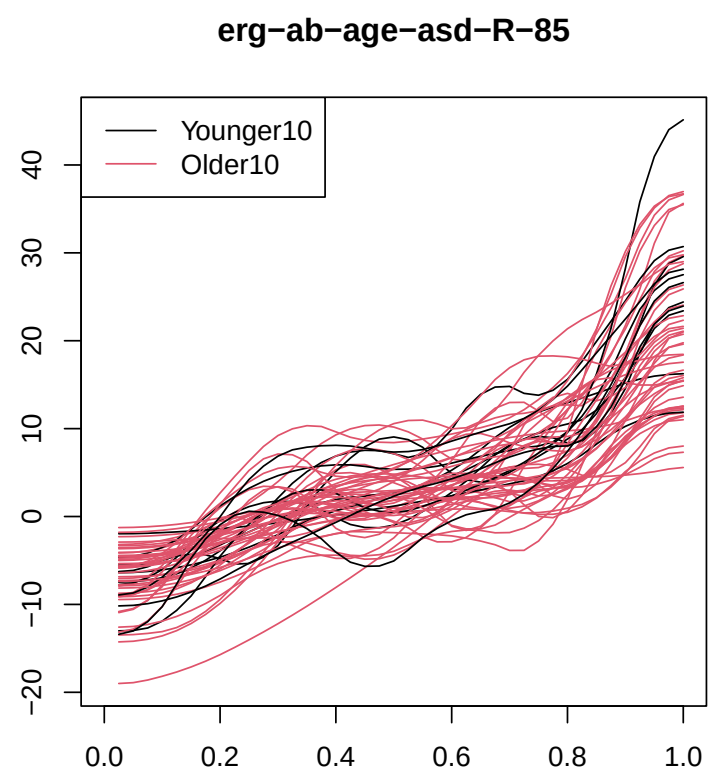
erg−ab−age−asd−R−85
Younger10
Older10

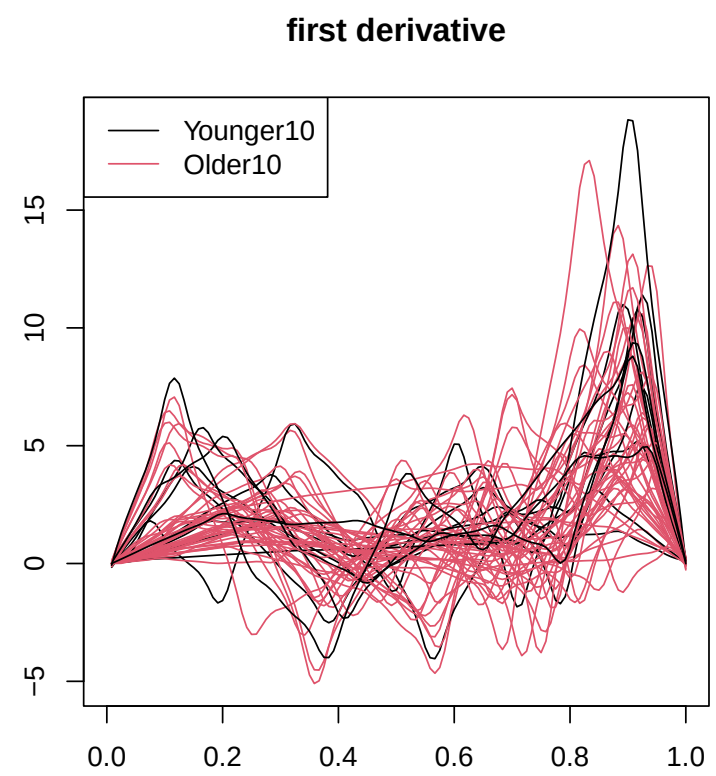
first derivative
Younger10
Older10

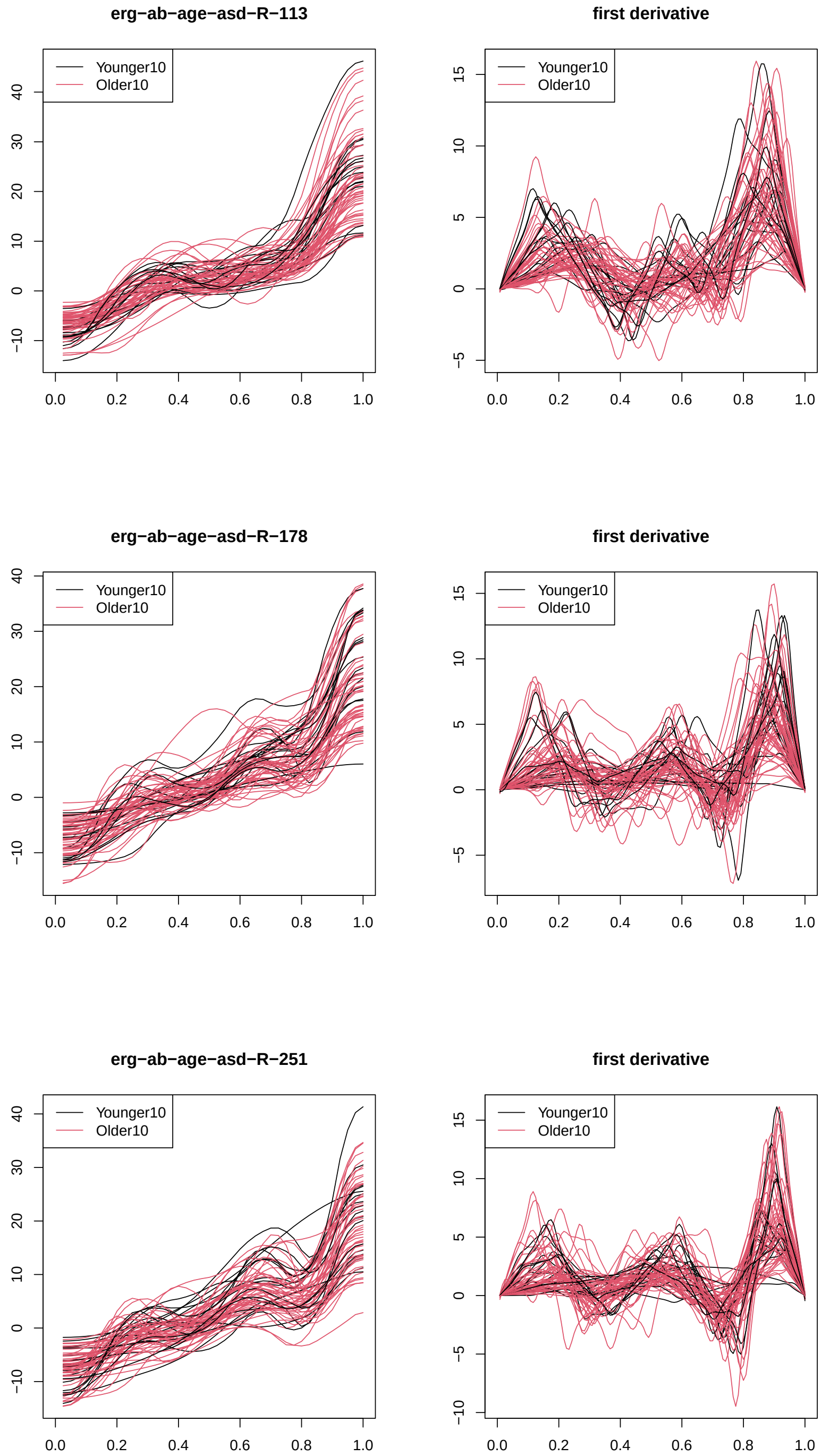

erg−ab−age−asd−R−113
first derivative
Younger10
Older10
erg−ab−age−asd−R−178
first derivative
Younger10
Older10
erg−ab−age−asd−R−251
first derivative
Younger10
Older10
0.0
0.2
0.4
0.6
0.8
1.0

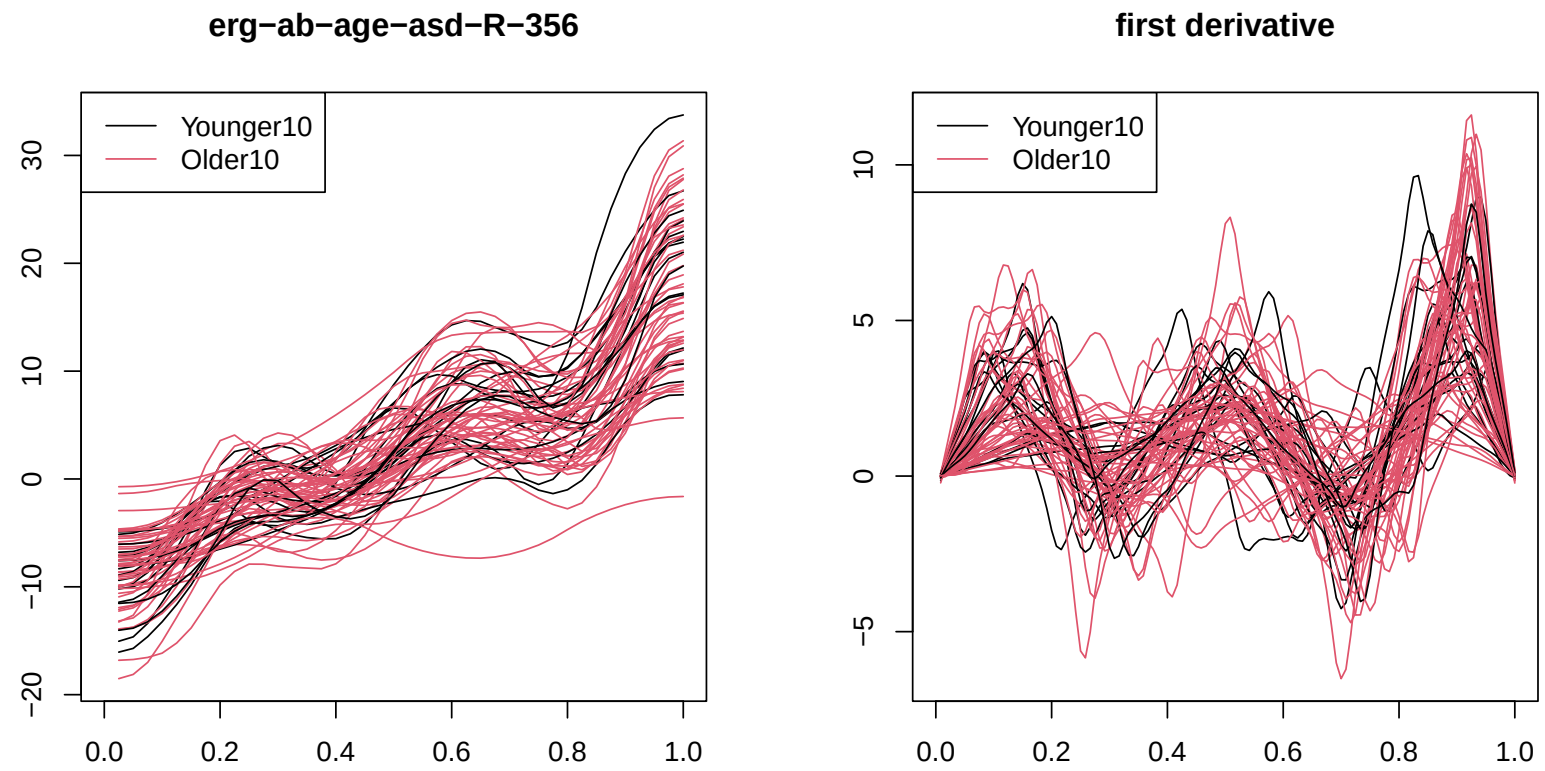
erg−ab−age−asd−R−356
Younger10
Older10
first derivative
Younger10
Older10

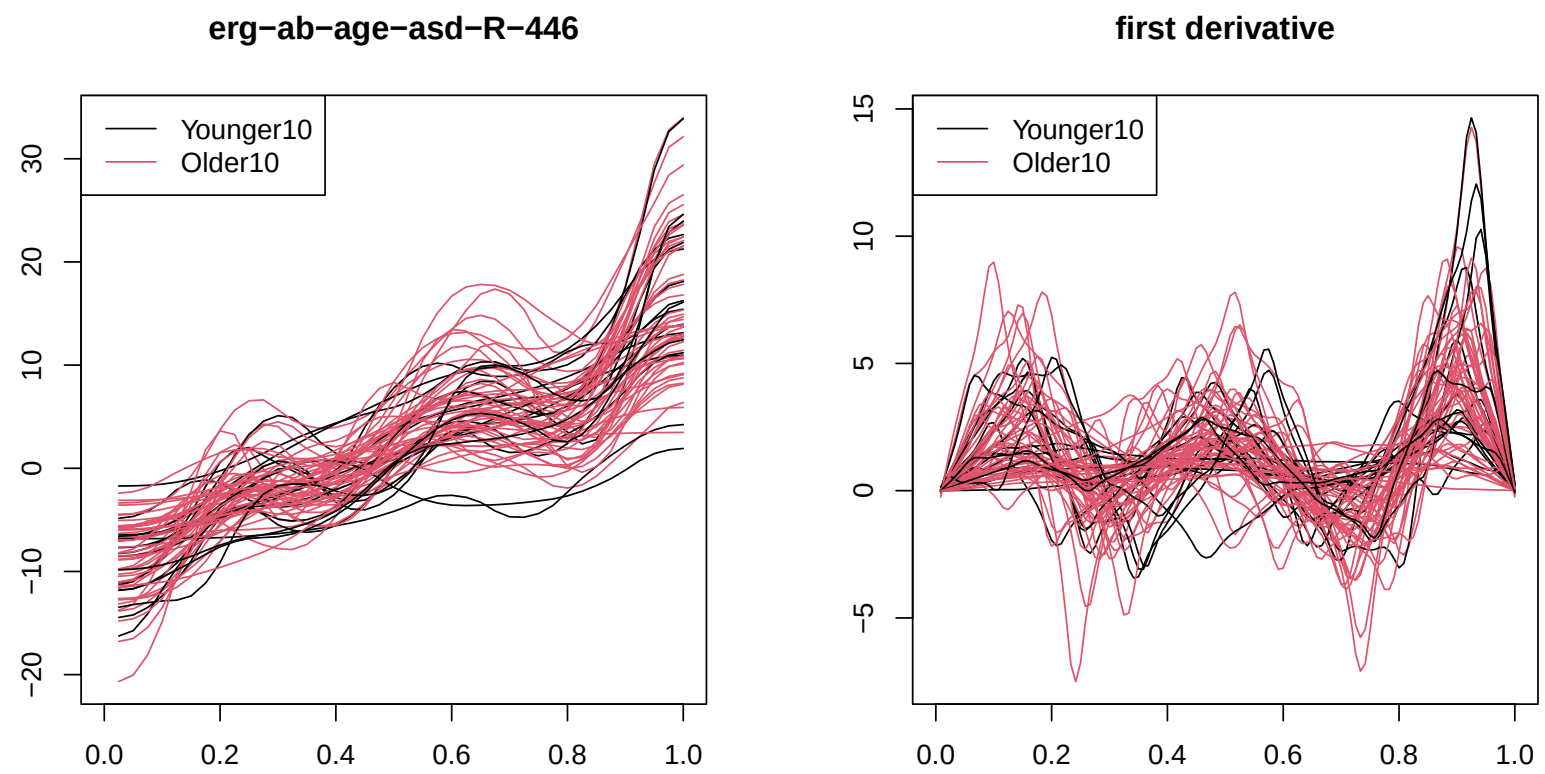
erg−ab−age−asd−R−446
Younger10
Older10
first derivative
Younger10
Older10

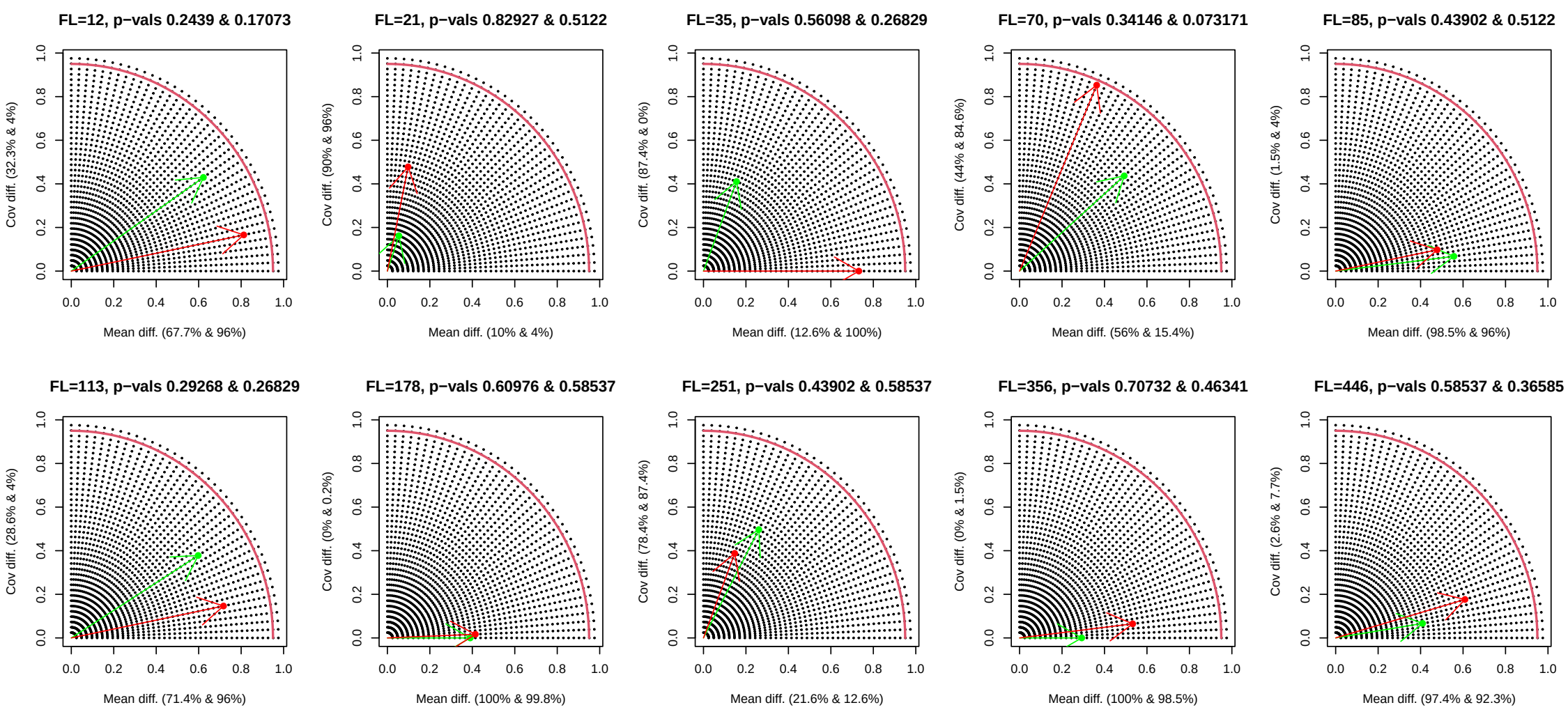
FL=12, p−vals 0.2439 & 0.17073
Cov diff. (32.3% & 4%)
Mean diff. (67.7% & 96%)
FL=21, p−vals 0.82927 & 0.5122
Cov diff. (90% & 96%)
Mean diff. (10% & 4%)
FL=35, p−vals 0.56098 & 0.26829
Cov diff. (87.4% & 0%)
Mean diff. (12.6% & 100%)
FL=70, p−vals 0.34146 & 0.073171
Cov diff. (44% & 84.6%)
Mean diff. (56% & 15.4%)
FL=85, p−vals 0.43902 & 0.5122
Cov diff. (1.5% & 4%)
Mean diff. (98.5% & 96%)
FL=113, p−vals 0.29268 & 0.26829
Cov diff. (28.6% & 4%)
Mean diff. (71.4% & 96%)
FL=178, p−vals 0.60976 & 0.58537
Cov diff. (0% & 0.2%)
Mean diff. (100% & 99.8%)
FL=251, p−vals 0.43902 & 0.58537
Cov diff. (78.4% & 87.4%)
Mean diff. (21.6% & 12.6%)
FL=356, p−vals 0.70732 & 0.46341
Cov diff. (0% & 1.5%)
Mean diff. (100% & 98.5%)
FL=446, p−vals 0.58537 & 0.36585
Cov diff. (2.6% & 7.7%)
Mean diff. (97.4% & 92.3%)

Table for ERG wave by age in atime–btime – asd subgroup

	pval-erg-ab	mean perc	cov perc	pval-derivative	mean perc	cov perc
12	0.2439			0.1707		
21	0.8293			0.5122		
35	0.5610			0.2683		
70	0.3415			0.0732		
85	0.4390			0.5122		
113	0.2927			0.2683		
178	0.6098			0.5854		
251	0.4390			0.5854		
356	0.7073			0.4634		
446	0.5854			0.3659		

2.19 ERG wave by age in btime–72 – ctr subgroup

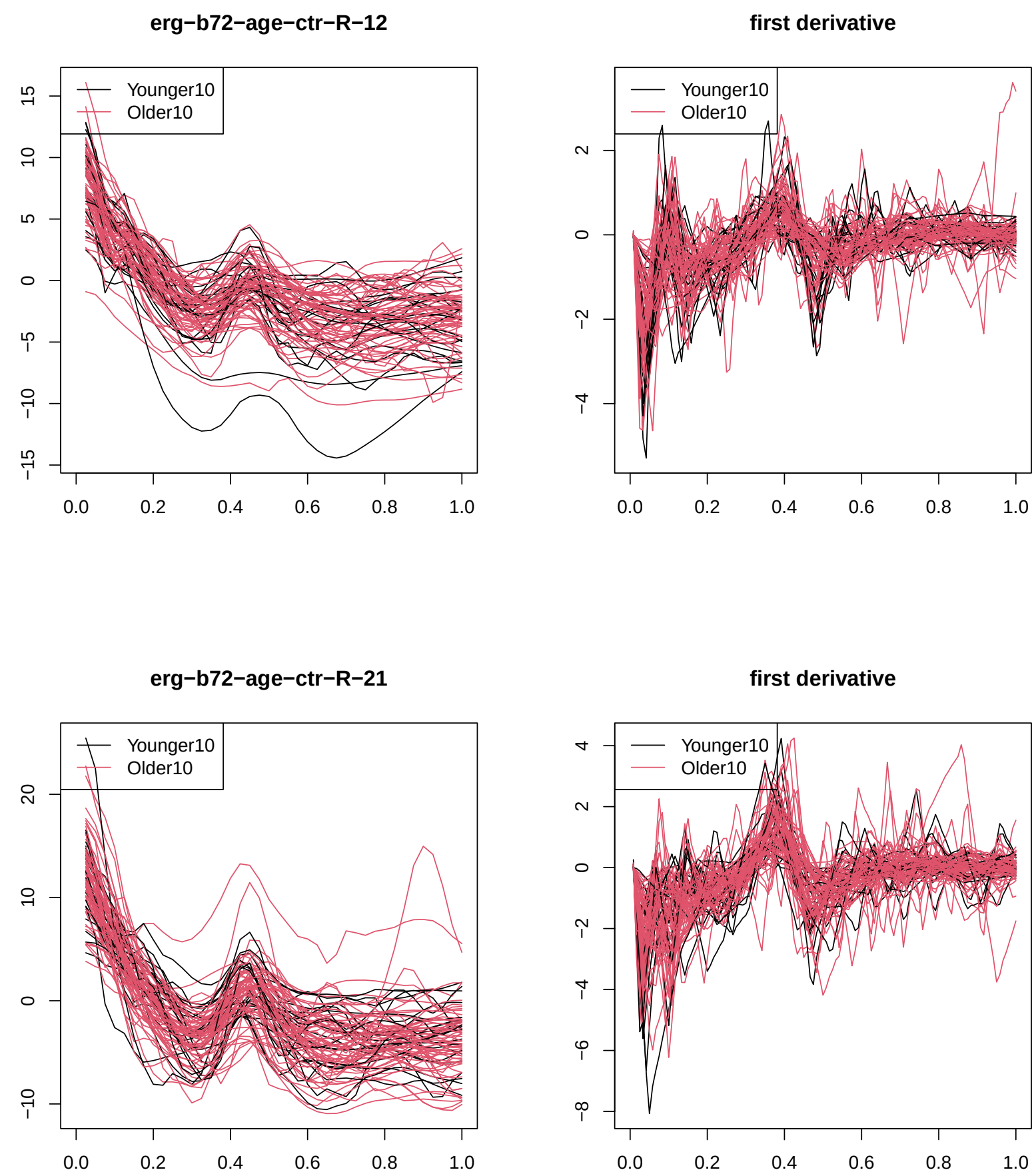

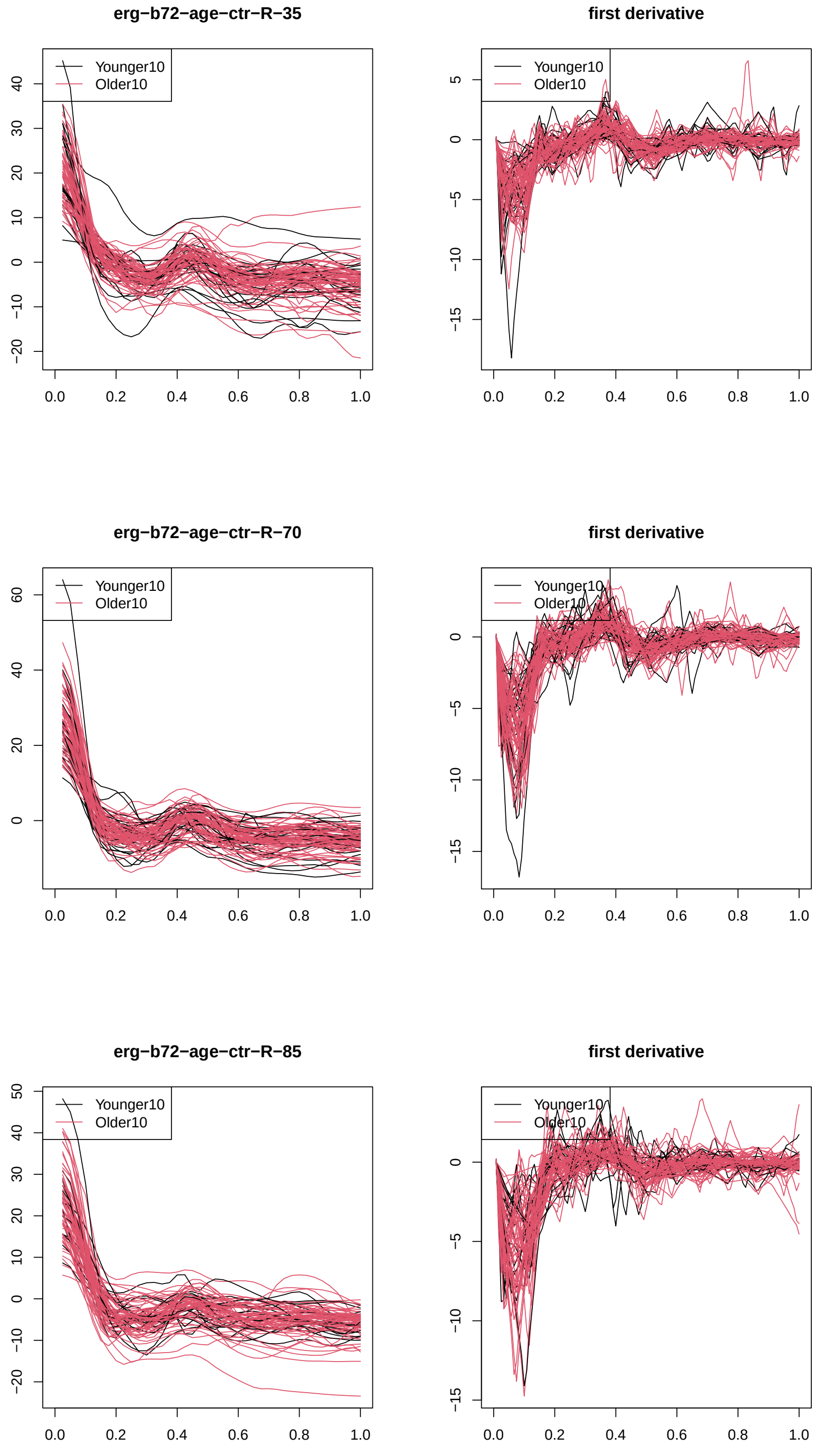
erg−b72−age−ctr−R−35
Younger10
Older10
first derivative
Younger10
Older10
erg−b72−age−ctr−R−70
Younger10
Older10
first derivative
Younger10
Older10
erg−b72−age−ctr−R−85
Younger10
Older10
first derivative
Younger10
Older10

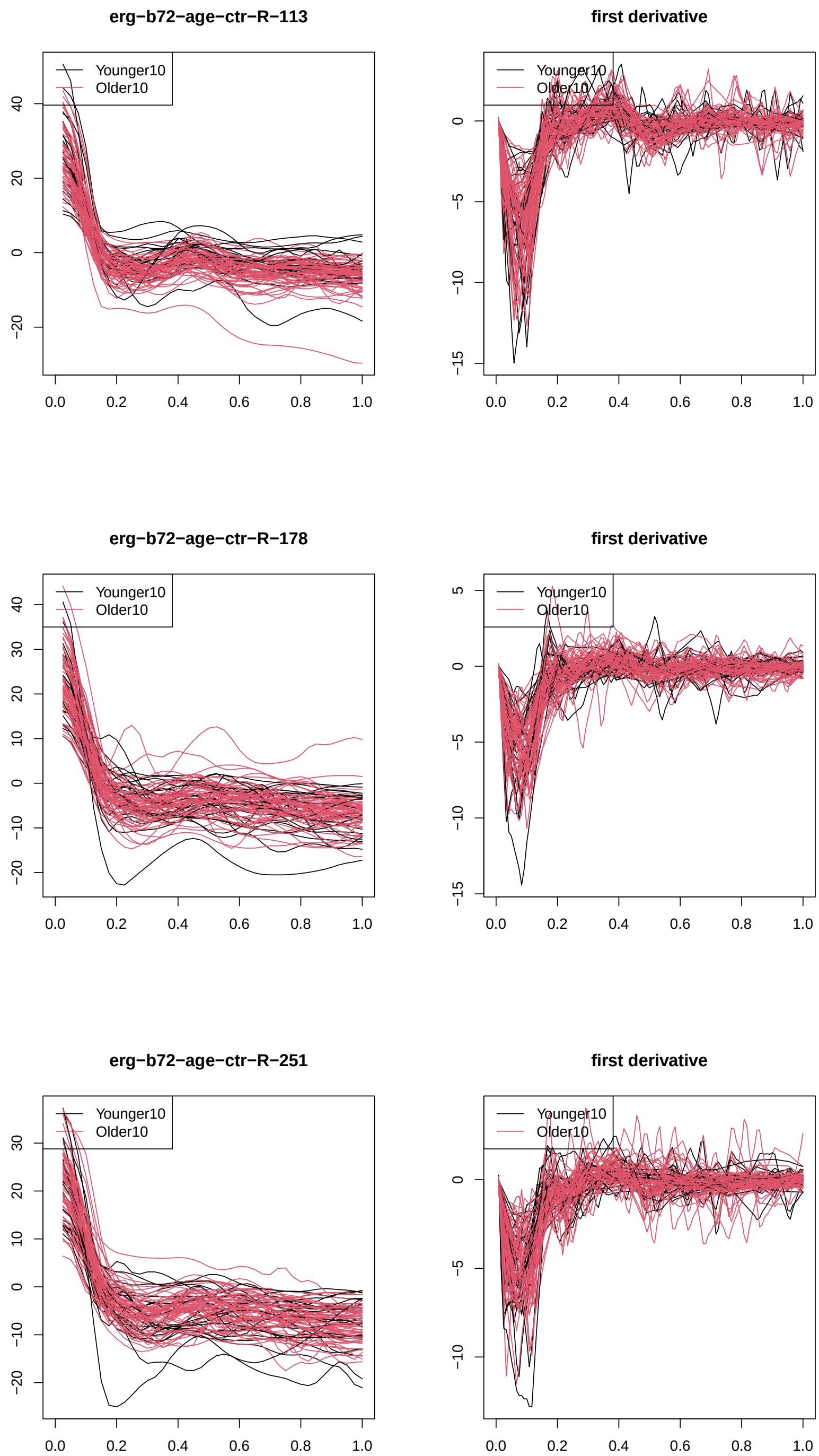
erg−b72−age−ctr−R−113
first derivative
Younger10
Older10
erg−b72−age−ctr−R−178
first derivative
Younger10
Older10
erg−b72−age−ctr−R−251
first derivative
Younger10
Older10

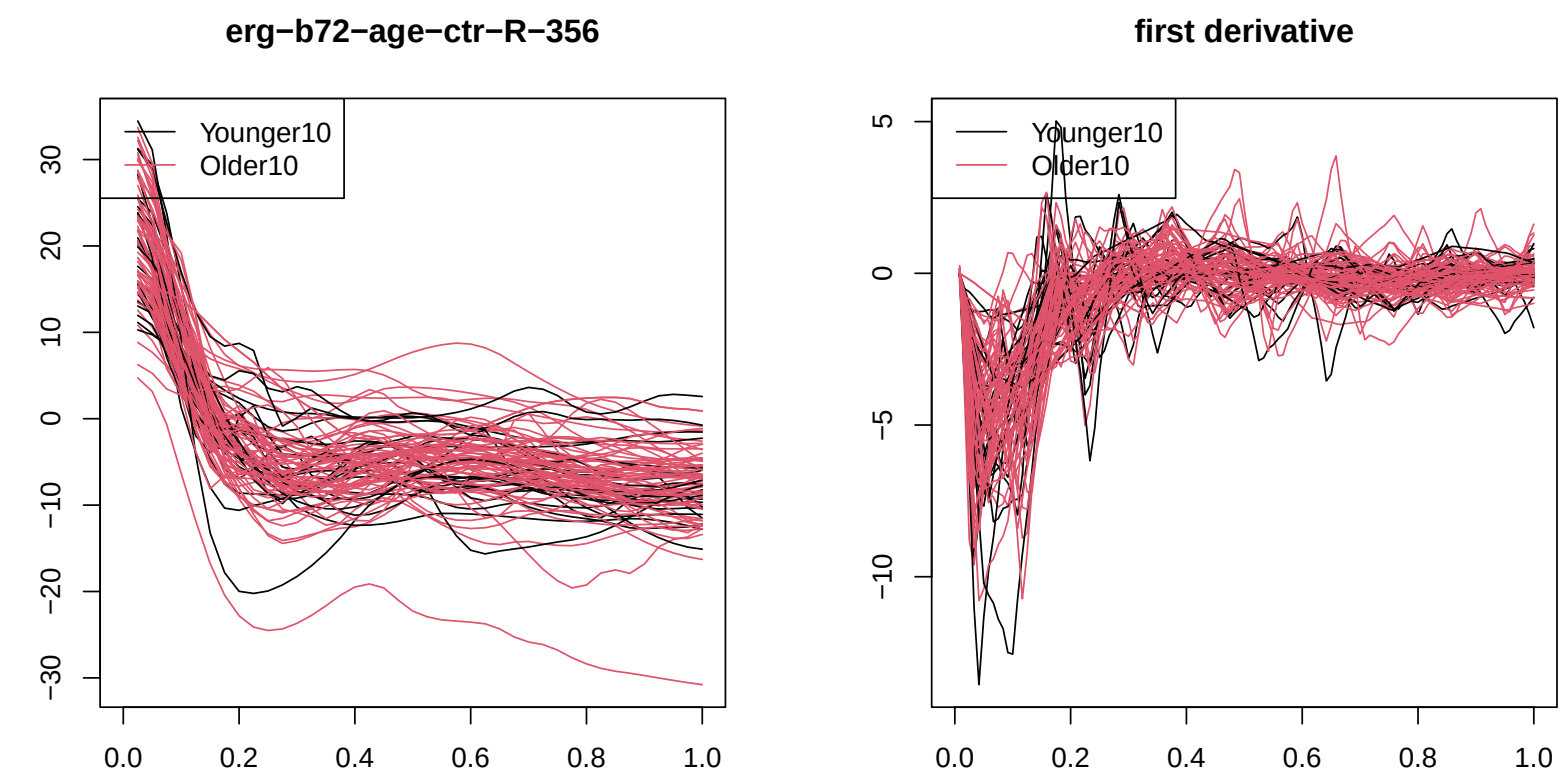
erg−b72−age−ctr−R−356
Younger10
Older10
first derivative
Younger10
Older10

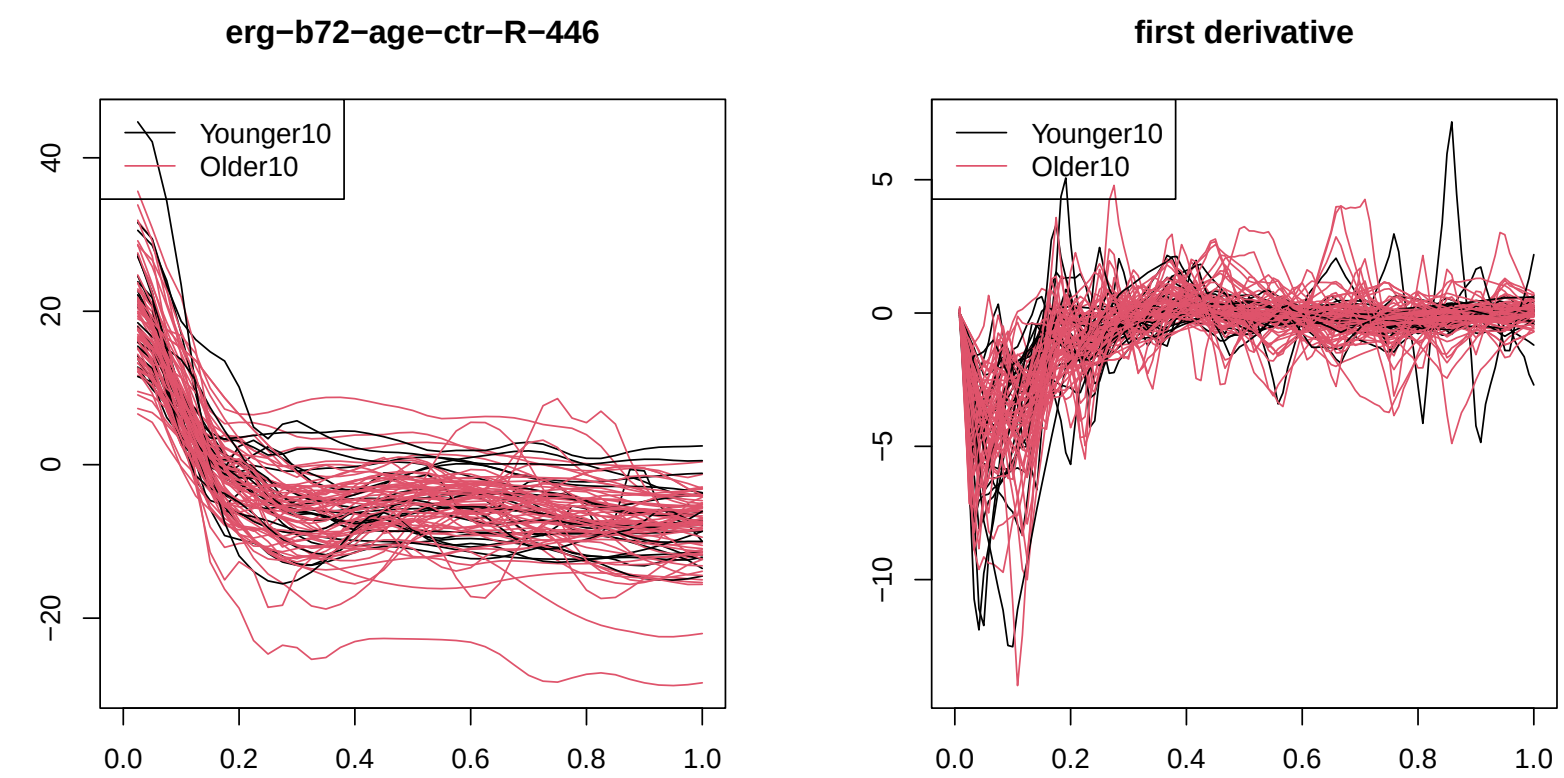
erg−b72−age−ctr−R−446
Younger10
Older10
first derivative
Younger10
Older10

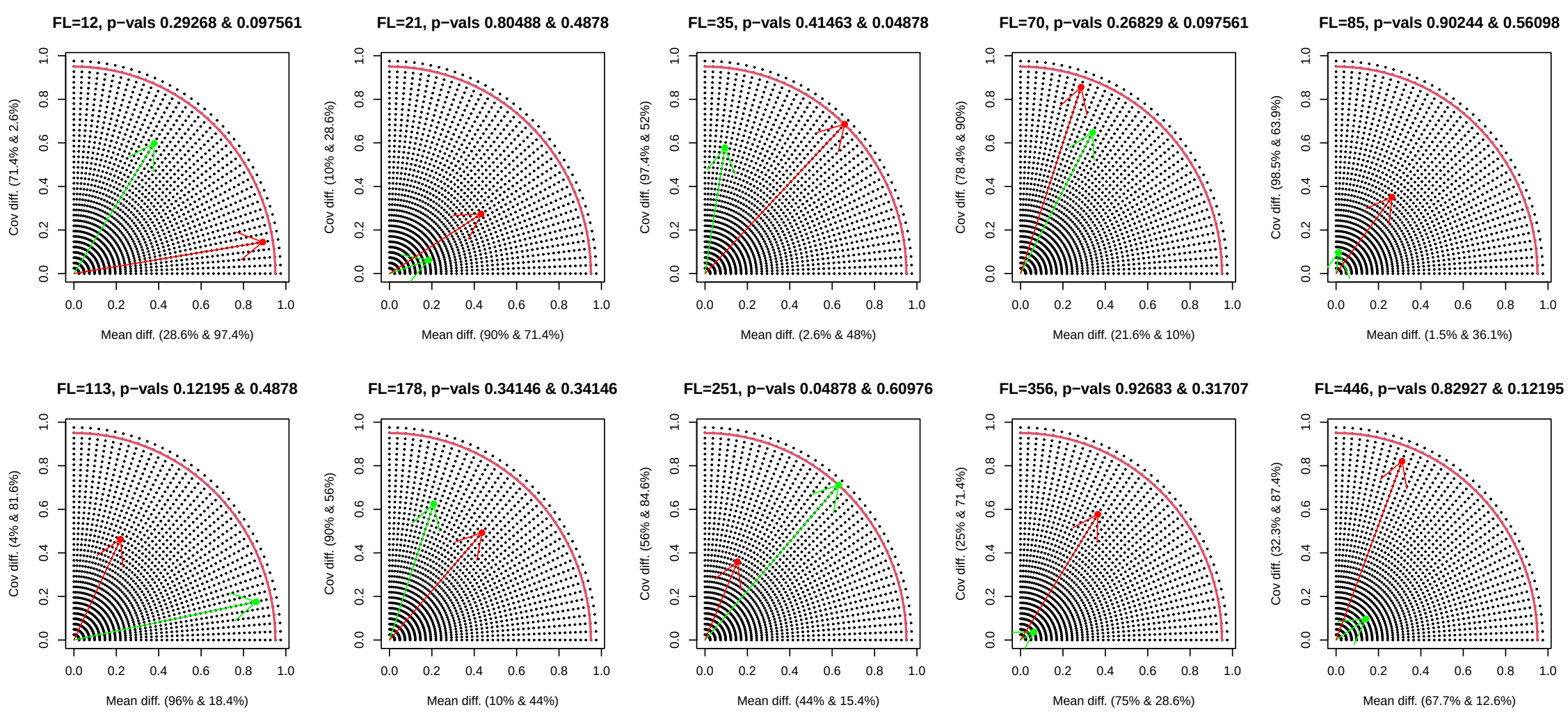
FL=12, p−vals 0.29268 & 0.097561
Mean diff. (28.6% & 97.4%)
Cov diff. (71.4% & 2.6%)
FL=21, p−vals 0.80488 & 0.4878
Mean diff. (90% & 71.4%)
Cov diff. (10% & 28.6%)
FL=35, p−vals 0.41463 & 0.04878
Mean diff. (2.6% & 48%)
Cov diff. (97.4% & 52%)
FL=70, p−vals 0.26829 & 0.097561
Mean diff. (21.6% & 10%)
Cov diff. (78.4% & 90%)
FL=85, p−vals 0.90244 & 0.56098
Mean diff. (1.5% & 36.1%)
Cov diff. (98.5% & 63.9%)
FL=113, p−vals 0.12195 & 0.4878
Mean diff. (96% & 18.4%)
Cov diff. (4% & 81.6%)
FL=178, p−vals 0.34146 & 0.34146
Mean diff. (10% & 44%)
Cov diff. (90% & 56%)
FL=251, p−vals 0.04878 & 0.60976
Mean diff. (44% & 15.4%)
Cov diff. (56% & 84.6%)
FL=356, p−vals 0.92683 & 0.31707
Mean diff. (75% & 28.6%)
Cov diff. (25% & 71.4%)
FL=446, p−vals 0.82927 & 0.12195
Mean diff. (67.7% & 12.6%)
Cov diff. (32.3% & 87.4%)

Table for ERG wave by age in btime–72 – ctr subgroup

	pval-erg-b72	mean perc	cov perc	pval-derivative	mean perc	cov perc
12	0.2927			0.0976		
21	0.8049			0.4878		
35	0.4146			0.0488	48	52
70	0.2683			0.0976		
85	0.9024			0.5610		
113	0.1220			0.4878		
178	0.3415			0.3415		
251	0.0488	44	56	0.6098		
356	0.9268			0.3171		
446	0.8293			0.1220		

2.20 ERG wave by age in btime–72 – asd subgroup

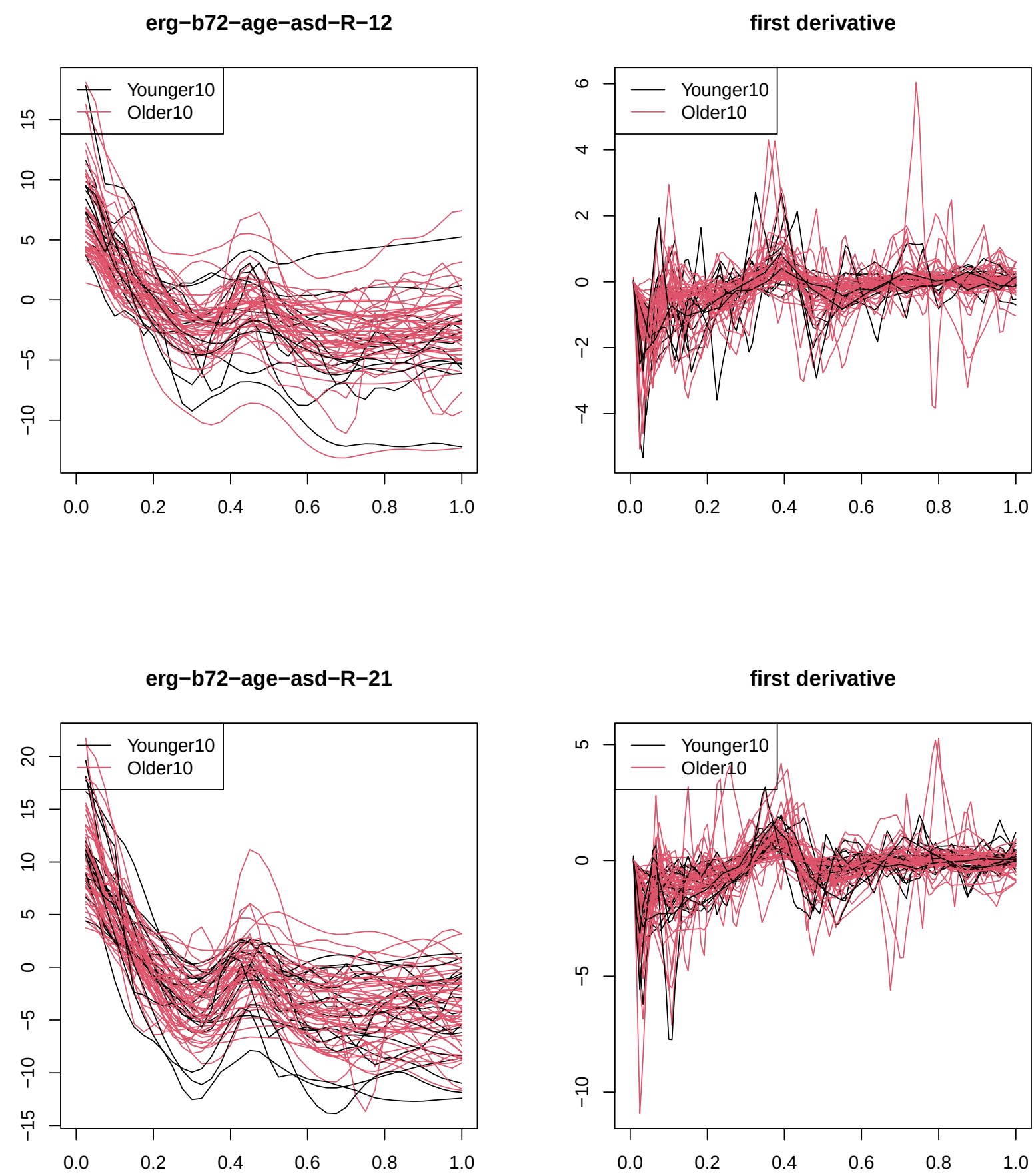

erg−b72−age−asd−R−35

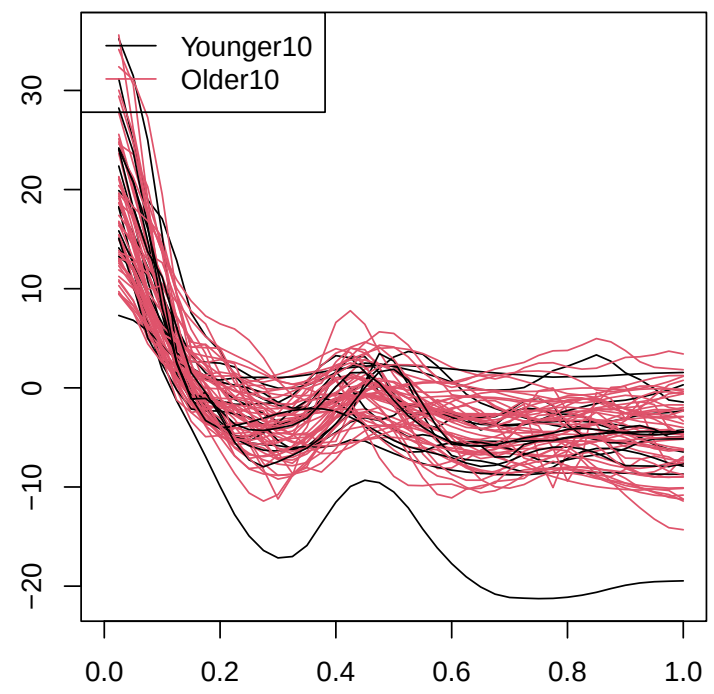

first derivative

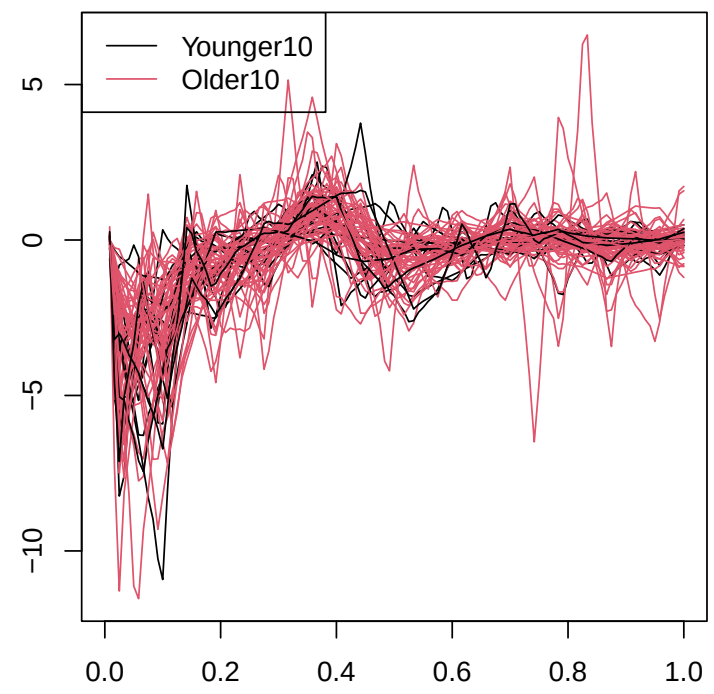

erg−b72−age−asd−R−70

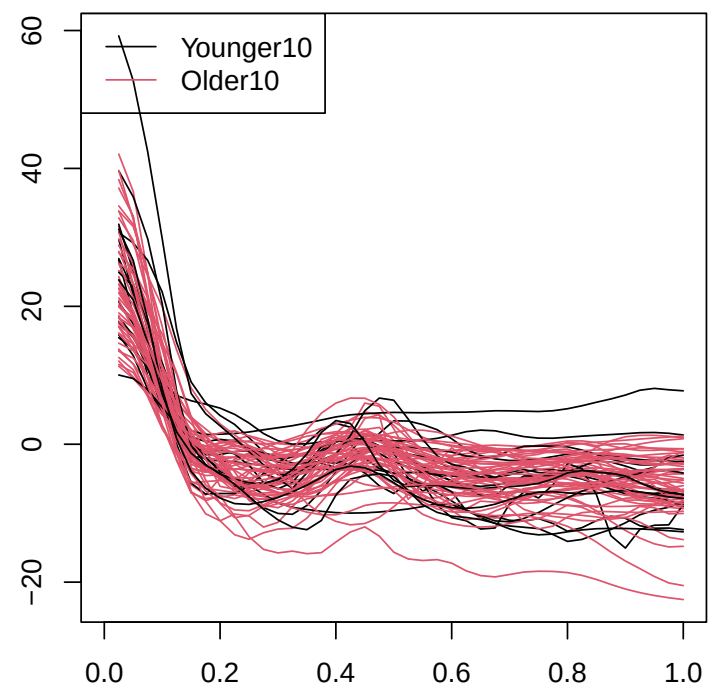

first derivative

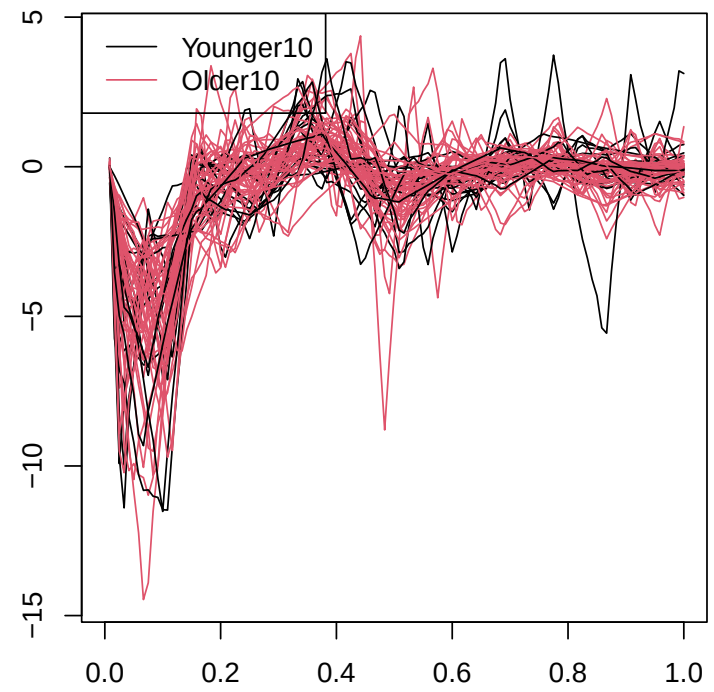

erg−b72−age−asd−R−85

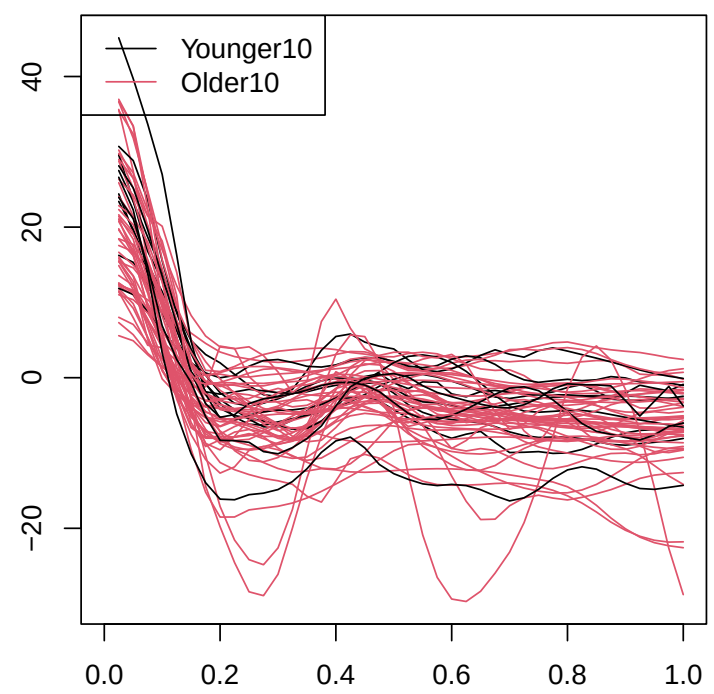

first derivative

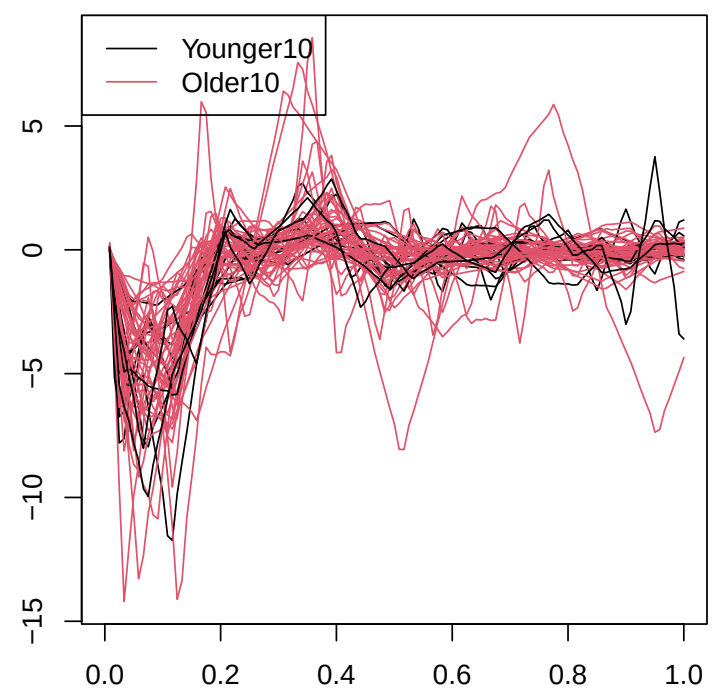

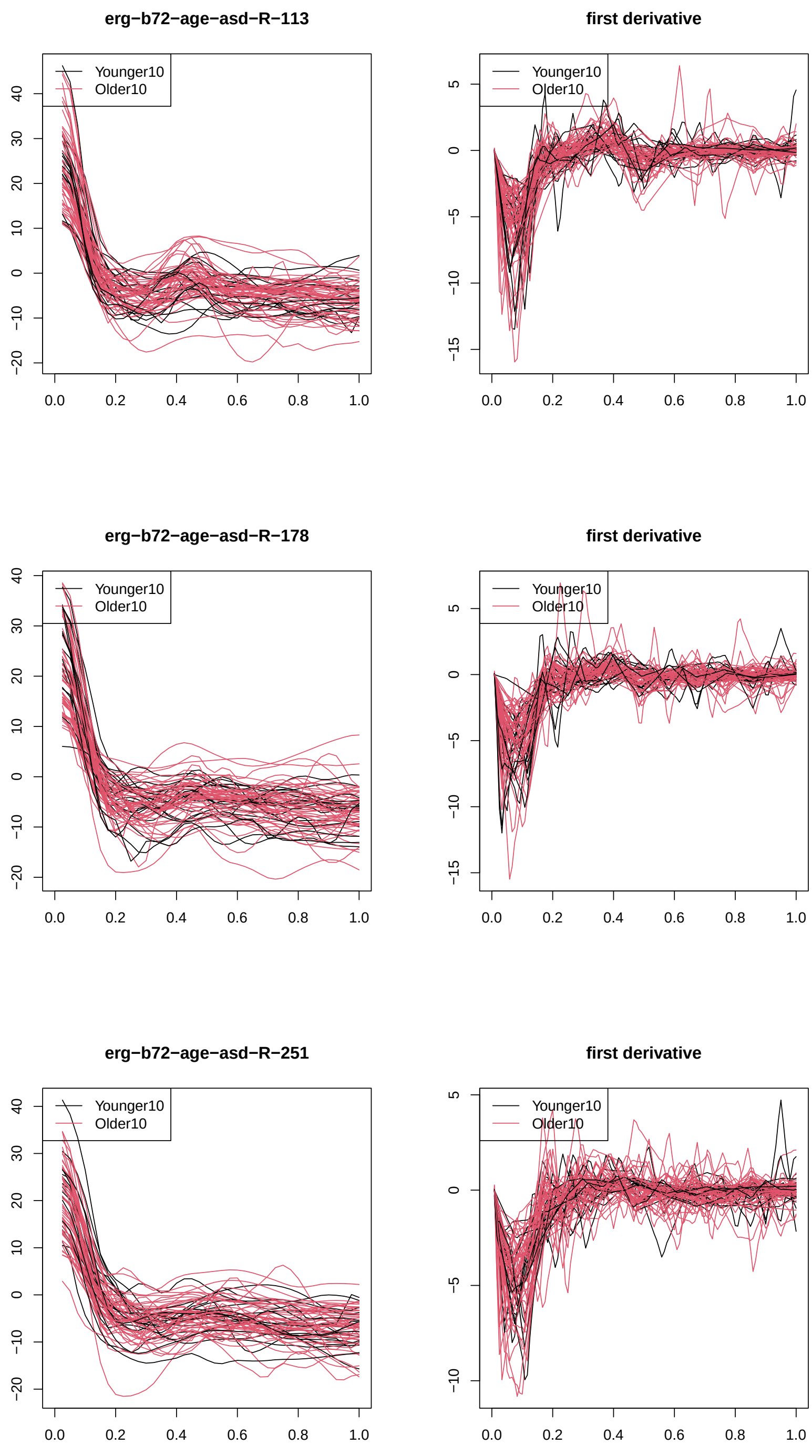
erg−b72−age−asd−R−113
first derivative
erg−b72−age−asd−R−178
first derivative
erg−b72−age−asd−R−251
first derivative
Younger10
Older10

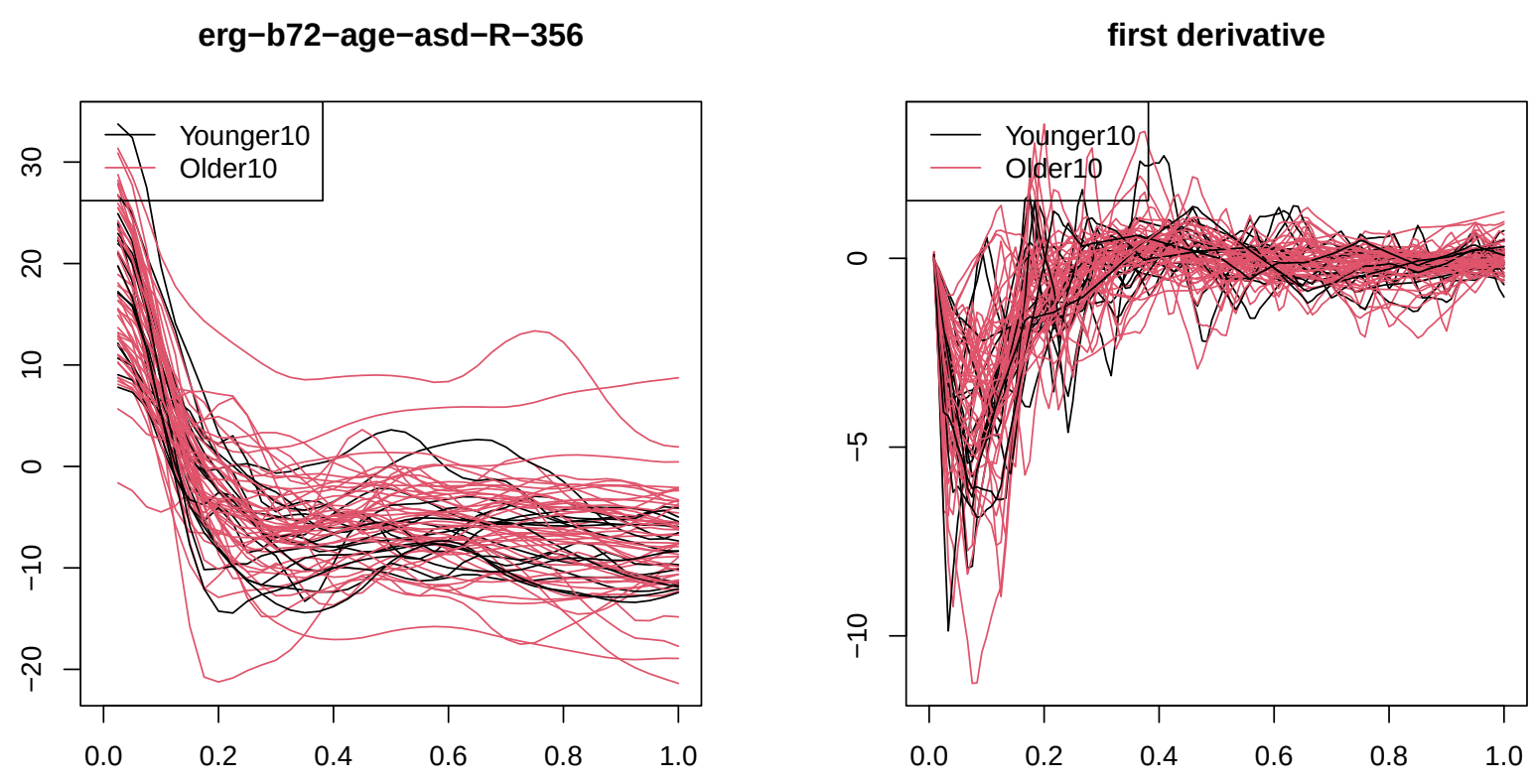
erg−b72−age−asd−R−356
first derivative
Younger10
Older10

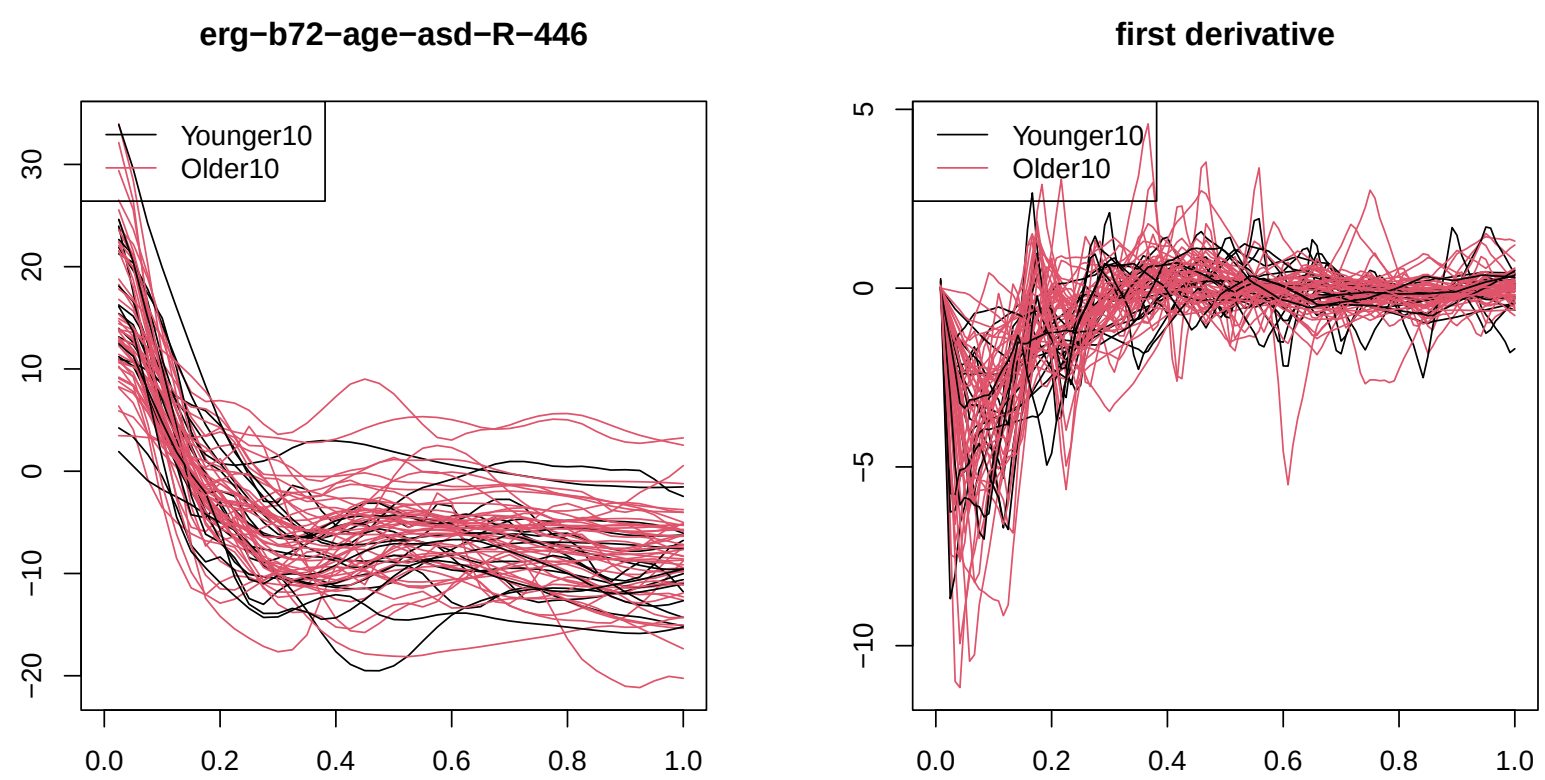
erg−b72−age−asd−R−446
first derivative
Younger10
Older10

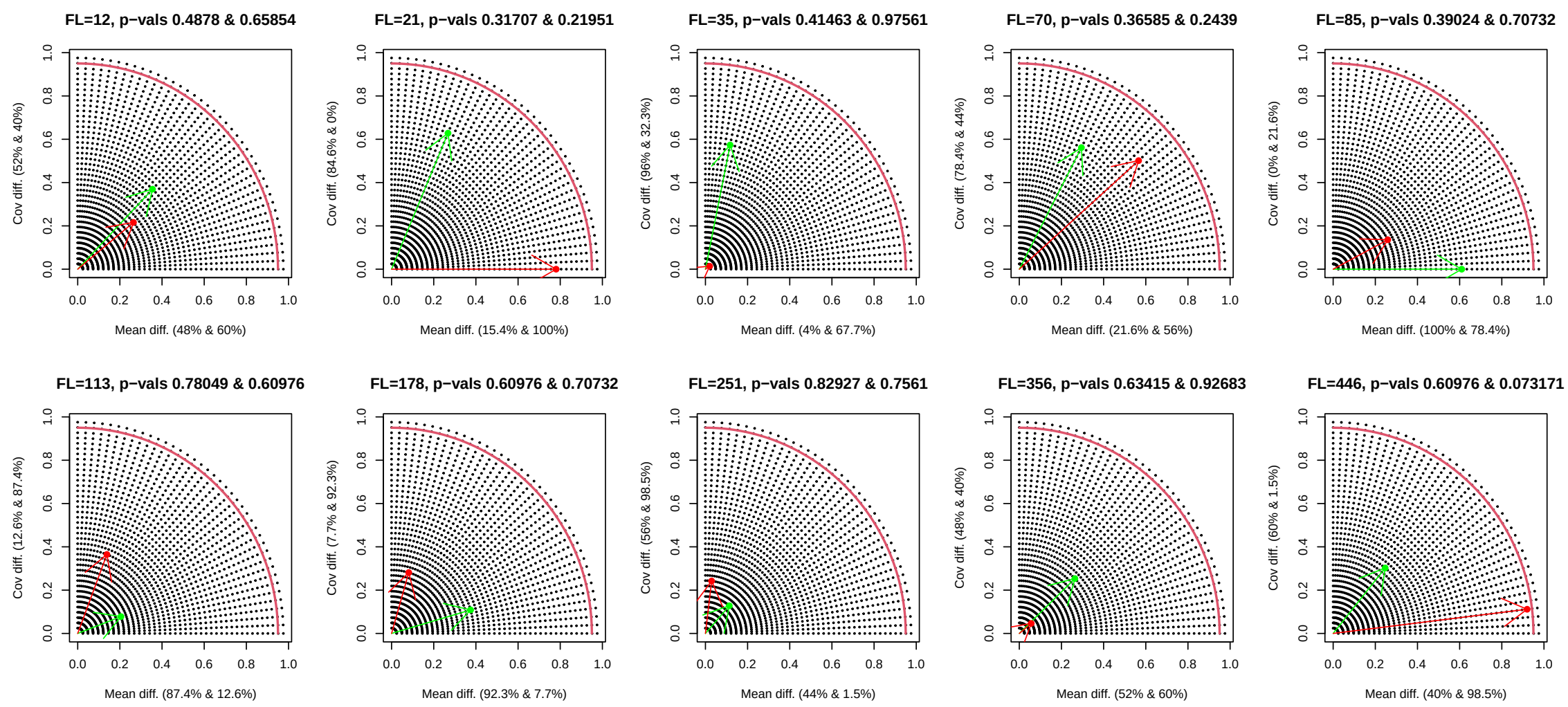
FL=12, p−vals 0.4878 & 0.65854
Mean diff. (48% & 60%)
FL=21, p−vals 0.31707 & 0.21951
Mean diff. (15.4% & 100%)
FL=35, p−vals 0.41463 & 0.97561
Mean diff. (4% & 67.7%)
FL=70, p−vals 0.36585 & 0.2439
Mean diff. (21.6% & 56%)
FL=85, p−vals 0.39024 & 0.70732
Mean diff. (100% & 78.4%)
FL=113, p−vals 0.78049 & 0.60976
Mean diff. (87.4% & 12.6%)
FL=178, p−vals 0.60976 & 0.70732
Mean diff. (92.3% & 7.7%)
FL=251, p−vals 0.82927 & 0.7561
Mean diff. (44% & 1.5%)
FL=356, p−vals 0.63415 & 0.92683
Mean diff. (52% & 60%)
FL=446, p−vals 0.60976 & 0.073171
Mean diff. (40% & 98.5%)

Table for ERG wave by age in btime–72 – asd subgroup

	pval-erg-b72	mean perc	cov perc	pval-derivative	mean perc	cov perc
12	0.4878			0.6585		
21	0.3171			0.2195		
35	0.4146			0.9756		
70	0.3659			0.2439		
85	0.3902			0.7073		
113	0.7805			0.6098		
178	0.6098			0.7073		
251	0.8293			0.7561		
356	0.6341			0.9268		
446	0.6098			0.0732		

Chapter 3

Two-sample simultaneous comparison of mean functions (the wave and its 1st derivative), right eye

Data set: ERGandOP.xlsx, spline approximations of the waves.

Similar analysis without the covariance operators. The test now combines simultaneous comparisons of mean functions of the waveform and 1st derivatives.

3.1 OP wave by asd in 0–60 – all obs.

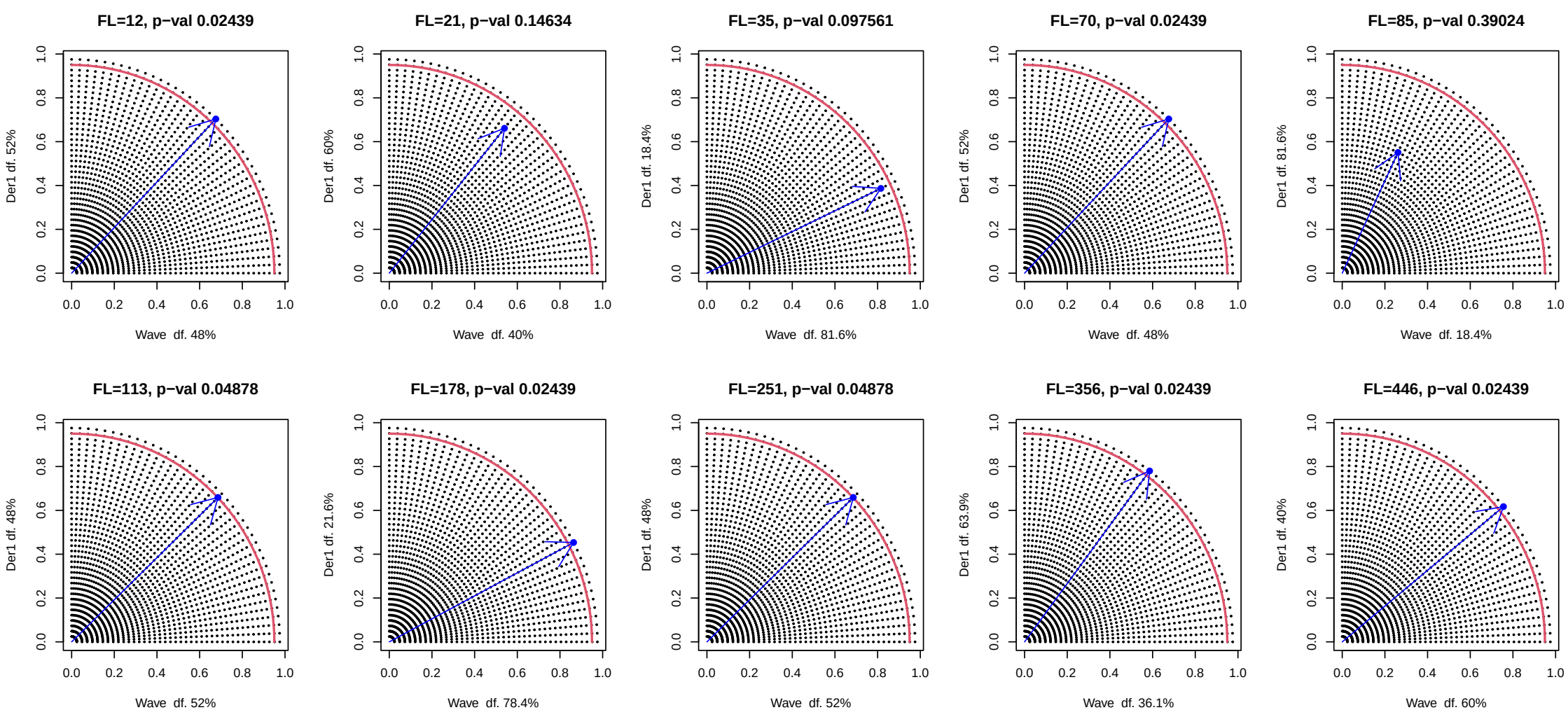

Table for OP wave by asd in 0–60 – all obs.

	pval-op-060	wave perc	der1 perc
12	0.0244	48	52
21	0.1463		
35	0.0976		
70	0.0244	48	52
85	0.3902		
113	0.0488	52	48
178	0.0244	78.4	21.6
251	0.0488	52	48
356	0.0244	36.1	63.9
446	0.0244	60	40

3.2 ERG wave by asd in 0–atime – all obs.

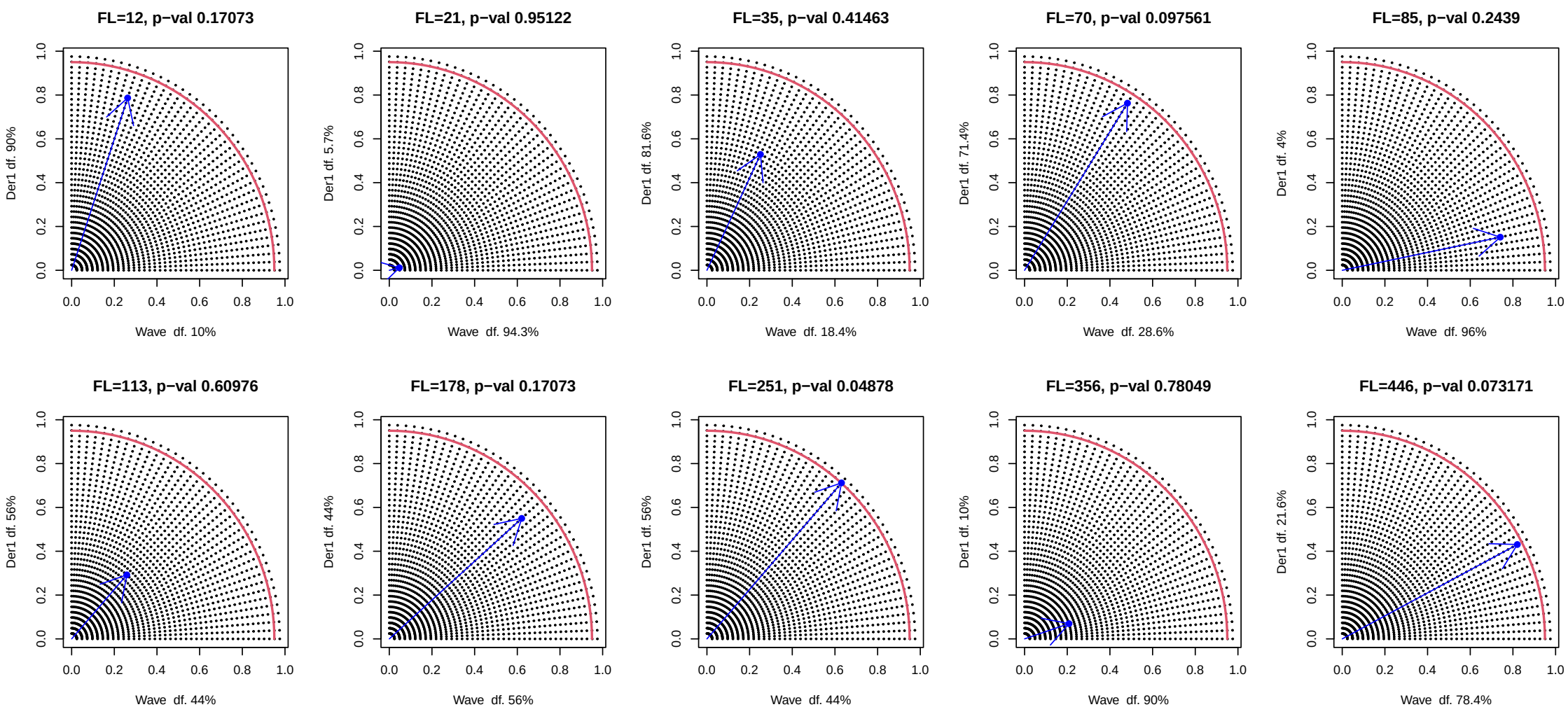

Table for ERG wave by asd in 0–atime – all obs.

	pval-erg-0a	wave perc	der1 perc
12	0.1707		
21	0.9512		
35	0.4146		
70	0.0976		
85	0.2439		
113	0.6098		
178	0.1707		
251	0.0488	44	56
356	0.7805		
446	0.0732		

3.3 ERG wave by asd in atime–btime – all obs.

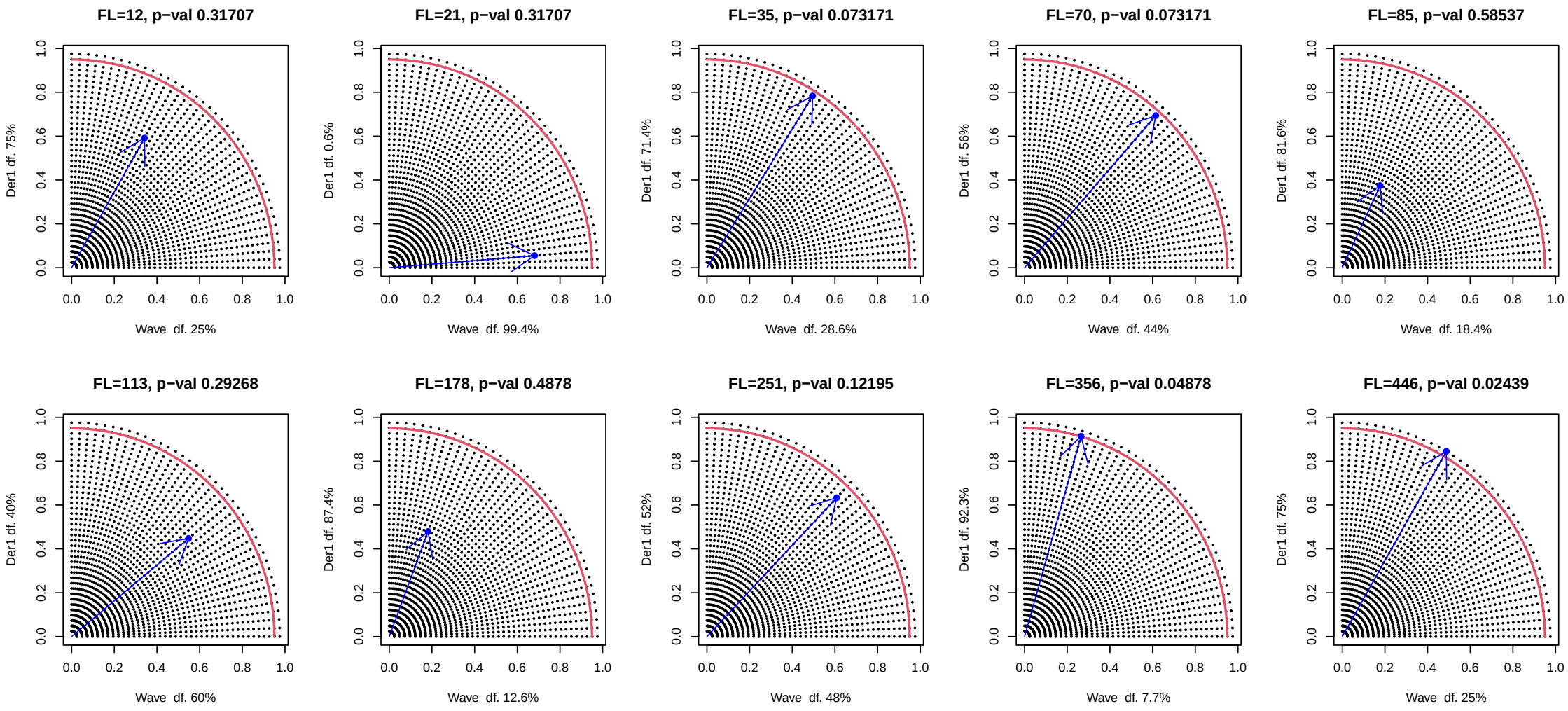

Table for ERG wave by asd in atime–btime – all obs.

	pval-erg-ab	wave perc	der1 perc
12	0.3171		
21	0.3171		
35	0.0732		
70	0.0732		
85	0.5854		
113	0.2927		
178	0.4878		
251	0.1220		
356	0.0488	7.7	92.3
446	0.0244	25	75

3.4 ERG wave by asd in btime–72 – all obs.

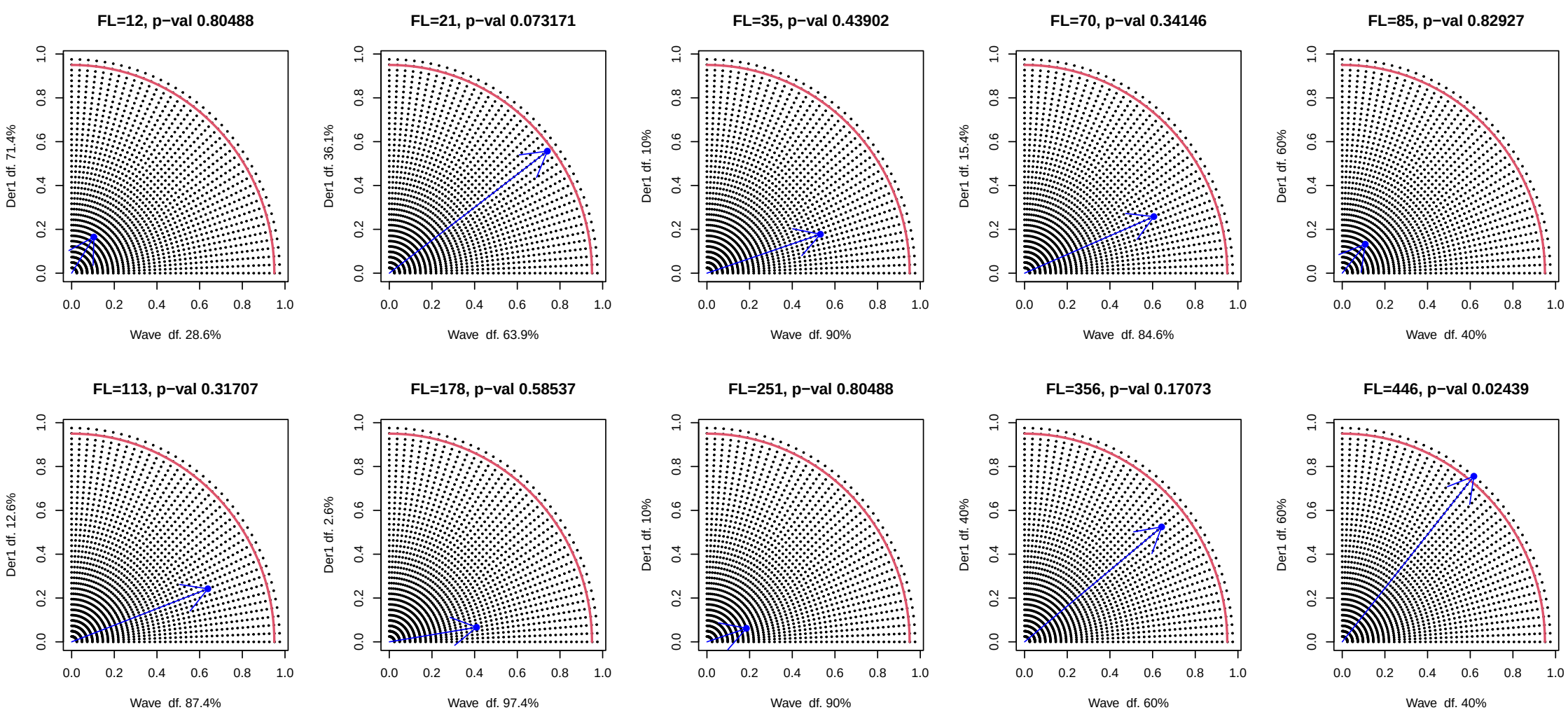

Table for ERG wave by asd in btime–72 – all obs.

	pval-erg-b72	wave perc	der1 perc
12	0.8049		
21	0.0732		
35	0.4390		
70	0.3415		
85	0.8293		
113	0.3171		
178	0.5854		
251	0.8049		
356	0.1707		
446	0.0244	40	60

3.5 OP wave by sex in 0–60 – ctr subgroup

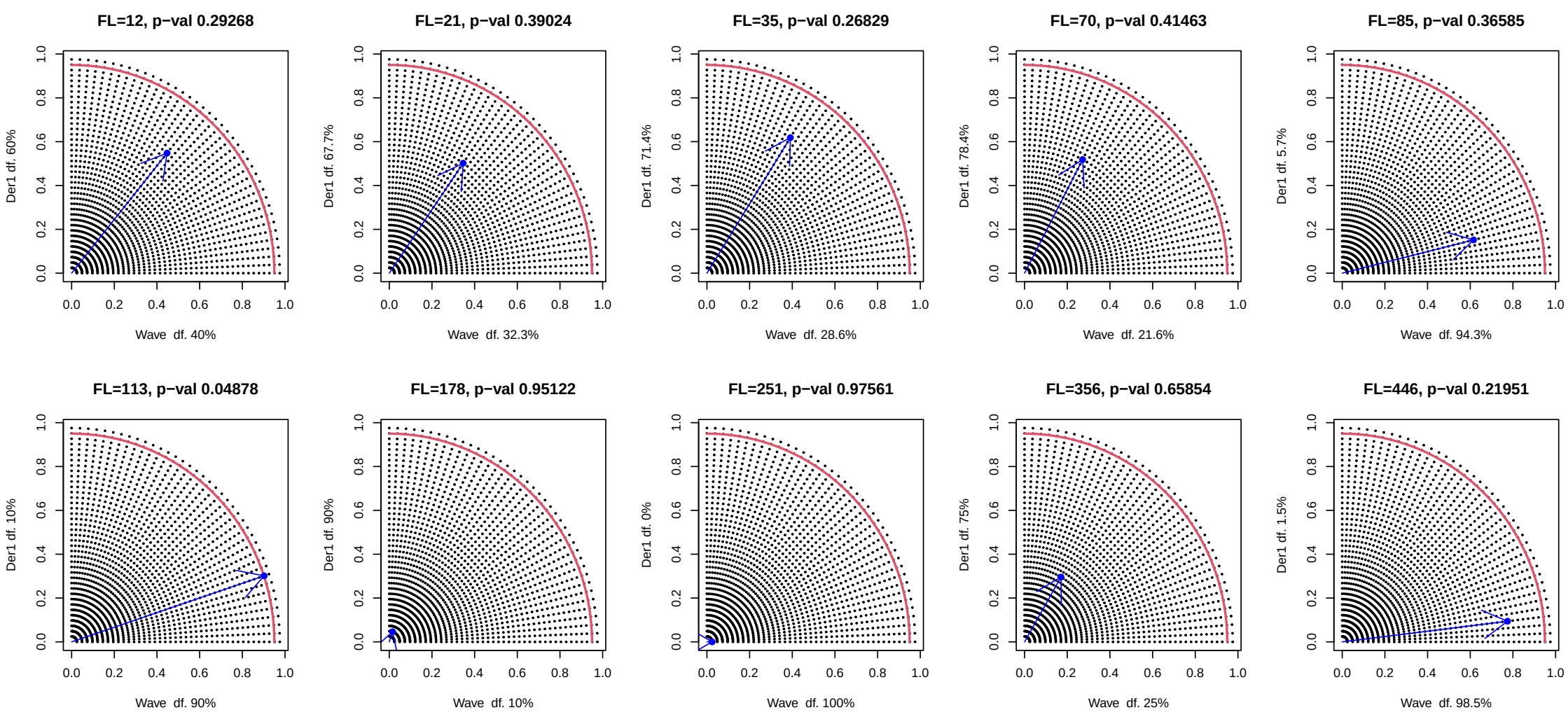

Table for OP wave by sex in 0–60 – ctr subgroup

	pval-op-060	wave perc	der1 perc
12	0.2927		
21	0.3902		
35	0.2683		
70	0.4146		
85	0.3659		
113	0.0488	90	10
178	0.9512		
251	0.9756		
356	0.6585		
446	0.2195		

3.6 OP wave by sex in 0–60 – asd subgroup

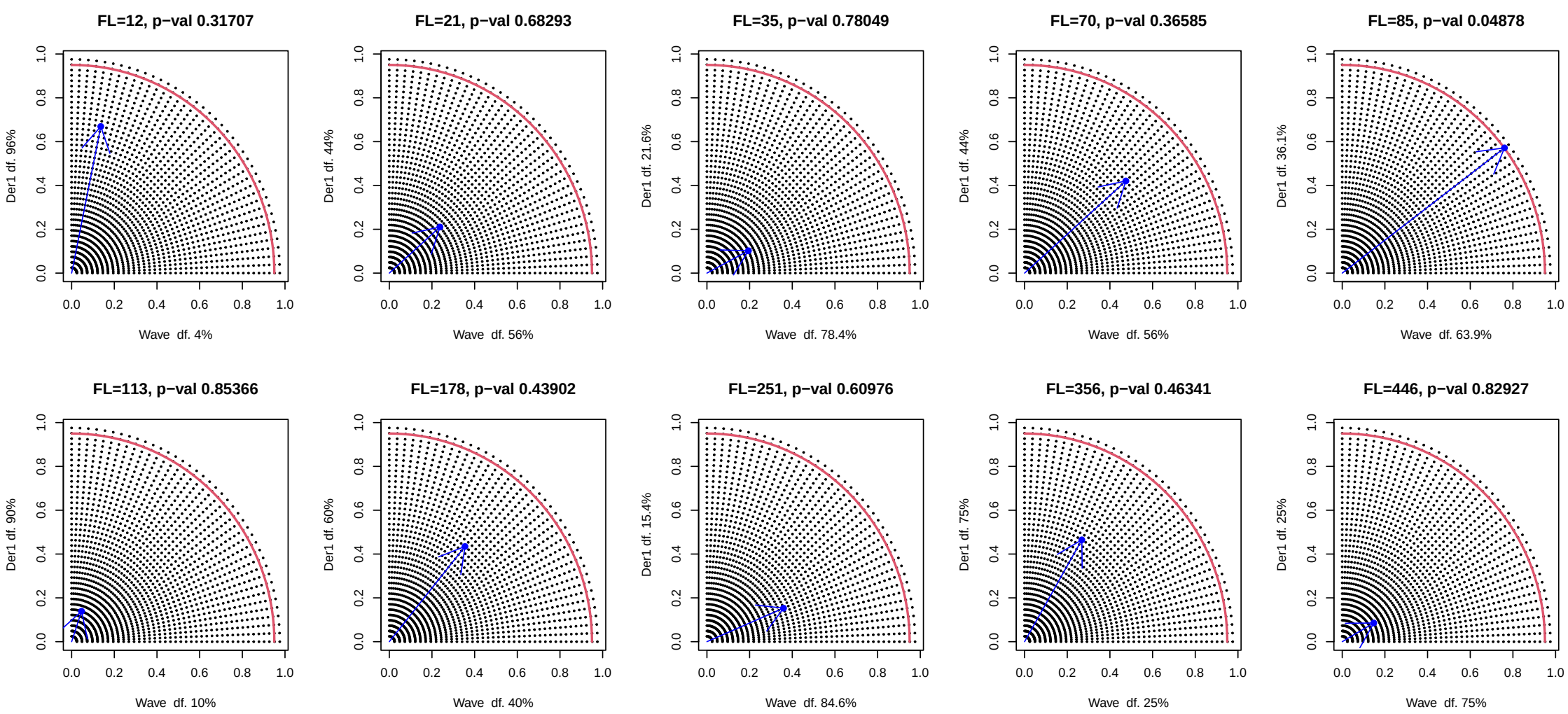

Table for OP wave by sex in 0–60 – asd subgroup

	pval-op-060	wave perc	der1 perc
12	0.3171		
21	0.6829		
35	0.7805		
70	0.3659		
85	0.0488	63.9	36.1
113	0.8537		
178	0.4390		
251	0.6098		
356	0.4634		
446	0.8293		

3.7 ERG wave by sex in 0–atime – ctr subgroup

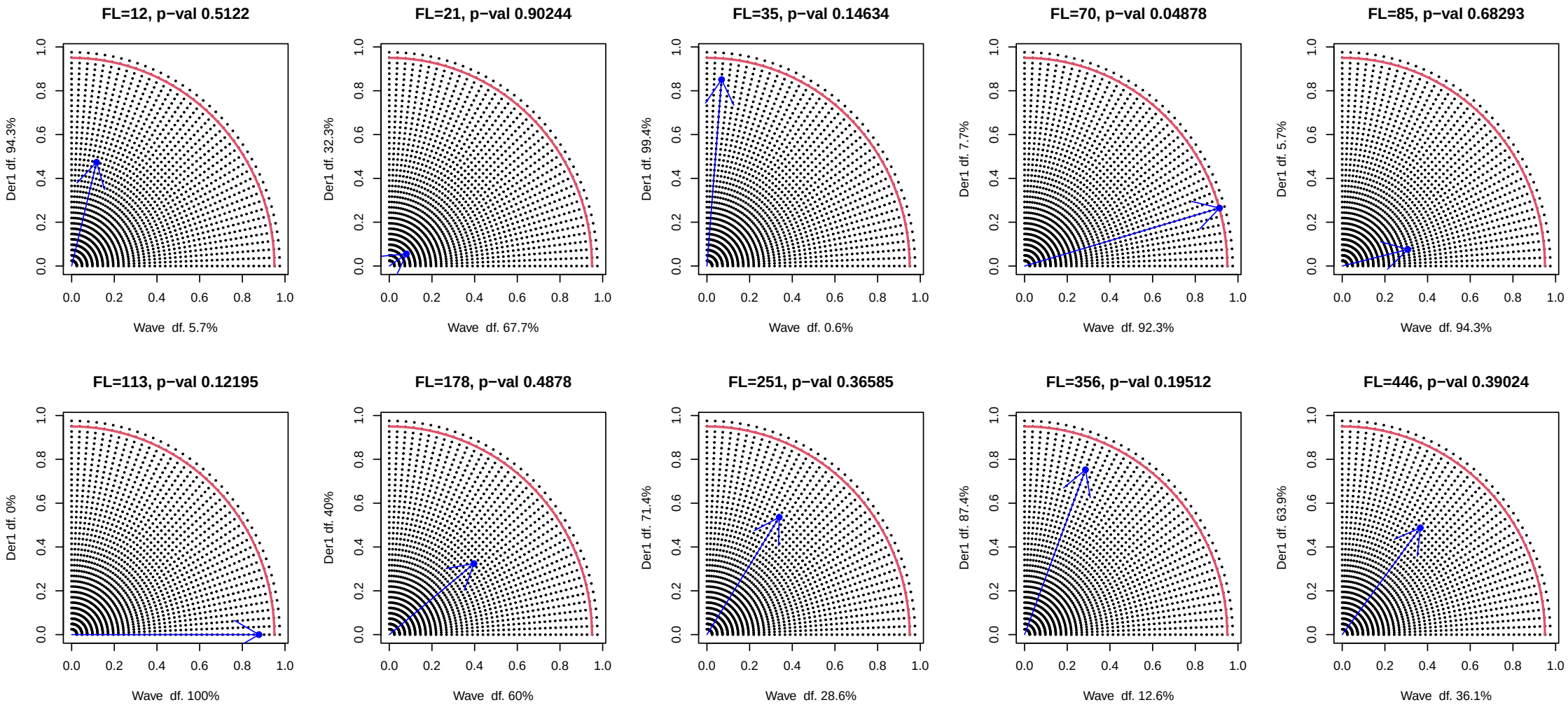

Table for ERG wave by sex in 0–atime – ctr subgroup

	pval-erg-0a	wave perc	der1 perc
12	0.5122		
21	0.9024		
35	0.1463		
70	0.0488	92.3	7.7
85	0.6829		
113	0.1220		
178	0.4878		
251	0.3659		
356	0.1951		
446	0.3902		

3.8 ERG wave by sex in 0–atime – asd subgroup

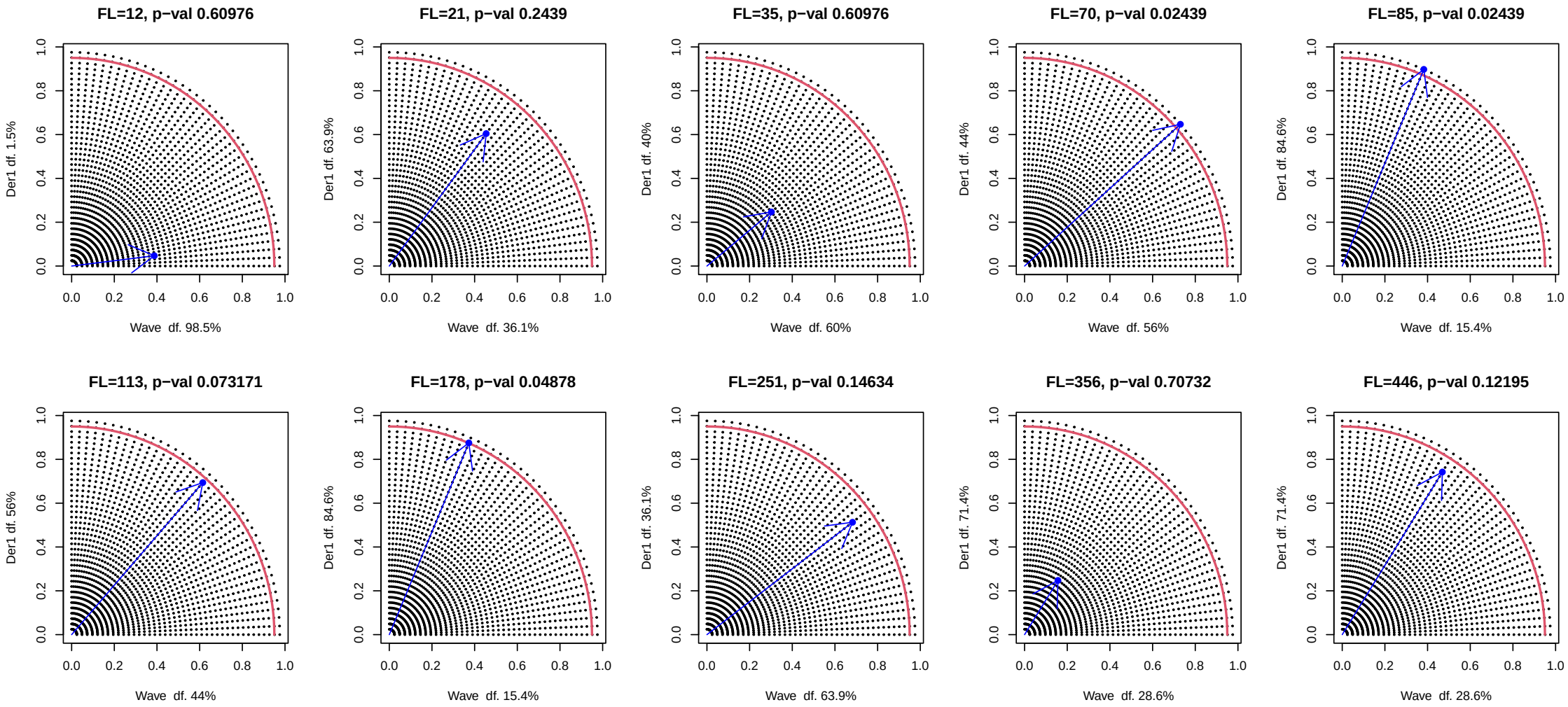

Table for ERG wave by sex in 0–atime – asd subgroup

	pval-erg-0a	wave perc	der1 perc
12	0.6098		
21	0.2439		
35	0.6098		
70	0.0244	56	44
85	0.0244	15.4	84.6
113	0.0732		
178	0.0488	15.4	84.6
251	0.1463		
356	0.7073		
446	0.1220		

3.9 ERG wave by sex in atime–btime – ctr subgroup

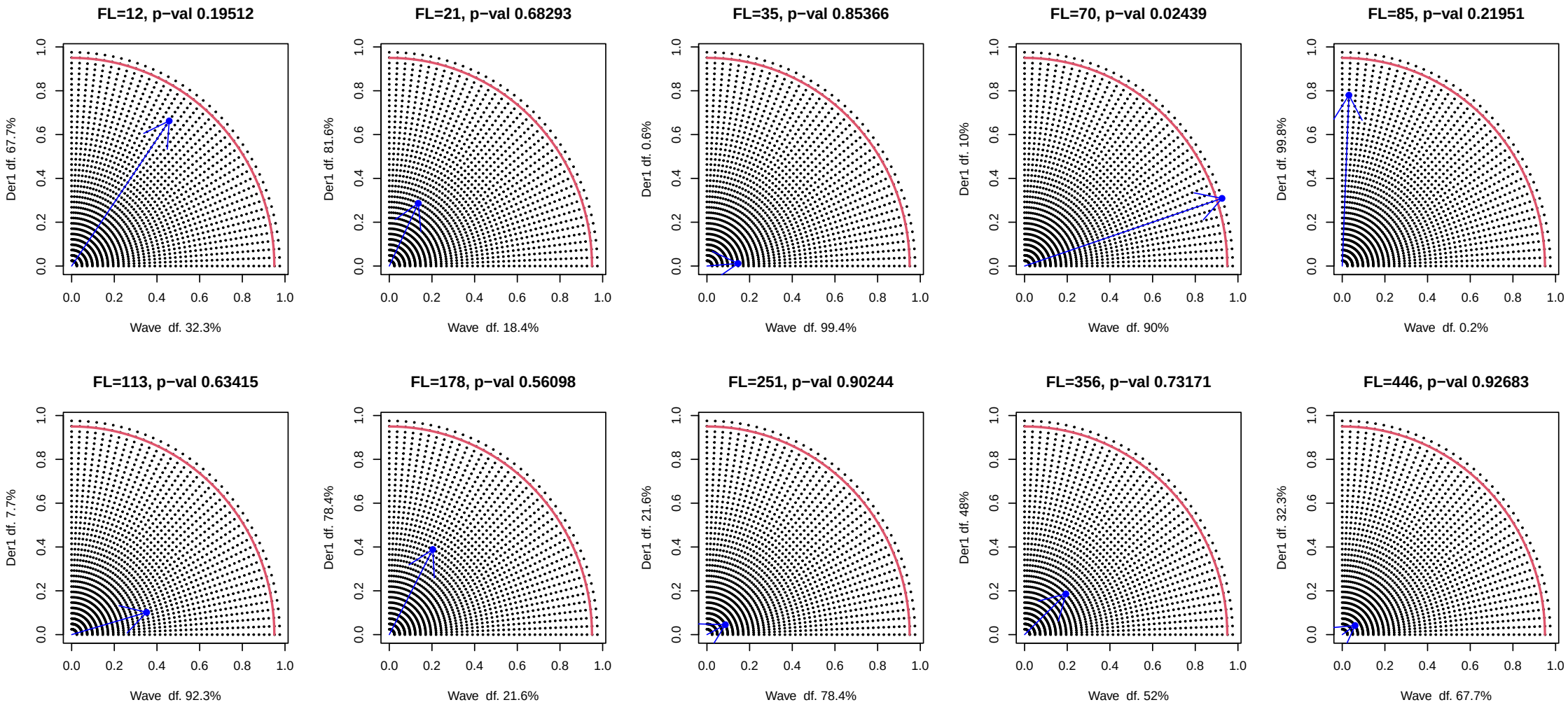

Table for ERG wave by sex in atime–btime – ctr subgroup

	pval-erg-ab	wave perc	der1 perc
12	0.1951		
21	0.6829		
35	0.8537		
70	0.0244	90	10
85	0.2195		
113	0.6341		
178	0.5610		
251	0.9024		
356	0.7317		
446	0.9268		

3.10 ERG wave by sex in atime–btime – asd subgroup

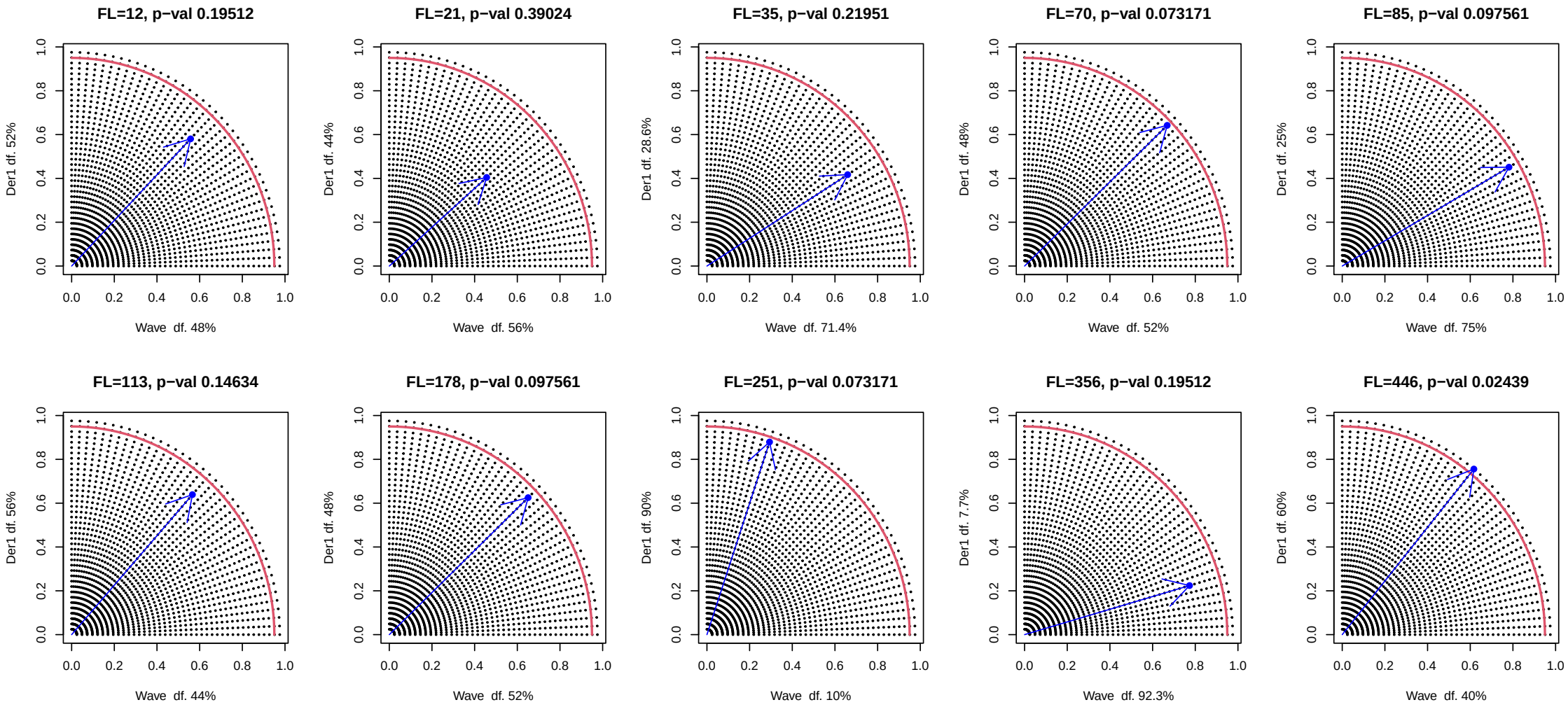

Table for ERG wave by sex in atime–btime – asd subgroup

	pval-erg-ab	wave perc	der1 perc
12	0.1951		
21	0.3902		
35	0.2195		
70	0.0732		
85	0.0976		
113	0.1463		
178	0.0976		
251	0.0732		
356	0.1951		
446	0.0244	40	60

3.11 ERG wave by sex in btime–72 – ctr subgroup

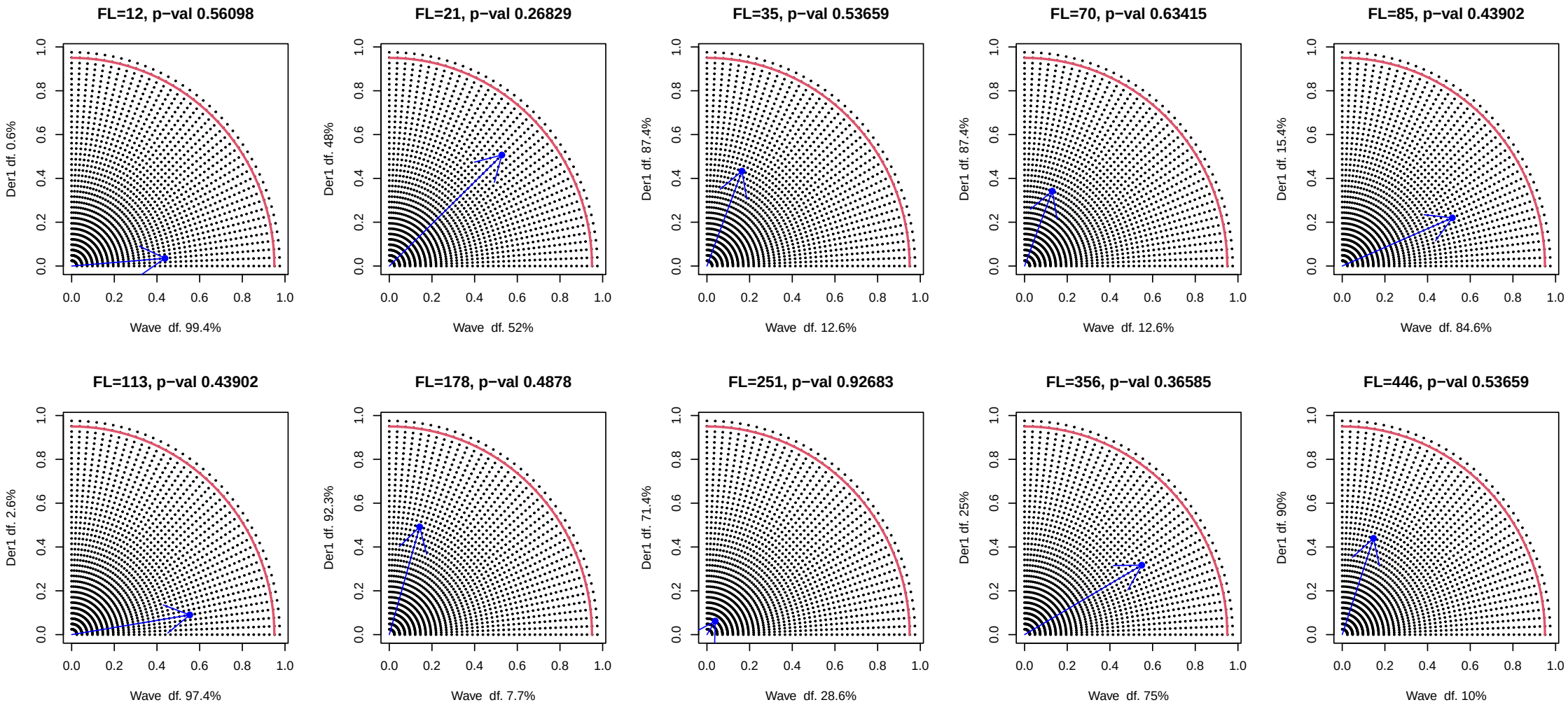

Table for ERG wave by sex in btime–72 – ctr subgroup

	pval-erg-b72	wave perc	der1 perc
12	0.5610		
21	0.2683		
35	0.5366		
70	0.6341		
85	0.4390		
113	0.4390		
178	0.4878		
251	0.9268		
356	0.3659		
446	0.5366		

3.12 ERG wave by sex in btime–72 – asd subgroup

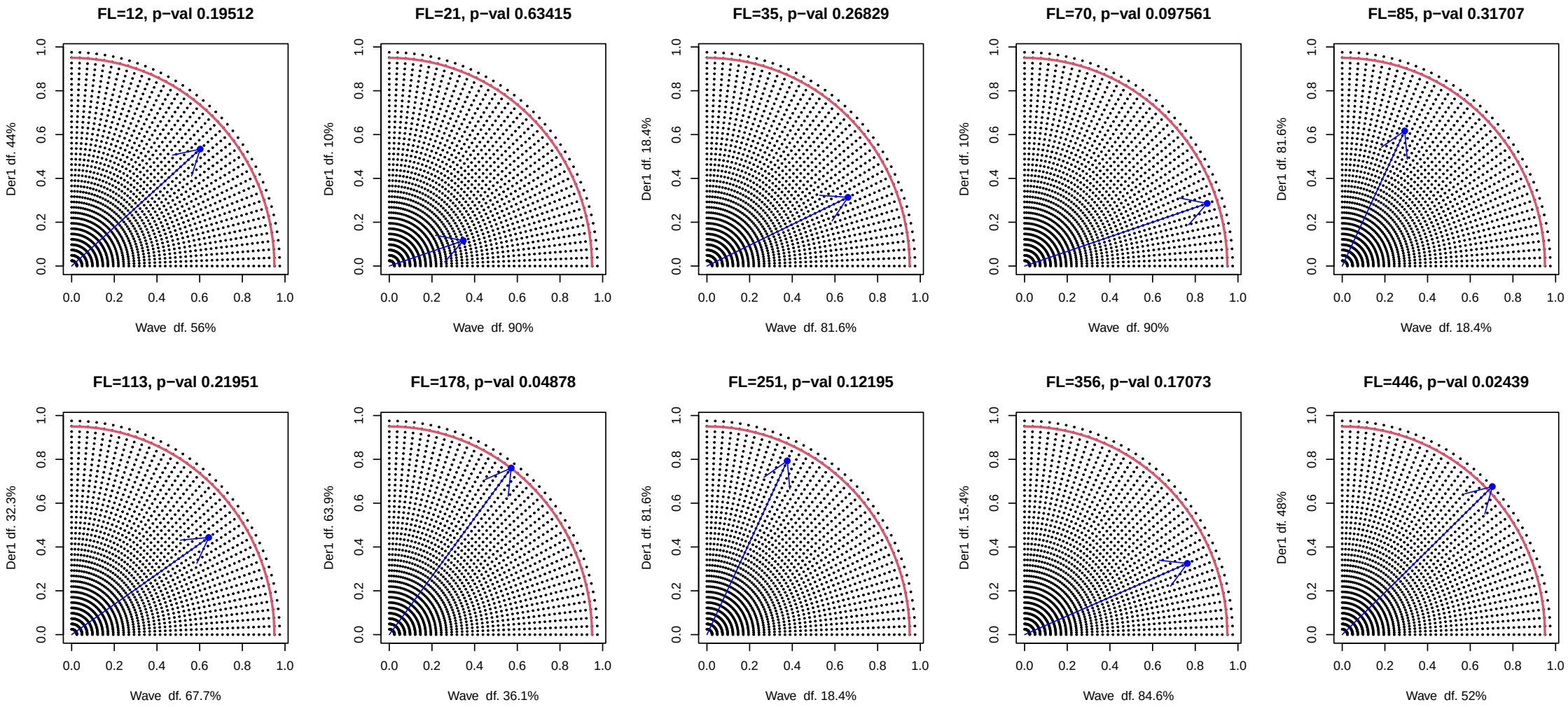

Table for ERG wave by sex in btime–72 – asd subgroup

	pval-erg-b72	wave perc	der1 perc
12	0.1951		
21	0.6341		
35	0.2683		
70	0.0976		
85	0.3171		
113	0.2195		
178	0.0488	36.1	63.9
251	0.1220		
356	0.1707		
446	0.0244	52	48

3.13 OP wave by age in 0–60 – ctr subgroup

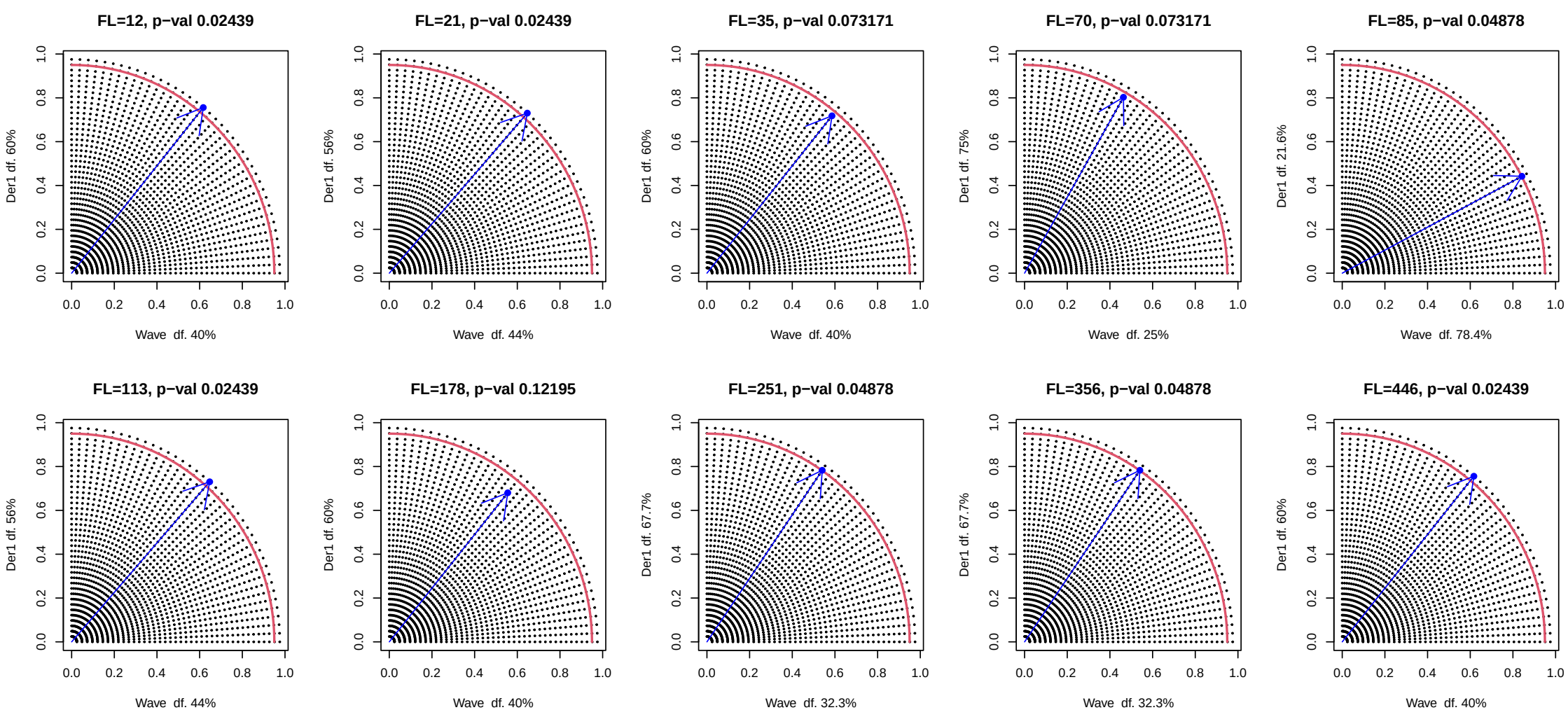

Table for OP wave by age in 0–60 – ctr subgroup

	pval-op-060	wave perc	der1 perc
12	0.0244	40	60
21	0.0244	44	56
35	0.0732		
70	0.0732		
85	0.0488	78.4	21.6
113	0.0244	44	56
178	0.1220		
251	0.0488	32.3	67.7
356	0.0488	32.3	67.7
446	0.0244	40	60

3.14 OP wave by age in 0–60 – asd subgroup

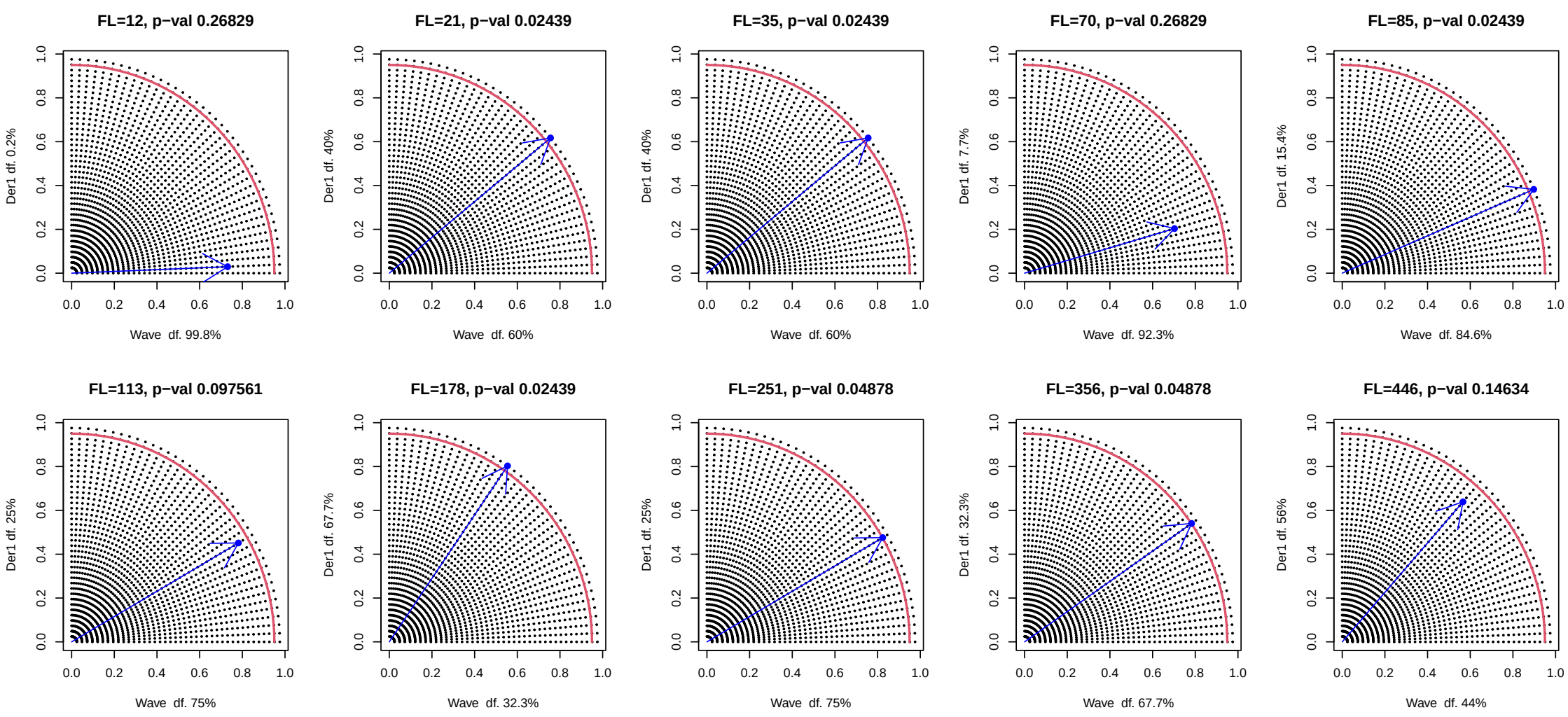

Table for OP wave by age in 0–60 – asd subgroup

	pval-op-060	wave perc	der1 perc
12	0.2683		
21	0.0244	60	40
35	0.0244	60	40
70	0.2683		
85	0.0244	84.6	15.4
113	0.0976		
178	0.0244	32.3	67.7
251	0.0488	75	25
356	0.0488	67.7	32.3
446	0.1463		

3.15 ERG wave by age in 0–atime – ctr subgroup

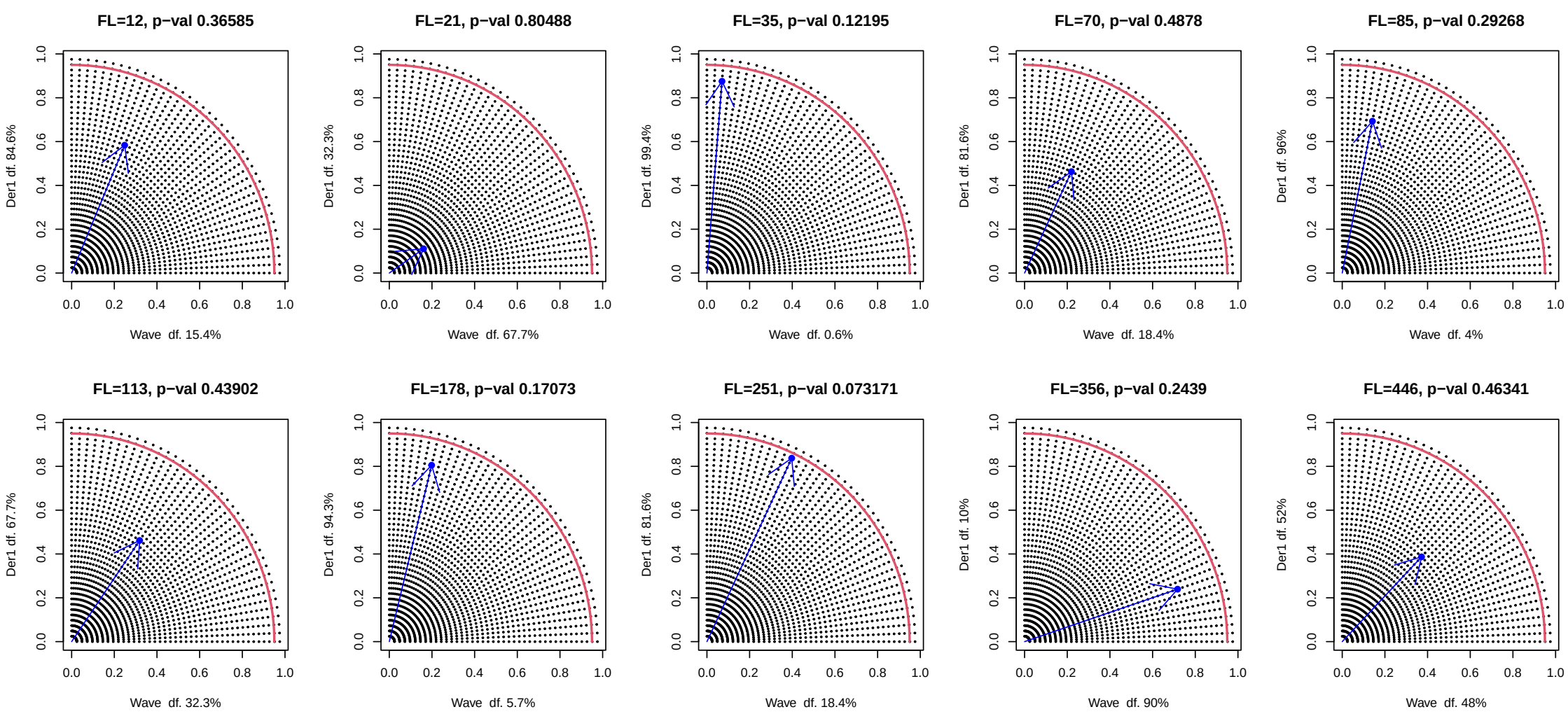

Table for ERG wave by age in 0–atime – ctr subgroup

	pval-erg-0a	wave perc	der1 perc
12	0.3659		
21	0.8049		
35	0.1220		
70	0.4878		
85	0.2927		
113	0.4390		
178	0.1707		
251	0.0732		
356	0.2439		
446	0.4634		

3.16 ERG wave by age in 0–atime – asd subgroup

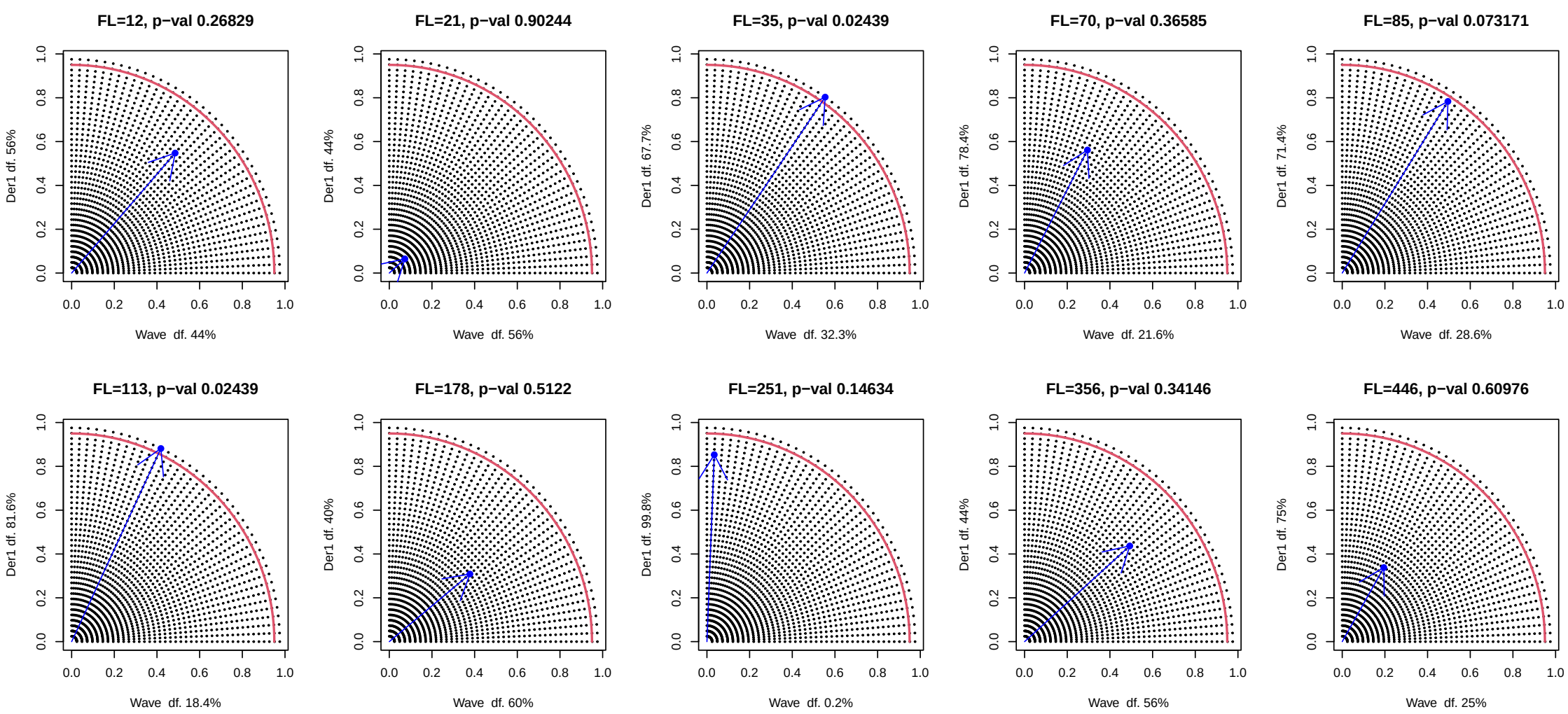

Table for ERG wave by age in 0–atime – asd subgroup

	pval-erg-0a	wave perc	der1 perc
12	0.2683		
21	0.9024		
35	0.0244	32.3	67.7
70	0.3659		
85	0.0732		
113	0.0244	18.4	81.6
178	0.5122		
251	0.1463		
356	0.3415		
446	0.6098		

3.17 ERG wave by age in atime–btime – ctr subgroup

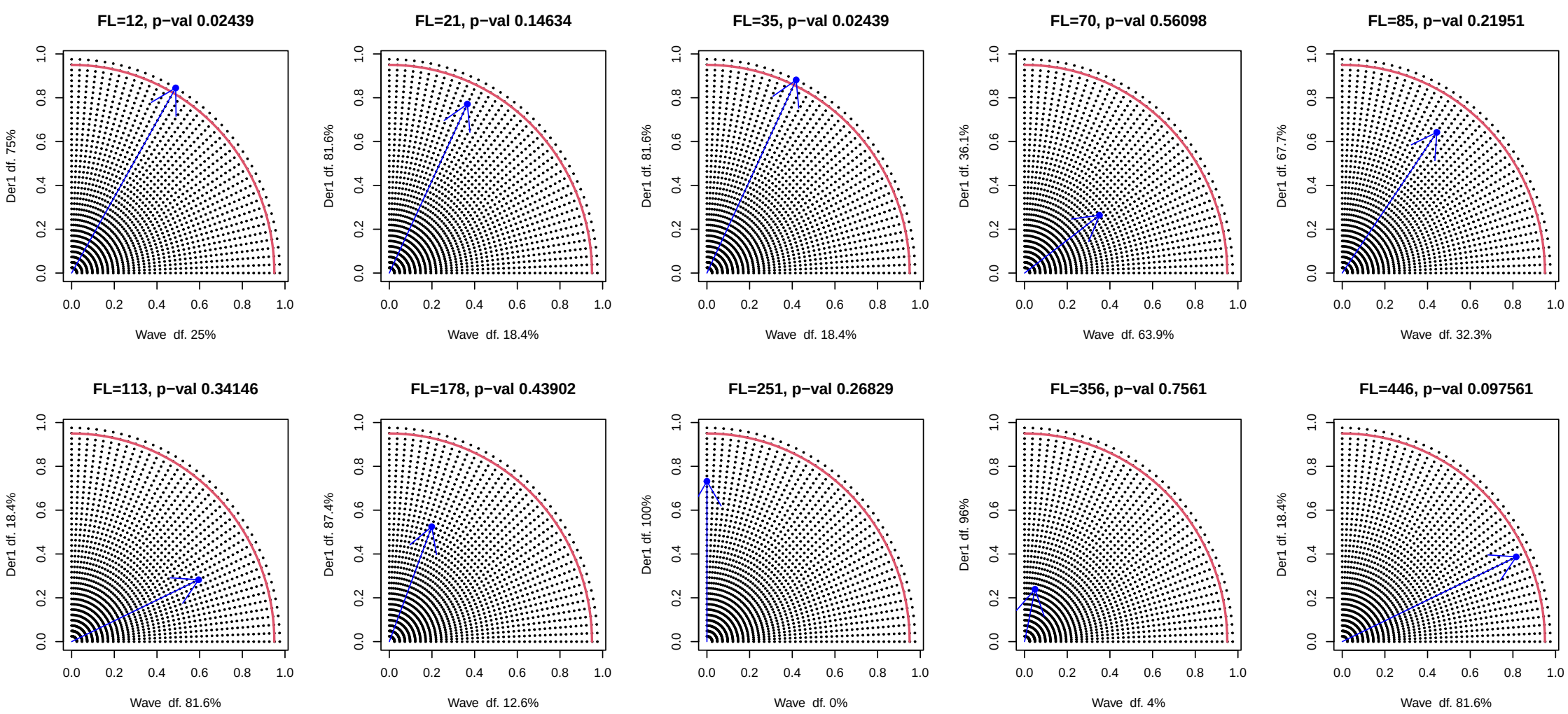

Table for ERG wave by age in atime–btime – ctr subgroup

	pval-erg-ab	wave perc	der1 perc
12	0.0244	25	75
21	0.1463		
35	0.0244	18.4	81.6
70	0.5610		
85	0.2195		
113	0.3415		
178	0.4390		
251	0.2683		
356	0.7561		
446	0.0976		

3.18 ERG wave by age in atime–btime – asd subgroup

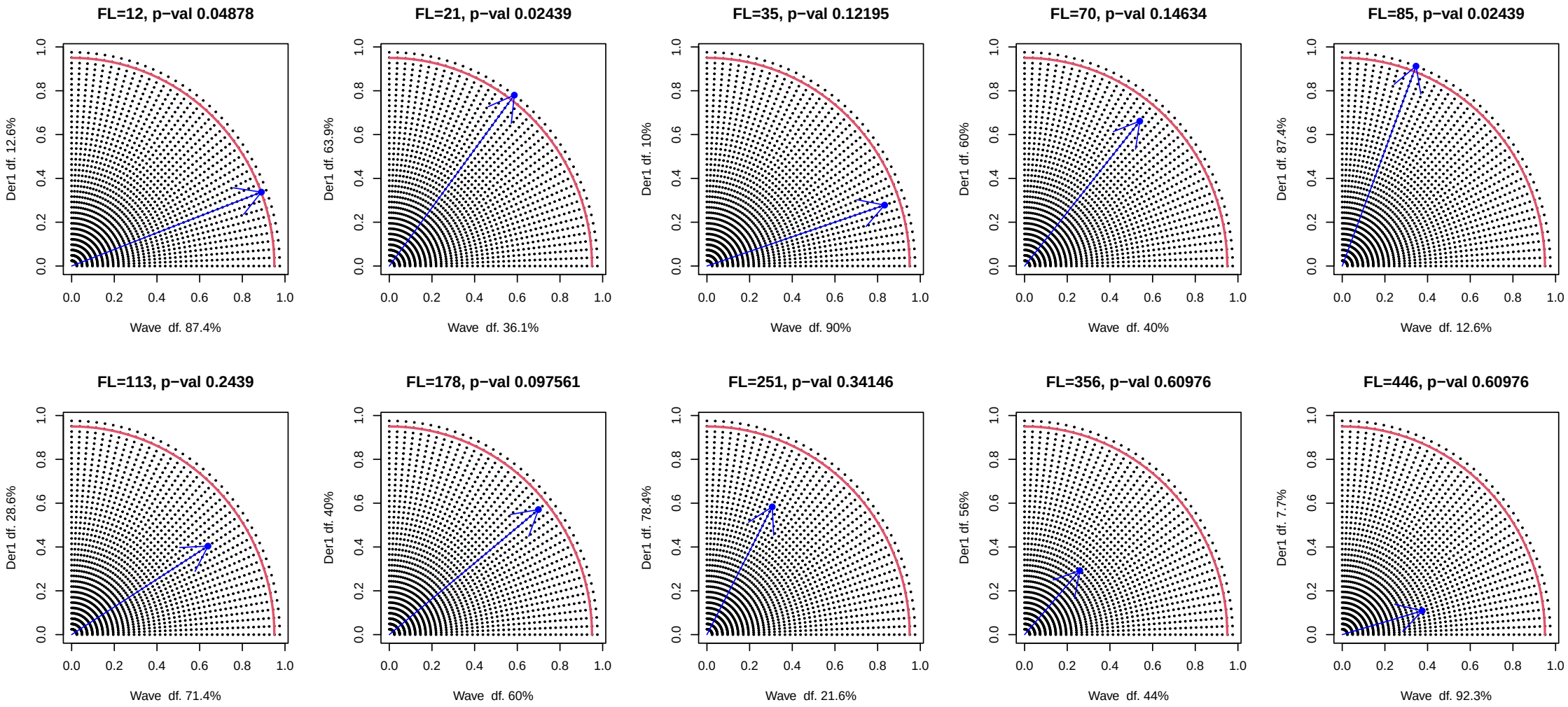

Table for ERG wave by age in atime–btime – asd subgroup

	pval-erg-ab	wave perc	der1 perc
12	0.0488	87.4	12.6
21	0.0244	36.1	63.9
35	0.1220		
70	0.1463		
85	0.0244	12.6	87.4
113	0.2439		
178	0.0976		
251	0.3415		
356	0.6098		
446	0.6098		

3.19 ERG wave by age in btime–72 – ctr subgroup

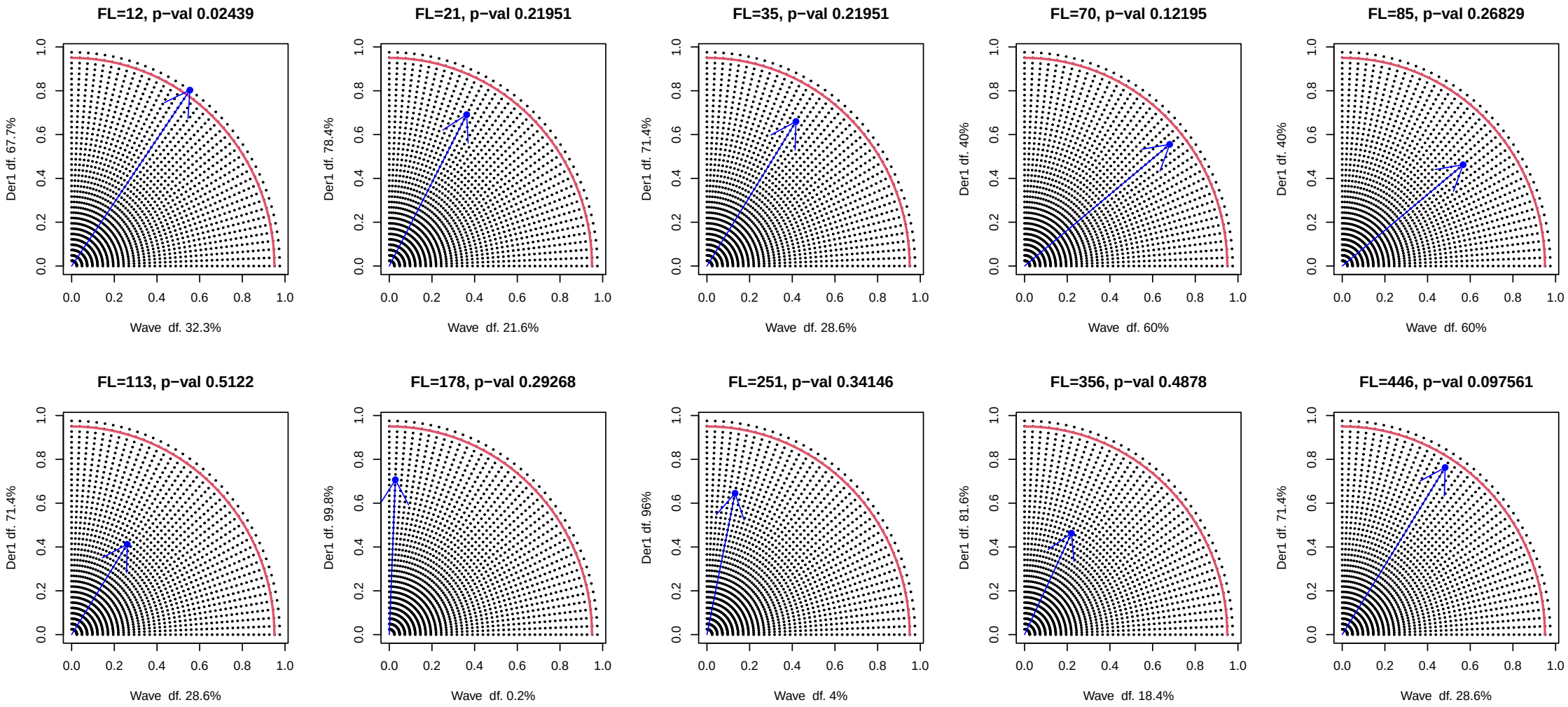

Table for ERG wave by age in btime–72 – ctr subgroup

	pval-erg-b72	wave perc	der1 perc
12	0.0244	32.3	67.7
21	0.2195		
35	0.2195		
70	0.1220		
85	0.2683		
113	0.5122		
178	0.2927		
251	0.3415		
356	0.4878		
446	0.0976		

3.20 ERG wave by age in btime–72 – asd subgroup

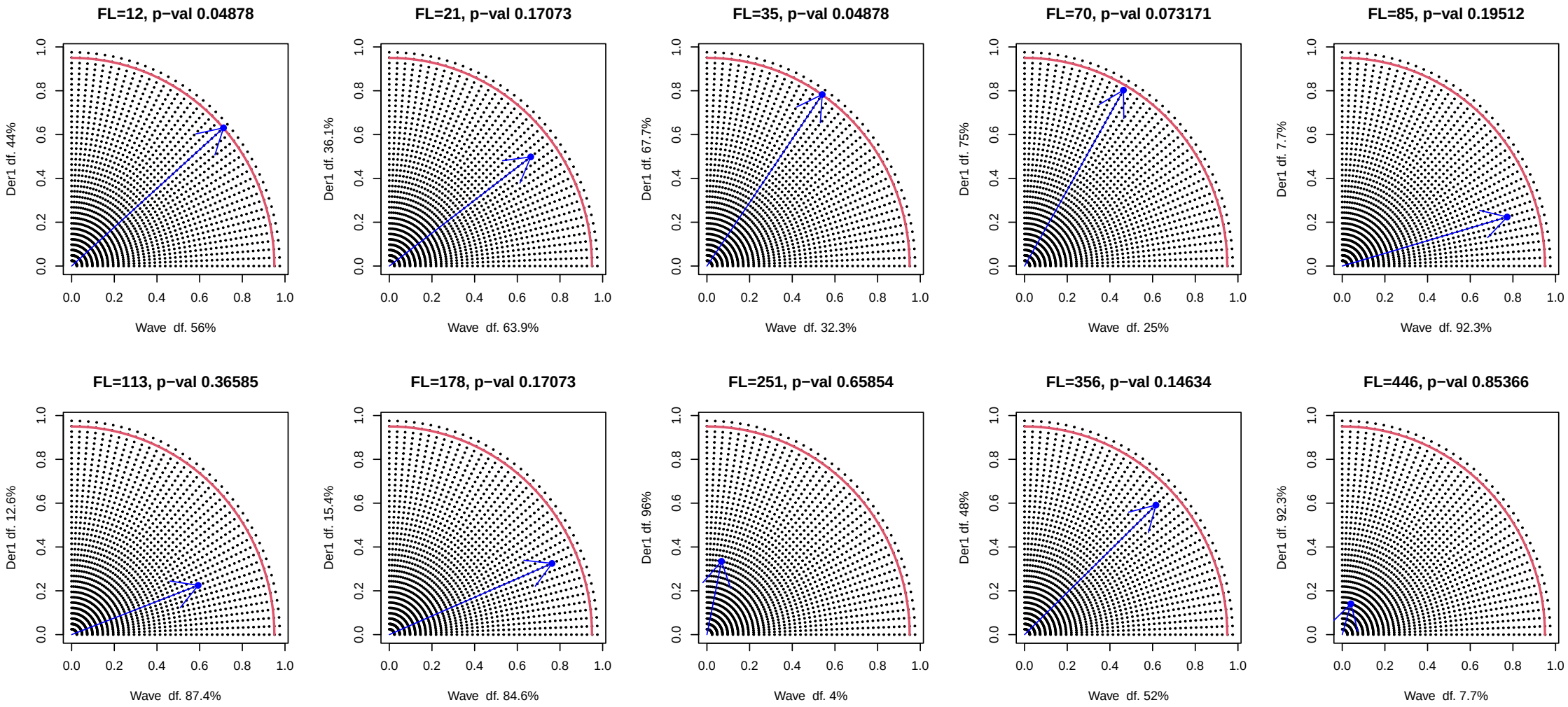

Table for ERG wave by age in btime–72 – asd subgroup

	pval-erg-b72	wave perc	der1 perc
12	0.0488	56	44
21	0.1707		
35	0.0488	32.3	67.7
70	0.0732		
85	0.1951		
113	0.3659		
178	0.1707		
251	0.6585		
356	0.1463		
446	0.8537		
